# Measuring attitudes towards biology major and non-major: Effect of students’ gender, group composition, and learning environment

**DOI:** 10.1371/journal.pone.0251453

**Published:** 2021-05-14

**Authors:** Firas Almasri, Gertrude I. Hewapathirana, Fatme Ghaddar, Nick Lee, Bashar Ibrahim

**Affiliations:** 1 Centre for Education Studies, University of Warwick, Coventry, United Kingdom; 2 Department of Mathematics and Natural Sciences, Gulf University for Science and Technology, Hawally, Kuwait; 3 College of Business, Gulf University for Science and Technology, Hawally, Kuwait; 4 Department of Computer Science, Gulf University for Science and Technology, Hawally, Kuwait; 5 European Virus Bioinformatics Center, Jena, Germany; 6 Department of Mathematics and Computer Science, University of Jena, Jena, Germany; University of Macau, MACAO

## Abstract

This study examined the effect of collaborative learning (CL) versus traditional lecture-based learning (TL) pedagogies and gender group composition in effecting positive or negative attitudes of biology major and nonmajor men and women students. The experimental research method was administered in experimental and control groups to test the hypotheses. Students’ attitudes refer to their positive or negative feelings and inclinations to learn biology. A nine-factor attitude scale was administered in (1) single-gender nonmajor biology, (2) mixed-gender nonmajor biology, (3) single-gender major biology, and (4) mixed-gender biology major groups. Men (221) and women (219) were randomly assigned into single and mixed-gender classes without groups and single-gender groups (4M) or (4W) and mix-gender (2M+2W) groups. In CL nonmajor and major single-gender groups, women demonstrated significantly higher positive attitudes than men. In contrast, men’s attitudes were significantly improved in mixed-gender CL groups for major and nonmajor sections, and the effect size was larger in mix-gender classes. Women feel less anxious in single-gender groups but more anxious in mixed-gender groups. In mixed-gender groups, men’s self-efficacy, general interest, and motivation enhanced significantly; overall, men experienced greater satisfaction and triggered their desire to collaborate better, affecting all nine attitudinal factors. There was an interaction effect demonstrating the teaching pedagogy’s impact on improving students’ attitudes toward biology; students’ gender and gender-specific group composition have been the most influential factor for nonmajor students. These findings suggest that there is a need for developing gender-specific and context-specific learning pedagogies, and instructors carefully select gender grouping in teaching undergraduate science subjects.

## Introduction

Science education has gained renewed interest and prominence today to fulfill the increasing demand for future medical practitioners and scientists and minimize the increasing gap of the skilled workforce [1–4]. Despite the growing importance of science education, many gaps and problems exist that need extensive research to explore underlying causes to propose remedial measures to attract students and cultivate positive science education attitudes [1, 5–7]. A few examples of such gaps are declining interest, high dropouts, a lack of career and grade motivation, low self-efficacy and self-determination, escalating assessment, and learning anxiety. Diminishing interest in taking over science-related careers, increasing gender inequity on enrollment, and academic achievement has been the major concerns for many universities and nations worldwide [8–12]. Especially, increasing gender inequity, diminishing minority and women enrollment at higher education level, "a widespread scientific ignorance in the general populace," rising importance of "economic utility of scientific knowledge" are a few that signify the importance of research to initiate remedial measures to boost positive attitudes toward science careers [13, 14]. In this endeavor, Biology education comes to the forefront of medical and care practitioner fields [15, 16]. Among many discussions about generating interest and increasing enrollments at higher education levels, students’ attitudes have become the center of attention to cultivating positive attitudes towards science education [10, 17]. Despite a few studies that assessed secondary school students’ attitudes in general science [18], there is a lack of research measuring biology education attitudes at the undergraduate level [19].

Research shows a high correlation between students’ attitudes towards academic achievement and a predictor of selecting science subjects and future careers [18, 20]. A few studies found that academic performance, self-efficacy, and motivation are linked to the outcomes of (STEM) science, technology, engineering, and mathematics education [21–23]. Hagay et al. (2012) posit that context-specific factors such as religion and culture have a higher effect on generating students’ interest, and therefore, developing interest-based teaching materials reduces the gender gap in biology in Islamic societies [24]. Russel and Hollander (1975) state that general interest is expressing positive feelings towards biology, and therefore, measuring general interest is a fundamental element of measuring attitudes.

Among many factors, students’ attitudes have been proven as the major drawback to promoting, attracting, and retaining students in science education [8]. Students’ motivation has a well-established connection with their attitudes, achievement, and interest in biology [25–27]. Despite the existence of research that assessed attitudes towards science [1, 8, 9, 28–36], there is a lack of research that focuses on measuring whether specific teaching pedagogies that lead to curriculum changes could generate students’ positive attitudes towards learning science subjects. The science education research literature suggests that negative attitudes towards science subjects such as biology have some correlational effects on low interest and motivation, low enrollment, negative perceptions, and low performance. Most of such research assessed attitudes towards science but not how a pedagogical method effectively cultivates students’ attitudes towards biology [37]. Therefore, we hypothesize using appropriate pedagogical methods; instructors would possibly generate positive attitudes and feelings towards learning biology affecting general interest, grade and career motivation, self-efficacy, and self-determination while reducing the anxiety of learning, eventually results in generating overall positive attitudes towards science careers. There is a lack of studies that examine the effect of pedagogical approaches on enhancing students’ attitudes [1, 38]. Further, not many studies analyze the differences and multi-dimensions of men and women’s attitudes in CL versus TL classroom based on gender composition and gender-specific grouping, mixed-gender vs single-gender classes specifically for nonmajor and major biology students, which gives our study a unique perspective to develop the research foundation. The subsequent sections include a literature review, theoretical frame, the research aims and hypothesis, methodology, results, a discussion followed by limitations, future research, and conclusion.

### Literature review

Assessing students’ attitudes have been suggested to be an urgent research agenda in science education [10] because students’ attitudes have been found to be an important predictor of not only academic achievements but also a determining factor of measuring the effectiveness of specific pedagogical approaches such as collaborative learning vs traditional lecture methods [8, 39] where attitudes are central to understanding their behavior [19]. However, based on the literature review by Chaiklin (2011), the relationship between attitude and behavior has been a conflicted path for years, and researchers have had a conflicted correlation in results on whether attitude influences behavior [40]. Gasiewski et al. (2012) suggest students’ attitudes play a critical role in enrollment and retention decisions in science education and future career intentions. Similarly, Fareo (2019) mentioned that the student’s attitude in secondary level schools influences the way he/she studies, grades achievements, and involvement in the overall subject in the future [41]. Tsybulsky et al. (2018) found students’ attitudes improve general interest in future careers in science in the same vein.

Researchers examined various factors such as gender, culture, and societal factors that shape students’ attitudes towards science education [42]. Nevertheless, a few studies assessed attitudes towards learning science subjects such as biology majors and nonmajors [6, 43]. Lovelace and Brickman (2013) suggested assessing students’ attitudes towards learning science subjects would help understand the underlying factors of decreasing interest and enrollment in biology majors and nonmajors [1]. Due to inadequate research on assessing the effectiveness of specific teaching pedagogies such as CL and TL in generating positive attitudes and interests within students towards science subjects, we cannot understand the underlying factors and their influence over generating positive or negative attitudes [7, 44]. Our literature review unveiled that even though for the last 20 years, researchers eyes have been laid widely on the CL pedagogy [44–47] and the impact of co-education with various gender compositions in groups [48–52], where studies suggest that the gender composition can largely influence the learning outcomes in a CL environment.

Measuring the effect of the state-of-the-art teaching pedagogies in shaping students’ attitudes towards biology education is rather blurred because researchers often found contradictory findings [53–56]. While a few studies assessed various factors such as gender, culture, and societal factors that shape students’ attitudes towards science education, and attitudes towards learning science subjects in majors and nonmajors, there is a vacuum of research to understand whether specific teaching approaches can boost renewed interest in science careers [1, 6, 43].

Previous studies and large-scale meta-analysis suggest that when students work together in a CL environment, they accomplish shared learning goals compared to TL [46, 57, 58]. When students are assigned group work that includes problem-solving, they are expected to work together and discuss them as a group [59]. CL offers a wide range of benefits to students when implemented in a class, such as leadership, decision-making, communication, teamwork, and conflict management skills [60]. Marquez (2017) found that students performed better and fostered positive attitudes in CL groups than in a lecture-based TL environment [61]. A study of undergraduate biology students in Indonesia found that CL enhances students’ motivation and collaborative team skills [60]. Similarly, Crawford (2000) found that when educators collaborate with the students and guide scientific inquiries, students’ motivation increases [62]. A study of engineering university students in Panjab found that CL increases the students’ independence, responsibility, confidence, motivation, skills, and positive interdependence [63]. A modeling instruction (MI) approach integrated with CL with small group activities showed decreased self-efficacy, regardless of ethnicity and gender [64]. Another research on nonmajor biology undergraduate class that used CL was positively affecting the usefulness and utility of biology education for students’ future careers; however, students’ self-efficacy towards biology was neutral [25]. When high school students in science classes were taught using CL, it was concluded that CL increased students’ interest, and the way the subject is being taught matters in forming positive or negative attitudes [61]. Tsybulsky et al. (2018) posit students’ attitudes are important as they predict the future selection of science courses and are context-dependent. Likewise, literature cites many merits of studying students’ attitudes [18].

Gender gaps have been witnessed for a long time until now, along with the man superiority in the STEM careers [65]. Previous research revealed that gender gaps are socially significant and closely related to cultural variations in opportunity structures for girls and women, as indicated by cross-national data analysis [65–67]. Despite Wang’s (2013) findings that women outperform men in primary and secondary schools, their interests diminish when they reach undergraduate, and graduate levels resulting in women underrepresented in STEM fields [23, 42, 68–70]. Researchers found that low enrollment of women in science education is due to gender-specific perceptions and attitudes, social and ethnicity beliefs, stereotypical stigmas attached to women’s roles in STEM careers, context-specific and socially accepted day-to-day life-related roles of men and women, and culturally rooted beliefs [71, 72]. Moreover, in biology, 60% of undergraduate students are women on average, assuming no gender inequality. However, it has been observed [73, 74] that there are gender disparities in participation in biology classrooms [53, 75, 76]. Another argument is that women’s low enrollment can be due to various subjects’ meanings and perceptions as masculine and feminine subjects that determine their future career trajectories [77, 78].

On the contrary, Telli et al. (2005b) found that students’ attitudes towards biology did not influence their interest and involvement in future careers, where students found learning biology fun but did not consider it for future careers. However, a strong correlation was found between students’ perceptions of biology and the class environment, such as teaching methods and teacher involvement in the class [79]. According to Hong (2011), in Asia, educators should be instructed on designing assessments that promote student-centered learning practices [80]. Some of the critical factors that lead to low enrollment, poor academic achievement, and a higher level of gender disparity in science education have been heavily discussed in relation to academic achievements and access to science education in different contexts as those researchers ignore the attitudinal factors [1, 5, 6], Among many factors, students’ negative attitudes have been proven as the major drawback to promoting, attracting, and retaining students in science education [8]. Having analyzed previous research, we found gender is an important variable in assessing attitudinal differences towards CL and TL in biology major and nonmajors that would be useful for educators in selecting appropriate pedagogies for men and women in various contexts.

Osboren et al. (2013) criticize that the attitude dimension of science education has been poorly defined and not yet recognized as a critical aspect of answering an important question why there is a lack of students’ interest towards science education while science educators’ thrust to increase students’ interest in science subjects [81]. Despite a few research that assessed attitudes towards science [1, 8, 32], there is a lack of research on measuring whether specific teaching pedagogies could generate positive attitudes towards science subjects. Decreasing interest in science education and increasing the supply and demand gap for future healthcare professionals and scientists are global concerns that need research to understand underlying causes in different contexts.

The attitude dimension has been described as a multifaceted concept that is hard to observe and assess [82]. For example, attitudes have cognitive (feelings, beliefs, and ideas), affect (likes, dislikes), and behavioral (tendency to take action) components [56, 81]. Reid (2006, p. 6) posits that attitude construct includes cognitive, affective, and behavioral dimensions in the same vein. Therefore, attitude measures need to have factors such as individuals’ psychological sensitivities, emotions, evaluative judgments, feelings about important and unimportant, pleasant or unpleasant, good and bad, and so on [81, 83]. Students’ attitudes also defined as individuals’ psychological reaction towards an object or things they encounter; depending on students’ psychological inclination or beliefs, it can generate either positive or negative force that influences students’ behavior [81], “feelings or interest of learners towards science” [37], “attitudes towards science are the notions and images created in the minds of learners by interacting directly with multiple situations (p. 67).” While there are contrasting criticisms about various definitions and measurements of the attitude construct in various disciplines [81], George (2000) states that the attitudinal dimension has engrained affective dimension and therefore assessing feelings, enjoyments, motivation, general interest and so on are essential to understand students’ true attitudes. Thus, attitudes have an evaluative judgment dimension; for example, a positive or negative experience in school activities potentially influences learner attitudes [82]. Researchers suggest that attitudes in science learning can be measured in terms of students’ values, beliefs, or perceptions about teaching methods or activities, curriculum, outcome expectancy, motivation, or individuals’ interest, values or rationality they attach to specific things or objects [8].

Measuring students’ attitudes would help educators understand why some students participate and why others do not have an interest in specific science subjects [8] to forecast and plan for the future scientific workforce [84, 85]. Researchers found a significant correlation between students’ attitudes and behavioral intentions, and therefore attitudes can generate a desire to enroll in science courses [86]. Though some researchers argue that there is a positive correlation between attitudes and achievements, some others argue that even poor performers can have positive attitudes towards science; therefore, those students still may enroll in science courses [87, 88]. It is evident that research on assessing students attitudes are limited to a few variables such as teacher perception, anxiety and concerns toward science, enjoyment of learning styles, experience and self-respect at science, motivation and interest towards science, classroom environment, achievement and fear, lack of success in science courses, attitudes of friends, peers, groups, parents’ attitudes towards science and attitudes towards teacher gender and so on [10]. Therefore, measuring the correlation between attitudes and achievements alone does not clarify why some students like and others dislike science subjects or what causes them to pursue or not pursue science education or scientific careers.

### Theoretical framework, study goals, research questions and hypotheses

For this research, we selected collaborative learning and lecture-based learning pedagogies as the study’s main focus, gender and gender group composition as independent variables, and the attitude as a dependent variable to measure pedagogies’ effect on improving students’ attitudes towards biology major and nonmajor. Collaborative learning is a learning strategy that involves groups of students working together to solve problems and learn new concepts [89]. In contrast, in traditional learning, students explore content by posing, examining, and answering questions under the instructor’s direct guidance [90–92]. A few studies posit that the 7E instructional and collaborative learning methods are more effective than traditional teaching biology lectures [37]. While there are discussions of the relationship of attitudes and learning achievements, there is little research that assesses the potential capability of specific teaching pedagogies such as collaborative learning (CL) and traditional lecture-based learning (TL) in terms of fostering interest, generating positive feeling towards learning biology, grade and career motivation and reducing learning anxiety among men and women [8, 19].

Researchers raised their concerns about the difficulty of assessing the attitudinal construct due to its complexity [81]. Therefore, it needs a carefully developed measurement scale that can capture most of the affective, cognitive, and emotional dimensions. For our study, we selected two attitudinal scales developed by Glynn et al. (2011) to assess the students’ attitudes towards science (SATS) and a biology attitude scale developed and validated by Russel and Hollander (1975) and customized to biology education of the current study [32, 87]. Glynn et al. (2011) contended student motivation to learn science is part of the attitude that generates a desire to learn science. They validated an instrument to measure five motivation components: “intrinsic motivation, self-determination, self-efficacy, career motivation, and grade motivation” in science majors and nonmajors, compared with men’s and women’s attitudes (p.1159). They suggest that the instrument is validated and an effective tool to assess students’ motivation to learn science. Russel and Hollander (1975) posit that assessing students’ attitudes towards biology can influence curriculum developers to determine potential teaching approaches to achieve cognitive goals, as they emphasize that it is important for instructors to understand students’ attitudinal characteristics [32]. They emphasize an attitude scale should have the capability to differentiate among various levels and shades of attitudes such as strongly favorable or agreed to strongly unfavorable/disagree. Thus they suggested using a Likert-like scale to assess specific statements related to each dimension of attitudes. A detailed description of the nine dimensions is given in the method section.

### The study goals

This study aims to measure CL vs TL pedagogies’ effects and the impact of gender grouping on men’s and women’s student’s attitudes towards undergraduate biology majors and nonmajors in Kuwait’s university environment. We focus on the following research questions:

1. Is there any difference between women’s and men’s attitudes towards biology implementing CL vs TL in major and non-major biology classes?
2. How do single-gender and mix-gender grouping affect women’s and men’s attitudes towards biology implementing CL and TL in major and non-major biology classes?
3. Is there a difference between the CL (experimental) vs TL (control) sections in terms of attitude towards biology in major and non-major classes?

As per Glynn et al. (2011), general interest and motivation are classified as individuals’ feelings, and therefore, improving general interest towards biology are included as separate statements in the attitude measurement scale. Kuwait undergraduate classroom learning is unique as it encourages single-gender classes and rarely has mixed-gender classes [93]. Often science majors and nonmajors are taught separately using a different curriculum. Therefore, for our study, we incorporated single and mixed-gender variables along with major and nonmajor as important dimensions of this research to measure the similarities and differences of attitudes of men and women. Based on an extensive literature review and for that purpose, we used an experimental study using four different groups (a) single-gender nonmajor biology, (b) mixed-gender nonmajor biology, (c) single-gender biology major, and (d) mixed-gender biology major in undergraduate introductory biology classes and administered a nine-factor attitudes scale to test the four hypotheses according to the research questions:

HO1: In non-major single-gender classes, there will be a significant difference between men/women students’ attitudes towards biology who have participated in CL and those who have participated in TL methods.
HO2: In non-major mix-gender classes, there will be a significant difference between men/women students’ attitudes towards biology who have participated in CL and those who have participated in TL methods.
HO3: In major single-gender classes, there will be a significant difference between men/women students’ attitudes towards biology who have participated in CL and those who have participated in TL methods.
HO4: In major mix-gender classes, there will be a significant difference between major biology men/women students’ attitudes towards biology who have participated in CL and those who have participated in TL methods.

### Variables

Independent variables of the study consisted of 1) teaching method used in class (TL vs CL method) 2) students’ gender (man & woman), 3) gender group composition (single-gender classes, mix-gender classes), biology (major, nonmajor). The instrument includes nine attitudinal dimensions as described in the method section. Student’s responses for each factor in the given questionnaire were calculated.

## Methodology

### Participants selection

Participants were enrolled in an introductory biology course at a university in Kuwait. Biology non-major students (264) and major students (252) were assigned randomly from program administrative databases into CL groups single-gender and mix-gender classes, and have the same chances of being in any group to eliminate any differences. We used simple random sampling wherein every student in the population can be selected for any section. After having a list of the full biology student population in an excel sheet, we used the random number method to assign every student a random number by random number generator in Microsoft Excel. Then randomly pick a subset of the population for each section. The random assignment gives advantages over preventing external validity and non-equivalent groups in an experimental study [94]. The participants were: a) Nonmajor undergraduate biology students having a biology course as an elective, b) Major biology students having biology as a pre-request course for their pre-medical or pre-dental school. The students admitted to the program were in an age range between 18–22 years. They were in their first year, and it has been confirmed from the participant’s survey response. The biology course goal was to develop a conceptual understanding of basic biology and to be able to apply students understanding to solve biological questions applying the same curriculum.

### Ethics statement

The researchers got approval from the Humanities & Social Sciences Research Ethics Committee (HSSREC) at the University of Warwick to carry out the study that follows the British Educational Research Association (BERA) [95]. To ensure as much group equivalence as possible among the treatment groups (i.e., identical classrooms, resources, and curriculum), the researchers ensured no objection to the study on ethical grounds, thus allowing them to carry out the study at the College. Moreover, the authors obtained informed written consent from the participants where the students read the study participation form and were free to ask any questions regarding the research. If they agreed to participate, they signed a consent form before taking part in the study, and it follows national regulations regarding personal data protection. Participants were comforted that their anonymity would be respected. Besides, it was made clear that no recriminations would occur irrespective of their choice.

### Attitude instrument

We adopted validated attitude scales by Russel and Hollander (1975) and Glynn et al. (2007) and later validated by Aydeniz and Kotowsk (2014). In this quantitative research, the researcher used a questionnaire to sample the population; The questionnaire used to reflect population perceptions, emotions, beliefs, behaviors, or unique features [96]. To indicate individual preferences accurately, using an instrument that includes statements where the individual can express the degree of agreement on the scale is the best practice. Therefore Lovelace et al. (2013) recommended using Russell and Hollander’s scale for research work [1]. The questionnaire was divided into two sections. Section 1: *Feeling toward biology questionnaires* taken from Russel and Hollander (1975) and Section 2: The questionnaire *Students’ Attitudes Towards science* (SATS) modified to biology course (SATB) taken from Glynn (2007). Moreover, biology teachers independently revised the questionnaire to ensure its validity and modified it to assess students’ attitudes towards biology learning. A copy of the questionnaire is included in the S1 Questionnaire. The questionnaire was in English and consisted of section # 1 includes (14 questions) about *feeling toward biology*, and section #2 has 8 sub-scales (48 questions) that included: *general interest* (1–5), *motivation towards learning biology* (6–15), *benefit and utility of biology* item (16–20), *career motivation* (21–25), *self-efficacy in biology learning* (26–33), *self-determination* (34–38), *grade motivation* (39–43) and *assessment anxiety* (44–48).

### Attitude scales measures and procedures

By the end of the semester, the students received an e-mail with the link to the electronic questionnaire that had a valid 62 statements with nine sub-scales. Qualtrics was used to administer the responses, and participants completed it online through the distribution of anonymous, protected links to the protocol that allowed secure data collection. The researcher explained each part of the questionnaire’s details to the students. Students responded to each question randomly on a five-point Likert-type scale of temporal frequency ranging; Strongly Disagree having a rate of 1(negative statements), Disagree having a rate of 2, Neither Agree nor Disagree having a rate of 3, Agree having a rate of 4, Strongly Agree having a rate of 5(positive statement). If a student did not understand a particular item, the researcher provided feedback individually. All questions were marked as required to avoid missing data, and the questionnaire could not be submitted unless all questions had been answered. Informed consent was obtained from the students following the ethical guidelines. The anxiety about science assessment items was reverse-scored when added to the total, so a higher score on this component means less anxiety, negative attitude items treated the same way. We confirmed the reliability and consistency of the tools used for the study. A Cronbach’s alpha above 0.70 is considered an acceptable value [95], with considerably lower values indicating a lack of reliability. According to Sekaran (2004), the values of Cronbach’s Alpha for each variable of the questionnaire and the entire questionnaire should exceed 0.70 to consider the result acceptable.

The nine factors mentioned in the questionnaire in total as the following:

1. *Feelings towards Biology*: Feeling towards biology is assessed using questions for 14 items. The questionnaire had statements such as "I do not like biology, and it scares me to have to take it" and "Biology is interesting to me." Based on psychological studies, the student’s feelings play a major role in a subject’s performance as emotions influence consciousness evolution and mental operations [104]. Moreover, students’ feelings towards a subject were associated with the classroom’s learning environment and how they portrayed subjects like Biology [105, 106].
2. *General Interest*: General interest in learning biology: individuals’ interest can be a sign of motivation that stimulates interest to learn biology by asking questions and actively participating in the class [97]. The instructor can understand why students strive to learn science, their emotions, and what specific teaching can stimulate students’ interest. "I like watching biology-related TV"; "I like reading about famous biologists" are some of the statements that the students were surveyed on. Understanding the students’ interest in a subject is essential. It acts as an important factor because it plays a major role in studying science and increasing their involvement with science in graduate studies [107]. Since students have a low interest in science than other subjects, the teaching environment was found to influence the students’ interest and increase the chance that students enroll in science courses in the future [108, 109].
3. *Motivation towards learning Biology*: Motivation is defined as the psychological concept that influences the behavior and effect on a person’s activities [110]. An intrinsic motivation that brings the joy of learning biology, including various motivational components, generates enjoyment of learning [87, 98]. Happy feelings will help gain higher self-confidence and motivation to learn [99]. Statements include "I will ask my teacher for an explanation if I do not understand the science topic." and "Getting a good grade in biology is important to me" were some of the statements included in the questionnaire to test the students’ motivation in biology. This factor is essential to survey students as it was found to have a strong connection with students’ attitudes when studying biology and their achievement rates [46–48]. Like previous factors, the teaching method was also highly influential towards the students’ motivation for studying biology [46].
4. *Benefits and Utility of Biology*: This aspect can potentially lead to a feeling of valuable things to do [92]. The benefits and utility are important factors linked to the students’ understanding of biology in class and the outside world. "What I learn in my biology class helps me to understand how things work in life." and "I use the biology that I learn in school in my life" were some of the statements included in the questionnaire. According to research studies, students studying science have low relevance to what they study in class that benefits real life. However, nonmajor biology students were found to have some relevance in topics related to biology, particularly in cellular biology application in real life [111–113].
5. *Career motivation*: The motivation to have a career in a specific domain is defined as the psychological process that helps regulate the direction and determination of the students’ learning behavior [114]. It enhances the desire to pursue a future career; it is an attribute of motivation [100] and a tangible aspect of a future career trajectory. Since career motivation was found to have a strong relationship with students’ attitude and interest in science, it is reasonable to include career motivation as a factor where statements like “Knowing biology will give me a career advantage.” and “My career will involve science” were surveyed by the students for further understanding of the influence of the factor. Previous studies suggest that students have a strong desire to study biology due to future career plans that involve the subject and vice versa [48, 107, 115].
6. *Self-efficacy*: The term self-efficacy is defined as the persons’ belief that she/he can execute certain actions as required by an incident [116]. Self-efficacy was associated with students’ grades, persistence, confidence, and motivation towards a subject [93, 117–120]. Therefore, the biology questionnaire for self-efficacy targeted students’ confidence towards biology learning and had statements such as "the idea of taking *biology* makes me excited" and "I am confident I will do well on *biology* tests.
7. *Self-determination*: Students’ control and trust in learning science is a way of defining self-determination in students [119]. In 2009, a study used a science motivation questionnaire to test statements like “I put enough effort into learning the science” and “I prepare well for the science tests and labs,” which are similar to the statements used in our questionnaire. The study found that these assessments are reliable for the self-determination factor. Additionally, the study found that self-determination correlated with factors like college GPA, high school preparation, and career relevance [61]. Students’ placement in a different and more interactive learning environment where the students are freer to communicate, select readings, and be involved with the teachers is proved to increase students’ self-determination in learning biology [120–122].
8. *Grade Motivation*: This refers to students’ confidence that they can achieve passing grades, leading to positive behavior [93]. The questionnaire had statements like “Getting a good biology grade is important to me” and “It is important that I get an "A" in biology.” Based on previous research, it was found that when the grade motivation of the student is high, the student tends to give more attention to scientific subjects even when there is no interest in the subject itself [17, 18], which is why it is important to analyze students grade motivation in biology.
9. *Assessment Anxiety*: Assessment anxiety refers to negative feelings of learning biology, and it is helpful to understand students’ feelings to reduce negativity by using appropriate pedagogical approaches [93]. The survey had statements like “I am nervous about how I will do on the biology tests.” and “I worry about failing the biology tests." the students were assessed on how worried or nervous they are when being assessed in biology. Assessing student’s anxiety towards biology is essential as it is found to influence the students’ grades and confidence in a subject, thereby affecting the attitude towards it [123–125].

### Intervention and collaborative learning procedures

#### Semester-wide

**Fig 1:**
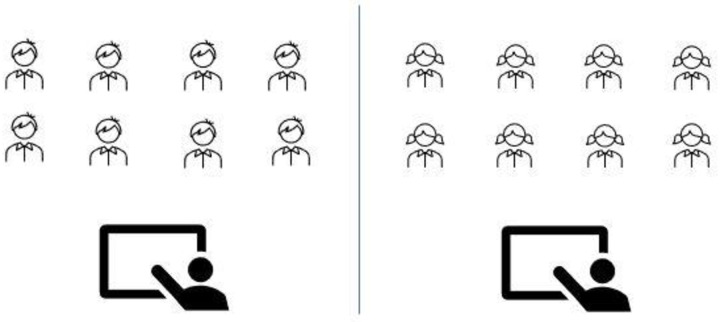
Single-gender traditional learning.

**Fig 2:**
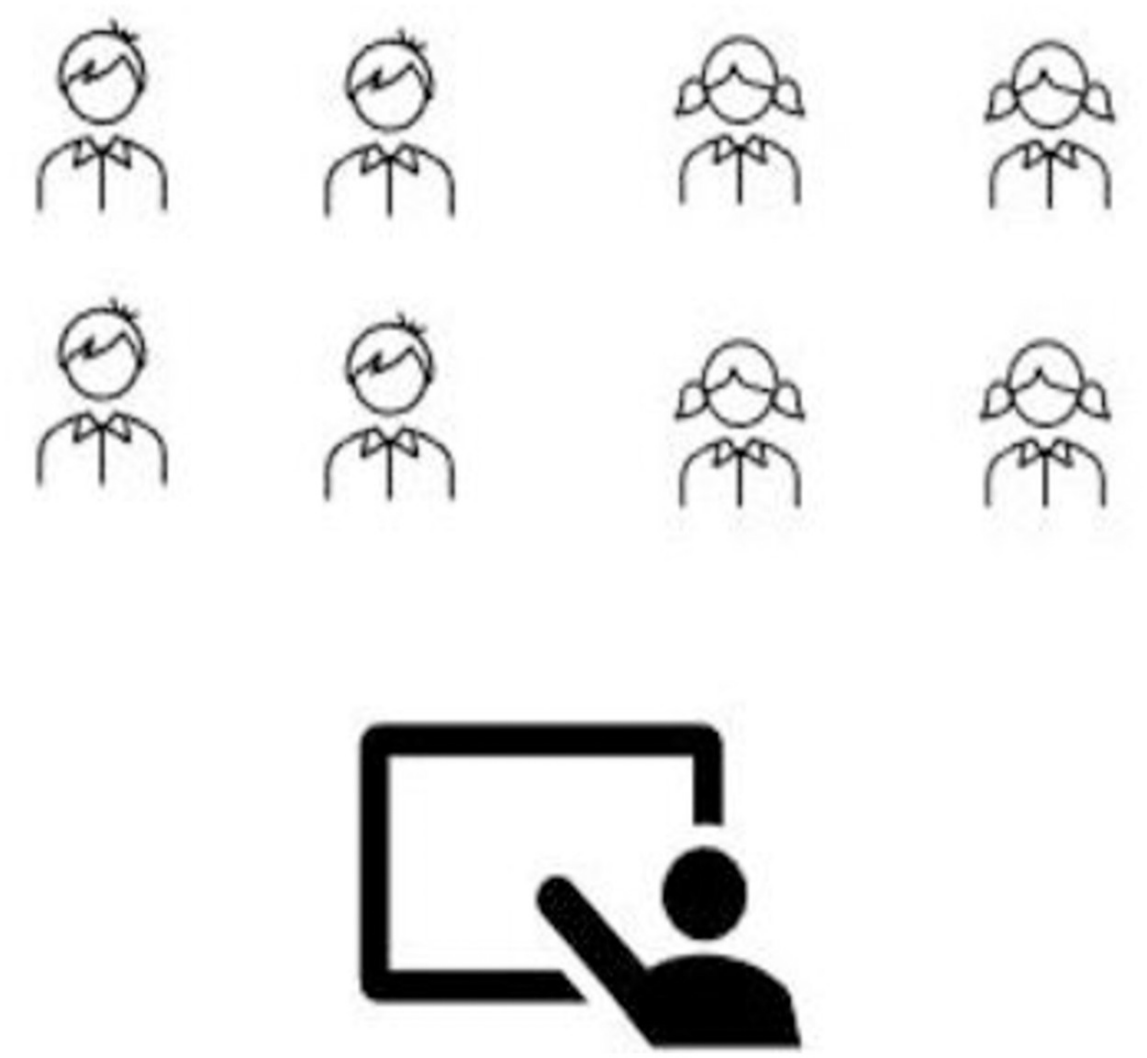
Mix-gender traditional learning men and women separated in the same classroom sitting on opposite sides.

**Fig 3:**
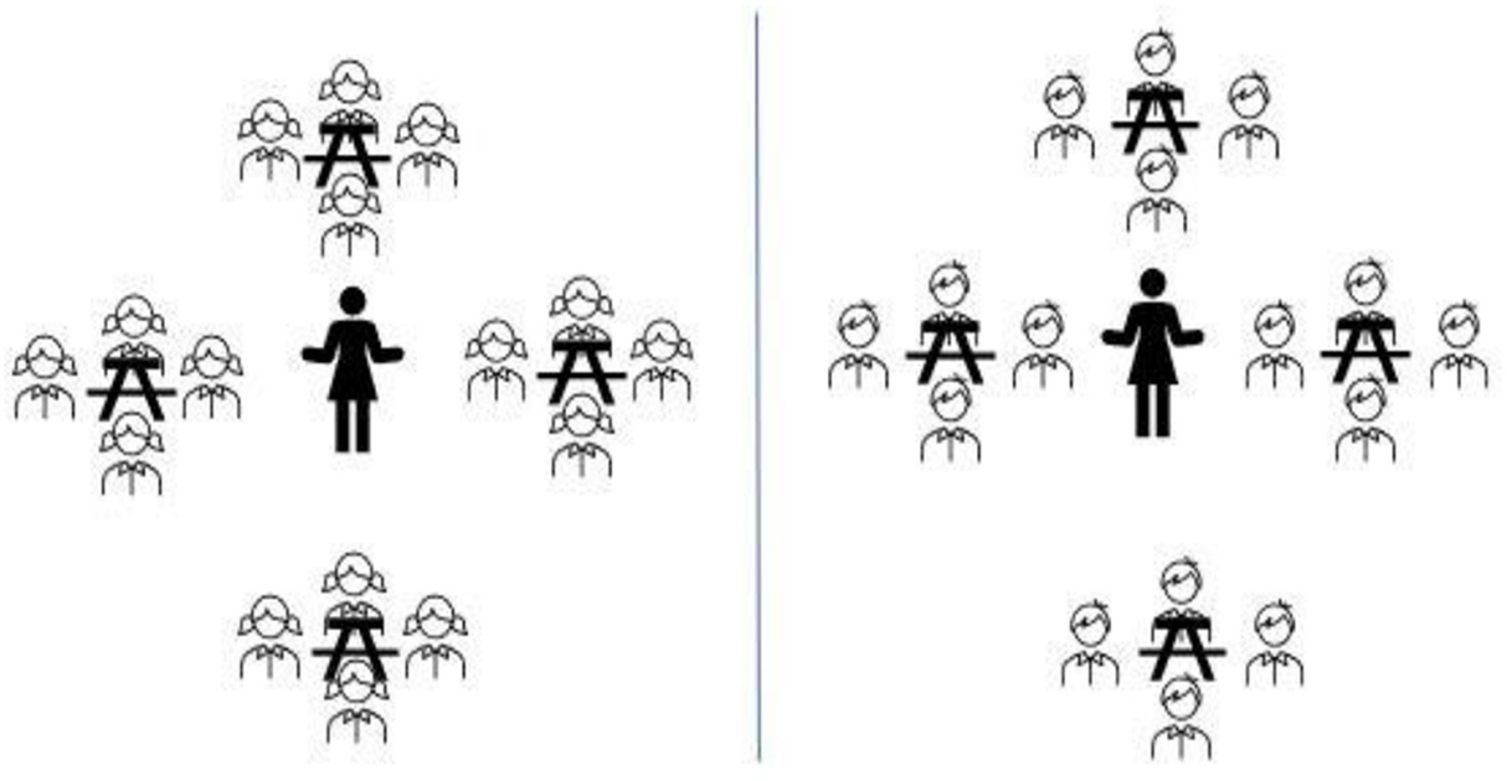
Single-gender collaborative learning (groups of (4W) or (4M)).

**Fig 4:**
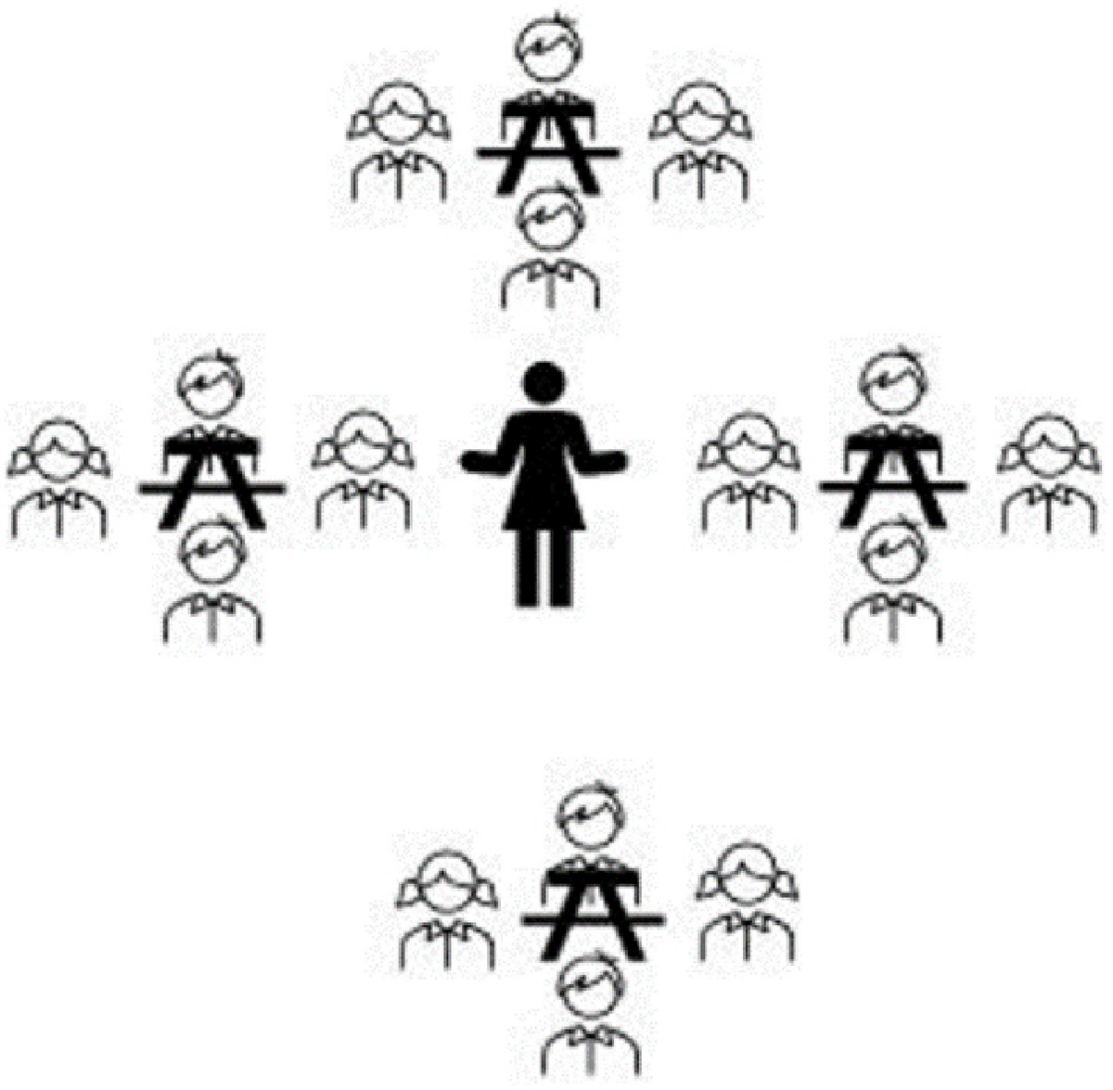
Mix-gender collaborative learning (groups of (2W+2M)).

To test our hypotheses, university science major and nonmajor students were in the introductory biology course (Table 1). The students had three lectures per week, each lecture an hour duration for a four-month semester. In traditional learning (TL) classes, students study content by posing and answering problems following the instructor’s direction, implementing TL in single-gender classes: Women and men were in separate classrooms (Fig 1). Implementing TL in mix-gender classes: Women and men sit separately on the opposite side in the same classroom (Fig 2). In collaborative learning (CL) classes, students work together to learn new concepts and solve problems; Implementing CL in single-gender classes, four women and four men were in a separate classroom working in groups of four (Fig 3); CL in mix-gender classes (2 women+2 men) working in groups of four in the same classroom (Fig 4). Both TL and CL students had the same assessment and examinations during the semester.

**Table 1:** Major and nonmajor biology student’s arrangements were implementing CL and TL methods.

Biology Students					
Gender Group	Teaching Method	Arrangement	Gender	Non-Major (N)	Major (N)
**Single Gender**	TL	Individual	Women (W)	30	30
			Men (M)	30	30
	CL	Groups of 4	Women (W)	31	31
			Men (M)	32	32
**Mixed Gender**	TL	Women and men are separated but are in the same class	Women (W)	35	32
			Men (M)	34	30
	CL	Groups of 4 (2 M & 2 W)	Women (W)	36	34
			Men (M)	36	33

In the collaborative learning sections, a random number generator was used to place the students in groups consisting of four members. Based on the social cohesion perspective, students placed in fixed groups during the semester, a more motivated, friendly, and effective teamwork environment is created among the students [26, 95]. Therefore, in our study, the students were placed with the same group during the entire semester. In terms of group compositions, Cen (2016) advised that having smaller groups as such composition is more effective in allowing a more substantial flow of ideas among the students than bigger groups [30]. The groups had a fixed plan scheduled for the lessons to be included in the class each week. Consequently, students prepare for the topic and divide lecture material between the group members before the class. Different parts were explained by each student to their group and were discussed to open a room for questions and answers [96]. After each discussion, the instructor provides the students with worksheet activities concerning the covered lesson, allowing them to discuss and solve the worksheet under the instructor’s supervision. These activities may comprise either a real-life scenario, fill in the blanks, or true or false type of questions. In total, three activities per lecture were handed out to students’ groups. Moreover, these activities take up almost 70% of the class time for around 45 minutes for each class. The instructor used the remaining time to summarize the points to the students. The intensity of the in-class activities becomes more challenging every 2–3 sessions. Such implementation in collaborative learning falls under the jigsaw method [97, 98] implementation. The focus is on the group discussion in an activity highly recommended when a subject to be taught consists of independent portions such as Biology. Moreover, collaborative learning is considered effective due to the instructor’s quality of guidance and feedback and the quality of the in-class activities [99]. Furthermore, the activities handed out to the students are in alignment with the examinations set by the instructor to some extent, where the questions in the upcoming exams are similar but not identical to the given activity [100, 101]. Handing out a difficult task or activity was found to lead to more effective collaborative learning as it leads to a higher level of creative thinking among the team members while solving the activity [102, 103].

### Statistical analysis

The quantitative data in this research were collected from the questionnaire and analyzed using descriptive statistics in the form of percentages, and means, as well inferential statistics such as Independent sample T-test were used to analyze the students’ responses [101, 102]. The data taken from the questionnaires that the students responded to were uploaded and analyzed using SPSS 27 or the Social Sciences Statistical Package. SPSS is widely used in research for data analysis, hence popular in academic circles [95]. In this paper, SPSS is used to compute the reliability continuously. To check the results consistency from the questionnaires, reliability is confirmed using Cronbach’s Alpha [96]. An acceptable range for a Cronbach’s Alpha should be 0.7 and above for reliability [96, 97]. Additionally, Independent Sample T-test was conducted to examine the significance of the observed differences between each factor for CL than TL for the same group of students. Independent sample t-test is important as it is used to check if there is a statistically significant difference in the mean scores for the two groups that are being studied or not [98]. The independent variables used in this study were two levels of gender (men, women) teaching method (TL, CL), gender group (single-gender, mix-gender), and biology students (major, nonmajor). One-way analysis of variance ANOVA was used to compare students’ attitudes due to learning style; an alpha level at *p*<.05 was used to test significance [67, 99, 100]. A factorial two-way ANOVA was conducted to compare the main effects of a student’s gender and the interaction effect between the student’s gender on attitude. The students’ attitude is the dependent variable, while gender and teaching methods are the two independent variables.

$$2\left(\text{Teaching}\;\text{method}:\text{TL}\;\text{vs}.\text{ CL}\right)*2\left(\text{Gender}:\text{Man}\;\text{vs}.\text{Woman}\right)$$

The three-way ANOVA is similar to the two-way ANOVA but instead has three independent variables on a continuous dependent variable [103]. In our study, the three independent variables were the teaching method (TL vs. CL), gender (Man vs. Woman), and the gender group (single vs. mixed), whereas the dependent variable was the students’ attitude.

$$2\;\text{Biology}\;\text{students}\left(\text{major},\text{nonmajor}\right)*2\left(\text{Teaching}\;\text{method}:\text{TL}\;\text{vs}.\text{CL}\right)*2\left(\text{Gender}:\text{Man}\;\text{vs}.\text{Woman}\right)$$

Cohen’s d effect size was used to compare two means. Cohen’s d effect size is used to be tied in with the reports of the t-test and ANOVA results. An effect size is used to infer the significance and extent of a result, and Cohen’s d specifically is used to express the standardized difference between two means of groups [104]. One major implication of Cohen’s d effect sizes is to show that even if a data value is found to be statistically significant if the d value was found to be below 0.2, the value is still trivial. Therefore, it has been standardized that when Cohen’s d value is discovered to be above 0.8, it has a large effect; the minimum accepted level of power is 0.8. When the value is between 0.2 and 0.5, it is of a medium effect, and when the value is below 0.2, it is of a small effect [101, 104, 105]. Finally, mean (average) calculations were performed to identify general trends in responses for each of the scales and items. Standard deviations were calculated to determine the degree of consistency among respondents for each scale. The complete statistical analysis is included in the S1 File.

## Results

The study examined dependent variable student’s attitudes toward biology versus independent variables students (major biology /nonmajor biology), students’ gender grouping woman (W)/ man (M), and the teaching methods applied TL and CL. The biology attitude questionnaire that measured student’s attitudes consisted of 62 items and nine subscales, *feeling towards biology*, *general interest*, *motivation towards learning biology item*, *benefit and utility of biology item*, *career motivation*, *self-efficacy in biology learning*, *self-determination*, *grade motivation*, *and assessment of anxiety* items. Four tables were used to present the results of this study (Table 2) Nonmajor biology single-gender (women and men in a separate classroom), (Table 3) Nonmajor biology mixed-gender (women and men in the same classrooms) students working alone applying the TL method or in groups (2M & 2W) applying CL method. (Table 4) Major biology single-gender students (women and men in separate classrooms). (Table 5) Major biology mixed-gender (women and men in the same classrooms) students working alone applying the TL method or in groups (2M & 2W) applying CL method. The tables present the individual students’ means and standard deviations attitude scores for each attitude factor towards biology in university students for major and nonmajor biology students with respect to gender and gender grouping. Calculating subscales’ internal consistency reliability, we computed Cronbach’s alpha coefficients (α) for each scale factor. Internal consistency reliability for each factor was higher than 0.7 for both women and men participating in both teaching methods. These findings indicate acceptable levels of internal consistency reliability for each factor. CL teaching method positively affects women’s attitude in single-gender nonmajor and negatively in mix-gender classes for nonmajor biology; however, the CL teaching method positively affects men’s attitude in single-gender nonmajor, and the effect was more significant in mix-gender classes for nonmajor biology.

**Table 2:** Nonmajor single-gender biology students, means and standard deviations attitude scores for each attitude factor.

**Attitude Factors Towards Biology**	**W (TL) N = 30**			**W (CL_4W) N = 31**			**TL *VS* CL P -Value Significance**	**TL *VS* CL Cohen’s d Effect Size**	**M (TL) N = 30**			**M (CL_4M) N = 32**			**TL *VS* CL P -Value Significance**	**TL *VS* CL Cohen’s d Effect Size**
	μ	SD	α	μ	SD	α			μ	SD	α	μ	SD	α		
Feeling toward biology	3.06	0.50	0.809	3.78	0.43	0.851	P<.001*	d = 1.5440	2.55	0.50	0.885	3.11	0.45	0.850	P<.001*	d = 1.1773
General interest	3.69	0.82	0.884	4.27	0.56	0.859	P = 0.002*	d = 0.8260	2.46	0.75	0.831	3.46	0.64	0.725	P<.001*	d = 1.4344
Motivation towards learning biology	3.75	0.56	0.850	4.17	0.49	0.798	P = 0.003*	d = 0.8073	2.41	0.65	0.772	3.34	0.50	0.860	P<.001*	d = 1.6038
Benefit and Utility of biology	3.66	0.58	0.818	4.16	0.89	0.881	P = 0.012*	d = 0.6656	2.98	0.58	0.889	3.42	0.62	0.818	P = 0.013*	d = 0.7329
Career Motivation	3.51	0.64	0.805	3.91	0.86	0.879	P = 0.046*	d = 0.5277	2.48	0.57	0.804	2.87	0.69	0.813	P = 0.027*	d = 0.6163
Self-Efficacy in biology learning	3.84	0.52	0.789	4.02	0.57	0.827	P = 0.213	d = 0.3299	2.25	0.58	0.761	3.59	0.34	0.858	P<.001*	d = 2.8187
Self-Determination	3.45	0.70	0.771	3.79	0.44	0.775	P = 0.024*	d = 0.5816	2.73	0.68	0.755	3.26	0.55	0.719	P = 0.001*	d = 0.8570
Grade Motivation	3.85	0.57	0.753	4.01	0.69	0.889	P = 0.337	d = 0.2485	2.57	0.61	0.770	3.10	0.41	0.819	P<.001*	d = 1.0198
Assessment anxiety^Ŕ^	3.50	0.71	0.809	4.27	0.58	0.729	P<.001*	d = 1.1877	2.42	0.45	0.778	3.42	0.52	0.778	P<.001*	d = 2.0565
**Attitude Factors Mean**^Á^	**μ**	**SD**	**α**	**μ**	**SD**	**α**	P<.001*	**d = 0.6240**	**μ**	**SD**	**α**	**μ**	**SD**	**α**	P<.001*	**d = 1.2460**
	**3.59**	**0.66**	**0.810**	**4.04**	**0.65**	**0.832**			**2.54**	**0.60**	**0.805**	**3.28**	**0.59**	**0.804**		

^Á^ Using a five-point Likert scale ranging from 1 (Strongly disagree) to 5 (Strongly agree).

^Ŕ^ Items are reverse-scored when added to the total, so a higher score on this component means less anxiety.

**Table 3:** Nonmajor mix-gender biology students, means and standard deviations attitude scores for each attitude factor.

**Attitude Factors Towards Biology**	**W (TL) N = 35**			**W (CL_2W+2M) N = 36**			**TL *VS* CL P -Value Significance**	**TL *VS* CL Cohen’s d Effect Size**	**M (TL) N = 34**			**M (CL_2M+2W) N = 36**			**TL *VS* CL P -Value Significance**	**TL *VS* CL Cohen’s d Effect Size**
	μ	SD	α	μ	SD	α			μ	SD	α	μ	SD	α		
Feeling toward biology	3.01	0.58	0.863	2.56	0.51	0.873	P = .001*	d = 1.1173	2.90	0.49	0.894	3.91	0.38	0.868	P<.001*	d = 2.3842
General interest	3.43	0.62	0.815	3.3	0.66	0.776	P = 0.415	d = 0.2030	2.79	0.42	0.804	3.90	0.51	0.874	P<.001*	d = 2.3759
Motivation towards learning biology	3.22	0.38	0.775	3.1	0.42	0.817	P = 0.219	d = 0.2994	3.08	0.41	0.880	3.77	0.51	0.815	P<.001*	d = 1.4391
Benefit and Utility of biology	3.31	0.58	0.820	2.9	0.57	0.868	P = 0.004*	d = 0.7098	2.29	0.88	0.780	3.65	0.95	0.871	P<.001*	d = 1.4852
Career Motivation	2.91	0.54	0.810	2.51	0.60	0.826	P = 0.004*	d = 0.7005	2.39	0.92	0.849	3.91	0.87	0.864	P<.001*	d = 1.6967
Self-Efficacy in biology learning	3.13	0.45	0.782	2.2	0.43	0.748	P<.001*	d = 2.1062	2.40	0.43	0.858	3.45	0.55	0.774	P<.001*	d = 2.1263
Self-Determination	2.80	0.39	0.800	2.43	0.40	0.771	P<.001*	d = 1.3849	2.64	0.44	0.807	3.55	0.55	0.777	P<.001*	d = 1.7293
Grade Motivation	3.12	0.48	0.776	2.26	0.53	0.790	P<.001*	d = 1.6999	2.80	0.40	0.848	3.91	0.62	0.866	P<.001*	d = 2.1225
Assessment anxiety^Ŕ^	2.71	0.44	0.772	2.1	0.40	0.787	P<.001*	d = 1.9344	2.91	0.61	0.792	3.66	0.52	0.793	P<.001*	d = 1.3007
**Attitude Factors Mean**^Á^	**μ**	**SD**	**α**	**μ**	**SD**	**α**	P<.001*	**d = 0.7091**	**μ**	**SD**	**α**	**μ**	**SD**	**α**	P<.001*	**d = 1.8417**
	**3.06**	**0.55**	**0.801**	**2.59**	**0.65**	**0.806**			**2.69**	**0.60**	**0.835**	**3.75**	**0.55**	**0.834**		

^Á^ Using a five-point Likert scale ranging from 1 (Strongly disagree) to 5 (Strongly agree).

^Ŕ^ Items are reverse-scored when added to the total, so a higher score on this component means less anxiety.

**Table 4:** Major single-gender biology students’ attitude means, standard deviations, and reliability of attitude scale components for each attitude factor.

**Attitude Factors Towards Biology**	**F (TL) N = 30**			**F (CL_4F) N = 31**			**TL *VS* CL P -Value Significance**	**TL *VS* CL Cohen’s d Effect Size**	**M (TL) N = 30**			**M (CL_4M) N = 32**			**TL *VS* CL P -Value Significance**	**TL *VS* CL Cohen’s d Effect Size**
	μ	SD	α	μ	SD	α			μ	SD	α	μ	SD	α		
Feeling toward biology	4.01	0.47	0.883	4.83	0.46	0.896	P<.001*	d = 1.7633	3.38	0.77	0.898	4.13	0.54	0.797	P<.001*	d = 1.1277
General interest	4.10	0.62	0.840	4.55	0.61	0.902	P = 0.006*	d = 0.7317	3.92	0.67	0.830	4.31	0.62	0.863	P = 0.021*	d = 0.6042
Motivation towards learning biology	4.42	0.49	0.881	4.76	0.53	0.867	P = 0.011*	d = 0.6662	4.11	0.65	0.885	4.63	0.66	0.848	P = 0.003*	d = 0.7939
Benefit and Utility of biology	3.92	0.59	0.824	4.40	0.54	0.962	P = 0.001*	d = 0.8487	4.07	0.64	0.823	4.37	0.49	0.845	P = 0.049*	d = 0.5264
Career Motivation	4.28	0.37	0.845	4.82	0.41	0.796	P<.001*	d = 1.3828	3.92	0.52	0.874	4.87	0.57	0.898	P<.001*	d = 1.7413
Self-Efficacy in biology learning	3.98	0.72	0.848	4.80	0.49	0.889	P<.001*	d = 1.3315	3.88	0.66	0.837	4.44	0.62	0.852	P = 0.002*	d = 0.8746
Self-Determination	4.12	0.70	0.757	4.69	0.57	0.817	P = 0.001*	d = 0.8929	3.58	0.76	0.716	4.54	0.58	0.828	P<.001*	d = 1.4002
Grade Motivation	4.09	0.65	0.721	4.71	0.47	0.747	P<.001*	d = 1.0931	3.77	0.63	0.834	4.86	0.59	0.792	P<.001*	d = 1.7859
Assessment anxiety^Ŕ^	3.80	0.55	0.809	4.67	0.57	0.805	P<.001*	d = 1.5533	3.66	0.59	0.801	4.34	0.53	0.870	P = 0.002*	d = 1.2125
**Attitude Factors Mean**^Á^	**μ**	**SD**	**α**	**μ**	**SD**	**α**	P<.001*	**d = 1.0471**	**μ**	**SD**	**α**	**μ**	**SD**	**α**	P<.001*	**d = 1.1892**
	**4.08**	**0.64**	**0.823**	**4.70**	**0.54**	**0.853**			**3.80**	**0.65**	**0.833**	**4.50**	**0.52**	**0.844**		

^Á^ Using a five-point Likert scale ranging from 1 (Strongly disagree) to 5 (Strongly agree).

^Ŕ^ Items are reverse-scored when added to the total, so a higher score on this component means less anxiety.

**Table 5:** Major mix-gender biology students’ attitude means, standard deviations, and reliability of attitude scale components for each attitude factor.

**Attitude Factors Towards Biology**	**F (TL) N = 28**			**F (CL_2F+2M) N = 32**			**TL *VS* CL P -Value Significance**	**TL *VS* CL Cohen’s d Effect Size**	**M (TL) N = 29**			**M (CL_2M+2F) N = 32**			**TL *VS* CL P -Value Significance**	**TL *VS* CL Cohen’s d Effect Size**
	**μ**	**SD**	**α**	**μ**	**SD**	**α**			**μ**	**SD**	**α**	**μ**	**SD**	**α**		
Feeling toward biology	4.31	0.51	0.850	4.17	0.66	0.899	P = 0.381	d = 0.2374	4.54	0.41	0.851	4.62	0.39	0.868	P = 0.434	d = 0.1999
General interest	4.00	0.66	0.895	3.78	0.63	0.755	P = 0.158	d = 0.3409	4.20	0.74	0.889	4.55	0.61	0.765	P = 0.049*	d = 0.5161
Motivation towards learning biology	4.34	0.51	0.872	3.93	0.69	0.845	P = 0.015*	d = 0.6758	4.10	0.52	0.875	4.63	0.58	0.877	P = 0.001*	d = 0.9622
Benefit and Utility of biology	3.90	0.59	0.814	3.79	0.60	0.812	P = 0.467	d = 0.1849	4.05	0.65	0.889	4.75	0.64	0.796	P<.001*	d = 1.0852
Career Motivation	4.07	0.49	0.779	3.53	0.71	0.792	P = 0.001*	d = 0.8852	3.89	0.42	0.728	4.88	0.63	0.803	P<.001*	d = 1.8491
Self-Efficacy in biology learning	3.70	0.65	0.804	3.40	0.82	0.826	P = 0.113	d = 0.4055	3.88	0.74	0.835	4.55	0.59	0.710	P<.001*	d = 1.0012
Self-Determination	4.21	0.81	0.830	3.08	0.89	0.713	P<.001*	d = 1.3279	3.85	0.59	0.732	4.36	0.75	0.728	P = 0.005*	d = 0.7558
Grade Motivation	4.73	0.49	0.747	3.85	0.63	0.809	P<.001*	d = 1.5593	3.77	0.53	0.749	4.85	0.63	0.792	P<.001*	d = 1.8552
Assessment anxiety^Ŕ^	3.92	0.56	0.749	3.01	0.78	0.717	P<.001*	d = 1.5962	3.66	0.71	0.738	4.79	0.67	0.743	P<.001*	d = 1.6369
**Attitude Factors Mean**^Á^	**μ**	**SD**	**α**	**μ**	**SD**	**α**	P<.001*	**d = 0.6997**	**μ**	**SD**	**α**	**μ**	**SD**	**α**	P<.001*	**d = 1.2460**
	**4.13**	**0.65**	**0.816**	**3.62**	**0.80**	**0.796**			**3.99**	**0.65**	**0.810**	**4.66**	**0.63**	**0.787**		

^Á^ Using a five-point Likert scale ranging from 1 (Strongly disagree) to 5 (Strongly agree).

^Ŕ^ Items is reverse-scored when added to the total, so a higher score on this component means less anxiety.

### Nonmajor biology students

Hypothesis 1 and Hypothesis 2 were tested and there was a statistically significant three-way interaction between gender, teaching method, and gender composition 4W,2W+2M,4M; (F (1, 2367) = 139.848, p<0.001 η^2^ = 0.056, R^2^ = 0.451). Supporting our hypothesis; Results indicated that nonmajor biology men have significantly higher positive attitude toward biology in CL classes compared to TL classes in both mix-gender (M_CL μ = 3.75 SD = 0.55 versus M_TL μ = 2.69 SD = 0.6) p<0.001 effect size Cohen’s d = 1.8417 and single-gender classes (M_CL μ = 3.28 SD = 0.59 versus M_TL μ = 2.54 SD = 0.60) p<0.001 effect size Cohen’s d = 1.2460. Men showed significantly higher positive attitude in CL mixed-gender groups (2M+2W), (F (1, 1265) = 502.49, p <0.001 partial η2 = 0.284) compared to men in CL single-gender groups effects (F (1, 1102) = 199.724, P < 0.001 partial η^2^ = 0.153) with an effect size Cohen’s d = 0.2769.

On the other hand, women developed higher level of positive attitude toward biology in single-gender classes CL (4W) compared to TL (W_CL μ = 4.04 SD = 0.65 versus W_TL μ = 3.59 SD = 0.66) p<0.01, d = 0.6240, and a negative attitude toward biology in CL (2M+2W) compared to TL mix-gender classes (W_CL μ = 2.59 SD = 0.65 versus W_TL μ = 3.06 SD = 0.55) p<0.01 Cohen’s d = 0.7091. Women in single-gender have significantly higher positive attitude toward biology in CL (4W) group compared to TL group (F (1,1102) = 57.975, p<0.001., η^2^ = 0.05); however, in mix-gender class, women students developed a higher positive attitude in TL compared to CL (F (1,265) = 97.27, p<.001, η^2^ = 0.071). Results indicate that gender and gender composition’s interaction depend on the teaching method for nonmajor biology students. Men developed a positive attitude applying CL compared to TL in single-gender and mix-gender classes. The CL effect was higher in mixed-gender compare to single-gender. Women developed positive attitudes from CL compared to TL in single-gender classes as their attitude dropped in CL mixed-gender classes, and it was higher in TL single-gender classes.

### Major biology students

Hypothesis 3 and Hypothesis 4 were tested, and there was a statistically significant three-way interaction between gender, teaching method, and gender composition (F (1, 2189) = 94.208, p<0.001 η^2^ = 0.041, R^2^ = 0.448). Supporting our hypothesis; results indicated that major biology men have higher positive attitude toward biology in CL classes compared to TL classes in both mix-gender (M_CL μ = 4.66 SD = 0.63 versus M_TL μ = 3.99 SD = 0.65) p<0.001 and for the single-gender classes (M_CL μ = 4.50 SD = 0.52 versus M_TL μ = 3.80 SD = 0.65) p<0.001 Cohen’s effect size d = 1.2460. Men have high positive attitudes in CL mixed-gender groups (2M+2W) (F (1,1087) = 129.409, p<0.001., η^2^ = 0.106) compared to men in CL single-gender groups (F (1,1102) = 162.188, p<0.001, η^2^ = 0.128); the Cohen’s effect size d = 0.2769. On the other hand, women have higher positive attitude toward biology in CL (4W) compared to TL single-gender classes (W_CL μ = 4.70 SD = 0.54 versus W_TL μ = 4.08 SD = 0.64) p<0.001, d = 1.0471, and negative attitudes toward biology in CL (2M+2F) compared to TL mix-gender classes (W_CL μ = 3.62 SD = 0.80 versus W_TL μ = 4.13 SD = 0.65) p<0.01 Cohen’s d = 0.6997. Women in single-gender have significantly higher positive attitude towards biology in CL (4W) group compared to TL group (F (1,1102) = 126.025, p<0.001, η^2^ = 0.103; however, in mix-gender women have a higher positive attitude in TL compared to CL (F (1,1087) = 74.810, p<0.001, η^2^ = 0.064). Results indicate that gender and gender composition’s interaction depend on the teaching method for major biology classes. Men developed a positive attitude applying CL compared to TL in single-gender, and the attitude was higher, applying CL compared to TL in mix-gender. Women performed better in CL single classes than TL and developed a negative attitude toward biology in CL in mix-gender classes compared to TL.

### Nonmajor single-gender men and women attitudes

**Fig 5:**
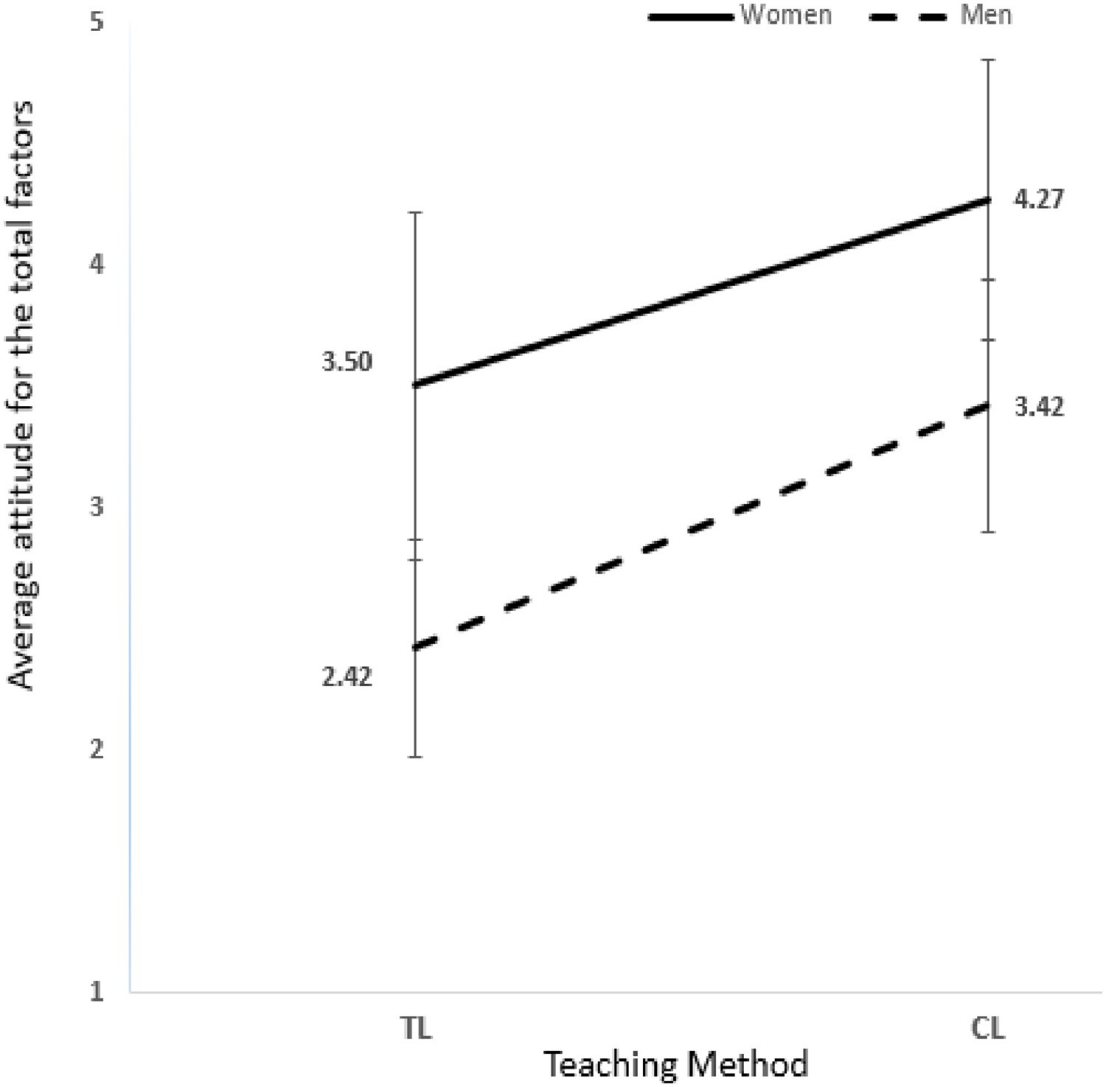
Nonmajor single-gender: Present the total nine attitude factors mean and SD for nonmajor single-gender students.

Table 2 shows nonmajor biology single-gender students, means and standard deviations attitude scores for the nine attitude items factor. A two-way ANOVA was conducted to compare the main effect of gender and teaching method and their interaction effect on student attitude toward biology. Results revealed that the interaction effect between (gender * teaching method) have a significant interaction effect (F (1, 1102) = 20.632, p< 0.05, η^2^ = 0.18) in the single-gender nonmajor biology indicates that there was a combined effect for gender. This interaction explained the teaching method on the student’s attitude towards biology and 18% of the variance in the students’ attitude. The main effect for student’s gender yielded an ***F*** ratio of (F (1, 1102) = 699.428, p<0.05 partial η^2^ = 0. 388) was significant, indicating that 38.8% of the variance in the students’ attitude was explained by gender; the effects showed that women were significantly having higher positive attitude score in general interest toward biology compared to men in both traditional method W(TL) VS M(TL) P<.01, d = 1.6586 (W(TL) μ = 3.59 SD = 0.66 VS M(TL) μ = 2.54 SD = 0.60), and collaborative method W(CL) VS M(CL) P<.01, d = 1.2193, W(CL) μ = 4.04 SD = 0.65 VS W(CL) μ = 3.28 SD = 0.59). The teaching method’s main effect yielded an ***F*** ratio of (F (1, 1102) = 91.443, p<0.05, η^2^ = 0.176), indicating the effect for the teaching method used was significant 17.6% of the variance in the students’ attitude was explained by teaching method. CL method has a higher impact on women and men attitude toward biology compared to TL method p<0.01, d = 0.624 W(CL) μ = 4.04 SD = 0.65 VS W(TL) μ = 3.59 SD = 0.66); however, this impact was higher on men and developed a significantly higher positive attitude in biology in CL compared to TL with a p<.01, d = 1.246 M(CL) μ = 3.28 SD = 0.59 VS M(TL) μ = 2.54 SD = 0.60) as (Fig 5) shows. The results indicate that the significant differences in attitude scores between students taught with CL & TL teaching methods are linked and depend on students’ gender, so combining the gender and teaching methods used in classes affects nonmajor biology students’ attitude towards biology.

The most influential attitudes of women in CL classes that were significantly different compared to TL method were feeling toward biology W(TL) μ = 3.06 SD = 0.50 VS W(CL) μ = 3.78 SD = 0.43 p<0.01, d = 1.544; while the least insignificant were grade motivation W(TL) μ = 3.85 SD = 0.57 VS W(CL) μ = 4.01 SD = 0.69 p = 0.337, d = 0.2485. The most influential attitude factor for men applying CL method were self-efficiency in learning biology M(TL) μ = 2.25 SD = 0.58 VS M(CL) μ = 3.59 SD = 0.34 p<0.01, d = 2.8187; while the least and insignificant differences were in benefit and utility of biology M(TL) μ = 2.98 SD = 0.58 VS M(CL) μ = 3.42 SD = 0.62 p = 0.013, d = 0.7329.

### Nonmajor mix-gender men and women attitudes

**Fig 6:**
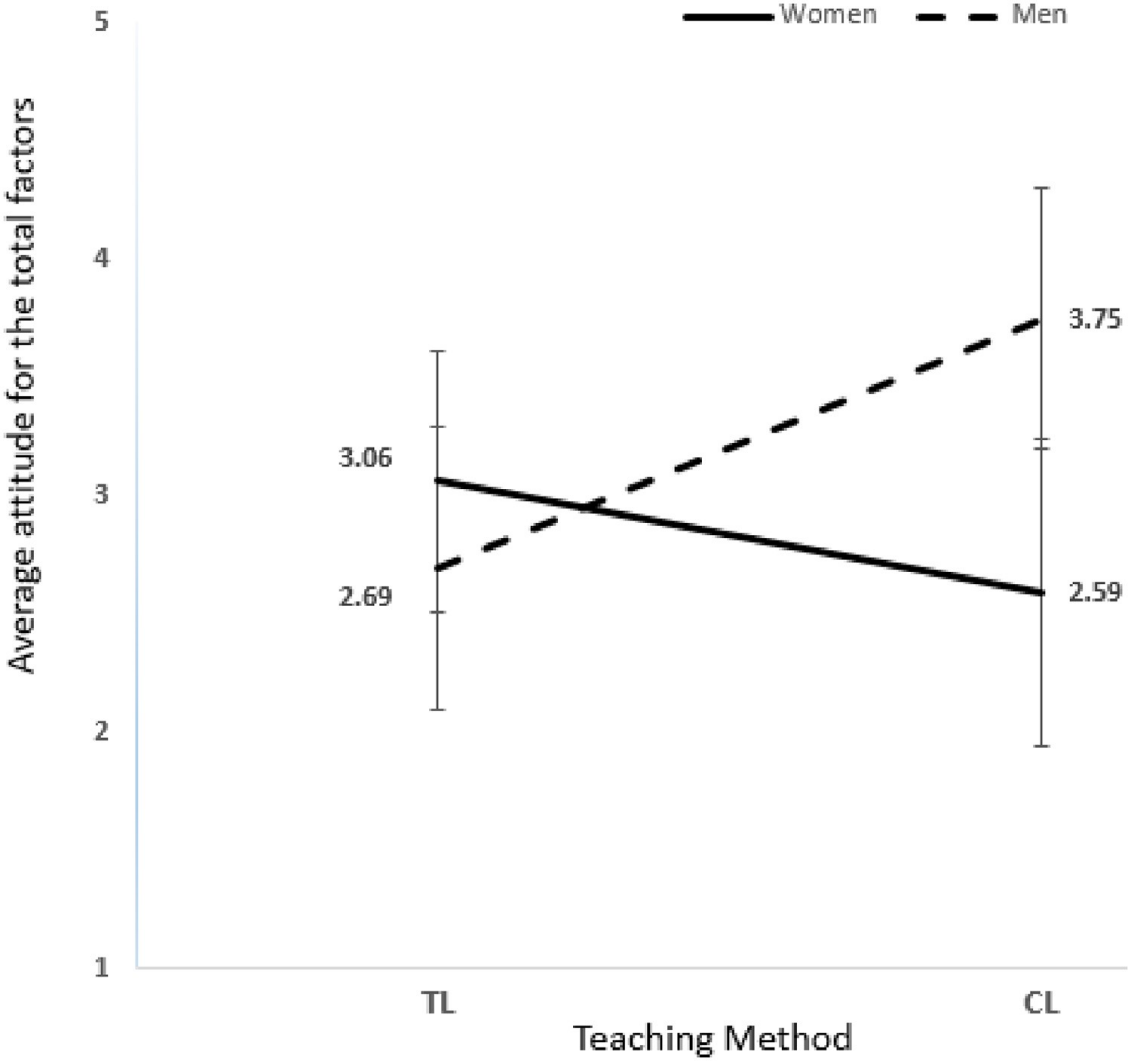
Nonmajor mix-gender: Present the total nine attitude factors mean and SD for nonmajor mix-gender students.

Table 3 shows nonmajor mix-gender biology students, means, and standard deviations attitude scores for the nine attitude items. A two-way ANOVA was conducted to compare the main effect of gender and teaching method and their interaction effect on student attitude toward biology. Results revealed that the interaction effect between (gender * teaching method) in mix-gender nonmajor have a significant interaction effect (F (1, 1265) = 552.49, p<0.05,η^2^ = 0.292) indicates that there was a combined effect for gender and teaching method on the student’s attitude toward biology, this interaction effect is much more important than either of the individual main effects for gender, and teaching method since 29.2% of the variance in the students’ attitude was explained by this interaction and almost as twice stronger than this interaction in single-gender nonmajor classes (F (1,1102) = 20.632, p<0.05,η^2^ = 0.18) were 18% of the variance in the students’ attitude was explained by this interaction. The main effect for student’s gender yielded an ***F*** ratio of (F (1, 1265) = 138.199, p<0.05, η^2^ = 0. 098) was significant, indicating that 9.8% of the students’ attitude was explained by gender; showed that women were significantly having higher attitude score in general toward biology compared to men in traditional method W(TL) VS M(TL) p<0.01, d = 0.6408 (W(TL) μ = 3.06 SD = 0.55 VS M(TL) μ = 2.69 SD = 0.60), and men were significantly having higher positive attitude score in collaborative method compared to women W(CL) VS M(CL) p<0.01, d = 1.9266, W(CL) μ = 2.59 SD = 0.65 VS M(CL) μ = 3.75 SD = 0.55. The teaching method’s main effect yielded an ***F*** ratio of (F (1, 1265) = 80.312, p<0.05, η^2^ = 0.060), indicating 6% of the students’ attitude was explained by the teaching method. The effect of the teaching method used was significant. CL method have a positive impact on men mix-gender section attitude M(CL) μ = 3.75 SD = 0.55 VS M(TL) μ = 2.69 SD = 0.60 p<0.01, d = 1.8417 and a high negative impact on women’s attitude in the mix-gender section, W(CL) μ = 2.59 SD = 0.65 VS W(TL) μ = 3.06 SD = 0.55 p<0.01, d = 0.7091 as (Fig 6) shows. The results indicate that the significant differences in attitude scores between students taught with CL & TL methods are linked and depend on students’ gender, so combining the gender and teaching methods used in classes affects nonmajor biology students’ attitude towards biology.

CL teaching method negatively affects women’s attitudes in nonmajor mix-gender classes for nonmajor biology students in all attitude factors. Most attitude factors have been affected negatively applying CL compared to TL, such as self-efficacy in biology learning W(TL) μ = 3.13 SD = 0.45 VS W(CL) μ = 2.2 SD = 0.43 p<.01, d = 2.1062. In contrast, the CL teaching method positively affects men’s attitude in mix-gender classes for nonmajor biology students. The most attitude factors being affected positively applying CL in mix-gender nonmajor classes were feeling toward biology M(TL) μ = 2.90 SD = 0.49 VS M(CL) μ = 3.91 SD = 0.38 p<0.01, d = 2.3842.

### Major single-gender men and women attitudes

Table 4 shows major single-gender biology students’ attitude means and standard deviations attitude scores for the nine attitude items. A two-way ANOVA was conducted to compare the main effect of gender and teaching method and their interaction effect on student attitude toward biology. Results revealed that the interaction effect between (gender * teaching method) was not significant (F (1, 1102) = 0.988, p = 0.320, η^2^ = 0.001) in the single-gender major biology indicates that there was no combined effect for gender and teaching method on the student’s attitude toward biology, the effect of teaching method on student’s attitude does not differ by gender. The main effect for students’ gender yielded an ***F*** ratio of (F (1, 1102) = 38.865, p< 0.05, η^2^ = .034) was significant, indicating that 3.4% of the variance in the students’ attitude was explained by gender; the effects showed that women were significantly having higher attitude score in general toward biology compared to men in both traditional method W(TL) VS M(TL) P<.01, d = 0.4341 (W(TL) μ = 4.08 SD = 0.64 VS M(TL) μ = 3.8 SD = 0.65), and collaborative method W(CL) VS M(CL) P<.01, d = 0.3772, W(CL) μ = 4.7 SD = 0.54 VS M(CL) μ = 4.5 SD = 0.52).

The teaching method’s main effect yielded an ***F*** ratio of (F (1, 1102) = 286.912, p< 0.05, η^2^ = .207), indicating the effect for the teaching method used was significant and that 20.7% of the variance in the students’ attitude was explained by teaching method. The relative impact of the teaching method is more than six times as strong as students’ gender. Adjusted R^2^ tells us that 22.8% of students’ attitude variance is attributable to the teaching method and gender.

**Fig 7:**
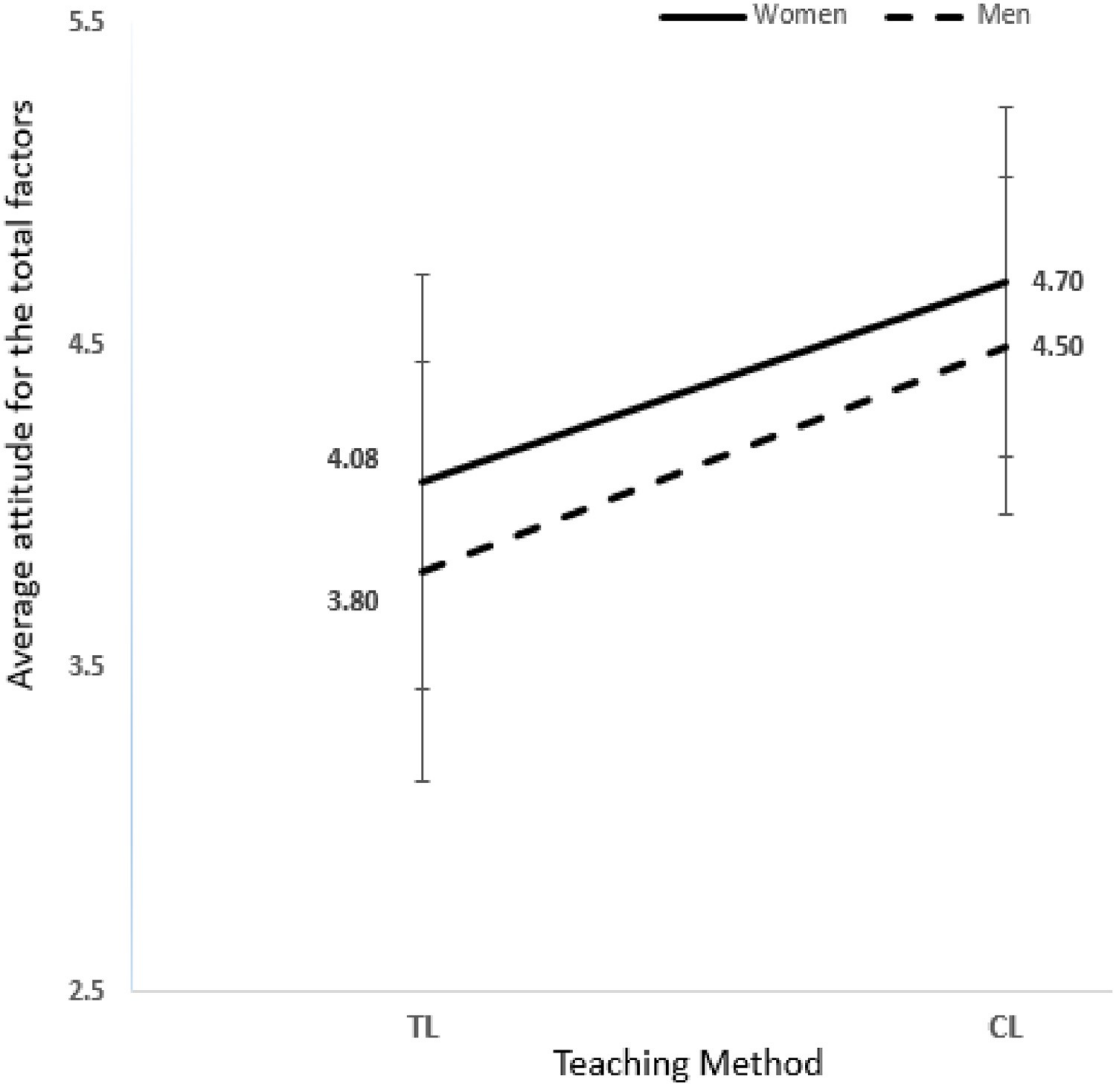
Major single-gender: Present the total nine attitude factors mean and SD for major single-gender students.

CL method has a higher impact on women and men attitude toward biology compared to TL method p<.01, d = 1.0471 W(CL) μ = 4.70 SD = 0.54 VS W(TL) μ = 4.08SD = 0.64); However, this impact was higher on men and benefited significantly more from CL compared to TL with a p<.01, d = 1.1892 M(CL) μ = 4.50 SD = 0.62 VS M(TL) μ = 3.89 SD = 0.60 as (Fig 7) shows. The results indicate that the teaching method’s effect on the attitude level does not matter whether the student is a woman or man since the teaching method affects the students from both genders significantly. The insignificance in an interaction could be attributed to the fact that the students in this category are majoring in biology; hence the students have a strong attitude towards the subject irrespective of the gender of the student.

The most influential attitude for women in major single-gender section applying CL method that was significantly different compared to TL method was feeling toward biology W(TL) μ = 4.01 SD = 0.47 VS W(CL) μ = 4.83 SD = 0.46 p<.01, d = 1.7633. While the least insignificant was general interest W(TL) μ = 4.10 SD = 0.62 VS W(CL) μ = 4.55 SD = 0.61 P = 0.006, d = 0.7317. The most influential attitude factor for men in major single-gender section applying CL method was grade motivation M(TL) μ = 3.77 SD = 0.63 VS M(CL) μ = 4.86 SD = 0.59 p<.01, d = 1.7859, while the least and insignificant differences were in benefit and utility of biology M(TL) μ = 4.07 SD = 0.64 VS M(CL) μ = 4.37 SD = 0.49 P = 0.049, d = 0.5264.

### Major mix-gender men and women attitudes

**Fig 8:**
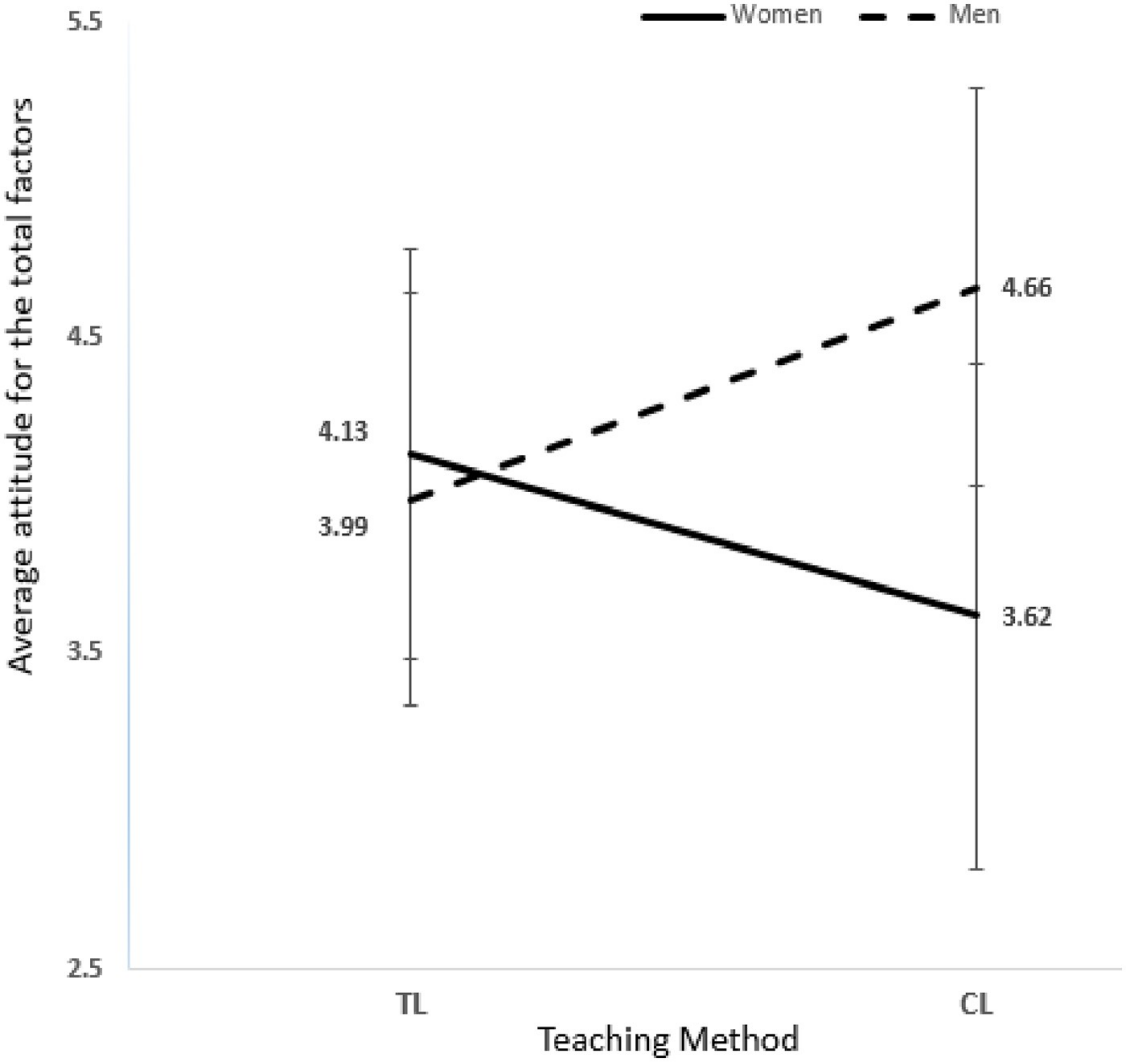
Major mix-gender: Present the total nine attitude factors mean and SD for major mix-gender students.

Table 5 shows major mix-gender biology students’ attitude means and standard deviations attitude scores for the nine attitude items factor. A two-way ANOVA was conducted to examine gender and teaching methods on students’ attitudes, using gender and teaching methods as the two fixed independent factors and attitudes as the dependent variable. Results revealed that the interaction effect between (gender * teaching method) was significant (F (1, 1087) = 200.245, p <0.05 partial η^2^ = 0.156) indicates that there was a combined effect for gender and teaching method on the student’s attitude toward biology, this interaction effect is much more important than either of the individual main effects for gender and teaching method since 15.6% of the variance in the students’ attitude was explained by this interaction and much stronger than the single major classes hence there was no significant interaction observed (F (1, 1102) = 0.988, p = 0.320, η^2^ = 0.001). The main effect for student’s gender yielded an ***F*** ratio of (F (1, 1087) = 118.487, p<0.05, η^2^ = 0.098) was significant indicating that 9.8% of the student’s attitude was explained by gender; showed that women were significantly having higher positive attitude score in general interest toward biology compared to men in TL method W(TL) VS M(TL) P<.01, d = 0.2170 (W(TL) μ = 4.13 SD = 0.65 VS M(TL) μ = 3.99 SD = 0.64), and men were significantly having higher positive attitude score in CL method compared to women W(CL) VS M(CL) P<.01, d = 1.4443, W(CL) μ = 3.62 SD = 0.80 VS M(CL) μ = 4.66 SD = 0.63). The teaching method’s main effect was not significant yielded an ***F*** ratio of (F (1, 1087) = 3.467, p = 0.063 a week effect η^2^ = 0.003). An assumption behind this insignificance could be that students majoring in biology are already interested in biology; hence, teaching methods are not as influential as nonmajor students, where students need exposure to different methods to gain interest. CL method has a positive impact on men mix-gender section attitude M(CL) μ = 4.66 SD = 0.63 VS M(TL) μ = 3.99 SD = 0.65 p<0.01, d = 1.2460 and a high negative impact on women attitude in the mix-gender section, W(CL) μ = 3.62 SD = 0.80 VS W(TL) μ = 4.13 SD = 0.65 p<0.01, d = 0.6997 as (Fig 8) shows. The results indicate that the significant difference in attitude scores between students taught with CL & TL teaching methods is linked and depends on the student’s gender, so combining the gender and teaching methods used in classes affects major biology students’ attitude towards biology.

CL method affects women’s attitude negatively in mix-gender major biology classes. The attitude factors that have been affected negatively applying CL compared to TL are assessment anxiety in biology learning W(TL) μ = 3.92 SD = 0.56 VS W(CL) μ = 3.01 SD = 0.58 p<.01, d = 1.5962. In contrast, the CL teaching method positively affects men’s attitude in mix-gender classes for major biology students. The most attitude factors that affected positively applying CL in mix-gender major classes were grade motivation M(TL) μ = 3.77 SD = 0.53 VS M(CL) μ = 4.85 SD = 0.63 p<0.01, d = 1.8552. In summary, we found statistically significant three-way interaction between gender, teaching method, and gender composition, and there was an interactive effect of CL vs TL in influencing students’ attitudes. The results indicated that major biology men have a higher positive attitude toward biology in CL classes compared to TL classes in both single-gender and mix-gender. Men have higher positive attitudes in CL mixed-gender groups (2M+2W) than men in CL single-gender groups in major and nonmajor classes. In contrast, women developed a higher level of a positive attitude toward biology in CL (4W) than TL single-gender classes and a negative attitude toward biology in CL (2M+2W) compared to TL mix-gender classes in both major and nonmajor classes. Women in the single-gender have a significantly higher positive attitude toward biology in CL (4W) group compared to TL groups. However, in mix-gender groups, women students developed higher positive attitudes in TL compared to CL. Overall, the results indicate that the interaction of gender and gender composition depends on the teaching method for both major and nonmajor biology students, and there were influential effects of CL and TL pedagogies in cultivating and nurturing attitudes.

## Discussion

In measuring the nine factors of the attitudinal scale administered at an undergraduate biology class of men and women in single and mixed-gender, applying CL and TL pedagogies in major and nonmajor classes in accordance with our hypothesis, we found that women showed a significantly greater positive attitude towards biology learning than men in single-gender TL and CL classes. The findings confirm gender-specific variations in attitudes as different pedagogies affected men and women differently. While women disliked mix-gender groping, men preferred mix-gender (2W+2M) group interventions. Women in major and nonmajor single-gender CL groups had the highest positive attitude towards biology with a large effect size compared to mix-gender CL. In nonmajor and major classes, men in mixed-gender CL (2W+2M) sections had the highest positive attitude as they enjoyed it more and experienced greater satisfaction toward biology with a large effect size compared to single-gender TL groups where they had the lowest attitude toward biology see Tables 2–5. These outcomes might be because men students favor a mix-gender group setting where women’s existence in the same group triggers their desire to collaborate better, getting more engaged in the subject and learning process. However, women students in mix-gender CL (2W+2M) groups had diminished attitudes toward biology, feeling anxious, and did not enjoy the mix-gender classes for both nonmajor and major sections. The effect size was more negative in nonmajor sections than the biology major since biology major students took the course more seriously because of their plans to proceed with their future biology-related careers, and nonmajor students took the biology course as an elective. Women experienced greater satisfaction and favored to study in same-gender groups. These results indicate that men and women students are different in their attitudes toward biology in groups with different gender combinations. These variations can be observed in each study discussed in the subsequent section.

### Effects of CL vs TL in single-gender nonmajor men and women’s attitudes

Our first study of nonmajor single-gender found that women experienced a significantly higher attitude score of general interest in biology compared to men in both TL and CL methods. See Table 2. These findings are consistent with Uitto’s findings that women, in general, have a higher attitude in biology as compared to men [106]. In contrast, Soltani (2011) found a similar positive attitude towards biology between girls and boys [9].

In our study, when Cohen’s d effect size was applied, it was found that the effect size of women (d = 0.6240) to be of a medium effect size while men (d = 1.2460) to be of large effect size. Thus, indicating that even though the p-values are significantly below 0.05 for both genders, the values of Cohen’s d illustrate that the effect size on the women was not as high as it was on men. Men showed a higher attitude than women in mix-gender nonmajor CL classes as well as in mix-gender major (2W+2M) group learning situations. This can be due to Kuwait’s unique socio-cultural beliefs, men superiority, and students are used to learning in gender-segregated classrooms. The CL pedagogy has a higher influence on attitudes over men compared to women in mixed-gender classes. This could be that men’s attitude from the beginning was not positive; once placed in a more practical CL setting where they could better understand the concepts, they developed a more positive attitude. Thus team-oriented learning in CL classes influenced men to develop an interest in biology.

Career motivation has been the least influential factor for men. Men are more comfortable moving things through space and better suited to using diagrams and pictures [107], which explains the results we have since men believe biology is more theoretical than practical. Among these stereotypical beliefs, a stereotype that has been present is that biology is for women, and physics is for men [108], which may be an attribute of why men in our study do not see themselves pursuing careers in biology. Our findings are consistent with Baram‐Tsabari & Yarden’s (2008) and Kang et al.’s (2018), and Hagay et al.’s (2012) research. Especially in the Middle Eastern countries, social, cultural, religious, and national affiliations and gender have been the influential factors affecting students’ attitudes towards biology [24, 108, 109].

Koballa and Glynn (2007), based on social cognitive theory, posit that student motivation has an attitudinal dimension related to their beliefs about career and grade motivation. Career and grade motivation is found to be higher among women compared to men [100]. Assessing students’ attitudes found to be linked to students’ choice or selecting science subjects [110], developing interest in science disciplines [111], student enrollment, and retention [112]. These findings evident that socio-cultural stereotypes affected men as well as women in selecting future career decisions.

In terms of assessment anxiety factor, irrespective of specific pedagogies, there was assessment anxiety among both women and men. In contrast, Kisoglu (2018) found no difference between women and men regarding testing anxiety. Kisoglu’s study on high school students found that test anxiety showed the highest mean score among other factors. This means that the students were anxious about the biology subject as the study mentions that the students are concerned about the lessons’ difficulty [27]. Similarly, Ali and Mohsin’s (2013) study found that assessment anxiety was higher in women than men. Glynn (2009) suggests that the reason behind women’s high assessment anxiety was due to social-cultural factors [69]. In this regard, it is important to understand Kuwait’s socio-cultural context associated with the education system.

### The contextual effect on gender and attitudes

Public education in Kuwait consists of segregated public schools, where gender-separated students are taught by teachers of similar gender [113, 114]. In Kuwait, girls in public schools go to all-girls schools with all women teachers from the first grade until the twelfth grade. Whereas boys go to all-men schools with women teachers until the sixth grade, then start having all men teachers until the twelfth class [115]. Especially in the Middle Eastern countries, women’s participation in science-related occupations is low as 25% of the total workforce [116] regardless of the average educational expenditure has increased to 16% of their total national wealth [4]. Some other researchers suggest that students’ negative perceptions that science subjects are challenging to learn and a lack of motivation for science subjects diminishes enrollments [2]. Some other reasons discussed in the low enrollment of women students in science subjects related to socio-cultural and family responsibilities and associated restrictions and negative perceptions [116]. Social, cultural, and religious parameters in the Middle Eastern countries such as epistemology, values, beliefs, communication patterns, and gender-specific role expectations affect students’ learning experience; thus, contextual factors exemplify the attitudes towards science learning [24, 117]. Despite many efforts to promote equitable enrollments, the low enrollment in science fields is due to low aspirations in science careers and a lack of interest in science subjects. Thus the attitudinal dimension comes to our focus [81]. Such contextual effects have been reflected in our study results that women showed negative attitudes when mixed-gender groups contrast to positive attitudes in single-gender groups. Therefore, our findings help develop context-specific learning pedagogies with activities to improve positive attitudes toward science education.

### Pedagogical effects in mixed-gender, nonmajor students’ attitudes

In mix-gender nonmajor Table 3, CL pedagogy negatively affected women’s attitude in all nine attitude factors as per Table 3. As mentioned above, the contextual factors and gender segregation practices from childhood could have influenced attitudinal differences between men and women in our study [115]. Women’s negative attitude occurs when placed in mixed groups, which could be due to women being uncomfortable when placed in groups with men (2M+2W). Previous research shows that girls in high schools performed better in single-gender groups when the task at hand is new. However, women performed better in mixed groups when the assigned task is familiar [118]. Similarly, women placed in a mixed-gender group were less task-oriented than in single-gender groups, whereas men were more task-oriented in mix-gender groups than single-gender groups [119]. This outcome could be an attribute that the women’s confidence level around men and the sense of confidence that comes when the topic is familiar, which could be another factor for the negative attitude of women in mixed groups and the reason behind why women in our study the highest negative influential factors had were self-efficacy, assessment anxiety, and grade motivation. The consistency in the differences in values from TL to CL in women and men means that women feel less comfortable working in mixed-gender groups, whereas men are unaffected, thus benefitted from the CL environment more. This change in attitude is because women are too shy to engage in learning equally with men, while men tend to show off their skills in front of the women, which makes them work harder, thus affecting socio-psychological parameters.

When Cohen’s d effect size was applied, we found that the effect size of women (d = 0.7091) to be of a medium effect size while men (d = 1.8417) to be of large effect size. Thus, similar to the single nonmajor category, these values indicate that even though the p-values are significantly below 0.05 for both genders, Cohen’s d illustrates that women’s effect size was not as powerful as it was on men. Thus, male superiority’s context-specific societal ideologies can be seen as an underlying driving factor for men to show superior performance when women are present in groups.

For nonmajor students, women in college taking biology courses were found to have a higher career motivation than men, which is consistent with previous findings [120]. Our findings are consistent with Yeoh & Ierardi’s (2015)s’ conclusion that women in nonmajor biology have a higher career motivation than men. However, the overall significance was ranged between low to medium, which is not very significant [69]. In our study, career motivation was not a major influence on students as compared to other factors. However, the influence of all the other factors in mixed-gender CL is higher for men than single-gender, which leads to the assumption that men’s overall attitude is favorable towards mixed-gender CL grouping.

Women’s self-determination W(TL) μ = 2.80, SD: 0.39; W(CL): M = 2.43, SD: 0.4) reduced significantly in a CL environment while men were more determined (M(TL) μ = 2.64, SD: 0.44; M(CL)μ = 3.55, SD: 0.55) when working with women in CL. We found contrasting differences in the attitude of men and women in self-efficacy and self-determination factors. According to our literature review, this is an interesting result because women usually have higher self-determination than men in biology courses [69, 87, 120], but when they were placed in CL mixed-gender groups, women’s self-determination dropped significantly. This result can be an attribute of socio-cultural beliefs because women had to learn in an unusual learning environment with men. We conclude that rather than the pedagogy, mixed-gender grouping affected women diminishing self-determination. Thus socio-cultural beliefs act as an influencing factor.

### Pedagogical effects on attitudes in major single-gender classes

Similar to Stump et al.’s findings, single-gender major in Table 4 found that women prefer to collaborate more often as they believe it is a successful learning strategy compared to men [121]. In our results, for both major and nonmajor, we found that men benefited as they developed a higher positive attitude towards Biology in mixed-gender groups as compared to women.

In terms of factors, both women and men had a higher effect on attitude in career motivation. This result makes sense since students who are already majoring in biology are motivated to learn science as they exhibit an assertive behavior of interest in science-related careers [122, 123]. A previous study mentioned that regression analysis revealed that interest and self-efficacy influenced women’s and men’s career motivation in high school [106]. However, in our study, women had the highest negative influence on self-efficacy, assessment anxiety, and grade motivation in mixed-gender (2W+2M) groups, thus reflecting the influence of socio-cultural belief of gender segregation.

### Pedagogical effect on attitudes on major mix-gender groups learning

In the mix-gender major, Table 5 shows that women in mixed-group CL classes in biology major demonstrated negative attitudes compared to single-gender classes. These findings are similar to what is resulted in nonmajor biology, which is consistent with the meta-analysis done by Hmelo-Silver (2017), where he mentioned that mixed gender grouping might affect women negatively in STEM domains [124]. Zeid and El-Bahey (2011) found that both genders’ overall course performance has improved by changing the software engineering classroom composition from a mixed to a single-gender classroom in a Gulf Cooperation Council (GCC) country [125]. However, the conclusions of Cen et al. (2016) and Le et al. (2017), a mixed-gender group is found to be more effective than a single-gender group due to variations of thoughts and creativity of both genders and having smaller groups are more effective than bigger groups that allow students a better chance in understanding each other’s ideas [48, 126]. Glynn (2009), Glynn (2011), and Yeoh (2015) found that women had higher self-determination than men studying science and biology as they believe that they have a more substantial control towards the subject; this is consistent with our results when women were placed in single-gender groups [69, 87, 120]. However, in our study, men had higher self-determination as compared to women in mixed-gender groups. Thus, in all mixed-gender classes, women showed negative attitudes as they disliked being mixed with men in groups. In addition to socio-cultural influences, we question that it can be due to women felt weaker when men take a dominant role in mixed-gender groups. These findings are consistent with Pahke et al.’s (2014) findings of separating girls and boys in classrooms to increase girls’ interest in academic learning. Our study shows that in Kuwait’s specific socio-cultural environment, irrespective of the pedagogical methods, women’s attitudes are affected due to gender variables. In contrast, men developed positive attitudes and general interest towards biology learning in CL mixed-gender group environment. In contrast, Freeman et al. (2014)’s conclusion that active learning enhances students’ performance and interest in STEM learning [127] is somewhat argumentative when compared to the findings of our study because women in both single-gender major and nonmajor and CL and TL classes demonstrated a higher level of positive attitudes and benefitted from both pedagogies. In summary, both the pedagogical method and gender have been critical variables of framing and nurturing positive or negative attitudes towards science learning.

### Contribution and novelty of the current research

Since this is the first comprehensive study carried out in measuring the effectiveness of teaching pedagogies on attitudes of men and women in a university in Kuwait, it contributes to the rare literature on how has teaching pedagogies impact framing attitudes towards biology education. A combination of teaching pedagogy and the development of an effective gender-specific learning environment could contribute to gender equality and encouragement for future careers related to biology. Previous research discussed students’ gender and context-specific cultural characteristics are essential determinants of gender equity in science education in many countries, including Islamic societies in the Middle East [128]. However, the current study is the first to examine the effect of pedagogical methods in forming positive or negative attitudes towards learning biology in Kuwait that is culturally considered a gender-segregated society. There is a higher gender inequity in science education. This study’s findings will impact designing gender and culture-specific teaching pedagogies that can positively impact attracting women and men in science education in Kuwait. Gender separation still a factor of inequitable education for men and women in science education in the Middle Eastern countries where similar socio-cultural contexts exist. We expect that the findings will lead to a rethinking of culture-specific pedagogies specific to Middle Eastern societies to align with unique social and cultural ideologies.

## Implications

### Theoretical implications

From a biological perspective, factors such as classroom environment, attitudes, instructors’ instructional methods, and learning styles affect individuals’ brains’ neurophysiology [129]. Based on Tanner and Allen’s (2004) research, due to the human brain’s plasticity, individuals respond differently to different learning environments, learning methods, and activities, thus forming attitudes and beliefs that ultimately relate to learning outcomes [129]. Gardner’s (2011) theory of multiple intelligence posits visual, auditory, and kinesthetic sensory modes affect learner preferences thus, forming attitudes [130]. Kolb’s (2006) learning style theory focuses on an individual’s abilities. However, Kolb’s learning style theory and Gardner’s multiple intelligence theory focus on individuals’ inner psychological variations and students’ perceptions [110, 131]. There are inadequacies of integrating gender and context-specific elements in these theories. Therefore, our research findings will lead to a new conceptualization beyond learning style theories [130]. We argue that scrutinized instructional methods integrated with contextual and socio-cultural, and socio-ideological elements into a diversity of activities would influence changing students’ stereotypical beliefs into positive attitudes. Future researchers would have the potential for a new theoretical dimension to accommodate gender differences, socio-cultural and socio-ideological influences in science teaching in different contexts.

### Implications for stakeholders

This research findings inform future researchers, educators and instructors to develop socio-cultural, and socio-psychological context-specific innovative teaching pedagogies to enhance positive attitudes in science education resulting in gender equity. There is a need to develop an appropriate curriculum to integrate a variety of activities and teaching pedagogies depending on student population, gender, and socio-cultural contexts in addition to their learning styles. To attract and retain women and minority students, educators may develop strategic approaches by changing the curriculum and teaching pedagogies, including course design, selecting various contexts, and gender-specific learning components to stimulate students’ positive attitudes toward science education. Educators, instructors, and policymakers need to consider students’ future career trajectories in different contexts such as men’s dominance and superiority, women’s underrepresentation in science fields, gender-specific perceptions, and attitudes in developing policies. The policy should not limit only to selecting appropriate pedagogies, classroom activities, gender division in groups, but it also needs to guide appropriate teaching modules and multiple strategic activities. Policy development and decision-making facilitate teachers to select appropriate classroom environments that spur students’ positive attitudes.

This policy should lead to instructor freedom to go beyond traditional teaching and learning styles to accommodate different and diversified instructions and activities to accommodate more diverse groups of students in science education. Instructors need to have the flexibility to structure their classes, and they need to be trained on improving classroom practices, solving problems, and adopting a variety of learning modules depending on mixed-gender or single-gender classes. Thus, each class becomes a unique and independent learning cluster. Students should have options to enroll in either mixed-gender or single-gender classes in different contexts. Nevertheless, it is ensured context-specific learning assignments and module selection while the basics of biology are in the center of the course learning.

### Limitations and future directions

Though this is the first comprehensive study conducted in a university in Kuwait at the undergraduate level, it has several limitations. First, this study is limited to one semester-long course, and it would be beneficial for future research to replicate similar research for a longitudinal period for generalizability. Kuwait has a unique culture, and students are mainly Kuwaiti nationals except a few. Due to students’ homogeneous cultural background, we could not assess students’ attitudes from different cultural and ethnic backgrounds. Therefore, we could not analyze and compare the cultural variations of attitudes between men and women of other ethnicities and socio-cultural backgrounds. Kuwait is predominantly using single-gender classes from kindergarten to graduate levels, and mixed-gender classes are rarely used at the undergraduate level. Thus, there can be socio-cultural effects unique to Kuwait that may have influenced cultivating and enhancing positive or negative attitudes towards mixed-gender and single-gender classes. Next, we customized two attitudinal scales validated by Russel and Hollander (1975) and Glynn et al. (2007) and later validated by Aydeniz and Kotowsk (2014). We suggest using various attitudinal scales for future studies and comparing and validating the findings of this study. Further, this study used CL and TL as the dominant pedagogical models, future studies could use various other pedagogies such as cooperative and experimental learning, and similar studies could be extended to STEM education in Kuwait. Lastly, this study is limited to one country, and it would be useful to conduct multi-country studies, especially in the Arab region, where slightly different socio-cultural variations exist.

## Conclusion

The findings advance our understanding of the pedagogical effect on cultivating positive or negative attitudes among men and women. The results confirm that teaching pedagogy, gender division, and gender-group composition in the classroom matters in fostering positive or negative attitudes, interest, and motivation towards biology education. In Kuwait’s specific situation, gender and teaching method combination has become one-factor determining men’s and women’s future career trajectories. The results show an interaction effect demonstrating that the teaching pedagogies could impact students’ attitudes and how they impact differently according to student gender. While men preferred mixed-gender group learning, women preferred single-gender groups as the best venue for them to enjoy and excel in biology. A combination of teaching pedagogy and creating an appropriate gender-specific learning environment could result in gender equity in biology education and a motivator for future biology-related careers. Selecting and developing appropriate pedagogies considering students’ gender and gender-specific parameters would help educators generate positive attitudes for science careers and prepare the future workforce. Lastly, these findings suggest a need to develop gender-specific and context-specific learning pedagogies in teaching biology to improve students’ attitudes toward science subjects to reduce the increasing healthcare and scientific workforce gap. These findings can be generalized to similar socio-cultural environments.

## Supporting information

S1 File (PDF)

S1 Questionnaire (PDF)
