# Correction: Measuring attitudes towards biology major and non-major: Effect of students’ gender, group composition, and learning environment

**DOI:** 10.1371/journal.pone.0269052

**Published:** 2022-05-23

**Authors:** Firas Almasri, Gertrude I. Hewapathirana, Fatme Ghaddar, Nick Lee, Bashar Ibrahim

The affiliation for the fifth author is incorrect. Bashar Ibrahim is not affiliated with #1 but with #2: Department of Mathematics and Natural Sciences, Gulf University for Science and Technology, Hawally, Kuwait.
