# Supplementary material for: Measuring attitudes towards biology major and non-major: Effect of students’ gender, group composition, and learning environment
Source: PLoS One. 2021 May 14;16(5):e0251453. doi: 10.1371/journal.pone.0251453 (PMC8121319; doi:10.1371/journal.pone.0251453)
Supplement: S1 File — (PDF) [file pone.0251453.s001.pdf]

Supplementary Documents

Table of Contents

Three-way ANOVA 2
- Non-Major Biology Students 2
- Major Biology Students 12

Two-Way ANOVA 22
- Non-Major Biology 22
  - Single Gender Students 22
  - Mixed Gender Students 26
- Major Biology 30
  - Single Gender Students 30
  - Mixed Gender Students 37

Attitude Factors Analysis (Each Factor) 43
- Non-Major Biology 43
  - Single Gender Students 43
  - Mixed Gender Students 122
- Major Biology 202
  - Single Gender Students 202
  - Mixed Gender Students 281

Questionnaire 360

Three-way ANOVA
Non-Major Biology Students

Univariate Analysis of Variance

Between-Subjects Factors

		Value Label	N
Gender	1	Women	1187
	2	Men	1188
Method	1	TL	1160
	2	CL	1215
Gender Composition	1.00	Single-Gender	1106
	2.00	Mix-Gender	1269

Table (1.1.1) Between-Subjects Factors including Gender, Method and Gender Composition

Descriptive Statistics

Dependent Variable: Attitude

Gender	Method	Gender Composition	Mean	Std. Deviation	N
Women	TL	Single-Gender	3.7002	.64419	269
		Mix-Gender	3.0594	.55055	315
		Total	3.3546	.67542	584
	CL	Single-Gender	4.1053	.64930	279
		Mix-Gender	2.5969	.65646	324
		Total	3.2948	.99623	603
	Total	Single-Gender	3.9064	.67726	548
		Mix-Gender	2.8249	.64877	639
		Total	3.3242	.85376	1187
Men	TL	Single-Gender	2.5393	.60263	270
		Mix-Gender	2.6899	.60389	306
		Total	2.6193	.60746	576
	CL	Single-Gender	3.2847	.59377	288
		Mix-Gender	3.7489	.55266	324
		Total	3.5305	.61712	612
	Total	Single-Gender	2.9240	.70433	558
		Mix-Gender	3.2346	.78375	630
		Total	3.0887	.76310	1188
Total	TL	Single-Gender	3.1186	.85197	539
		Mix-Gender	2.8774	.60588	621
		Total	2.9895	.74014	1160

	CL	Single-Gender	3.6885	.74461	567
		Mix-Gender	3.1729	.83660	648
		Total	3.4135	.83529	1215
	Total	Single-Gender	3.4108	.84770	1106
		Mix-Gender	3.0283	.74730	1269
		Total	3.2064	.81804	2375

Table (1.1.2) Descriptive statistics with the attitude as the dependent variable where the mean, standard deviation, and the total number of students per category have been specified

Tests of Between-Subjects Effects

Dependent Variable: Attitude

Source	Type III Sum of Squares	df	Mean Square	F	Sig.	Partial Eta Squared
Corrected Model	717.095[a]	7	102.442	278.219	.000	.451
Intercept	24425.430	1	24425.430	66336.071	.000	.966
Gender	53.063	1	53.063	144.110	.000	.057
Method	112.662	1	112.662	305.975	.000	.114
GenderComposition	86.880	1	86.880	235.955	.000	.091
Gender * Method	127.940	1	127.940	347.467	.000	.128
Gender * GenderComposition	281.974	1	281.974	765.803	.000	.244
Method * GenderComposition	11.332	1	11.332	30.777	.000	.013
Gender * Method * GenderComposition	51.493	1	51.493	139.848	.000	.056
Error	871.547	2367	.368			
Total	26006.181	2375				
Corrected Total	1588.642	2374				

a. R Squared = .451 (Adjusted R Squared = .450)

Table (1.1.3) Tests where gender, method and gender composition have been analysed to find the Type III sum of squares, df, mean square, F value, significance and partial eta squared.

1.1.4 Estimated Marginal Means

1.1.4.1 Gender

Estimates

Dependent Variable: Attitude

Gender	Mean	Std. Error	95% Confidence Interval Lower Bound	95% Confidence Interval Upper Bound
Women	3.365	.018	3.331	3.400
Men	3.066	.018	3.031	3.100

Table (1.1.4.1.1) Estimates of women and men with the attitude as the dependent variable

Pairwise Comparisons

Dependent Variable: Attitude

(I) Gender	(J) Gender	Mean Difference (I-J)	Std. Error	Sig.[b]	95% Confidence Interval for Difference[b] Lower Bound	95% Confidence Interval for Difference[b] Upper Bound
Women	Men	.300*	.025	.000	.251	.349
Men	Women	-.300*	.025	.000	-.349	-.251

Table (1.1.4.1.2)

Based on estimated marginal means

*. The mean difference is significant at the .05 level.

b. Adjustment for multiple comparisons: Least Significant Difference (equivalent to no adjustments).

Univariate Tests

Dependent Variable: Attitude

	Sum of Squares	df	Mean Square	F	Sig.	Partial Eta Squared
Contrast	53.063	1	53.063	144.110	.000	.057
Error	871.547	2367	.368			

Table (1.1.4.1.3)

The F tests the effect of Gender. This test is based on the linearly independent pairwise comparisons among the estimated marginal means.

Univariate Tests

Dependent Variable: Attitude

Gender	Gender Composition		Sum of Squares	df	Mean Square	F	Sig.	Partial Eta Squared
Women	Single-Gender	Contrast	22.479	1	22.479	61.051	.000	.025
		Error	871.547	2367	.368			
	Mix-Gender	Contrast	34.162	1	34.162	92.780	.000	.038
		Error	871.547	2367	.368			
Men	Single-Gender	Contrast	77.442	1	77.442	210.321	.000	.082
		Error	871.547	2367	.368			
	Mix-Gender	Contrast	176.484	1	176.484	479.306	.000	.168
		Error	871.547	2367	.368			

Table (1.1.4.1.4)

Each F tests the simple effects of Method within each level combination of the other effects shown. These tests are based on the linearly independent pairwise comparisons among the estimated marginal means.

1.1.4.2 Method

Estimates

Dependent Variable: Attitude

Method	Mean	Std. Error	95% Confidence Interval Lower Bound	95% Confidence Interval Upper Bound
TL	2.997	.018	2.962	3.032
CL	3.434	.017	3.400	3.468

Table (1.1.4.2.1) Estimates for the teaching methods with the attitude of the students as the dependent variable

Pairwise Comparisons

Dependent Variable: Attitude

(I) Method	(J) Method	Mean Difference (I-J)	Std. Error	Sig.[b]	95% Confidence Interval for Difference[b] Lower Bound	Upper Bound
TL	CL	-.437*	.025	.000	-.486	-.388
CL	TL	.437*	.025	.000	.388	.486

Table (1.1.4.2.2)

Based on estimated marginal means

*. The mean difference is significant at the .05 level.

b. Adjustment for multiple comparisons: Least Significant Difference (equivalent to no adjustments).

Univariate Tests

Dependent Variable: Attitude

	Sum of Squares	df	Mean Square	F	Sig.	Partial Eta Squared
Contrast	112.662	1	112.662	305.975	.000	.114
Error	871.547	2367	.368			

Table (1.1.4.2.3)

The F tests the effect of Method. This test is based on the linearly independent pairwise comparisons among the estimated marginal means.

1.1.4.3. <u>Gender Composition</u>

Estimates

Dependent Variable: Attitude

Gender Composition	Mean	Std. Error	95% Confidence Interval Lower Bound	Upper Bound
Single-Gender	3.407	.018	3.372	3.443
Mix-Gender	3.024	.017	2.990	3.057

Table (1.1.4.3.1) Estimates for the gender composition with the attitude of the students as the dependent variable

Pairwise Comparisons

Dependent Variable: Attitude

(I) Gender Composition	(J) Gender Composition	Mean Difference (I-J)	Std. Error	Sig.[b]	95% Confidence Interval for Difference[b]	
					Lower Bound	Upper Bound
Single-Gender	Mix-Gender	.384*	.025	.000	.335	.433
Mix-Gender	Single-Gender	-.384*	.025	.000	-.433	-.335

Based on estimated marginal means

*. The mean difference is significant at the .05 level.

b. Adjustment for multiple comparisons: Least Significant Difference (equivalent to no adjustments).

Table (1.1.4.3.2)

Univariate Tests

Dependent Variable: Attitude

	Sum of Squares	df	Mean Square	F	Sig.	Partial Eta Squared
Contrast	86.880	1	86.880	235.955	.000	.091
Error	871.547	2367	.368			

Table (1.1.4.3.3)

The F tests the effect of Gender Composition. This test is based on the linearly independent pairwise comparisons among the estimated marginal means.

1.1.4.4 Gender * Method

Estimates

Dependent Variable: Attitude

Gender	Method	Mean	Std. Error	95% Confidence Interval	
				Lower Bound	Upper Bound
Women	TL	3.380	.025	3.330	3.429
	CL	3.351	.025	3.303	3.400
Men	TL	2.615	.025	2.565	2.664
	CL	3.517	.025	3.469	3.565

Table (1.1.4.4.1) Estimates for the gender*method with the attitude of the students as the dependent variable

1.1.4.5 Gender * Gender Composition

Estimates

Dependent Variable: Attitude

Gender	Gender Composition	Mean	Std. Error	95% Confidence Interval Lower Bound	95% Confidence Interval Upper Bound
Women	Single-Gender	3.903	.026	3.852	3.954
	Mix-Gender	2.828	.024	2.781	2.875
Men	Single-Gender	2.912	.026	2.862	2.962
	Mix-Gender	3.219	.024	3.172	3.267

Table (1.1.4.5.1) Estimates for the gender*gender composition with the attitude of the students as the dependent variable

1.1.4.6 Method * Gender Composition

Estimates

Dependent Variable: Attitude

Method	Gender Composition	Mean	Std. Error	95% Confidence Interval Lower Bound	95% Confidence Interval Upper Bound
TL	Single-Gender	3.120	.026	3.068	3.171
	Mix-Gender	2.875	.024	2.827	2.922
CL	Single-Gender	3.695	.025	3.645	3.745
	Mix-Gender	3.173	.024	3.126	3.220

Table (1.1.4.6.1) Estimates for the method*gender composition with the attitude of the students as the dependent variable

1.1.4.7 Gender * Method * Gender Composition

Estimates

Dependent Variable: Attitude

Gender	Method	Gender Composition	Mean	Std. Error	95% Confidence Interval Lower Bound	95% Confidence Interval Upper Bound
Women	TL	Single-Gender	3.700	.037	3.628	3.773
		Mix-Gender	3.059	.034	2.992	3.126
	CL	Single-Gender	4.105	.036	4.034	4.177
		Mix-Gender	2.597	.034	2.531	2.663
Men	TL	Single-Gender	2.539	.037	2.467	2.612
		Mix-Gender	2.690	.035	2.622	2.758

	CL	Single-Gender	3.285	.036	3.215	3.355
		Mix-Gender	3.749	.034	3.683	3.815

Table (1.1.4.7.1) Estimates for the gender*method*gender composition with the attitude of the students as the dependent variable

Pairwise Comparisons

Dependent Variable: Attitude

Gender	Gender Composition	(I) Method	(J) Method	Mean Difference (I-J)	Std. Error	Sig.[b]	95% Confidence Interval for Difference[b] Lower Bound	Upper Bound
Women	Single-Gender	TL	CL	-.405*	.052	.000	-.507	-.303
		CL	TL	.405*	.052	.000	.303	.507
	Mix-Gender	TL	CL	.462*	.048	.000	.368	.557
		CL	TL	-.462*	.048	.000	-.557	-.368
Men	Single-Gender	TL	CL	-.745*	.051	.000	-.846	-.645
		CL	TL	.745*	.051	.000	.645	.846
	Mix-Gender	TL	CL	-1.059*	.048	.000	-1.154	-.964
		CL	TL	1.059*	.048	.000	.964	1.154

Table (1.1.4.7.2)

Based on estimated marginal means

*. The mean difference is significant at the .05 level.

b. Adjustment for multiple comparisons: Least Significant Difference (equivalent to no adjustments).

1.1.5. <u>Profile Plots</u>

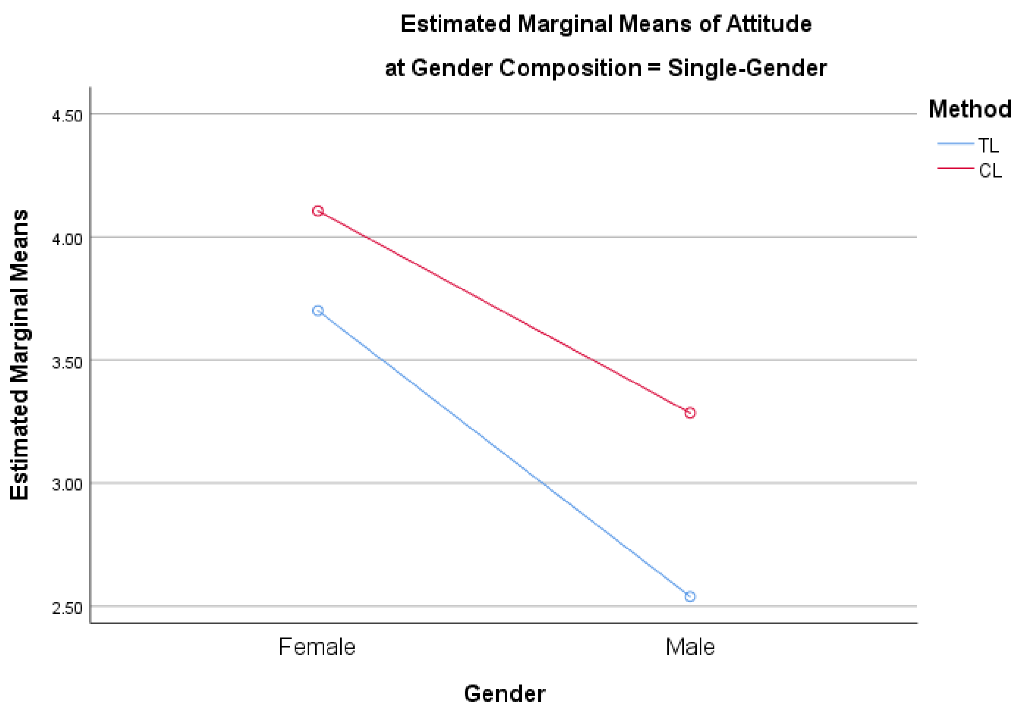

Figure (1.1.5.1)

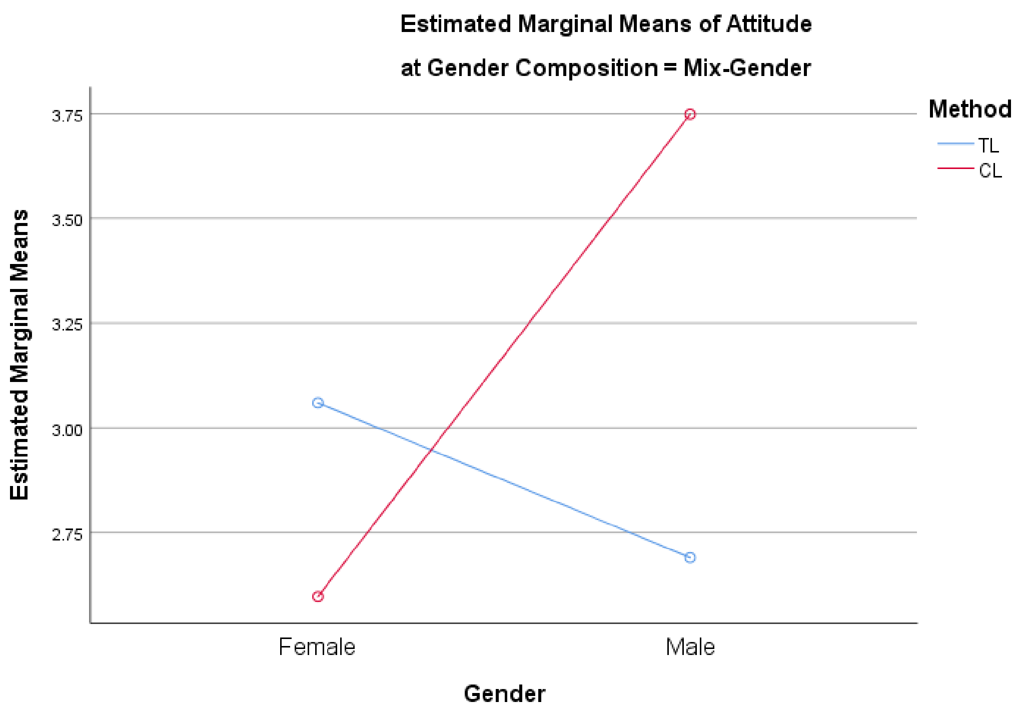

Figure (1.1.12.2)

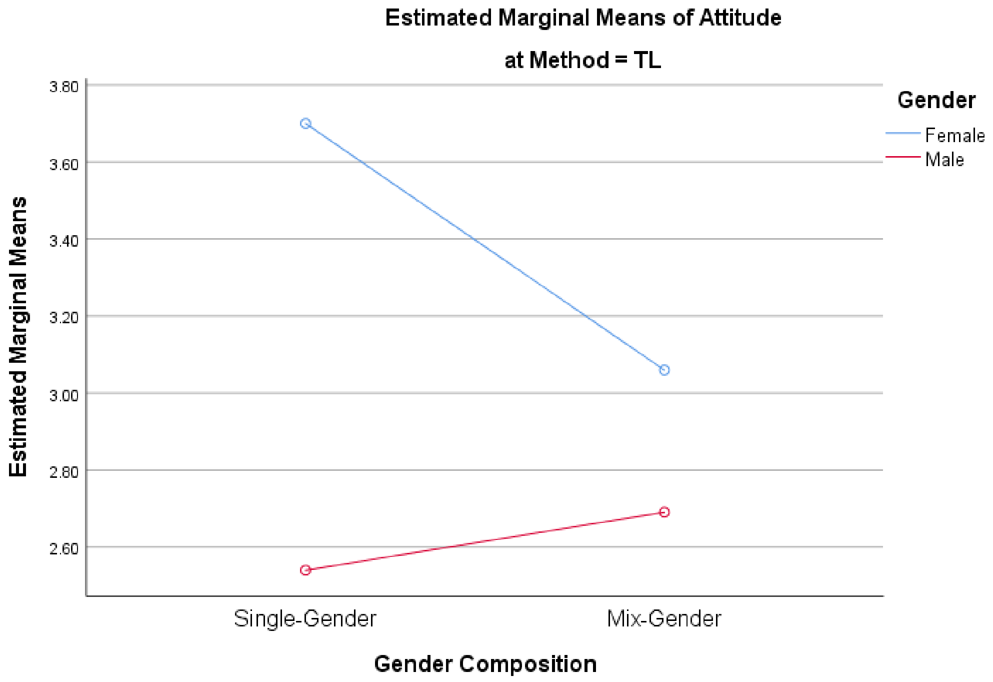
Estimated Marginal Means of Attitude
at Method = TL
Estimated Marginal Means
3.80
3.60
3.40
3.20
3.00
2.80
2.60
Single-Gender
Mix-Gender
Gender Composition
Gender
Female
Male

Figure (1.1.5.3)

Major Biology Students

Univariate Analysis of Variance

Between-Subjects Factors

		Value Label	N
Gender	1	Women	1089
	2	Men	1108
Method	1	TL	1053
	2	CL	1144
Gender Composition	1.00	Single-Gender	1106
	2.00	Mix-Gender	1091

Table (1.2.1) Between-Subjects Factors including Gender, Method and Gender Composition

Descriptive Statistics

Dependent Variable: Attitude

Gender	Method	Gender Composition	Mean	Std. Deviation	N
Women	TL	Single-Gender	4.0821	.64667	270
		Mix-Gender	4.1311	.65403	252
		Total	4.1057	.65007	522
	CL	Single-Gender	4.7001	.53671	278
		Mix-Gender	3.6174	.80113	289
		Total	4.1482	.87236	567
	Total	Single-Gender	4.3956	.66869	548
		Mix-Gender	3.8567	.77904	541
		Total	4.1279	.77376	1089
Men	TL	Single-Gender	3.8020	.75679	270
		Mix-Gender	3.9946	.64565	261
		Total	3.8966	.71026	531
	CL	Single-Gender	4.4970	.62199	288
		Mix-Gender	4.6639	.63349	289
		Total	4.5806	.63277	577
	Total	Single-Gender	4.1607	.77252	558
		Mix-Gender	4.3463	.72102	550
		Total	4.2528	.75281	1108
Total	TL	Single-Gender	3.9420	.71707	540
		Mix-Gender	4.0616	.65273	513
		Total	4.0003	.68876	1053
	CL	Single-Gender	4.5967	.58997	566
		Mix-Gender	4.1407	.89160	578

		Total	4.3663	.79082	1144
	Total	Single-Gender	4.2771	.73211	1106
		Mix-Gender	4.1035	.78899	1091
		Total	4.1909	.76565	2197

Table (1.2.2) Descriptive statistics with the attitude as the dependent variable where the mean, standard deviation, and the total number of students per category have been specified

Tests of Between-Subjects Effects

Dependent Variable: Attitude

Source	Type III Sum of Squares	df	Mean Square	F	Sig.	Partial Eta Squared
Corrected Model	313.789[a]	7	44.827	100.793	.000	.244
Intercept	38410.011	1	38410.011	86364.102	.000	.975
Gender	6.240	1	6.240	14.030	.000	.006
Method	73.877	1	73.877	166.110	.000	.071
Gender*Composition	15.567	1	15.567	35.003	.000	.016
Gender * Method	54.388	1	54.388	122.290	.000	.053
Gender * Gender Composition	66.490	1	66.490	149.501	.000	.064
Method * Gender Composition	45.872	1	45.872	103.143	.000	.045
Gender * Method * Gender Composition	41.899	1	41.899	94.208	.000	.041
Error	973.547	2189	.445			
Total	39874.263	2197				
Corrected Total	1287.336	2196				

a. R Squared = .244 (Adjusted R Squared = .241)

Table (1.2.3) Tests where gender, method and gender composition have been analysed to find the Type III sum of squares, df, mean square, F value, significance and partial eta squared.

1.2.4 Estimated Marginal Means

1.2.4.1 Gender

Estimates

Dependent Variable: Attitude

Gender	Mean	Std. Error	95% Confidence Interval Lower Bound	Upper Bound
Women	4.133	.020	4.093	4.172
Men	4.239	.020	4.200	4.279

Table (1.2.4.1.1) Estimates of women and men with the attitude as the dependent variable

Pairwise Comparisons

Dependent Variable: Attitude

(I) Gender	(J) Gender	Mean Difference (I-J)	Std. Error	Sig.[b]	95% Confidence Interval for Difference[b] Lower Bound	Upper Bound
Women	Men	-.107*	.028	.000	-.163	-.051
Men	Women	.107*	.028	.000	.051	.163

Table (1.2.4.1.2)

Based on estimated marginal means

*. The mean difference is significant at the .05 level.

b. Adjustment for multiple comparisons: Least Significant Difference (equivalent to no adjustments).

Univariate Tests

Dependent Variable: Attitude

Gender	Gender Composition		Sum of Squares	df	Mean Square	F	Sig.	Partial Eta Squared
Women	Single-Gender	Contrast	52.306	1	52.306	117.609	.000	.051
		Error	973.547	2189	.445			
	Mix-Gender	Contrast	35.524	1	35.524	79.875	.000	.035
		Error	973.547	2189	.445			
Men	Single-Gender	Contrast	67.315	1	67.315	151.356	.000	.065

	Error	973.547	2189	.445			
Mix-Gender	Contrast	61.451	1	61.451	138.171	.000	.059
	Error	973.547	2189	.445			

Table (1.2.4.1.3)

Each F tests the simple effects of Method within each level combination of the other effects shown. These tests are based on the linearly independent pairwise comparisons among the estimated marginal means.

1.2.4.2. Method

Estimates

Dependent Variable: Attitude

Method	Mean	Std. Error	95% Confidence Interval Lower Bound	Upper Bound
TL	4.002	.021	3.962	4.043
CL	4.370	.020	4.331	4.408

Table (1.2.4.2.1) Estimates for the teaching methods with the attitude of the students as the dependent variable

Pairwise Comparisons

Dependent Variable: Attitude

(I) Method	(J) Method	Mean Difference (I-J)	Std. Error	Sig.[b]	95% Confidence Interval for Difference[b] Lower Bound	Upper Bound
TL	CL	-.367*	.028	.000	-.423	-.311
CL	TL	.367*	.028	.000	.311	.423

Table (1.2.4.2.2)

Based on estimated marginal means

*. The mean difference is significant at the .05 level.

b. Adjustment for multiple comparisons: Least Significant Difference (equivalent to no adjustments).

Univariate Tests

Dependent Variable: Attitude

	Sum of Squares	df	Mean Square	F	Sig.	Partial Eta Squared
Contrast	73.877	1	73.877	166.110	.000	.071
Error	973.547	2189	.445			

Table (1.2.4.2.3)

The F tests the effect of Method, this test is based on the linearly independent pairwise comparisons among the estimated marginal means.

1.2.4.3. Gender Composition

Estimates

Dependent Variable: Attitude

Gender Composition	Mean	Std. Error	95% Confidence Interval Lower Bound	Upper Bound
Single-Gender	4.270	.020	4.231	4.310
Mix-Gender	4.102	.020	4.062	4.141

Table (1.2.4.3.1) Estimates for the gender composition with the attitude of the students as the dependent variable

Pairwise Comparisons

Dependent Variable: Attitude

(I) Gender Composition	(J) Gender Composition	Mean Difference (I-J)	Std. Error	Sig.[b]	95% Confidence Interval for Difference[b]	
					Lower Bound	Upper Bound
Single-Gender	Mix-Gender	.169*	.028	.000	.113	.224
Mix-Gender	Single-Gender	-.169*	.028	.000	-.224	-.113

Table (1.2.4.3.2)

Based on estimated marginal means

*. The mean difference is significant at the .05 level.

b. Adjustment for multiple comparisons: Least Significant Difference (equivalent to no adjustments).

Univariate Tests

Dependent Variable: Attitude

	Sum of Squares	df	Mean Square	F	Sig.	Partial Eta Squared
Contrast	15.567	1	15.567	35.003	.000	.016
Error	973.547	2189	.445			

Table (1.2.4.3.3)

The F tests the effect of Gender Composition. This test is based on the linearly independent pairwise comparisons among the estimated marginal means.

1.2.4.4 Gender * Method

Estimates

Dependent Variable: Attitude

Gender	Method	Mean	Std. Error	95% Confidence Interval	
				Lower Bound	Upper Bound
Women	TL	4.107	.029	4.049	4.164
	CL	4.159	.028	4.104	4.214
Men	TL	3.898	.029	3.841	3.955
	CL	4.580	.028	4.526	4.635

Table (1.2.4.4.1) Estimates for the gender*method with the attitude of the students as the dependent variable

1.2.4.5 Gender * Gender Composition

Estimates

Dependent Variable: Attitude

Gender	Gender Composition	Mean	Std. Error	95% Confidence Interval Lower Bound	95% Confidence Interval Upper Bound
Women	Single-Gender	4.391	.028	4.335	4.447
	Mix-Gender	3.874	.029	3.818	3.931
Men	Single-Gender	4.149	.028	4.094	4.205
	Mix-Gender	4.329	.028	4.273	4.385

Table (1.2.4.5.1) Estimates for the gender*gender composition with the attitude of the students as the dependent variable

1.2.4.6 Method * Gender Composition

Estimates

Dependent Variable: Attitude

Method	Gender Composition	Mean	Std. Error	95% Confidence Interval Lower Bound	95% Confidence Interval Upper Bound
TL	Single-Gender	3.942	.029	3.886	3.998
	Mix-Gender	4.063	.029	4.005	4.121
CL	Single-Gender	4.599	.028	4.544	4.654
	Mix-Gender	4.141	.028	4.086	4.195

Table (1.2.4.6.1) Estimates for the method*gender composition with the attitude of the students as the dependent variable

1.2.4.7 Gender * Method * Gender Composition

Estimates

Dependent Variable: Attitude

Gender	Method	Gender Composition	Mean	Std. Error	95% Confidence Interval Lower Bound	95% Confidence Interval Upper Bound
Women	TL	Single-Gender	4.082	.041	4.003	4.162
		Mix-Gender	4.131	.042	4.049	4.213
	CL	Single-Gender	4.700	.040	4.622	4.779
		Mix-Gender	3.617	.039	3.540	3.694
Men	TL	Single-Gender	3.802	.041	3.722	3.882
		Mix-Gender	3.995	.041	3.914	4.076
	CL	Single-Gender	4.497	.039	4.420	4.574
		Mix-Gender	4.664	.039	4.587	4.741

Table (1.2.4.7.1) Estimates for the gender*method*gender composition with the attitude of the students as the dependent variable

Pairwise Comparisons

Dependent Variable: Attitude

Gender	Gender Composition	(I) Method	(J) Method	Mean Difference (I-J)	Std. Error	Sig.[b]	95% Confidence Interval for Difference[b] Lower Bound	95% Confidence Interval for Difference[b] Upper Bound
Women	Single-Gender	TL	CL	-.618*	.057	.000	-.730	-.506
		CL	TL	.618*	.057	.000	.506	.730
	Mix-Gender	TL	CL	.514*	.057	.000	.401	.626
		CL	TL	-.514*	.057	.000	-.626	-.401
Men	Single-Gender	TL	CL	-.695*	.056	.000	-.806	-.584
		CL	TL	.695*	.056	.000	.584	.806
	Mix-Gender	TL	CL	-.669*	.057	.000	-.781	-.558
		CL	TL	.669*	.057	.000	.558	.781

Table (1.2.4.7.2)

Based on estimated marginal means

*. The mean difference is significant at the .05 level.

b. Adjustment for multiple comparisons: Least Significant Difference (equivalent to no adjustments).

1.2.5. Profile Plots

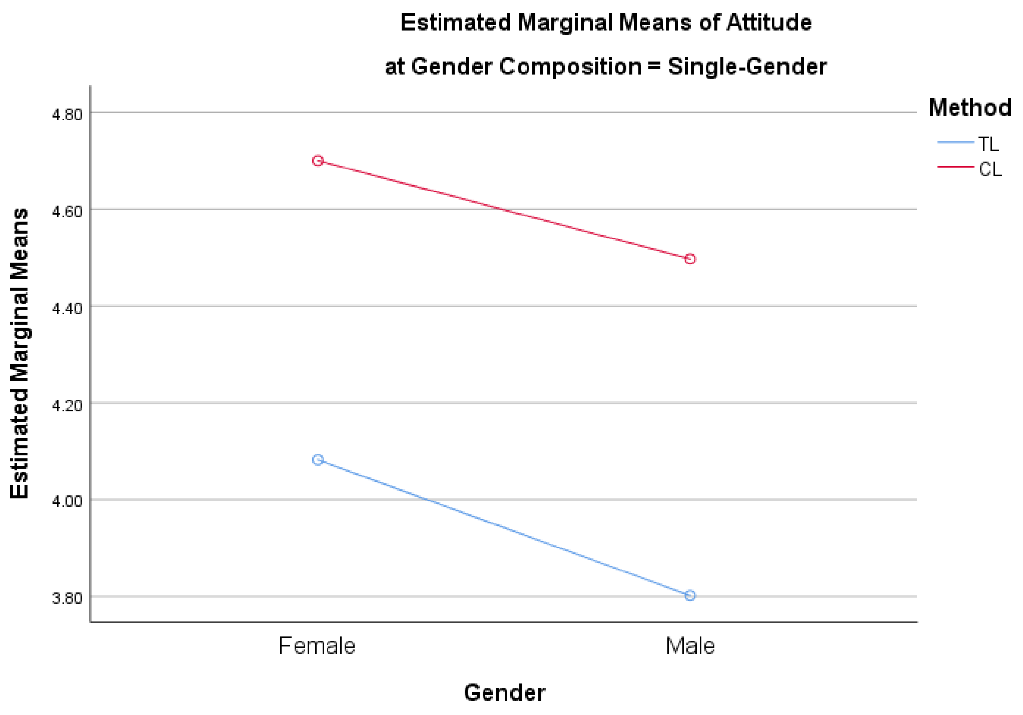

Figure (1.2.5.1)

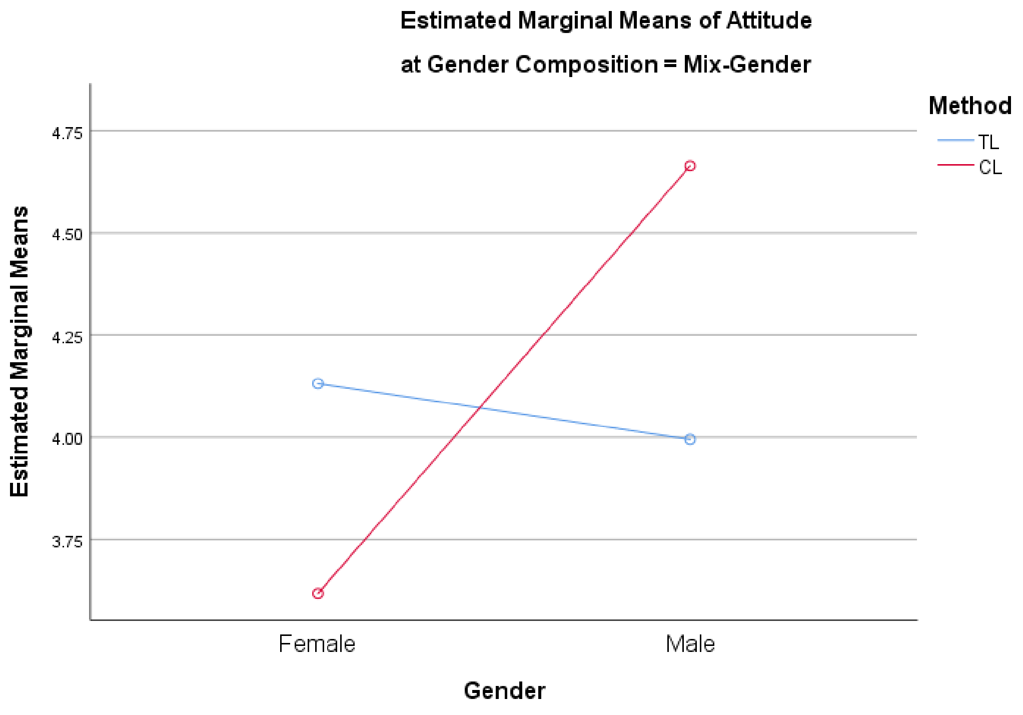

Figure (1.2.5.2)

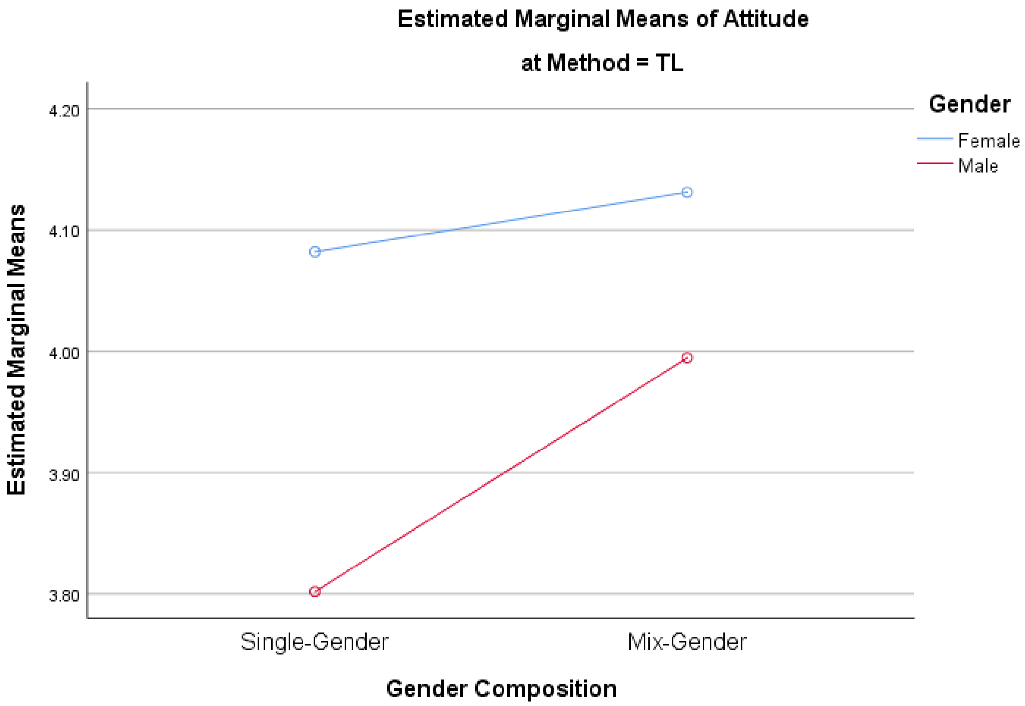

Figure (1.2.5.3)

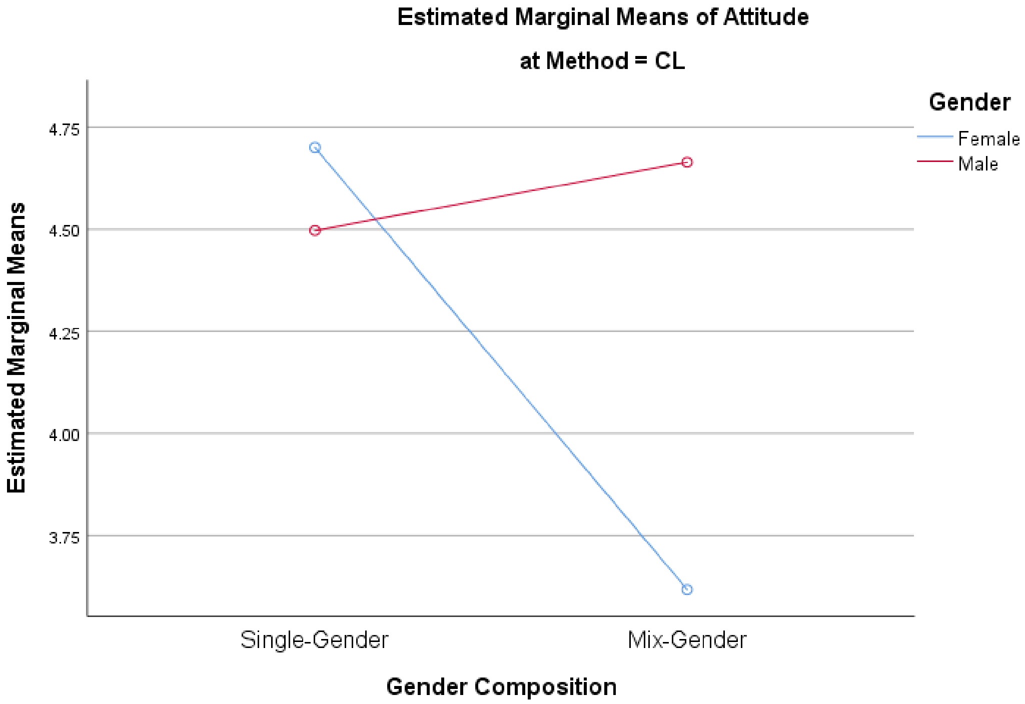

Figure (1.2.5.4)

Two-Way ANOVA

Non-Major Biology

Single Gender Students

Univariate Analysis of Variance

Between-Subjects Factors

		Value Label	N
Gender	1.00	Women	548
	2.00	Men	558
Method	1.00	TL	539
	2.00	CL	567

Table (2.1.1.1) Between-Subjects Factors including gender and method

Descriptive Statistics

Dependent Variable: Attitude_9 Factors

Gender	Method	Mean	Std. Deviation	N
Women	TL	3.7002	.64419	269
	CL	4.1053	.64930	279
	Total	3.9064	.67726	548
Men	TL	2.5393	.60263	270
	CL	3.2847	.59377	288
	Total	2.9240	.70433	558
Total	TL	3.1186	.85197	539
	CL	3.6885	.74461	567
	Total	3.4108	.84770	1106

Table (2.1. 1.2) The table provides the mean and standard deviation for each combination of the groups of the independent variables. In addition, the table provides "Total" rows, which allows means and standard deviations for groups only.

Tests of Between-Subjects Effects

Dependent Variable: Attitude_9 Factors

Source	Type III Sum of Squares	df	Mean Square	F	Sig.	Partial Eta Squared
Corrected Model	366.762[a]	3	122.254	315.296	.000	.462
Intercept	12830.972	1	12830.972	33091.436	.000	.968
Gender	271.198	1	271.198	699.428	.000	.388
Method	91.443	1	91.443	235.835	.000	.176
Gender * Method	8.000	1	8.000	20.632	.000	.018
Error	427.293	1102	.388			
Total	13660.635	1106				
Corrected Total	794.055	1105				

Table (2.1. 1.3) Tests where gender and method have been analysed to find the Type III sum of squares, df, mean square, F value, significance and partial eta squared where R Squared = .462 (Adjusted R Squared = .460)

2.1. 1.4 Estimated Marginal Means

2.1. 1.4.1 Gender * Method

Estimates

Dependent Variable: Attitude_9 Factors

				97.5% Confidence Interval	
Gender	Method	Mean	Std. Error	Lower Bound	Upper Bound
Women	TL	3.700	.038	3.615	3.785
	CL	4.105	.037	4.022	4.189
Men	TL	2.539	.038	2.454	2.624
	CL	3.285	.037	3.202	3.367

Table (2.1. 1.4.1.1) Estimates of women and men with the attitude as the dependent variable

Pairwise Comparisons

Dependent Variable: Attitude_9 Factors

Gender	(I) Method	(J) Method	Mean Difference (I-J)	Std. Error	Sig.[b]	97.5% Confidence Interval for Difference[b] Lower Bound	Upper Bound
Women	TL	CL	-.405*	.053	.000	-.525	-.286
	CL	TL	.405*	.053	.000	.286	.525
Men	TL	CL	-.745*	.053	.000	-.864	-.627
	CL	TL	.745*	.053	.000	.627	.864

Table (2.1.1.4.1.2)

Based on estimated marginal means

*. The mean difference is significant at the .025 level.

b. Adjustment for multiple comparisons: Least Significant Difference (equivalent to no adjustments).

Univariate Tests

Dependent Variable: Attitude_9 Factors

Gender		Sum of Squares	df	Mean Square	F	Sig.	Partial Eta Squared
Women	Contrast	22.479	1	22.479	57.975	.000	.050
	Error	427.293	1102	.388			
Men	Contrast	77.442	1	77.442	199.724	.000	.153
	Error	427.293	1102	.388			

Table (2.1.1.4.1.3) Each F tests the simple effects of Method within each level combination of the other effects shown. These tests are based on the linearly independent pairwise comparisons among the estimated marginal means.

2.2.1.5. Profile Plots

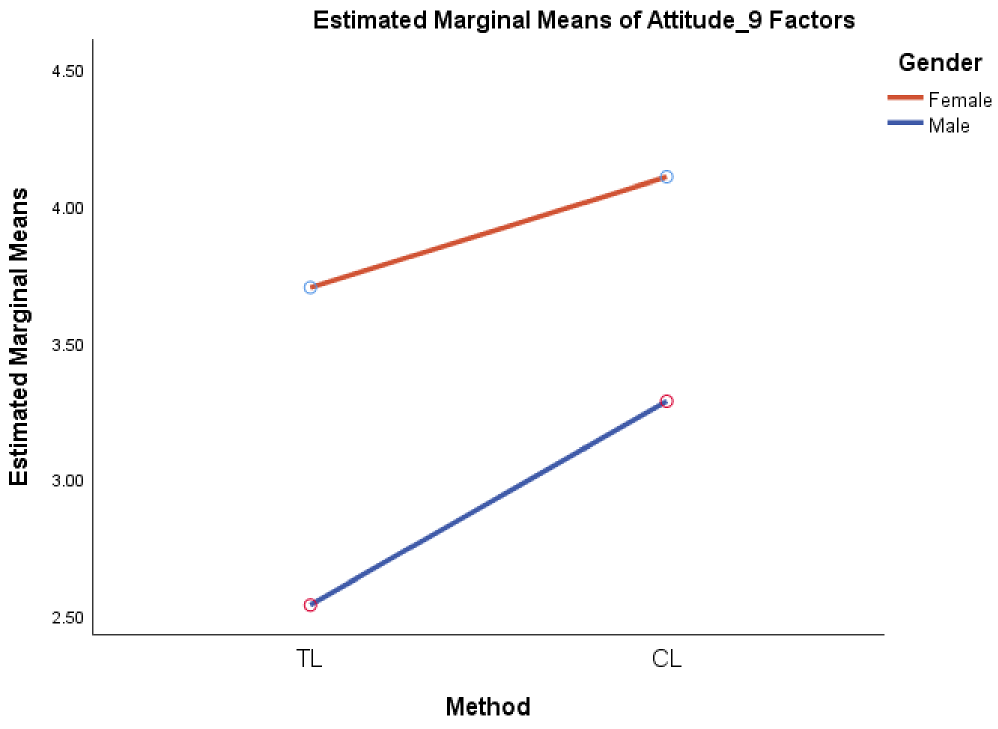

Figure (2.2. 1.5.1)

Mixed Gender Students

Univariate Analysis of Variance

Between-Subjects Factors

		Value Label	N
Gender	1.00	Women	639
	2.00	Men	630
Method	1.00	TL	621
	2.00	CL	648

Table (2.2.2.1.1) Between-Subjects Factors including Gender and Method

Descriptive Statistics

Dependent Variable: Attitude_9 Factors

Gender	Method	Mean	Std. Deviation	N
Women	TL	3.0594	.55055	315
	CL	2.5969	.65646	324
	Total	2.8249	.64877	639
Men	TL	2.6899	.60389	306
	CL	3.7489	.55266	324
	Total	3.2346	.78375	630
Total	TL	2.8774	.60588	621
	CL	3.1729	.83660	648
	Total	3.0283	.74730	1269

Table (2.2.2.2) The table provides the mean and standard deviation for each combination of the groups of the independent variables. In addition, the table provides "Total" rows, which allows means and standard deviations for groups only.

Tests of Between-Subjects Effects

Dependent Variable: Attitude_9 Factors

Source	Type III Sum of Squares	df	Mean Square	F	Sig.	Partial Eta Squared
Corrected Model	366.762[a]	3	122.254	315.296	.000	.462
Intercept	12830.972	1	12830.972	33091.436	.000	.968
Gender	271.198	1	271.198	699.428	.000	.388
Method	91.443	1	91.443	235.835	.000	.176
Gender * Method	8.000	1	8.000	20.632	.000	.018
Error	427.293	1102	.388			
Total	13660.635	1106				
Corrected Total	794.055	1105				

Table (2.2.2.3.) Tests where gender and method have been analysed to find the Type III sum of squares, df, mean square, F value, significance and partial eta squared where R Squared = .462 (Adjusted R Squared = .460)

2.2.2.4 Estimated Marginal Means

2.2.2.4.1 Gender * Method

Estimates

Dependent Variable: Attitude_9 Factors

Gender	Method	Mean	Std. Error	95% Confidence Interval Lower Bound	Upper Bound
Women	TL	3.059	.033	2.994	3.125
	CL	2.597	.033	2.532	2.662
Men	TL	2.690	.034	2.623	2.756
	CL	3.749	.033	3.684	3.814

Table (2.2.2.4.1.1) Estimates of women and men with the attitude as the dependent variable

Pairwise Comparisons

Dependent Variable: Attitude_9 Factors

Gender	(I) Method	(J) Method	Mean Difference (I-J)	Std. Error	Sig.[b]	95% Confidence Interval for Difference[b]	
						Lower Bound	Upper Bound
Women	TL	CL	.462*	.047	.000	.370	.554
	CL	TL	-.462*	.047	.000	-.554	-.370
Men	TL	CL	-1.059*	.047	.000	-1.152	-.966
	CL	TL	1.059*	.047	.000	.966	1.152

Table (2.2.2.4.1.2)

Based on estimated marginal means

*. The mean difference is significant at the .05 level.

b. Adjustment for multiple comparisons: Least Significant Difference (equivalent to no adjustments).

Univariate Tests

Dependent Variable: Attitude_9 Factors

Gender		Sum of Squares	df	Mean Square	F	Sig.	Partial Eta Squared	Noncent. Parameter	Observed Power[a]
Women	Contrast	34.162	1	34.162	97.276	.000	.071	97.276	1.000
	Error	444.254	1265	.351					
Men	Contrast	176.484	1	176.484	502.533	.000	.284	502.533	1.000
	Error	444.254	1265	.351					

Table (2.2.2.4.1.3)

Each F tests the simple effects of Method within each level combination of the other effects shown. These tests are based on the linearly independent pairwise comparisons among the estimated marginal means.

a. Computed using alpha = .05

Tests of Between-Subjects Effects

Dependent Variable: Attitude_9 Factors

Source	Type III Sum of Squares	df	Mean Square	F	Sig.	Partial Eta Squared	Noncent. Parameter	Observed Power[b]
Corrected Model	263.876[a]	3	87.959	250.460	.000	.373	751.380	1.000
Intercept	11596.488	1	11596.488	33020.646	.000	.963	33020.646	1.000
Gender	48.534	1	48.534	138.199	.000	.098	138.199	1.000
Method	28.205	1	28.205	80.312	.000	.060	80.312	1.000
Gender * Method	183.495	1	183.495	522.497	.000	.292	522.497	1.000
Error	444.254	1265	.351					
Total	12345.546	1269						
Corrected Total	708.130	1268						

Table (2.2.2.4.1.4) Tests where gender and method have been analysed to find the Type III sum of squares, df, mean square, F value, significance and partial eta squared where R Squared = .462 (Adjusted R Squared = .460)

2.2.2.5. Profile Plots

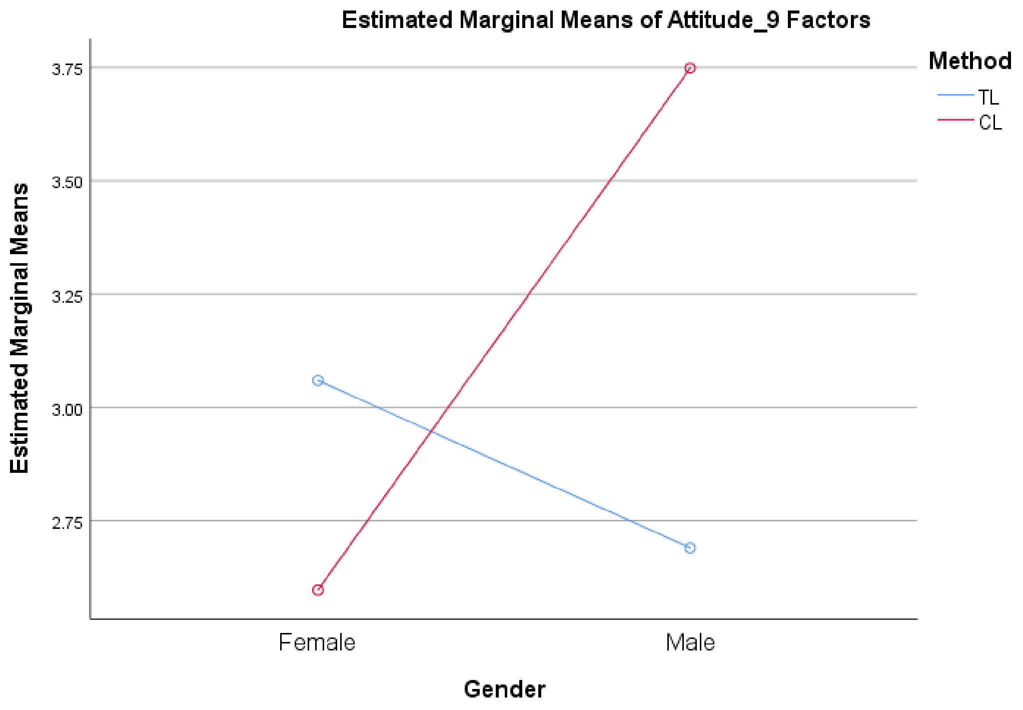

Figure (2.2.2.5.1)

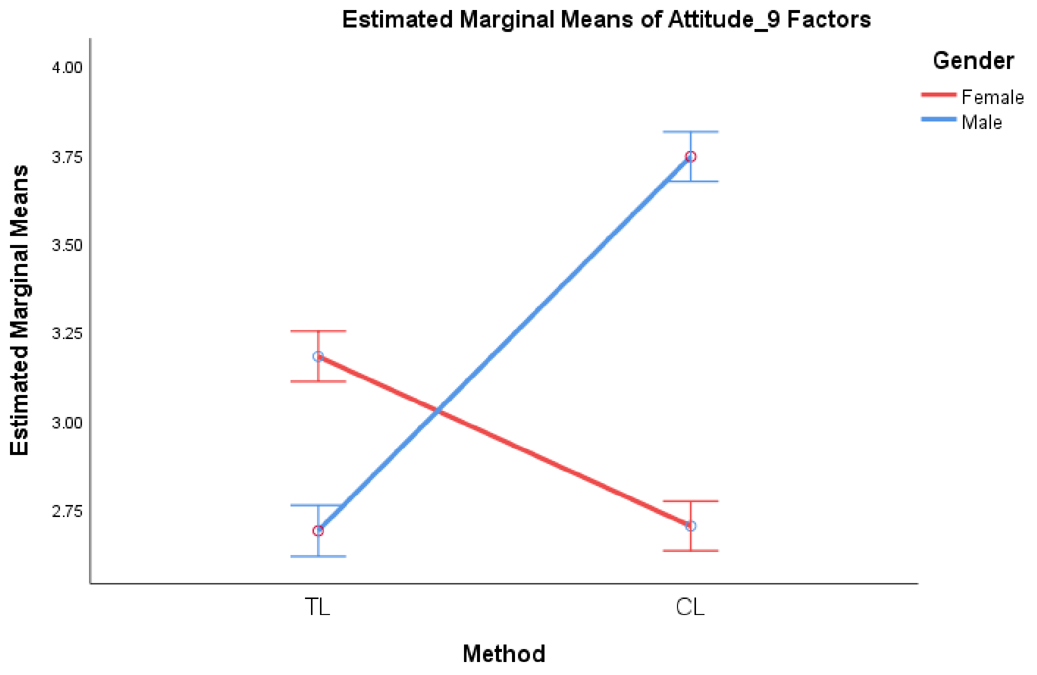

Figure (2.2.2.5.2)

Major Biology
Single Gender Students

Univariate Analysis of Variance

Between-Subjects Factors

		Value Label	N
Method	1.00	TL	540
	2.00	CL	566
Gender	1.00	Women	548
	2.00	Men	558

Table (2.3.1.1) Between-Subjects Factors including Gender and Method

Descriptive Statistics

Dependent Variable: Attitude_9 Factors

Method	Gender	Mean	Std. Deviation	N
TL	Women	4.0821	.64667	270
	Men	3.8020	.75679	270
	Total	3.9420	.71707	540
CL	Women	4.7001	.53671	278
	Men	4.4970	.62199	288
	Total	4.5967	.58997	566
Total	Women	4.3956	.66869	548
	Men	4.1607	.77252	558
	Total	4.2771	.73211	1106

Table (2.3.1.2) The table provides the mean and standard deviation for each combination of the groups of the independent variables. In addition, the table provides "Total" rows, which allows means and standard deviations for groups only.

Tests of Between-Subjects Effects

Dependent Variable: Attitude_9 Factors

Source	Type III Sum of Squares	df	Mean Square	F	Sig.	Partial Eta Squared	Noncent. Parameter	Observed Power[b]
Corrected Model	134.879[a]	3	44.960	108.325	.000	.228	324.975	1.000
Intercept	20154.026	1	20154.026	48558.755	.000	.978	48558.755	1.000
Method	119.081	1	119.081	286.912	.000	.207	286.912	1.000
Gender	16.131	1	16.131	38.865	.000	.034	38.865	1.000
Method * Gender	.410	1	.410	.988	.320	.001	.988	.168
Error	457.379	1102	.415					
Total	20824.769	1106						
Corrected Total	592.258	1105						

Table (2.3.1.3.) Tests where gender and method have been analysed to find the Type III sum of squares, df, mean square, F value, significance and partial eta squared where R Squared = .462 (Adjusted R Squared = .460)

2.3.1.4 Estimated Marginal Means

2.3.1.4.1 Gender and Method

Univariate Tests

Dependent Variable: Attitude_9 Factors

Gender		Sum of Squares	df	Mean Square	F	Sig.	Partial Eta Squared	Noncent. Parameter	Observed Power[a]
Women	Contrast	52.306	1	52.306	126.025	.000	.103	126.025	1.000
	Error	457.379	1102	.415					
Men	Contrast	67.315	1	67.315	162.188	.000	.128	162.188	1.000
	Error	457.379	1102	.415					

Table (2.3.1.4.1.1) Each F tests the simple effects of gender within each level combination of the other effects shown. These tests are based on the linearly independent pairwise comparisons among the estimated marginal means.

a. Computed using alpha = .05

Estimates

Dependent Variable: Attitude_9 Factors

Method	Gender	Mean	Std. Error	95% Confidence Interval Lower Bound	Upper Bound
TL	Women	4.082	.039	4.005	4.159
	Men	3.802	.039	3.725	3.879
CL	Women	4.700	.039	4.624	4.776
	Men	4.497	.038	4.422	4.571

Table (2.3.1.4.1.2) Estimates of method effect on women and men with the attitude as the dependent variable based on the teaching method

Pairwise Comparisons

Dependent Variable: Attitude_9 Factors

Gender	(I) Method	(J) Method	Mean Difference (I-J)	Std. Error	Sig.[b]	95% Confidence Interval for Difference[b] Lower Bound	Upper Bound
Women	TL	CL	-.618*	.055	.000	-.726	-.510
	CL	TL	.618*	.055	.000	.510	.726
Men	TL	CL	-.695*	.055	.000	-.802	-.588
	CL	TL	.695*	.055	.000	.588	.802

Table (2.3.1.4.1.3)

Based on estimated marginal means

*. The mean difference is significant at the .05 level.

b. Adjustment for multiple comparisons: Least Significant Difference (equivalent to no adjustments).

2.3.1.4.2 Method

Estimates

Dependent Variable: Attitude_9 Factors

Method	Mean	Std. Error	95% Confidence Interval Lower Bound	Upper Bound
TL	3.942	.028	3.888	3.996
CL	4.599	.027	4.545	4.652

Table (2.3.1.4.2.1) Estimates of the teaching method with the attitude as the dependent variable based on the teaching method

Pairwise Comparisons

Dependent Variable: Attitude_9 Factors

(I) Method	(J) Method	Mean Difference (I-J)	Std. Error	Sig.[b]	95% Confidence Interval for Difference[b] Lower Bound	Upper Bound
TL	CL	-.656*	.039	.000	-.733	-.580
CL	TL	.656*	.039	.000	.580	.733

Table (2.3.1.4.2.2)

Based on estimated marginal means

*. The mean difference is significant at the .05 level.

b. Adjustment for multiple comparisons: Bonferroni.

Univariate Tests

Dependent Variable: Attitude_9 Factors

	Sum of Squares	Df	Mean Square	F	Sig.	Partial Eta Squared	Noncent. Parameter	Observed Power[a]
Contrast	119.081	1	119.081	286.912	.000	.207	286.912	1.000
Error	457.379	1102	.415					

Table (2.3.1.4.2.3) The F tests the effect of Method. This test is based on the linearly independent pairwise comparisons among the estimated marginal means.

a. Computed using alpha = .05

Tests of Between-Subjects Effects

Dependent Variable: Attitude_9 Factors

Source	Type III Sum of Squares	df	Mean Square	F	Sig.	Partial Eta Squared
Corrected Model	366.762[a]	3	122.254	315.296	.000	.462
Intercept	12830.972	1	12830.972	33091.436	.000	.968
Gender	271.198	1	271.198	699.428	.000	.388
Method	91.443	1	91.443	235.835	.000	.176
Gender * Method	8.000	1	8.000	20.632	.000	.018
Error	427.293	1102	.388			
Total	13660.635	1106				
Corrected Total	794.055	1105				

Table (2.3.1.4.2.4) Tests where gender and method have been analysed to find the Type III sum of squares, df, mean square, F value, significance and partial eta squared to understand the influence of method on students attitude where R Squared = .462 (Adjusted R Squared = .460)

2.3.1.5. Profile Plots

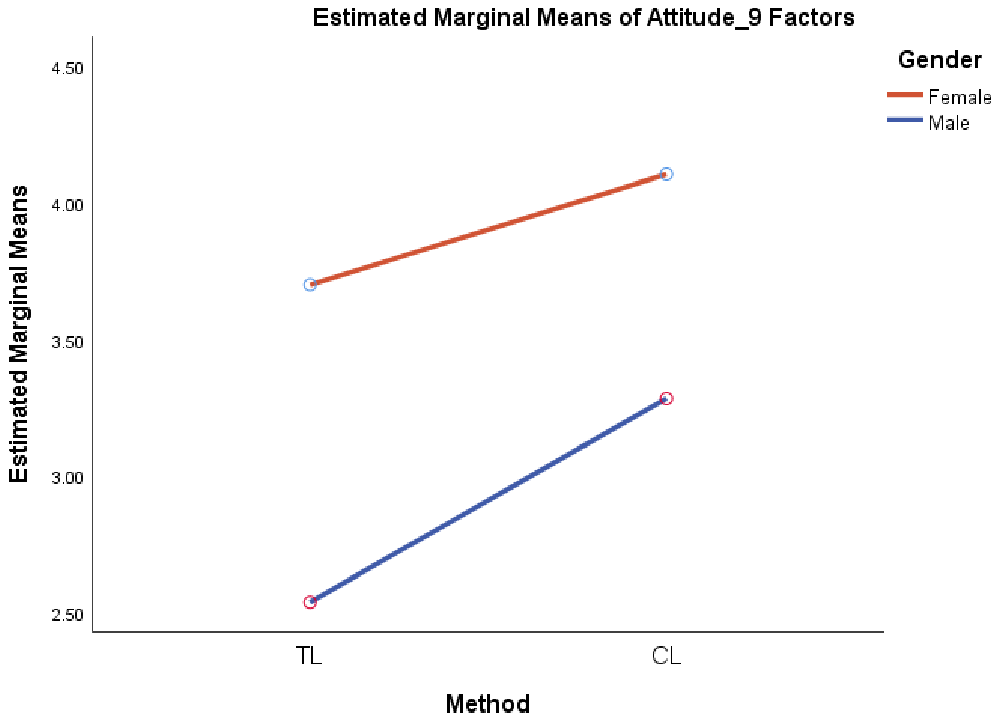

Figure (2.3.1.5.1)

Mixed Gender Students

Univariate Analysis of Variance

Between-Subjects Factors

		Value Label	N
Gender	1.00	Women	541
	2.00	Men	550
Method	1.00	TL	513
	2.00	CL	578

Table (2.3.2.1) Between-Subjects Factors including Gender and Method

Descriptive Statistics

Dependent Variable: Attitude_9 Factors

Gender	Method	Mean	Std. Deviation	N
Women	TL	4.1311	.65403	252
	CL	3.6174	.80113	289
	Total	3.8567	.77904	541
Men	TL	3.9946	.64565	261
	CL	4.6639	.63349	289
	Total	4.3463	.72102	550
Total	TL	4.0616	.65273	513
	CL	4.1407	.89160	578
	Total	4.1035	.78899	1091

Table (2.3.2.2) The table provides the mean and standard deviation for each combination of the groups of the independent variables. In addition, the table provides "Total" rows, which allows means and standard deviations for groups only.

Tests of Between-Subjects Effects

Dependent Variable: Attitude_9 Factors

Source	Type III Sum of Squares	df	Mean Square	F	Sig.	Partial Eta Squared
Corrected Model	162.361[a]	3	54.120	113.972	.000	.239
Intercept	18287.111	1	18287.111	38510.863	.000	.973
Gender	56.264	1	56.264	118.487	.000	.098
Method	1.647	1	1.647	3.467	.063	.003
Gender * Method	95.087	1	95.087	200.245	.000	.156
Error	516.168	1087	.475			
Total	19049.494	1091				
Corrected Total	678.529	1090				

Table (2.3.2.3.) Tests where gender and method have been analysed to find the Type III sum of squares, df, mean square, F value, significance and partial eta squared where R Squared = .462 (Adjusted R Squared = .460)

2.3.2.4 Estimated Marginal Means

2.3.2.4.1 Gender

Estimates

Dependent Variable: Attitude_9 Factors

Gender	Mean	Std. Error	95% Confidence Interval	
			Lower Bound	Upper Bound
Women	3.874	.030	3.816	3.932
Men	4.329	.029	4.272	4.387

Table (2.3.2.4.1.1) Estimates of the gender with the attitude as the dependent variable.

Pairwise Comparisons

Dependent Variable: Attitude_9 Factors

(I) Gender	(J) Gender	Mean Difference (I-J)	Std. Error	Sig.[b]	95% Confidence Interval for Difference[b]	
					Lower Bound	Upper Bound
Women	Men	-.455*	.042	.000	-.537	-.373
Men	Women	.455*	.042	.000	.373	.537

Table (2.3.2.4.1.2)

Based on estimated marginal means

*. The mean difference is significant at the .05 level.

b. Adjustment for multiple comparisons: Least Significant Difference (equivalent to no adjustments).

Univariate Tests

Dependent Variable: Attitude_9 Factors

	Sum of Squares	df	Mean Square	F	Sig.	Partial Eta Squared
Contrast	56.264	1	56.264	118.487	.000	.098
Error	516.168	1087	.475			

Table (2.3.2.4.1.3) The F tests the effect of Gender. This test is based on the linearly independent pairwise comparisons among the estimated marginal means.

2.3.2.4.2 Method

Estimates

Dependent Variable: Attitude_9 Factors

Method	Mean	Std. Error	95% Confidence Interval	
			Lower Bound	Upper Bound
TL	4.063	.030	4.003	4.123
CL	4.141	.029	4.084	4.197

Table (2.3.2.4.2.1) Estimates of the teaching methods with the attitude as the dependent variable.

Pairwise Comparisons

Dependent Variable: Attitude_9 Factors

(I) Method	(J) Method	Mean Difference (I-J)	Std. Error	Sig.[a]	95% Confidence Interval for Difference[a] Lower Bound	Upper Bound
TL	CL	-.078	.042	.063	-.160	.004
CL	TL	.078	.042	.063	-.004	.160

Table (2.3.2.4.2.2)

Based on estimated marginal means

a. Adjustment for multiple comparisons: Least Significant Difference (equivalent to no adjustments).

Univariate Tests

Dependent Variable: Attitude_9 Factors

	Sum of Squares	df	Mean Square	F	Sig.	Partial Eta Squared
Contrast	1.647	1	1.647	3.467	.063	.003
Error	516.168	1087	.475			

Table (2.3.2.4.2.3) The F tests the effect of Method. This test is based on the linearly independent pairwise comparisons among the estimated marginal means.

2.3.2.4.3. Gender * Method

Estimates

Dependent Variable: Attitude_9 Factors

Gender	Method	Mean	Std. Error	95% Confidence Interval Lower Bound	Upper Bound
Women	TL	4.131	.043	4.046	4.216
	CL	3.617	.041	3.538	3.697
Men	TL	3.995	.043	3.911	4.078
	CL	4.664	.041	4.584	4.743

Table (2.3.2.4.3.1) Estimates of the gender with the teaching methods with the attitude as the dependent variable.

Pairwise Comparisons

Dependent Variable: Attitude_9 Factors

Gender	(I) Method	(J) Method	Mean Difference (I-J)	Std. Error	Sig.[b]	95% Confidence Interval for Difference[b] Lower Bound	Upper Bound
Women	TL	CL	.514*	.059	.000	.397	.630
	CL	TL	-.514*	.059	.000	-.630	-.397
Men	TL	CL	-.669*	.059	.000	-.785	-.554
	CL	TL	.669*	.059	.000	.554	.785

Table (2.3.2.4.3.2)

Based on estimated marginal means

*. The mean difference is significant at the .05 level.

b. Adjustment for multiple comparisons: Least Significant Difference (equivalent to no adjustments).

Univariate Tests

Dependent Variable: Attitude_9 Factors

Gender		Sum of Squares	df	Mean Square	F	Sig.	Partial Eta Squared
Women	Contrast	35.524	1	35.524	74.810	.000	.064
	Error	516.168	1087	.475			
Men	Contrast	61.451	1	61.451	129.409	.000	.106
	Error	516.168	1087	.475			

Table (2.3.2.4.3.3) Each F tests the simple effects of Method within each level combination of the other effects shown. These tests are based on the linearly independent pairwise comparisons among the estimated marginal means.

2.3.2.5. Profile Plots

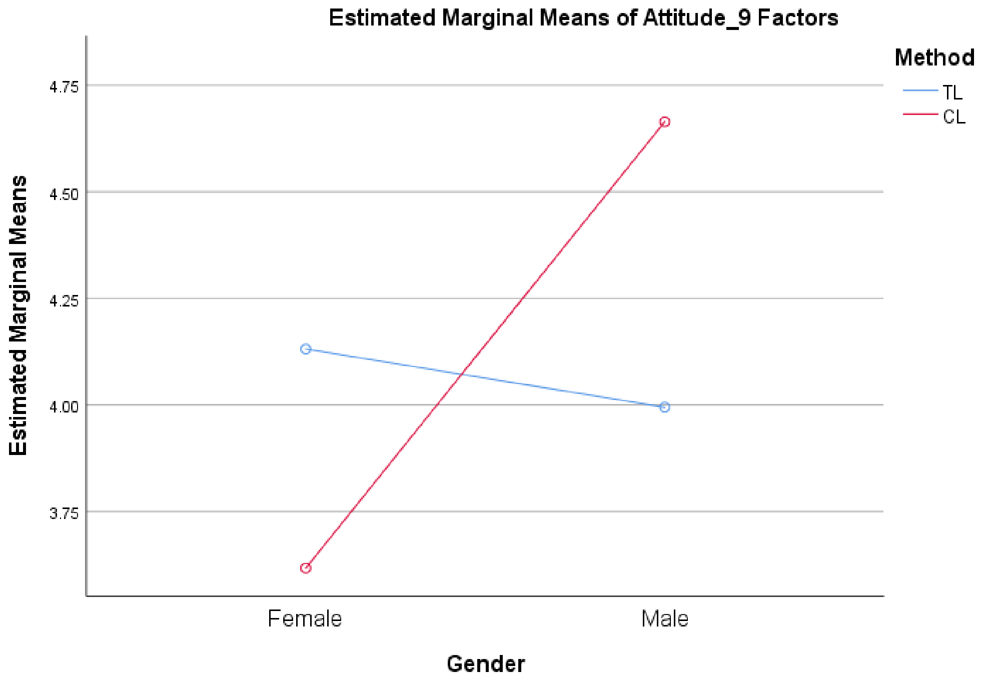

Figure (2.3.2.5.1)

Attitude Factors Analysis (Each Factor)
Non-Major Biology
Single Gender Students
Factor 1 Feelings towards Biology
Traditional Learning

Case Processing Summary

		N	%
Cases	Valid	30	100.0
	Excluded[a]	0	.0
	Total	30	100.0

Table (3.1.1.1.1) Listwise deletion based on all variables in the procedure.

Women

		N	%
Cases	Valid	30	100.0
	Excluded[a]	0	.0
	Total	30	100.0

Men
Table (3.1.1.1.1) Listwise deletion based on all variables in the procedure.

Reliability Statistics

Cronbach's Alpha	Cronbach's Alpha Based on Standardized Items	N of Items
.809	.810	14

Women

Cronbach's Alpha	Cronbach's Alpha Based on Standardized Items	N of Items
.885	.883	14

Men
Table (3.1.1.1.2) The Cronbach's alpha for the factor is calculated based on the number of items.

Item Statistics

	Mean	Std. Deviation	N
Biology is very interesting to me.	2.93	1.015	30
I have always enjoyed studying biology in school.	3.30	.877	30
I am always under a terrible strain in a biology class.	2.80	.961	30
I feel a definite positive reaction to biology; it's enjoyable.	3.00	.910	30
Biology makes me feel secure, and at the same time it is stimulating.	3.23	.898	30
I feel at ease in biology and like it very much.	3.10	.923	30
In general, I have a good feeling toward biology.	3.13	1.042	30
I really like biology.	3.03	.964	30
Biology is fascinating and fun.	2.97	1.033	30
When I hear the word biology, I have a feeling of dislike.	3.07	.828	30
I approach biology with a feeling of hesitation.	3.00	1.050	30
It makes me nervous to even think about doing a biology experiment.	3.03	.850	30
Biology makes me feel uncomfortable, restless, irritable, and impatient.	2.93	1.112	30
I don't like biology, and it scares me to have to take it.	3.23	.858	30

Women

	Mean	Std. Deviation	N
Biology is very interesting to me.	2.53	.776	30
I have always enjoyed studying biology in school.	2.53	.776	30
I am always under a terrible strain in a biology class.	2.60	.855	30
I feel a definite positive reaction to biology; it's enjoyable.	2.47	.681	30
Biology makes me feel secure, and at the same time it is stimulating.	2.70	.794	30
I feel at ease in biology and like it very much.	2.43	.898	30
In general, I have a good feeling toward biology.	2.53	.776	30
I really like biology.	2.47	.819	30
Biology is fascinating and fun.	2.67	.844	30
When I hear the word biology, I have a feeling of dislike.	2.57	.858	30
I approach biology with a feeling of hesitation.	2.57	.817	30
It makes me nervous to even think about doing a biology experiment.	2.57	.774	30
Biology makes me feel uncomfortable, restless, irritable, and impatient.	2.47	.937	30
I don't like biology, and it scares me to have to take it.	2.47	.900	30

Men

Table (3.1.1.1.3) The statistics for the mean and standard deviation for each item in the factor in the questionnaire.

Item-Total Statistics

	Scale Mean if Item Deleted	Scale Variance if Item Deleted	Corrected Item-Total Correlation	Squared Multiple Correlation	Cronbach's Alpha if Item Deleted
Biology is very interesting to me.	39.83	43.730	.486	.623	.792
I have always enjoyed studying biology in school.	39.47	43.568	.601	.618	.785
I am always under a terrible strain in a biology class.	39.97	44.723	.439	.571	.796
I feel a definite positive reaction to biology; it's enjoyable.	39.77	43.840	.550	.649	.788
Biology makes me feel secure, and at the same time it is stimulating.	39.53	47.637	.230	.645	.811
I feel at ease in biology and like it very much.	39.67	43.540	.566	.612	.787
In general, I have a good feeling toward biology.	39.63	43.137	.516	.611	.790
I really like biology.	39.73	45.168	.400	.305	.799
Biology is fascinating and fun.	39.80	46.234	.284	.284	.809
When I hear the word biology, I have a feeling of dislike.	39.70	46.148	.396	.524	.800
I approach biology with a feeling of hesitation.	39.77	46.047	.290	.554	.809
It makes me nervous to even think about doing a biology experiment.	39.73	48.547	.170	.198	.814
Biology makes me feel uncomfortable, restless, irritable, and impatient.	39.83	42.489	.522	.550	.789
I don't like biology, and it scares me to have to take it.	39.53	43.430	.630	.655	.783

Women

Item-Total Statistics

	Scale Mean if Item Deleted	Scale Variance if Item Deleted	Corrected Item-Total Correlation	Squared Multiple Correlation	Cronbach's Alpha if Item Deleted
Biology is very interesting to me.	33.03	49.413	.300	.518	.888
I have always enjoyed studying biology in school.	33.03	48.930	.346	.353	.886
I am always under a terrible strain in a biology class.	32.97	47.275	.449	.613	.882
I feel a definite positive reaction to biology; it's enjoyable.	33.10	48.990	.402	.619	.883
Biology makes me feel secure, and at the same time it is stimulating.	32.87	44.878	.731	.684	.869
I feel at ease in biology and like it very much.	33.13	44.120	.701	.740	.870
In general, I have a good feeling toward biology.	33.03	45.551	.681	.616	.871
I really like biology.	33.10	45.266	.667	.719	.872
Biology is fascinating and fun.	32.90	47.128	.470	.673	.881
When I hear the word biology, I have a feeling of dislike.	33.00	45.517	.607	.653	.874
I approach biology with a feeling of hesitation.	33.00	46.414	.557	.516	.877
It makes me nervous to even think about doing a biology experiment.	33.00	46.345	.602	.637	.875
Biology makes me feel uncomfortable, restless, irritable, and impatient.	33.10	44.231	.656	.684	.872
I don't like biology, and it scares me to have to take it.	33.10	44.921	.627	.672	.873

Men

Table (3.1.1.1.4) The total statistics for each item in the factor in the questionnaire.

Collaborative Learning

Case Processing Summary

		N	%
Cases	Valid	31	100.0
	Excluded[a]	0	.0
	Total	31	100.0

Women

		N	%
Cases	Valid	32	100.0
	Excluded[a]	0	.0
	Total	32	100.0

Men

Table (3.1.1.2.1) Listwise deletion based on all variables in the procedure.

Reliability Statistics

Cronbach's Alpha	Cronbach's Alpha Based on Standardized Items	N of Items
.823	.811	14

Women

Cronbach's Alpha	Cronbach's Alpha Based on Standardized Items	N of Items
.850	.833	14

Men

Table (3.1.1.2.2) The Cronbach's alpha for the feelings towards biology factor is calculated based on the number of items

Item Statistics

	Mean	Std. Deviation	N
Biology is very interesting to me.	3.74	.815	31
I have always enjoyed studying biology in school.	3.52	.769	31
I am always under a terrible strain in a biology class.	3.81	.910	31
I feel a definite positive reaction to biology; it's enjoyable.	3.74	.815	31
Biology makes me feel secure, and at the same time it is stimulating.	3.77	.805	31
I feel at ease in biology and like it very much.	3.71	.693	31
In general, I have a good feeling toward biology.	3.81	.873	31
I really like biology.	3.77	.845	31
Biology is fascinating and fun.	3.77	.617	31
When I hear the word biology, I have a feeling of dislike.	3.87	.619	31
I approach biology with a feeling of hesitation.	3.81	.792	31
It makes me nervous to even think about doing a biology experiment.	3.77	.805	31
Biology makes me feel uncomfortable, restless, irritable, and impatient.	3.81	.873	31

I don't like biology, and it scares me to have to take it.	3.81	.654	31

Women

	Mean	Std. Deviation	N
Biology is very interesting to me.	3.06	.435	32
I have always enjoyed studying biology in school.	3.03	.740	32
I am always under a terrible strain in a biology class.	2.94	.619	32
I feel a definite positive reaction to biology; it's enjoyable.	3.09	.777	32
Biology makes me feel secure, and at the same time it is stimulating.	3.09	.734	32
I feel at ease in biology and like it very much.	3.03	.647	32
In general, I have a good feeling toward biology.	3.00	.916	32
I really like biology.	3.28	.813	32
Biology is fascinating and fun.	2.84	.920	32
When I hear the word biology, I have a feeling of dislike.	3.22	.832	32
I approach biology with a feeling of hesitation.	3.19	.821	32
It makes me nervous to even think about doing a biology experiment.	3.16	.767	32
Biology makes me feel uncomfortable, restless, irritable, and impatient.	3.13	.833	32

I don't like biology, and it scares me to have to take it.	3.25	1.016	32

Men

Table (3.1.1.2.3) The statistics for the mean and standard deviation for each item in the factor in the questionnaire.

Item-Total Statistics

	Scale Mean if Item Deleted	Scale Variance if Item Deleted	Corrected Item-Total Correlation	Squared Multiple Correlation	Cronbach's Alpha if Item Deleted
Biology is very interesting to me.	48.97	30.099	.632	.588	.798
I have always enjoyed studying biology in school.	49.19	32.561	.371	.489	.818
I am always under a terrible strain in a biology class.	48.90	31.024	.450	.427	.813
I feel a definite positive reaction to biology; it's enjoyable.	48.97	29.699	.681	.621	.795
Biology makes me feel secure, and at the same time it is stimulating.	48.94	29.529	.713	.861	.792
I feel at ease in biology and like it very much.	49.00	34.667	.155	.448	.830
In general, I have a good feeling toward biology.	48.90	30.024	.589	.662	.801
I really like biology.	48.94	30.596	.546	.780	.805
Biology is fascinating and fun.	48.94	35.996	.005	.318	.837
When I hear the word biology, I have a feeling of dislike.	48.84	33.206	.396	.568	.816

I approach biology with a feeling of hesitation.	48.90	33.690	.228	.761	.828
It makes me nervous to even think about doing a biology experiment.	48.94	29.396	.730	.789	.791
Biology makes me feel uncomfortable, restless, irritable, and impatient.	48.90	30.290	.558	.621	.804
I don't like biology, and it scares me to have to take it.	48.90	34.757	.159	.349	.829

Women

Item-Total Statistics

	Scale Mean if Item Deleted	Scale Variance if Item Deleted	Corrected Item-Total Correlation	Squared Multiple Correlation	Cronbach's Alpha if Item Deleted
Biology is very interesting to me.	40.25	41.419	-.063	.550	.861
I have always enjoyed studying biology in school.	40.28	37.176	.391	.428	.846
I am always under a terrible strain in a biology class.	40.38	38.823	.266	.555	.851
I feel a definite positive reaction to biology; it's enjoyable.	40.22	37.467	.335	.656	.849
Biology makes me feel secure, and at the same time it is stimulating.	40.22	37.273	.384	.414	.846
I feel at ease in biology and like it very much.	40.28	37.370	.438	.487	.843
In general, I have a good feeling toward biology.	40.31	33.125	.692	.763	.826
I really like biology.	40.03	35.967	.475	.653	.841
Biology is fascinating and fun.	40.47	33.870	.611	.694	.832
When I hear the word biology, I have a feeling of dislike.	40.09	33.830	.695	.752	.827
I approach biology with a feeling of hesitation.	40.13	34.500	.631	.853	.831
It makes me nervous to even think about doing a biology experiment.	40.16	34.265	.713	.845	.827
Biology makes me feel uncomfortable, restless, irritable, and impatient.	40.19	35.835	.474	.817	.841
I don't like biology, and it scares me to have to take it.	40.06	32.835	.634	.593	.830

Men

Table (3.1.1.2.4) The total statistics for each item in the factor in the questionnaire.

TL vs CL

Group Statistics

	Method	N	Mean	Std. Deviation	Std. Error Mean
Feeling toward biology	TL	30	3.0660	.50232	.09171
	CL	31	3.7803	.42695	.07668

Women

	Method	N	Mean	Std. Deviation	Std. Error Mean
Feeling toward biology	TL	30	2.5520	.50456	.09212
	CL	32	3.1050	.44971	.07950

Men

Table (3.1.1.3.1) The group statistics for each item in the factors Questionnaire with method.

Independent Samples Test

		Levene's Test for Equality of Variances		t-test for Equality of Means						
									95% Confidence Interval of the Difference	
		F	Sig.	t	df	Sig. (2-tailed)	Mean Difference	Std. Error Difference	Lower	Upper
Feeling toward biology	Equal variances assumed	1.099	.299	-5.991	59	.000	-.71432	.11923	-.95289	-.47575
	Equal variances not assumed			-5.975	56.859	.000	-.71432	.11955	-.95372	-.47492

Women

Independent Samples Test

		Levene's Test for Equality of Variances		t-test for Equality of Means						
									95% Confidence Interval of the Difference	
		F	Sig.	t	df	Sig. (2-tailed)	Mean Difference	Std. Error Difference	Lower	Upper
Feeling toward biology	Equal variances assumed	.355	.553	-4.562	60	.000	-.55300	.12122	-.79548	-.31052
	Equal variances not assumed			-4.545	58.122	.000	-.55300	.12168	-.79656	-.30944

Men

Table (3.1.1.3.2) The independent samples test for feelings towards biology questionnaire to determine the F values and significance.

Factor 2 General Interest

Traditional Learning

Case Processing Summary

		N	%
Cases	Valid	30	100.0
	Excluded[a]	0	.0
	Total	30	100.0

Women

		N	%
Cases	Valid	30	100.0
	Excluded[a]	0	.0
	Total	30	100.0

Men

Table (3.1.2.1.1) Listwise deletion based on all variables in the procedure.

Reliability Statistics

Cronbach's Alpha	Cronbach's Alpha Based on Standardized Items	N of Items
.884	.887	5

Women

Cronbach's Alpha	Cronbach's Alpha Based on Standardized Items	N of Items
.831	.832	5

Men

Table (3.1.2.1.2) The Cronbach's alpha for the factor is calculated based on the number of items.

Item Statistics

	Mean	Std. Deviation	N

I like watching biology related TV.	3.50	1.280	30
biology is my favorite subject in school.	3.70	.915	30
I like reading about famous biologist	3.90	.845	30
I find what we learn in my biology class interesting.	3.50	1.075	30
I would enjoy working in a biology lab.	3.90	1.062	30

Women

Men

Table (3.1.2.1.3) The statistics for the mean and standard deviation for each item in the factor in the questionnaire.

Item-Total Statistics

	Scale Mean if Item Deleted	Scale Variance if Item Deleted	Corrected Item-Total Correlation	Squared Multiple Correlation	Cronbach's Alpha if Item Deleted
I like watching biology related TV.	15.00	10.483	.791	.633	.846
biology is my favorite subject in school.	14.80	13.338	.673	.487	.870
I like reading about famous biologiest	14.60	13.697	.682	.515	.870
I find what we learn in my biology class interesting.	15.00	11.517	.822	.689	.834
I would enjoy working in a biology lab.	14.60	12.455	.679	.512	.868

Women

Men

Table (3.1.2.1.4) The total statistics for each item in the factor in the questionnaire.

Collaborative Learning

Case Processing Summary

		N	%
Cases	Valid	31	100.0
	Excluded[a]	0	.0
	Total	31	100.0

Women

		N	%
Cases	Valid	32	100.0
	Excluded[a]	0	.0
	Total	32	100.0

Men

Table (3.1.2.2.1) Listwise deletion based on all variables in the procedure.

Reliability Statistics

Cronbach's Alpha	Cronbach's Alpha Based on Standardized Items	N of Items
.859	.857	5

Women

Cronbach's Alpha	Cronbach's Alpha Based on Standardized Items	N of Items
.725	.721	5

Men

Table (3.1.2.2.2) The Cronbach's alpha for the factor is calculated based on the number of items.

Item Statistics

	Mean	Std. Deviation	N
I like watching biology related TV.	4.19	.703	31
biology is my favourite subject in school.	4.19	.792	31
I like reading about famous biologist	4.52	.677	31
I find what we learn in my biology class interesting.	4.45	.723	31
I would enjoy working in a biology lab.	4.23	.805	31

Women

	Mean	Std. Deviation	N
I like watching biology related TV.	3.38	.793	32
biology is my favorite subject in school.	3.69	1.030	32
I like reading about famous biologist	3.47	.879	32
I find what we learn in my biology class interesting.	3.41	.911	32
I would enjoy working in a biology lab.	3.31	.965	32

Men

Table (3.1.2.1.3) The statistics for the mean and standard deviation for each item in the factor in the questionnaire.

Item-Total Statistics

	Scale Mean if Item Deleted	Scale Variance if Item Deleted	Corrected Item-Total Correlation	Squared Multiple Correlation	Cronbach's Alpha if Item Deleted
I like watching biology related TV.	17.39	6.112	.626	.416	.841
biology is my favorite subject in school.	17.39	5.245	.802	.728	.794
I like reading about famous biologiest	17.06	6.129	.656	.454	.835
I find what we learn in my biology class interesting.	17.13	6.316	.536	.316	.863
I would enjoy working in a biology lab.	17.35	5.303	.765	.697	.805

Women

Item-Total Statistics

	Scale Mean if Item Deleted	Scale Variance if Item Deleted	Corrected Item-Total Correlation	Squared Multiple Correlation	Cronbach's Alpha if Item Deleted
I like watching biology related TV.	13.88	8.048	.308	.187	.738
biology is my favorite subject in school.	13.56	6.448	.489	.310	.679
I like reading about famous biologiest	13.78	6.822	.538	.439	.659
I find what we learn in my biology class interesting.	13.84	6.910	.485	.277	.678
I would enjoy working in a biology lab.	13.94	6.190	.613	.469	.624

Men

Table (3.1.2.2.4) The total statistics for each item in the factor in the questionnaire.

TL vs CL

Group Statistics

	Method	N	Mean	Std. Deviation	Std. Error Mean
General interest	TL	30	3.6853	.81697	.14916
	CL	31	4.2739	.55708	.10005

Women

	Method	N	Mean	Std. Deviation	Std. Error Mean
General interest	TL	30	2.4627	.63258	.11549
	CL	32	3.4625	.63443	.11215

Men

Table (3.1.2.3.1) The group statistics for each item in the factors questionnaire with method.

Independent Samples Test

		Levene's Test for Equality of Variances		t-test for Equality of Means						
									97.5% Confidence Interval of the Difference	
		F	Sig.	t	df	Sig. (2-tailed)	Mean Difference	Std. Error Difference	Lower	Upper
General interest	Equal variances assumed	6.231	.015	-3.297	59	.002	-.58854	.17852	-.99913	-.17794
	Equal variances not assumed			-3.277	50.990	.002	-.58854	.17961	-1.00334	-.17373

Women

Independent Samples Test

		Levene's Test for Equality of Variances		t-test for Equality of Means						
									95% Confidence Interval of the Difference	
		F	Sig.	t	df	Sig. (2-tailed)	Mean Difference	Std. Error Difference	Lower	Upper
General interest	Equal variances assumed	.029	.866	-6.210	60	.000	-.99983	.16100	-1.32188	-.67778
	Equal variances not assumed			-6.211	59.765	.000	-.99983	.16099	-1.32188	-.67779

Men

Table (3.1.2.3.2) The independent samples test for feelings towards biology questionnaire to determine the F values and significance.

Factor 3 Motivation Towards Learning

Traditional Learning

Case Processing Summary

		N	%
Cases	Valid	30	100.0
	Excluded[a]	0	.0
	Total	30	100.0

Women

		N	%
Cases	Valid	30	100.0
	Excluded[a]	0	.0
	Total	30	100.0

Men

Table (3.1.3.1.1) Listwise deletion based on all variables in the procedure

Reliability Statistics

Cronbach's Alpha	Cronbach's Alpha Based on Standardized Items	N of Items
.850	.851	10

Women

Cronbach's Alpha	Cronbach's Alpha Based on Standardized Items	N of Items
.772	.765	10

Men

Table (3.1.3.1.2) The Cronbach's alpha for the factor is calculated based on the number of items.

Item Statistics

	Mean	Std. Deviation	N
I will ask my teacher for an explanation if I do not understand the science topic.	3.7000	.95231	30
I will look for an explanation in the textbook if I do not understand the science topic.	3.8667	.93710	30
I care about completing assignments in this class.	3.7333	.86834	30
Getting a good grade in biology is important to me.	3.6333	.88992	30
I am interested in understanding the teacher in this class.	3.6333	.92786	30
The biology I learn is relevant to my life.	3.9667	.71840	30
Learning biology is interesting.	3.6333	.80872	30
Learning biology makes my life more meaningful.	3.7000	.98786	30
I am curious about discoveries in biology.	3.7333	.94443	30
I enjoy learning biology	3.7667	1.04000	30

Women

Item Statistics

	Mean	Std. Deviation	N
I will ask my teacher for an explanation if I do not understand the science topic.	2.4333	.67891	30

I will look for an explanation in the textbook if I do not understand the science topic.	2.2667	.78492	30
I care about completing assignments in this class.	2.3333	.66089	30
Getting a good grade in biology is important to me.	2.3333	.80230	30
I am interested in understanding the teacher in this class.	2.3000	.87691	30
The biology I learn is relevant to my life.	2.3667	.76489	30
Learning biology is interesting.	2.4667	.89955	30
Learning biology makes my life more meaningful.	2.5667	.81720	30
I am curious about discoveries in biology.	2.5667	.93526	30
I enjoy learning biology	2.5333	.97320	30

Men

Table (3.1.3.1.3) The statistics for the mean and standard deviation for each item in the factor in the questionnaire.

Item-Total Statistics

	Scale Mean if Item Deleted	Scale Variance if Item Deleted	Corrected Item-Total Correlation	Squared Multiple Correlation	Cronbach's Alpha if Item Deleted
I will ask my teacher for an explanation if I do not understand the science topic.	33.6667	27.747	.667	.662	.825
I will look for an explanation in the textbook if I do not understand the science topic.	33.5000	29.224	.517	.445	.839
I care about completing assignments in this class.	33.6333	29.275	.566	.571	.835
Getting a good grade in biology is important to me.	33.7333	29.720	.498	.324	.840
I am interested in understanding the teacher in this class.	33.7333	29.444	.500	.408	.840
The biology I learn is relevant to my life.	33.4000	29.490	.684	.506	.828
Learning biology is interesting.	33.7333	31.099	.398	.512	.848
Learning biology makes my life more meaningful.	33.6667	27.678	.644	.503	.827
I am curious about discoveries in biology.	33.6333	29.895	.441	.463	.846
I enjoy learning biology	33.6000	27.490	.621	.573	.829

Women

Item-Total Statistics

	Scale Mean if Item Deleted	Scale Variance if Item Deleted	Corrected Item-Total Correlation	Squared Multiple Correlation	Cronbach's Alpha if Item Deleted
I will ask my teacher for an explanation if I do not understand the science topic.	21.7333	20.064	.289	.439	.769
I will look for an explanation in the textbook if I do not understand the science topic.	21.9000	21.128	.075	.559	.795
I care about completing assignments in this class.	21.8333	20.557	.215	.308	.776
Getting a good grade in biology is important to me.	21.8333	17.109	.682	.622	.720
I am interested in understanding the teacher in this class.	21.8667	18.051	.465	.464	.748
The biology I learn is relevant to my life.	21.8000	17.752	.612	.646	.730
Learning biology is interesting.	21.7000	16.562	.671	.642	.717
Learning biology makes my life more meaningful.	21.6000	18.248	.482	.714	.746
I am curious about discoveries in biology.	21.6000	18.317	.386	.433	.760
I enjoy learning biology	21.6333	17.275	.502	.654	.743

Men

Table (3.1.3.1.4) The total statistics for each item in the factor in the questionnaire.

Collaborative Learning

Case Processing Summary

		N	%
Cases	Valid	31	100.0
	Excluded[a]	0	.0
	Total	31	100.0

Women

Cases	Valid	32	100.0
	Excluded[a]	0	.0
	Total	32	100.0

Men

Table (3.1.3.2.1) Listwise deletion based on all variables in the procedure

Reliability Statistics

Cronbach's Alpha	Cronbach's Alpha Based on Standardized Items	N of Items
.879	.871	10

Women

Cronbach's Alpha	Cronbach's Alpha Based on Standardized Items	N of Items
.860	.852	10

Men

Table (3.1.3.2.2) The Cronbach's alpha for the factor is calculated based on the number of items.

Item Statistics

	Mean	Std. Deviation	N
I will ask my teacher for an explanation if I do not understand the science topic.	4.1613	.77875	31
I will look for an explanation in the textbook if I do not understand the science topic.	4.0645	.99785	31
I care about completing assignments in this class.	4.1613	.52261	31
Getting a good grade in biology is important to me.	4.1290	.95715	31
I am interested in understanding the teacher in this class.	4.1290	.61870	31
The biology I learn is relevant to my life.	4.1935	.83344	31
Learning biology is interesting.	4.2581	.44480	31
Learning biology makes my life more meaningful.	4.1290	.84624	31
I am curious about discoveries in biology.	4.2258	.66881	31
I enjoy learning biology	4.2581	.85509	31

Women

	Mean	Std. Deviation	N
I will ask my teacher for an explanation if I do not understand the science topic.	3.2813	.45680	32

I will look for an explanation in the textbook if I do not understand the science topic.	3.3125	.73780	32
I care about completing assignments in this class.	3.3750	.60907	32
Getting a good grade in biology is important to me.	3.3125	.73780	32
I am interested in understanding the teacher in this class.	3.5938	.61484	32
The biology I learn is relevant to my life.	3.4375	.75935	32
Learning biology is interesting.	3.5938	.71208	32
Learning biology makes my life more meaningful.	3.3750	.94186	32
I am curious about discoveries in biology.	3.3750	.60907	32
I enjoy learning biology	3.3125	.99798	32

Men

Table (3.1.3.2.3) The statistics for the mean and standard deviation for each item in the factor in the questionnaire.

Item-Total Statistics

	Scale Mean if Item Deleted	Scale Variance if Item Deleted	Corrected Item-Total Correlation	Squared Multiple Correlation	Cronbach's Alpha if Item Deleted
I will ask my teacher for an explanation if I do not understand the science topic.	37.5484	22.723	.694	.644	.860
I will look for an explanation in the textbook if I do not understand the science topic.	37.6452	20.370	.790	.810	.851
I care about completing assignments in this class.	37.5484	25.389	.535	.445	.873
Getting a good grade in biology is important to me.	37.5806	20.452	.822	.813	.847
I am interested in understanding the teacher in this class.	37.5806	26.652	.226	.344	.890
The biology I learn is relevant to my life.	37.5161	22.991	.600	.507	.867
Learning biology is interesting.	37.4516	26.656	.354	.397	.882
Learning biology makes my life more meaningful.	37.5806	22.318	.681	.650	.861
I am curious about discoveries in biology.	37.4839	24.258	.573	.451	.869
I enjoy learning biology	37.4516	21.989	.718	.658	.857

Women

Item-Total Statistics

	Scale Mean if Item Deleted	Scale Variance if Item Deleted	Corrected Item-Total Correlation	Squared Multiple Correlation	Cronbach's Alpha if Item Deleted
I will ask my teacher for an explanation if I do not understand the science topic.	30.6875	23.383	.056	.243	.876
I will look for an explanation in the textbook if I do not understand the science topic.	30.6563	19.459	.589	.592	.845
I care about completing assignments in this class.	30.5938	21.217	.401	.524	.859
Getting a good grade in biology is important to me.	30.6563	19.072	.655	.634	.840
I am interested in understanding the teacher in this class.	30.3750	20.435	.544	.542	.849
The biology I learn is relevant to my life.	30.5313	18.709	.693	.596	.836
Learning biology is interesting.	30.3750	19.081	.683	.690	.837
Learning biology makes my life more meaningful.	30.5938	17.410	.705	.754	.834
I am curious about discoveries in biology.	30.5938	19.862	.664	.768	.841
I enjoy learning biology	30.6563	17.459	.645	.738	.842

Men

Table (3.1.3.2.4) The total statistics for each item in the factor in the questionnaire.

TL vs CL

Group Statistics

	Method	N	Mean	Std. Deviation	Std. Error Mean
Motivation Towards Learning Biology	TL	30	3.7460	.56114	.10245
	CL	31	4.1713	.49010	.08802

Women

	Method	N	Mean	Std. Deviation	Std. Error Mean
Motivation Towards Learning Biology	TL	30	2.4097	.46614	.08511
	CL	32	3.3397	.41895	.07406

Men

Table (3.1.3.3.1) The group statistics for each item in the factors Questionnaire with method.

Independent Samples Test

		Levene's Test for Equality of Variances		t-test for Equality of Means						
									95% Confidence Interval of the Difference	
		F	Sig.	t	df	Sig. (2-tailed)	Mean Difference	Std. Error Difference	Lower	Upper
Motivation Towards Learning Biology	Equal variances assumed	1.187	.280	-3.156	59	.003	-.42529	.13477	-.69496	-.15562
	Equal variances not assumed			-3.149	57.389	.003	-.42529	.13507	-.69573	-.15486

Women

Independent Samples Test

		Levene's Test for Equality of Variances		t-test for Equality of Means						
									95% Confidence Interval of the Difference	
		F	Sig.	t	df	Sig. (2-tailed)	Mean Difference	Std. Error Difference	Lower	Upper
Motivation Towards Learning Biology	Equal variances assumed	.204	.653	-8.272	60	.000	-.93002	.11243	-1.15490	-.70514
	Equal variances not assumed			-8.244	58.285	.000	-.93002	.11282	-1.15583	-.70422

Men

Table (3.1.3.3.2) The independent samples test for feelings towards biology Questionnaire to determine the F values and significance.

Factor 4 Benefit and Utility of biology

Traditional Learning

Case Processing Summary

		N	%
Cases	Valid	30	100.0
	Excluded[a]	0	.0
	Total	30	100.0

Women

		N	%
Cases	Valid	30	100.0
	Excluded[a]	0	.0
	Total	30	100.0

Men

Table (3.1.4.1.1) Listwise deletion based on all variables in the procedure

Cronbach's Alpha	Cronbach's Alpha Based on Standardized Items	N of Items
.818	.821	5

Women

Cronbach's Alpha	Cronbach's Alpha Based on Standardized Items	N of Items
.889	.889	5

Men

Table (3.1.4.1.2) The Cronbach's alpha for the factor is calculated based on the number of items.

Item Statistics

	Mean	Std. Deviation	N
I use the biology that I learn in school in my life.	3.73	.944	30
What I learn in my biology class helps me understand how things work in life.	3.60	.724	30
Learning biology makes me curious about things that I observe in my life.	3.70	.596	30
What we learn in biology class helps me to understand how biology affects my life.	3.63	.615	30
Learning biology helps me to make wiser decisions about my lifestyle and health.	3.73	.980	30

Women

	Mean	Std. Deviation	N
I use the biology that I learn in school in my life.	2.93	1.048	30
What I learn in my biology class helps me understand how things work in life.	2.97	.964	30
Learning biology makes me curious about things that I observe in my life.	3.07	.944	30
What we learn in biology class helps me to understand how biology affects my life.	2.97	.999	30
Learning biology helps me to make wiser decisions about my lifestyle and health.	3.07	.907	30

Men

Table (3.1.4.1.3) The statistics for the mean and standard deviation for each item in the factor in the questionnaire.

Item-Total Statistics

	Scale Mean if Item Deleted	Scale Variance if Item Deleted	Corrected Item-Total Correlation	Squared Multiple Correlation	Cronbach's Alpha if Item Deleted
I use the biology that I learn in school in my life.	14.67	5.057	.720	.543	.748
What I learn in my biology class helps me understand how things work in life.	14.80	5.821	.762	.636	.741
Learning biology makes me curious about things that I observe in my life.	14.70	7.252	.436	.272	.827
What we learn in biology class helps me to understand how biology affects my life.	14.77	6.875	.544	.493	.803
Learning biology helps me to make wiser decisions about my lifestyle and health.	14.67	5.126	.658	.485	.774

Women

Item-Total Statistics

	Scale Mean if Item Deleted	Scale Variance if Item Deleted	Corrected Item-Total Correlation	Squared Multiple Correlation	Cronbach's Alpha if Item Deleted
I use the biology that I learn in school in my life.	12.07	9.995	.803	.676	.847
What I learn in my biology class helps me understand how things work in life.	12.03	10.654	.767	.659	.856
Learning biology makes me curious about things that I observe in my life.	11.93	11.237	.677	.551	.876
What we learn in biology class helps me to understand how biology affects my life.	12.03	10.792	.704	.548	.871
Learning biology helps me to make wiser decisions about my lifestyle and health.	11.93	11.306	.702	.577	.871

Men

Table (3.1.4.1.4) The total statistics for each item in the factor in the questionnaire.

Collaborative Learning

Case Processing Summary

		N	%
Cases	Valid	31	100.0
	Excluded[a]	0	.0
	Total	31	100.0

Women

Cases	Valid	32	100.0
	Excluded[a]	0	.0
	Total	32	100.0

Men

Table (3.1.4.2.1) Listwise deletion based on all variables in the procedure

Reliability Statistics

Cronbach's Alpha	Cronbach's Alpha Based on Standardized Items	N of Items
.881	.882	5

Women

Cronbach's Alpha	Cronbach's Alpha Based on Standardized Items	N of Items
.818	.821	5

Men

Table (3.1.4.2.2) The Cronbach's alpha for the factor is calculated based on the number of items.

Item Statistics

	Mean	Std. Deviation	N
I use the biology that I learn in school in my life.	4.16	.969	31
What I learn in my biology class helps me understand how things work in life.	4.39	1.086	31
Learning biology makes me curious about things that I observe in my life.	4.10	1.012	31
What we learn in biology class helps me to understand how biology affects my life.	4.23	1.023	31
Learning biology helps me to make wiser decisions about my lifestyle and health.	4.03	1.303	31

Women

	Mean	Std. Deviation	N
I use the biology that I learn in school in my life.	3.50	.803	32
What I learn in my biology class helps me understand how things work in life.	3.47	.761	32
Learning biology makes me curious about things that I observe in my life.	3.25	.718	32
What we learn in biology class helps me to understand how biology affects my life.	3.34	.827	32

Learning biology helps me to make wiser decisions about my lifestyle and health.	3.38	.793	32

Men

Table (3.1.4.2.3) The statistics for the mean and standard deviation for each item in the factor in the questionnaire.

Item-Total Statistics

	Scale Mean if Item Deleted	Scale Variance if Item Deleted	Corrected Item-Total Correlation	Squared Multiple Correlation	Cronbach's Alpha if Item Deleted
I use the biology that I learn in school in my life.	16.74	14.665	.586	.431	.883
What I learn in my biology class helps me understand how things work in life.	16.52	12.191	.869	.764	.818
Learning biology makes me curious about things that I observe in my life.	16.81	13.628	.710	.598	.857
What we learn in biology class helps me to understand how biology affects my life.	16.68	13.692	.689	.556	.862
Learning biology helps me to make wiser decisions about my lifestyle and health.	16.87	11.583	.752	.651	.851

Women

Item-Total Statistics

	Scale Mean if Item Deleted	Scale Variance if Item Deleted	Corrected Item-Total Correlation	Squared Multiple Correlation	Cronbach's Alpha if Item Deleted
I use the biology that I learn in school in my life.	13.44	6.190	.500	.346	.814
What I learn in my biology class helps me understand how things work in life.	13.47	5.612	.733	.665	.745
Learning biology makes me curious about things that I observe in my life.	13.69	5.964	.671	.528	.766
What we learn in biology class helps me to understand how biology affects my life.	13.59	5.926	.552	.351	.800
Learning biology helps me to make wiser decisions about my lifestyle and health.	13.56	5.867	.609	.515	.782

Men

Table (3.1.4.2.4) The total statistics for each item in the factor in the questionnaire.

TL vs CL

Group Statistics

	Method	N	Mean	Std. Deviation	Std. Error Mean
Benefit and Utility of biology	TL	30	3.6660	.57600	.10516
	CL	31	4.1635	.88576	.15909

Women

	Method	N	Mean	Std. Deviation	Std. Error Mean
Benefit and Utility of biology	TL	30	2.9827	.77728	.14191
	CL	32	3.4225	.56725	.10028

Men

Table (3.1.4.3.1) The group statistics for each item in the factors Questionnaire with method.

Independent Samples Test

		Levene's Test for Equality of Variances		t-test for Equality of Means						
									95% Confidence Interval of the Difference	
		F	Sig.	t	df	Sig. (2-tailed)	Mean Difference	Std. Error Difference	Lower	Upper
Benefit and Utility of biology	Equal variances assumed	3.775	.057	-2.591	59	.012	-.49755	.19200	-.88173	-.11336
	Equal variances not assumed			-2.609	51.729	.012	-.49755	.19070	-.88027	-.11483

Women

Independent Samples Test

		Levene's Test for Equality of Variances		t-test for Equality of Means						
									95% Confidence Interval of the Difference	
		F	Sig.	t	df	Sig. (2-tailed)	Mean Difference	Std. Error Difference	Lower	Upper
Benefit and Utility of biology	Equal variances assumed	1.323	.255	-2.557	60	.013	-.43983	.17204	-.78396	-.09571
	Equal variances not assumed			-2.531	52.861	.014	-.43983	.17377	-.78838	-.09128

Men

Table (3.1.4.3.2) The independent samples test for feelings towards biology Questionnaire to determine the F values and significance.

Factor 5 Career Motivation

Traditional Learning

Case Processing Summary

		N	%
Cases	Valid	30	100.0
	Excluded[a]	0	.0
	Total	30	100.0

Women

		N	%
Cases	Valid	30	100.0
	Excluded[a]	0	.0
	Total	30	100.0

Men

Table (3.1.5.1.1) Listwise deletion based on all variables in the procedure

Reliability Statistics

Cronbach's Alpha	Cronbach's Alpha Based on Standardized Items	N of Items
.805	.804	5

Women

Cronbach's Alpha	Cronbach's Alpha Based on Standardized Items	N of Items
.804	.807	5

Men

Table (3.1.5.1.2) The Cronbach's alpha for the factor is calculated based on the number of items.

Item Statistics

	Mean	Std. Deviation	N
Learning biology will help me get a good job.	3.37	.718	30
Knowing biology will give me a career advantage.	3.57	.898	30
Understanding biology will benefit me in my career.	3.40	.814	30
My career will involve science.	3.77	.898	30
I will use biology problem-solving skills in my career	3.50	.938	30

Women

	Mean	Std. Deviation	N
Learning biology will help me get a good job.	2.50	.682	30
Knowing biology will give me a career advantage.	2.50	.900	30
Understanding biology will benefit me in my career.	2.50	.861	30
My career will involve science.	2.53	.681	30
I will use biology problem-solving skills in my career	2.53	.937	30

Men

Table (3.1.5.1.3) The statistics for the mean and standard deviation for each item in the factor in the questionnaire.

Item-Total Statistics

	Scale Mean if Item Deleted	Scale Variance if Item Deleted	Corrected Item-Total Correlation	Squared Multiple Correlation	Cronbach's Alpha if Item Deleted
Learning biology will help me get a good job.	14.23	7.564	.566	.354	.778
Knowing biology will give me a career advantage.	14.03	6.447	.672	.466	.741
Understanding biology will benefit me in my career.	14.20	7.890	.386	.193	.825
My career will involve science.	13.83	6.420	.680	.567	.739
I will use biology problem-solving skills in my career	14.10	6.300	.667	.561	.743

Women

Item-Total Statistics

	Scale Mean if Item Deleted	Scale Variance if Item Deleted	Corrected Item-Total Correlation	Squared Multiple Correlation	Cronbach's Alpha if Item Deleted
Learning biology will help me get a good job.	10.07	7.168	.491	.375	.795
Knowing biology will give me a career advantage.	10.07	5.375	.777	.611	.701
Understanding biology will benefit me in my career.	10.07	6.685	.449	.293	.811
My career will involve science.	10.03	6.723	.634	.525	.759
I will use biology problem-solving skills in my career	10.03	5.689	.640	.464	.752

Men

Table (3.1.5.1.4) The total statistics for each item in the factor in the questionnaire.

Collaborative Learning

Case Processing Summary

		N	%
Cases	Valid	31	100.0
	Excluded[a]	0	.0
	Total	31	100.0

Women

Cases	Valid	32	100.0
	Excluded[a]	0	.0
	Total	32	100.0

Men

Table (3.1.5.2.1) Listwise deletion based on all variables in the procedure

Reliability Statistics

Cronbach's Alpha	Cronbach's Alpha Based on Standardized Items	N of Items
.879	.880	5

Women

Cronbach's Alpha	Cronbach's Alpha Based on Standardized Items	N of Items
.813	.811	5

Men

Table (3.1.5.2.2) The Cronbach's alpha for the factor is calculated based on the number of items.

Item Statistics

	Mean	Std. Deviation	N
I use the biology that I learn in school in my life.	3.94	1.093	31
What I learn in my biology class helps me understand how things work in life.	3.84	1.098	31
Learning biology makes me curious about things that I observe in my life.	3.77	.990	31
What we learn in biology class helps me to understand how biology affects my life.	4.23	.884	31
Learning biology helps me to make wiser decisions about my lifestyle and health.	3.81	1.276	31

Women

	Mean	Std. Deviation	N
I use the biology that I learn in school in my life.	2.59	.756	32
What I learn in my biology class helps me understand how things work in life.	2.94	1.045	32
Learning biology makes me curious about things that I observe in my life.	2.88	1.040	32

What we learn in biology class helps me to understand how biology affects my life.	2.81	.931	32
Learning biology helps me to make wiser decisions about my lifestyle and health.	3.09	.856	32

Men

Table (3.1.5.2.3) The statistics for the mean and standard deviation for each item in the factor in the questionnaire.

Item-Total Statistics

	Scale Mean if Item Deleted	Scale Variance if Item Deleted	Corrected Item-Total Correlation	Squared Multiple Correlation	Cronbach's Alpha if Item Deleted
I use the biology that I learn in school in my life.	15.65	12.703	.721	.540	.851
What I learn in my biology class helps me understand how things work in life.	15.74	12.598	.733	.606	.848
Learning biology makes me curious about things that I observe in my life.	15.81	13.295	.726	.531	.851
What we learn in biology class helps me to understand how biology affects my life.	15.35	14.637	.607	.429	.877
Learning biology helps me to make wiser decisions about my lifestyle and health.	15.77	11.114	.797	.661	.834

Women

Item-Total Statistics

	Scale Mean if Item Deleted	Scale Variance if Item Deleted	Corrected Item-Total Correlation	Squared Multiple Correlation	Cronbach's Alpha if Item Deleted
I use the biology that I learn in school in my life.	11.72	9.757	.442	.326	.818
What I learn in my biology class helps me understand how things work in life.	11.38	7.726	.619	.480	.772
Learning biology makes me curious about things that I observe in my life.	11.44	7.222	.736	.565	.731
What we learn in biology class helps me to understand how biology affects my life.	11.50	8.774	.503	.287	.805
Learning biology helps me to make wiser decisions about my lifestyle and health.	11.22	8.112	.732	.558	.740

Men

Table (3.1.5.2.4) The total statistics for each item in the factor in the questionnaire.

TL vs CL

Group Statistics

	Method	N	Mean	Std. Deviation	Std. Error Mean
Career Motivation	TL	30	3.5143	.63659	.11622
	CL	31	3.9106	.85870	.15423

Women

	Method	N	Mean	Std. Deviation	Std. Error Mean
Career Motivation	TL	30	2.4843	.61567	.11241
	CL	32	2.8659	.70145	.12400

Men

Table (3.1.5.3.1) The group statistics for each item in the factors Questionnaire with method.

Independent Samples Test

		Levene's Test for Equality of Variances		t-test for Equality of Means						
									97.5% Confidence Interval of the Difference	
		F	Sig.	t	df	Sig. (2-tailed)	Mean Difference	Std. Error Difference	Lower	Upper
Career Motivation	Equal variances assumed	2.095	.153	-2.042	59	.046	-.39631	.19405	-.84265	.05002
	Equal variances not assumed			-2.052	55.300	.045	-.39631	.19312	-.84127	.04864

Women

Independent Samples Test

		Levene's Test for Equality of Variances		t-test for Equality of Means						
									95% Confidence Interval of the Difference	
		F	Sig.	t	df	Sig. (2-tailed)	Mean Difference	Std. Error Difference	Lower	Upper
Career Motivation	Equal variances assumed	.695	.408	-2.270	60	.027	-.38160	.16808	-.71781	-.04540
	Equal variances not assumed			-2.280	59.751	.026	-.38160	.16737	-.71641	-.04680

Men

Table (3.1.5.3.2) The independent samples test for feelings towards biology Questionnaire to determine the F values and significance.

Factor 6 Self-Efficacy in Biology Learning

Traditional Learning

Case Processing Summary

		N	%
Cases	Valid	30	100.0
	Excluded[a]	0	.0
	Total	30	100.0

Women

		N	%
Cases	Valid	30	100.0
	Excluded[a]	0	.0
	Total	30	100.0

Men

Table (3.1.6.1.1) Listwise deletion based on all variables in the procedure

Reliability Statistics

Cronbach's Alpha	Cronbach's Alpha Based on Standardized Items	N of Items
.789	.785	8

Women

Cronbach's Alpha	Cronbach's Alpha Based on Standardized Items	N of Items
.761	.754	8

Men

Table (3.1.6.1.2) The Cronbach’s alpha for the factor is calculated based on the number of items.

Item Statistics

	Mean	Std. Deviation	N
If I study hard I can do well in biology	3.7667	.85836	30
I believe biology is too easy for me to learn	3.6667	.92227	30
The idea of taking biology makes me excited.	3.9333	.86834	30
I am confident I will do well on biology tests.	3.9667	.61495	30
I am confident I will do well on biology labs and projects.	3.6667	.84418	30
I believe I can master biology knowledge and skills.	3.7333	.82768	30
I believe I can earn a grade of "A" in biology.	3.9333	.78492	30
I am sure I can understand biology.	4.1000	.92289	30

Women

	Mean	Std. Deviation	N
If I study hard I can do well in biology	2.2000	.80516	30
I believe biology is too easy for me to learn	2.1333	.89955	30
The idea of taking biology makes me excited.	2.1667	.87428	30
I am confident I will do well on biology tests.	2.4000	.72397	30
I am confident I will do well on biology labs and projects.	2.4667	.73030	30
I believe I can master biology knowledge and skills.	2.0667	.63968	30

I believe I can earn a grade of "A" in biology.	2.3667	.71840	30
I am sure I can understand biology.	2.1000	.80301	30

Men

Table (3.1.6.1.3) The statistics for the mean and standard deviation for each item in the factor in the questionnaire.

Item-Total Statistics

	Scale Mean if Item Deleted	Scale Variance if Item Deleted	Corrected Item-Total Correlation	Squared Multiple Correlation	Cronbach's Alpha if Item Deleted
If I study hard I can do well in biology	27.0000	13.034	.690	.730	.733
I believe biology is too easy for me to learn	27.1000	13.955	.470	.561	.771
The idea of taking biology makes me excited.	26.8333	12.626	.756	.786	.720
I am confident I will do well on biology tests.	26.8000	15.959	.348	.347	.786
I am confident I will do well on biology labs and projects.	27.1000	14.921	.370	.359	.786
I believe I can master biology knowledge and skills.	27.0333	14.930	.380	.507	.784
I believe I can earn a grade of "A" in biology.	26.8333	15.040	.393	.520	.781
I am sure I can understand biology.	26.6667	13.402	.561	.680	.755

Women

Item-Total Statistics

	Scale Mean if Item Deleted	Scale Variance if Item Deleted	Corrected Item-Total Correlation	Squared Multiple Correlation	Cronbach's Alpha if Item Deleted
If I study hard I can do well in biology	15.7000	10.700	.600	.497	.709
I believe biology is too easy for me to learn	15.7667	10.185	.612	.603	.704
The idea of taking biology makes me excited.	15.7333	10.961	.480	.380	.732
I am confident I will do well on biology tests.	15.5000	12.672	.254	.367	.768
I am confident I will do well on biology labs and projects.	15.4333	11.840	.424	.401	.741
I believe I can master biology knowledge and skills.	15.8333	12.351	.389	.452	.747
I believe I can earn a grade of "A" in biology.	15.5333	12.326	.330	.254	.757
I am sure I can understand biology.	15.8000	10.855	.568	.467	.715

Men

Table (3.1.6.1.4) The total statistics for each item in the factor in the questionnaire.

Collaborative Learning

Case Processing Summary

		N	%
Cases	Valid	31	100.0
	Excluded[a]	0	.0
	Total	31	100.0

Women

Cases	Valid	32	100.0
	Excluded[a]	0	.0
	Total	32	100.0

Men

Table (3.1.6.2.1) Listwise deletion based on all variable in the procedure

Reliability Statistics

Cronbach's Alpha	Cronbach's Alpha Based on Standardized Items	N of Items
.827	.812	8

Women

Cronbach's Alpha	Cronbach's Alpha Based on Standardized Items	N of Items
.858	.857	8

Men

Table (3.1.6.2.2) The Cronbach's alpha for the factor is calculated based on the number of items.

Item Statistics

	Mean	Std. Deviation	N
If I study hard I can do well in biology	4.0645	.85383	31
I believe biology is too easy for me to learn	3.7097	.97275	31
The idea of taking biology makes me excited.	3.8387	1.00322	31
I am confident I will do well on biology tests.	3.9677	.75206	31
I am confident I will do well on biology labs and projects.	4.0000	.93095	31
I believe I can master biology knowledge and skills.	4.0323	.83602	31
I believe I can earn a grade of “A” in biology.	4.1613	.68784	31
I am sure I can understand biology.	4.0645	1.03071	31

Women

	Mean	Std. Deviation	N
If I study hard I can do well in biology	3.6563	.65300	32
I believe biology is too easy for me to learn	3.6563	.65300	32
The idea of taking biology makes me excited.	3.6250	.65991	32
I am confident I will do well on biology tests.	3.6250	.70711	32
I am confident I will do well on biology labs and projects.	3.4688	.56707	32
I believe I can master biology knowledge and skills.	3.5625	.66901	32

I believe I can earn a grade of "A" in biology.	3.6875	.59229	32
I am sure I can understand biology.	3.5938	.71208	32

Men

Table (3.1.6.2.3) The statistics for the mean and standard deviation for each item in the factor in the questionnaire.

Item-Total Statistics

	Scale Mean if Item Deleted	Scale Variance if Item Deleted	Corrected Item-Total Correlation	Squared Multiple Correlation	Cronbach's Alpha if Item Deleted
If I study hard I can do well in biology	27.7742	17.181	.711	.613	.785
I believe biology is too easy for me to learn	28.1290	16.783	.654	.624	.791
The idea of taking biology makes me excited.	28.0000	16.733	.634	.520	.794
I am confident I will do well on biology tests.	27.8710	19.983	.356	.363	.829
I am confident I will do well on biology labs and projects.	27.8387	16.673	.710	.563	.783
I believe I can master biology knowledge and skills.	27.8065	18.561	.511	.435	.812
I believe I can earn a grade of "A" in biology.	27.6774	21.959	.079	.211	.855
I am sure I can understand biology.	27.7742	16.114	.696	.621	.784

Women

Item-Total Statistics

	Scale Mean if Item Deleted	Scale Variance if Item Deleted	Corrected Item-Total Correlation	Squared Multiple Correlation	Cronbach's Alpha if Item Deleted
If I study hard I can do well in biology	25.2188	11.144	.494	.350	.854
I believe biology is too easy for me to learn	25.2188	10.305	.714	.586	.828
The idea of taking biology makes me excited.	25.2500	10.129	.753	.584	.823
I am confident I will do well on biology tests.	25.2500	10.129	.688	.554	.831
I am confident I will do well on biology labs and projects.	25.4063	11.604	.466	.261	.856
I believe I can master biology knowledge and skills.	25.3125	10.931	.530	.380	.850
I believe I can earn a grade of "A" in biology.	25.1875	11.319	.516	.328	.851
I am sure I can understand biology.	25.2813	10.209	.662	.467	.834

Men

Table (3.1.6.2.4) The total statistics for each item in the factor in the questionnaire.

TL vs CL

Group Statistics

	Method	N	Mean	Std. Deviation	Std. Error Mean
Career Motivation	TL	30	3.8397	.51851	.09467
	CL	31	4.0161	.57397	.10309

Women

	Method	N	Mean	Std. Deviation	Std. Error Mean
Career Motivation	TL	30	2.2503	.45640	.08333
	CL	32	3.5881	.41333	.07307

Men

Table (3.1.6.3.1) The group statistics for each item in the factors Questionnaire with method.

Independent Samples Test

		Levene's Test for Equality of Variances		t-test for Equality of Means						
									95% Confidence Interval of the Difference	
		F	Sig.	t	df	Sig. (2-tailed)	Mean Difference	Std. Error Difference	Lower	Upper
Self-Efficacy in biology Learning	Equal variances assumed	1.233	.271	-1.259	59	.213	-.17646	.14020	-.45700	.10407
	Equal variances not assumed			-1.261	58.728	.212	-.17646	.13996	-.45655	.10363

Women

Independent Samples Test

		Levene's Test for Equality of Variances		t-test for Equality of Means						
									95% Confidence Interval of the Difference	
		F	Sig.	t	df	Sig. (2-tailed)	Mean Difference	Std. Error Difference	Lower	Upper
Self-Efficacy in biology Learning	Equal variances assumed	1.162	.285	-12.110	60	.000	-1.33779	.11047	-1.55876	-1.11683
	Equal variances not assumed			-12.071	58.427	.000	-1.33779	.11082	-1.55960	-1.11599

Men

Table (3.1.6.3.2) The independent samples test for factor in the questionnaire to determine the F values and significance.

Factor 7 Self-Determination

Traditional Learning

Case Processing Summary

		N	%
Cases	Valid	30	100.0
	Excluded[a]	0	.0
	Total	30	100.0

Women

		N	%
Cases	Valid	30	100.0
	Excluded[a]	0	.0
	Total	30	100.0

Men

Table (3.1.7.1.1) Listwise deletion based on all variables in the procedure

Reliability Statistics

Cronbach's Alpha	Cronbach's Alpha Based on Standardized Items	N of Items
.771	.765	5

Women

Cronbach's Alpha	Cronbach's Alpha Based on Standardized Items	N of Items
.755	.764	5

Men

Table (3.1.7.1.2) The Cronbach's alpha for the factor is calculated based on the number of items.

Item Statistics

	Mean	Std. Deviation	N
I put enough effort into learning biology.	3.33	.959	30
I use strategies to learn biology well.	3.27	.980	30
I spend a lot of time learning biology.	3.60	1.037	30
I prepare well for biology tests and labs.	3.47	.819	30
I study hard to learn biology.	3.50	.974	30

Women

	Mean	Std. Deviation	N
I put enough effort into learning biology.	2.63	.928	30
I use strategies to learn biology well.	2.87	.860	30
I spend a lot of time learning biology.	2.80	.761	30
I prepare well for biology tests and labs.	2.60	.894	30
I study hard to learn biology.	2.83	1.117	30

Men

Table (3.1.7.1.3) The statistics for the mean and standard deviation for each item in the factor in the questionnaire.

Item-Total Statistics

	Scale Mean if Item Deleted	Scale Variance if Item Deleted	Corrected Item-Total Correlation	Squared Multiple Correlation	Cronbach's Alpha if Item Deleted
I put enough effort into learning biology.	13.83	8.006	.555	.378	.724
I use strategies to learn biology well.	13.90	7.403	.670	.634	.682
I spend a lot of time learning biology.	13.57	7.564	.578	.484	.716
I prepare well for biology tests and labs.	13.70	9.666	.314	.268	.795
I study hard to learn biology.	13.67	7.747	.598	.421	.709

Women

Item-Total Statistics

	Scale Mean if Item Deleted	Scale Variance if Item Deleted	Corrected Item-Total Correlation	Squared Multiple Correlation	Cronbach's Alpha if Item Deleted
I put enough effort into learning biology.	11.10	8.162	.314	.102	.783
I use strategies to learn biology well.	10.87	7.361	.553	.320	.702
I spend a lot of time learning biology.	10.93	7.306	.681	.504	.668
I prepare well for biology tests and labs.	11.13	7.361	.520	.293	.712
I study hard to learn biology.	10.90	6.093	.607	.462	.681

Men

Table (3.1.7.1.4) The total statistics for each item in the factor in the questionnaire.

Collaborative Learning

Case Processing Summary

		N	%
Cases	Valid	31	100.0
	Excluded[a]	0	.0
	Total	31	100.0

Women

Cases	Valid	32	100.0
	Excluded[a]	0	.0
	Total	32	100.0

Men

Table (3.1.7.2.1) Listwise deletion based on all variable in the procedure

Reliability Statistics

Cronbach's Alpha	Cronbach's Alpha Based on Standardized Items	N of Items
.775	.778	5

Women

Cronbach's Alpha	Cronbach's Alpha Based on Standardized Items	N of Items
.719	.711	5

Men

Table (3.1.7.2.2) The Cronbach's alpha for the factor is calculated based on the number of items.

Item Statistics

	Mean	Std. Deviation	N
I use the biology that I learn in school in my life.	3.84	.735	31
What I learn in my biology class helps me understand how things work in life.	3.94	.727	31
Learning biology makes me curious about things that I observe in my life.	3.61	.715	31
What we learn in biology class helps me to understand how biology affects my life.	4.00	.683	31
Learning biology helps me to make wiser decisions about my lifestyle and health.	3.65	.839	31

Women

	Mean	Std. Deviation	N
I use the biology that I learn in school in my life.	3.19	.592	32
What I learn in my biology class helps me understand how things work in life.	3.34	.787	32
Learning biology makes me curious about things that I observe in my life.	3.25	.762	32
What we learn in biology class helps me to understand how biology affects my life.	3.09	.641	32

Learning biology helps me to make wiser decisions about my lifestyle and health.	3.31	.738	32

Men

Table (3.1.7.2.3) The statistics for the mean and standard deviation for each item in the factor in the questionnaire.

Item-Total Statistics

	Scale Mean if Item Deleted	Scale Variance if Item Deleted	Corrected Item-Total Correlation	Squared Multiple Correlation	Cronbach's Alpha if Item Deleted
I use the biology that I learn in school in my life.	15.19	4.895	.553	.414	.731
What I learn in my biology class helps me understand how things work in life.	15.10	4.557	.691	.510	.683
Learning biology makes me curious about things that I observe in my life.	15.42	4.652	.670	.467	.692
What we learn in biology class helps me to understand how biology affects my life.	15.03	5.432	.419	.188	.773
Learning biology helps me to make wiser decisions about my lifestyle and health.	15.39	4.912	.435	.265	.778

Women

Item-Total Statistics

	Scale Mean if Item Deleted	Scale Variance if Item Deleted	Corrected Item-Total Correlation	Squared Multiple Correlation	Cronbach's Alpha if Item Deleted
I use the biology that I learn in school in my life.	13.00	4.903	.246	.132	.747
What I learn in my biology class helps me understand how things work in life.	12.84	3.620	.554	.335	.639
Learning biology makes me curious about things that I observe in my life.	12.94	3.867	.484	.351	.669
What we learn in biology class helps me to understand how biology affects my life.	13.09	4.217	.484	.247	.671
Learning biology helps me to make wiser decisions about my lifestyle and health.	12.88	3.597	.628	.439	.606

Men

Table (3.1.6.2.4) The total statistics for each item in the factor in the questionnaire.

TL vs CL

Group Statistics

	Method	N	Mean	Std. Deviation	Std. Error Mean
Self-Determination	TL	30	3.4437	.70457	.12864
	CL	31	3.7926	.44180	.07935

Women

	Method	N	Mean	Std. Deviation	Std. Error Mean
Self-Determination	TL	30	2.7290	.64614	.11797
	CL	32	3.2597	.48398	.08556

Men

Table (3.1.7.3.1) The group statistics for each item in the factor's questionnaire with method.

Independent Samples Test

		Levene's Test for Equality of Variances		t-test for Equality of Means					95% Confidence Interval of the Difference	
		F	Sig.	t	df	Sig. (2-tailed)	Mean Difference	Std. Error Difference	Lower	Upper
Self-Determination	Equal variances assumed	1.971	.166	-2.325	59	.024	-.34891	.15005	-.64916	-.04867
	Equal variances not assumed			-2.309	48.482	.025	-.34891	.15114	-.65273	-.04510

Women

Independent Samples Test

		Levene's Test for Equality of Variances		t-test for Equality of Means						
									95% Confidence Interval of the Difference	
		F	Sig.	t	df	Sig. (2-tailed)	Mean Difference	Std. Error Difference	Lower	Upper
Self-Determination	Equal variances assumed	1.622	.208	-3.675	60	.001	-.53069	.14439	-.81951	-.24187
	Equal variances not assumed			-3.642	53.646	.001	-.53069	.14573	-.82290	-.23848

Men

Table (3.1.7.3.2) The independent samples test for factors to determine the F values and significance.

Factor 8 Grade Motivation

Traditional Learning

Case Processing Summary

		N	%
Cases	Valid	30	100.0
	Excluded[a]	0	.0
	Total	30	100.0

Women

		N	%
Cases	Valid	30	100.0
	Excluded[a]	0	.0
	Total	30	100.0

Men

Table (3.1.8.1.1) Listwise deletion based on all variables in the procedure

Reliability Statistics

Cronbach's Alpha	Cronbach's Alpha Based on Standardized Items	N of Items
.753	.754	5

Women

Cronbach's Alpha	Cronbach's Alpha Based on Standardized Items	N of Items
.770	.746	5

Men

Table (3.1.8.1.2) The Cronbach's alpha for the factor is calculated based on the number of items.

Item Statistics

	Mean	Std. Deviation	N

I like to do better than other students on biology tests.	3.77	.774	30
Getting a good biology grade is important to me.	3.67	.844	30
It is important that I get an "A" in biology.	4.00	.788	30
I think about the grade I will get in biology.	4.07	.740	30
Scoring high on biology tests and labs matters to me.	3.80	1.095	30

Women

	Mean	Std. Deviation	N
I like to do better than other students on biology tests.	2.60	.498	30
Getting a good biology grade is important to me.	2.63	.669	30
It is important that I get an "A" in biology.	2.70	.651	30
I think about the grade I will get in biology.	2.50	.682	30
Scoring high on biology tests and labs matters to me.	2.57	.817	30

Men

Table (3.1.8.1.3) The statistics for the mean and standard deviation for each item in the factor in the questionnaire.

Item-Total Statistics

	Scale Mean if Item Deleted	Scale Variance if Item Deleted	Corrected Item-Total Correlation	Squared Multiple Correlation	Cronbach's Alpha if Item Deleted
I like to do better than other students on biology tests.	15.53	6.602	.516	.555	.711
Getting a good biology grade is important to me.	15.63	5.964	.624	.462	.670
It is important that I get an "A" in biology.	15.30	7.114	.361	.355	.760
I think about the grade I will get in biology.	15.23	6.737	.512	.425	.713
Scoring high on biology tests and labs matters to me.	15.50	5.017	.618	.408	.674

Women

Item-Total Statistics

	Scale Mean if Item Deleted	Scale Variance if Item Deleted	Corrected Item-Total Correlation	Squared Multiple Correlation	Cronbach's Alpha if Item Deleted
I like to do better than other students on biology tests.	10.40	5.283	.145	.120	.827
Getting a good biology grade is important to me.	10.37	3.689	.672	.526	.681
It is important that I get an "A" in biology.	10.30	4.148	.486	.402	.746
I think about the grade I will get in biology.	10.50	3.707	.643	.475	.690
Scoring high on biology tests and labs matters to me.	10.43	3.013	.769	.609	.632

Men

Table (3.1.8.1.4) The total statistics for each item in the factor in the questionnaire.

Collaborative Learning

Case Processing Summary

		N	%
Cases	Valid	31	100.0
	Excluded[a]	0	.0
	Total	31	100.0

Women

Cases	Valid	32	100.0
	Excluded[a]	0	.0
	Total	32	100.0

Men

Table (3.1.8.2.1) Listwise deletion based on all variable in the procedure

Reliability Statistics

Cronbach's Alpha	Cronbach's Alpha Based on Standardized Items	N of Items
.889	.892	5

Women

Cronbach's Alpha	Cronbach's Alpha Based on Standardized Items	N of Items
.819	.816	5

Men

Table (3.1.8.2.2) The Cronbach's alpha for the factor is calculated based on the number of items.

Item Statistics

	Mean	Std. Deviation	N
I like to do better than other students on biology tests.	4.03	1.016	31
Getting a good biology grade is important to me.	4.19	.792	31
It is important that I get an "A" in biology.	3.94	.854	31
I think about the grade I will get in biology.	4.13	.846	31
Scoring high on biology tests and labs matters to me.	4.06	.814	31

Women

	Mean	Std. Deviation	N
I use the biology that I learn in school in my life.	3.00	.622	32
What I learn in my biology class helps me understand how things work in life.	3.13	.793	32
Learning biology makes me curious about things that I observe in my life.	3.03	.822	32
What we learn in biology class helps me to understand how biology affects my life.	3.34	.653	32
Learning biology helps me to make wiser decisions about my lifestyle and health.	3.13	1.040	32

Men

Table (3.1.8.2.3) The statistics for the mean and standard deviation for each item in the factor in the questionnaire.

Item-Total Statistics

	Scale Mean if Item Deleted	Scale Variance if Item Deleted	Corrected Item-Total Correlation	Squared Multiple Correlation	Cronbach's Alpha if Item Deleted
I like to do better than other students on biology tests.	16.32	7.892	.720	.571	.871
Getting a good biology grade is important to me.	16.16	9.006	.715	.576	.869
It is important that I get an "A" in biology.	16.42	8.585	.744	.560	.861
I think about the grade I will get in biology.	16.23	8.514	.771	.630	.855
Scoring high on biology tests and labs matters to me.	16.29	8.880	.720	.607	.867

Women

Item-Total Statistics

	Scale Mean if Item Deleted	Scale Variance if Item Deleted	Corrected Item-Total Correlation	Squared Multiple Correlation	Cronbach's Alpha if Item Deleted
I use the biology that I learn in school in my life.	12.63	7.339	.459	.357	.822
What I learn in my biology class helps me understand how things work in life.	12.50	6.129	.641	.568	.774
Learning biology makes me curious about things that I observe in my life.	12.59	5.604	.769	.646	.733
What we learn in biology class helps me to understand how biology affects my life.	12.28	7.112	.498	.259	.813
Learning biology helps me to make wiser decisions about my lifestyle and health.	12.50	4.839	.734	.543	.749

Men

Table (3.1.8.2.4) The total statistics for each item in the factor in the questionnaire.

TL vs CL

Group Statistics

	Method	N	Mean	Std. Deviation	Std. Error Mean
Self-Determination	TL	30	3.8507	.58749	.10726
	CL	31	4.0103	.69315	.12449

Women

	Method	N	Mean	Std. Deviation	Std. Error Mean
Self-Determination	TL	30	2.5647	.39650	.07239
	CL	32	3.1003	.60219	.10645

Men

Table (3.1.8.3.1) The group statistics for each item in the factor's questionnaire with method.

Independent Samples Test

		Levene's Test for Equality of Variances		t-test for Equality of Means						
									95% Confidence Interval of the Difference	
		F	Sig.	t	df	Sig. (2-tailed)	Mean Difference	Std. Error Difference	Lower	Upper
Grade Motivation	Equal variances assumed	.000	.991	-.969	59	.337	-.15966	.16478	-.48937	.17006
	Equal variances not assumed			-.972	58.005	.335	-.15966	.16433	-.48859	.16928

Women

Independent Samples Test

		Levene's Test for Equality of Variances		t-test for Equality of Means						
									95% Confidence Interval of the Difference	
		F	Sig.	t	df	Sig. (2-tailed)	Mean Difference	Std. Error Difference	Lower	Upper
Grade Motivation	Equal variances assumed	1.429	.237	-4.107	60	.000	-.53565	.13041	-.79651	-.27478
	Equal variances not assumed			-4.161	53.964	.000	-.53565	.12873	-.79375	-.27754

Men

Table (3.1.8.3.2) The independent samples test for the factor to determine the F values and significance.

Factor 9 Assessment anxiety

Traditional Learning

Case Processing Summary

		N	%
Cases	Valid	30	100.0
	Excluded[a]	0	.0
	Total	30	100.0

Women

		N	%
Cases	Valid	30	100.0
	Excluded[a]	0	.0
	Total	30	100.0

Men

Table (3.1.9.1.1) Listwise deletion based on all variables in the procedure

Reliability Statistics

Cronbach's Alpha	Cronbach's Alpha Based on Standardized Items	N of Items
.809	.816	5

Women

Cronbach's Alpha	Cronbach's Alpha Based on Standardized Items	N of Items
.778	.781	5

Men

Table (3.1.9.1.2) The Cronbach's alpha for the factor is calculated based on the number of items.

Item Statistics

	Mean	Std. Deviation	N
I am nervous about how I will do on the biology tests.	3.47	1.008	30
I become anxious when it is time to take a biology test.	3.73	.868	30
I worry about failing the biology tests.	3.27	.944	30
I am concerned that the other students are better in biology.	3.63	.850	30
I hate taking the biology tests.	3.53	1.074	30

Women

	Mean	Std. Deviation	N
I am nervous about how I will do on the biology tests.	2.30	.794	30
I become anxious when it is time to take a biology test.	2.40	.814	30
I worry about failing the biology tests.	2.37	.890	30
I am concerned that the other students are better in biology.	2.53	.776	30
I hate taking the biology tests.	2.40	1.003	30

Men

Table (3.1.9.1.3) The statistics for the mean and standard deviation for each item in the factor in the questionnaire.

Item-Total Statistics

	Scale Mean if Item Deleted	Scale Variance if Item Deleted	Corrected Item-Total Correlation	Squared Multiple Correlation	Cronbach's Alpha if Item Deleted
I am nervous about how I will do on the biology tests.	14.17	8.971	.476	.301	.810
I become anxious when it is time to take a biology test.	13.90	8.231	.778	.615	.720
I worry about failing the biology tests.	14.37	8.378	.657	.470	.753
I am concerned that the other students are better in biology.	14.00	9.172	.576	.423	.779
I hate taking the biology tests.	14.10	8.369	.537	.310	.794

Women

Item-Total Statistics

	Scale Mean if Item Deleted	Scale Variance if Item Deleted	Corrected Item-Total Correlation	Squared Multiple Correlation	Cronbach's Alpha if Item Deleted
I am nervous about how I will do on the biology tests.	9.70	6.700	.599	.383	.724
I become anxious when it is time to take a biology test.	9.60	6.455	.647	.455	.707
I worry about failing the biology tests.	9.63	6.723	.494	.255	.758
I am concerned that the other students are better in biology.	9.47	7.292	.453	.215	.768
I hate taking the biology tests.	9.60	5.903	.591	.377	.726

Men

Table (3.1.9.1.4) The total statistics for each item in the factor in the questionnaire.

Collaborative Learning

Case Processing Summary

		N	%
Cases	Valid	31	100.0
	Excluded[a]	0	.0
	Total	31	100.0

Women

Cases	Valid	32	100.0
	Excluded[a]	0	.0
	Total	32	100.0

Men

Table (3.1.9.2.1) Listwise deletion based on all variable in the procedure

Reliability Statistics

Cronbach's Alpha	Cronbach's Alpha Based on Standardized Items	N of Items
.729	.695	5

Women

Cronbach's Alpha	Cronbach's Alpha Based on Standardized Items	N of Items
.778	.774	5

Men

Table (3.1.9.2.2) The Cronbach's alpha for the factor is calculated based on the number of items.

Item Statistics

	Mean	Std. Deviation	N
I am nervous about how I will do on the biology tests.	4.29	.739	31
I become anxious when it is time to take a biology test.	4.39	.803	31
I worry about failing the biology tests.	4.16	.523	31
I am concerned that the other students are better in biology.	4.13	.991	31
I hate taking the biology tests.	4.45	.810	31

Women

Table (3.1.9.2.3) The statistics for the mean and standard deviation for each item in the factor in the questionnaire.

Item-Total Statistics

	Scale Mean if Item Deleted	Scale Variance if Item Deleted	Corrected Item-Total Correlation	Squared Multiple Correlation	Cronbach's Alpha if Item Deleted
I am nervous about how I will do on the biology tests.	17.13	5.049	.559	.421	.658
I become anxious when it is time to take a biology test.	17.03	5.232	.428	.360	.707
I worry about failing the biology tests.	17.26	6.998	.065	.178	.794
I am concerned that the other students are better in biology.	17.29	3.813	.686	.571	.592
I hate taking the biology tests.	16.97	4.366	.718	.608	.586

Women
Men

Table (3.1.9.2.4) The total statistics for each item in the factor in the questionnaire.

TL vs CL

Group Statistics

	Method	N	Mean	Std. Deviation	Std. Error Mean
Self-Determination	TL	30	3.5043	.71399	.13036
	CL	31	4.2706	.57835	.10387

Women

	Method	N	Mean	Std. Deviation	Std. Error Mean
Self-Determination	TL	30	2.4180	.60067	.10967
	CL	32	3.4188	.69515	.12289

Men

Table (3.1.9.3.1) The group statistics for each item in the factor's questionnaire with method.

Independent Samples Test

		Levene's Test for Equality of Variances		t-test for Equality of Means						
									95% Confidence Interval of the Difference	
		F	Sig.	t	df	Sig. (2-tailed)	Mean Difference	Std. Error Difference	Lower	Upper
Aassessment anxiety	Equal variances assumed	2.005	.162	-4.613	59	.000	-.76631	.16611	-1.09869	-.43394
	Equal variances not assumed			-4.597	55.780	.000	-.76631	.16668	-1.10024	-.43238

Women

Independent Samples Test

		Levene's Test for Equality of Variances		t-test for Equality of Means						
									95% Confidence Interval of the Difference	
		F	Sig.	t	df	Sig. (2-tailed)	Mean Difference	Std. Error Difference	Lower	Upper
Aassessment anxiety	Equal variances assumed	.932	.338	-6.047	60	.000	-1.00075	.16549	-1.33178	-.66972
	Equal variances not assumed			-6.076	59.618	.000	-1.00075	.16470	-1.33025	-.67125

Men

Table (3.1.9.3.2) The independent samples test for factor in questionnaire to determine the F values and significance.

All factors comparison

Group Statistics

	Method	N	Mean	Std. Deviation	Std. Error Mean
Attitude_9 Factors	TL	270	3.5907	.66377	.04040
	CL	279	4.0433	.64799	.03879

Women

	Method	N	Mean	Std. Deviation	Std. Error Mean
Attitude_9 Factors	TL	270	2.5393	.60263	.03667
	CL	288	3.2847	.59377	.03499

Men

Table (3.1.10.3.1) The group statistics for each item in the factor's questionnaire with method

Independent Samples Test

		Levene's Test for Equality of Variances		t-test for Equality of Means						
									95% Confidence Interval of the Difference	
		F	Sig.	t	df	Sig. (2-tailed)	Mean Difference	Std. Error Difference	Lower	Upper
Attitude_9 Factors	Equal variances assumed	1.136	.287	-8.084	547	.000	-.45259	.05599	-.56257	-.34262
	Equal variances not assumed			-8.081	545.235	.000	-.45259	.05601	-.56261	-.34258

Women

Independent Samples Test

		Levene's Test for Equality of Variances		t-test for Equality of Means						
									95% Confidence Interval of the Difference	
		F	Sig.	t	df	Sig. (2-tailed)	Mean Difference	Std. Error Difference	Lower	Upper
Attitude_9 Factors	Equal variances assumed	.057	.811	-14.714	556	.000	-.74546	.05066	-.84498	-.64595
	Equal variances not assumed			-14.707	552.511	.000	-.74546	.05069	-.84503	-.64590

Men

Table (3.1.10.3.2) The independent samples test for all factor in questionnaire to determine the F values and significance.

Attitude Women TL (All factors) VS Men TL (All factors)

Between-Subjects Factors

		Value Label	N
Gender	1.00	Women	269
	2.00	Men	270
Method	1.00	TL	539

Table (3.1.10.3.3) The subject factor based on gender and method

Descriptive Statistics

Dependent Variable: Attitude_9 Factors

Gender	Method	Mean	Std. Deviation	N
Women	TL	3.7002	.64419	269
	Total	3.7002	.64419	269
Men	TL	2.5393	.60263	270
	Total	2.5393	.60263	270
Total	TL	3.1186	.85197	539
	Total	3.1186	.85197	539

Table (3.1.10.3.4) The mean and standard deviation of students in TL

Estimates

Dependent Variable: Attitude_9 Factors

			95% Confidence Interval	
Gender	Mean	Std. Error	Lower Bound	Upper Bound
Women	3.700	.038	3.625	3.775
Men	2.539	.038	2.465	2.614

Table (3.1.10.3.5) Estimates of mean and standard deviation for women and men in TL

Univariate Tests

Dependent Variable: Attitude_9 Factors

	Sum of Squares	df	Mean Square	F	Sig.	Partial Eta Squared
Contrast	181.603	1	181.603	466.819	.000	.465
Error	208.905	537	.389			

Table (3.1.10.3.6) The F tests the effect of Gender. This test is based on the linearly independent pairwise comparisons among the estimated marginal means.

Group Statistics

	Gender	N	Mean	Std. Deviation	Std. Error Mean
Attitude_9 Factors	Women	269	3.7002	.64419	.03928
	Men	270	2.5393	.60263	.03667

Table (3.1.10.3.7) The group statistics for women TL and men TL with method.

Independent Samples Test

		Levene's Test for Equality of Variances		t-test for Equality of Means						
									95% Confidence Interval of the Difference	
		F	Sig.	t	df	Sig. (2-tailed)	Mean Difference	Std. Error Difference	Lower	Upper
Attitude_9 Factors	Equal variances assumed	1.139	.286	21.606	537	.000	1.16091	.05373	1.05536	1.26646
	Equal variances not assumed			21.603	534.359	.000	1.16091	.05374	1.05535	1.26647

Table (3.1.10.3.8) The independent samples test for factor in questionnaire to determine the F values and significance.

Attitude Women CL (All factors) VS Men CL (All factors)

Table (3.1.10.3.9) Estimates for students in CL

Between-Subjects Factors

		Value Label	N
Gender	1.00	Women	279
	2.00	Men	288
Method	2.00	CL	567

Gender	Mean	Std. Error	95% Confidence Interval Lower Bound	Upper Bound
Women	4.105	.037	4.032	4.178
Men	3.285	.037	3.213	3.357

Table (3.1.10.3.10.) The subject factor based on gender and method

Descriptive Statistics

Dependent Variable: Attitude_9 Factors

Gender	Method	Mean	Std. Deviation	N
Women	CL	4.1053	.64930	279
	Total	4.1053	.64930	279
Men	CL	3.2847	.59377	288
	Total	3.2847	.59377	288
Total	CL	3.6885	.74461	567
	Total	3.6885	.74461	567

Table (3.1.10.3.11) The mean and standard deviation of students in CL

Univariate Tests

Dependent Variable: Attitude_9 Factors

	Sum of Squares	df	Mean Square	F	Sig.	Partial Eta Squared
Contrast	95.424	1	95.424	246.876	.000	.304
Error	218.388	565	.387			

Table (3.1.10.3.12) The F tests the effect of Gender. This test is based on the linearly independent pairwise comparisons among the estimated marginal means.

Mixed Gender Students

Factor 1 Feelings towards Biology

Traditional Learning

Case Processing Summary

		N	%
Cases	Valid	35	100.0
	Excluded[a]	0	.0
	Total	35	100.0

Women

		N	%
Cases	Valid	34	100.0
	Excluded[a]	0	.0
	Total	34	100.0

Men

Table (3.2.1.1.1) Listwise deletion based on all variables in the procedure.

Reliability Statistics

Cronbach's Alpha	Cronbach's Alpha Based on Standardized Items	N of Items
.863	.861	14

Women

Cronbach's Alpha	Cronbach's Alpha Based on Standardized Items	N of Items
.894	.894	14

Men

Table (3.2.1.1.2) The Cronbach's alpha for the factor is calculated based on the number of items.

Item Statistics

	Mean	Std. Deviation	N
Biology is very interesting to me.	2.86	.845	35
I have always enjoyed studying biology in school.	2.91	1.040	35
I am always under a terrible strain in a biology class.	2.66	.802	35
I feel a definite positive reaction to biology; it's enjoyable.	3.03	.923	35
Biology makes me feel secure, and at the same time it is stimulating.	3.09	.919	35
I feel at ease in biology and like it very much.	3.11	1.132	35

In general, I have a good feeling toward biology.	3.06	.906	35
I really like biology.	3.17	1.071	35
Biology is fascinating and fun.	3.14	1.061	35
When I hear the word biology, I have a feeling of dislike.	3.03	1.150	35
I approach biology with a feeling of hesitation.	3.14	.912	35
It makes me nervous to even think about doing a biology experiment.	2.71	1.073	35
Biology makes me feel uncomfortable, restless, irritable, and impatient.	2.94	1.056	35
I don't like biology, and it scares me to have to take it.	2.94	.998	35

	Women		
	Mean	Std. Deviation	N
Biology is very interesting to me.	2.68	.768	34
I have always enjoyed studying biology in school.	2.88	.808	34
I am always under a terrible strain in a biology class.	3.00	.696	34
I feel a definite positive reaction to biology; it's enjoyable.	2.91	.866	34
Biology makes me feel secure, and at the same time it is stimulating.	2.91	.830	34
I feel at ease in biology and like it very much.	2.85	.702	34
In general, I have a good feeling toward biology.	2.82	.869	34

I really like biology.	2.82	.834	34
Biology is fascinating and fun.	2.85	.744	34
When I hear the word biology, I have a feeling of dislike.	2.94	.919	34
I approach biology with a feeling of hesitation.	2.91	.712	34
It makes me nervous to even think about doing a biology experiment.	2.91	.668	34
Biology makes me feel uncomfortable, restless, irritable, and impatient.	2.91	.793	34
I don't like biology, and it scares me to have to take it.	2.74	.790	34

Men

Table (3.2.1.1.3) The statistics for the mean and standard deviation for each item in the factor in the questionnaire.

Item-Total Statistics

	Scale Mean if Item Deleted	Scale Variance if Item Deleted	Corrected Item-Total Correlation	Squared Multiple Correlation	Cronbach's Alpha if Item Deleted
Biology is very interesting to me.	38.94	64.820	.336	.454	.863
I have always enjoyed studying biology in school.	38.89	63.222	.351	.490	.863
I am always under a terrible strain in a biology class.	39.14	64.655	.373	.387	.861
I feel a definite positive reaction to biology; it's enjoyable.	38.77	64.182	.343	.396	.863
Biology makes me feel secure, and at the same time it is stimulating.	38.71	60.034	.648	.626	.847

I feel at ease in biology and like it very much.	38.69	57.987	.629	.517	.847
In general, I have a good feeling toward biology.	38.74	59.903	.669	.756	.846
I really like biology.	38.63	60.534	.506	.705	.855
Biology is fascinating and fun.	38.66	57.585	.708	.644	.843
When I hear the word biology, I have a feeling of dislike.	38.77	57.476	.648	.660	.846
I approach biology with a feeling of hesitation.	38.66	61.761	.524	.576	.854
It makes me nervous to even think about doing a biology experiment.	39.09	59.787	.553	.519	.852
Biology makes me feel uncomfortable, restless, irritable, and impatient.	38.86	60.773	.499	.477	.855
I don't like biology, and it scares me to have to take it.	38.86	62.479	.420	.527	.859

Women

	Scale Mean if Item Deleted	Scale Variance if Item Deleted	Corrected Item-Total Correlation	Squared Multiple Correlation	Cronbach's Alpha if Item Deleted
Biology is very interesting to me.	37.47	46.439	.412	.525	.894
I have always enjoyed studying biology in school.	37.26	43.837	.641	.725	.884
I am always under a terrible strain in a biology class.	37.15	46.190	.493	.584	.890
I feel a definite positive reaction to biology; it's enjoyable.	37.24	42.428	.724	.739	.880

Biology makes me feel secure, and at the same time it is stimulating.	37.24	45.034	.504	.481	.890
I feel at ease in biology and like it very much.	37.29	45.002	.621	.607	.885
In general, I have a good feeling toward biology.	37.32	44.104	.561	.518	.888
I really like biology.	37.32	43.559	.644	.677	.884
Biology is fascinating and fun.	37.29	44.456	.638	.589	.884
When I hear the word biology, I have a feeling of dislike.	37.21	43.381	.588	.543	.887
I approach biology with a feeling of hesitation.	37.24	48.125	.274	.503	.899
It makes me nervous to even think about doing a biology experiment.	37.24	45.640	.582	.576	.887
Biology makes me feel uncomfortable, restless, irritable, and impatient.	37.24	44.004	.638	.614	.884
I don't like biology, and it scares me to have to take it.	37.41	42.613	.785	.846	.878

Men

Table (3.2.1.1.4) The total statistics for each item in the factor in the questionnaire.

Collaborative Learning

Case Processing Summary

		N	%
Cases	Valid	36	100.0
	Excluded[a]	0	.0
	Total	36	100.0

Women

		N	%
Cases	Valid	36	100.0
	Excluded[a]	0	.0
	Total	36	100.0

Men

Table (3.2.1.2.1) Listwise deletion based on all variables in the procedure.

Reliability Statistics

Cronbach's Alpha	Cronbach's Alpha Based on Standardized Items	N of Items
.873	.869	14

Women

Cronbach's Alpha	Cronbach's Alpha Based on Standardized Items	N of Items
.868	.870	14

Men

Table (3.2.1.2.2) The Cronbach's alpha for factor is calculated based on the number of items

Item Statistics

	Mean	Std. Deviation	N

Biology is very interesting to me.	2.64	.639	36
I have always enjoyed studying biology in school.	2.61	.688	36
I am always under a terrible strain in a biology class.	2.61	.934	36
I feel a definite positive reaction to biology; it's enjoyable.	2.58	.996	36
Biology makes me feel secure, and at the same time it is stimulating.	2.83	.971	36
I feel at ease in biology and like it very much.	2.56	.998	36
In general, I have a good feeling toward biology.	2.50	.878	36
I really like biology.	2.50	.910	36
Biology is fascinating and fun.	2.31	.856	36
When I hear the word biology, I have a feeling of dislike.	2.39	.903	36
I approach biology with a feeling of hesitation.	2.44	.909	36
It makes me nervous to even think about doing a biology experiment.	2.56	.843	36
Biology makes me feel uncomfortable, restless, irritable, and impatient.	2.50	.811	36
I don't like biology, and it scares me to have to take it.	2.72	.914	36

	Women		
	Mean	Std. Deviation	N
Biology is very interesting to me.	4.00	.632	36

I have always enjoyed studying biology in school.	3.75	.649	36
I am always under a terrible strain in a biology class.	3.78	.681	36
I feel a definite positive reaction to biology; it's enjoyable.	3.89	.887	36
Biology makes me feel secure, and at the same time it is stimulating.	3.81	.668	36
I feel at ease in biology and like it very much.	4.00	.478	36
In general, I have a good feeling toward biology.	4.00	.793	36
I really like biology.	3.97	.609	36
Biology is fascinating and fun.	3.94	.630	36
When I hear the word biology, I have a feeling of dislike.	3.94	.860	36
I approach biology with a feeling of hesitation.	3.94	.715	36
It makes me nervous to even think about doing a biology experiment.	4.06	.583	36
Biology makes me feel uncomfortable, restless, irritable, and impatient.	4.17	.447	36
I don't like biology, and it scares me to have to take it.	3.83	.811	36

Men

Table (3.2.1.2.3) The statistics for the mean and standard deviation for each item in the factor in the questionnaire.

Item-Total Statistics

	Scale Mean if Item Deleted	Scale Variance if Item Deleted	Corrected Item-Total Correlation	Squared Multiple Correlation	Cronbach's Alpha if Item Deleted
Biology is very interesting to me.	33.11	54.330	.288	.440	.875
I have always enjoyed studying biology in school.	33.14	53.837	.311	.601	.874
I am always under a terrible strain in a biology class.	33.14	51.094	.410	.424	.871
I feel a definite positive reaction to biology; it's enjoyable.	33.17	47.400	.660	.672	.858
Biology makes me feel secure, and at the same time it is stimulating.	32.92	48.879	.562	.642	.863
I feel at ease in biology and like it very much.	33.19	46.275	.749	.707	.852
In general, I have a good feeling toward biology.	33.25	51.507	.410	.447	.871
I really like biology.	33.25	49.393	.565	.739	.863
Biology is fascinating and fun.	33.44	48.711	.670	.645	.858
When I hear the word biology, I have a feeling of dislike.	33.36	48.352	.659	.690	.858
I approach biology with a feeling of hesitation.	33.31	51.247	.413	.467	.871
It makes me nervous to even think about doing a biology experiment.	33.19	48.447	.706	.745	.856
Biology makes me feel uncomfortable, restless, irritable, and impatient.	33.25	51.793	.429	.387	.870
I don't like biology, and it scares me to have to take it.	33.03	48.999	.595	.533	.862

Women

	Scale Mean if Item Deleted	Scale Variance if Item Deleted	Corrected Item-Total Correlation	Squared Multiple Correlation	Cronbach's Alpha if Item Deleted
Biology is very interesting to me.	51.08	28.993	.663	.678	.852
I have always enjoyed studying biology in school.	51.33	30.571	.406	.621	.865
I am always under a terrible strain in a biology class.	51.31	31.590	.242	.517	.873
I feel a definite positive reaction to biology; it's enjoyable.	51.19	27.018	.661	.766	.851
Biology makes me feel secure, and at the same time it is stimulating.	51.28	29.121	.602	.649	.855
I feel at ease in biology and like it very much.	51.08	30.593	.583	.661	.858
In general, I have a good feeling toward biology.	51.08	28.193	.604	.703	.854
I really like biology.	51.11	29.930	.541	.728	.858
Biology is fascinating and fun.	51.14	30.809	.386	.497	.866
When I hear the word biology, I have a feeling of dislike.	51.14	28.009	.567	.603	.857
I approach biology with a feeling of hesitation.	51.14	28.694	.614	.603	.854
It makes me nervous to even think about doing a biology experiment.	51.03	30.942	.405	.518	.864
Biology makes me feel uncomfortable, restless, irritable, and impatient.	50.92	31.107	.521	.660	.861
I don't like biology, and it scares me to have to take it.	51.25	27.907	.624	.785	.853

Men

Table (3.2.1.2.4) The total statistics for each item in the factor in the questionnaire.

TL vs CL

Group Statistics

	Method	N	Mean	Std. Deviation	Std. Error Mean
Feeling toward biology	TL	35	3.0054	.58255	.09847
	CL	36	2.5689	.50949	.08491

Women

	Method	N	Mean	Std. Deviation	Std. Error Mean
Feeling toward biology	TL	34	2.8956	.50381	.08640
	CL	36	3.9111	.33015	.05502

Men

Table (3.2.1.3.1) The group statistics for each item in the factor questionnaire with method.

Independent Samples Test

		Levene's Test for Equality of Variances		t-test for Equality of Means						
									95% Confidence Interval of the Difference	
		F	Sig.	t	df	Sig. (2-tailed)	Mean Difference	Std. Error Difference	Lower	Upper
Feeling toward biology	Equal variances assumed	.266	.608	3.364	69	.001	.43654	.12978	.17764	.69544
	Equal variances not assumed			3.357	67.245	.001	.43654	.13003	.17703	.69605

Women

Independent Samples Test

		Levene's Test for Equality of Variances		t-test for Equality of Means						
									95% Confidence Interval of the Difference	
		F	Sig.	t	df	Sig. (2-tailed)	Mean Difference	Std. Error Difference	Lower	Upper
Feeling toward biology	Equal variances assumed	1.614	.208	-10.029	68	.000	-1.01552	.10126	-1.21758	-.81347
	Equal variances not assumed			-9.914	56.442	.000	-1.01552	.10244	-1.22069	-.81035

Men

Table (3.2.1.3.2) The independent samples test for factor questionnaire to determine the F values and significance.

Factor 2 General Interest

Traditional Learning

Case Processing Summary

		N	%
Cases	Valid	35	100.0
	Excluded[a]	0	.0
	Total	35	100.0

Women

		N	%
Cases	Valid	34	100.0
	Excluded[a]	0	.0
	Total	34	100.0

Men

Table (3.2.2.1.1) Listwise deletion based on all variables in the procedure.

Reliability Statistics

Cronbach's Alpha	Cronbach's Alpha Based on Standardized Items	N of Items
.815	.813	5

Women

Cronbach's Alpha	Cronbach's Alpha Based on Standardized Items	N of Items
.804	.800	5

Men

Table (3.2.2.1.2) The Cronbach's alpha for the factor is calculated based on the number of items.

Item Statistics

	Mean	Std. Deviation	N
I like watching biology related TV.	3.40	.775	35
biology is my favorite subject in school.	3.37	.808	35
I like reading about famous biologiest	3.26	1.010	35
I find what we learn in my biology class interesting.	3.60	.736	35
I would enjoy working in a biology lab.	3.31	.900	35

Women

Item Statistics

	Mean	Std. Deviation	N
I like watching biology related TV.	3.00	1.073	34
biology is my favorite subject in school.	2.65	1.178	34
I like reading about famous biologiest	2.94	1.043	34
I find what we learn in my biology class interesting.	2.85	.989	34
I would enjoy working in a biology lab.	2.62	1.129	34

Men

Table (3.2.2.1.3) The statistics for the mean and standard deviation for each item in the factor in the questionnaire.

Item-Total Statistics

	Scale Mean if Item Deleted	Scale Variance if Item Deleted	Corrected Item-Total Correlation	Squared Multiple Correlation	Cronbach's Alpha if Item Deleted
I like watching biology related TV.	13.54	7.197	.628	.580	.773
biology is my favorite subject in school.	13.57	7.311	.560	.440	.791
I like reading about famous biologiest	13.69	6.045	.673	.515	.759
I find what we learn in my biology class interesting.	13.34	7.938	.465	.237	.816
I would enjoy working in a biology lab.	13.63	6.358	.714	.591	.743

Women

	Scale Mean if Item Deleted	Scale Variance if Item Deleted	Corrected Item-Total Correlation	Squared Multiple Correlation	Cronbach's Alpha if Item Deleted
I like watching biology related TV.	11.06	11.330	.554	.320	.777
biology is my favorite subject in school.	11.41	9.704	.735	.594	.716
I like reading about famous biologiest	11.12	12.531	.388	.353	.824
I find what we learn in my biology class interesting.	11.21	11.865	.534	.457	.783
I would enjoy working in a biology lab.	11.44	9.890	.749	.598	.713

Men

Table (3.2.2.1.4) The total statistics for each item in the factor in the questionnaire.

Collaborative Learning

Case Processing Summary

		N	%
Cases	Valid	36	100.0
	Excluded[a]	0	.0
	Total	36	100.0

Women

		N	%
Cases	Valid	36	100.0
	Excluded[a]	0	.0
	Total	36	100.0

Men

Table (3.2.2.2.1) Listwise deletion based on all variables in the procedure.

Reliability Statistics

Cronbach's Alpha	Cronbach's Alpha Based on Standardized Items	N of Items
.776	.770	5

Women

Cronbach's Alpha	Cronbach's Alpha Based on Standardized Items	N of Items
.874	.885	5

Men

Table (3.2.2.2.2) The Cronbach's alpha for the factor is calculated based on the number of items.

Item Statistics

	Mean	Std. Deviation	N
I like watching biology related TV.	3.06	.791	36
biology is my favorite subject in school.	3.25	.937	36
I like reading about famous biologiest	3.19	1.009	36
I find what we learn in my biology class interesting.	3.61	.838	36
I would enjoy working in a biology lab.	3.33	.986	36

Women

	Mean	Std. Deviation	N
I like watching biology related TV.	3.89	.950	36
biology is my favorite subject in school.	3.97	.506	36
I like reading about famous biologiest	3.92	.770	36
I find what we learn in my biology class interesting.	3.97	.560	36
I would enjoy working in a biology lab.	3.92	.692	36

Men

Table (3.2.2.1.3) The statistics for the mean and standard deviation for each item in the factor in the questionnaire.

Item-Total Statistics

	Scale Mean if Item Deleted	Scale Variance if Item Deleted	Corrected Item-Total Correlation	Squared Multiple Correlation	Cronbach's Alpha if Item Deleted
I like watching biology related TV.	13.39	8.073	.524	.398	.744
biology is my favorite subject in school.	13.19	6.504	.768	.618	.653
I like reading about famous biologiest	13.25	7.164	.532	.332	.742
I find what we learn in my biology class interesting.	12.83	9.057	.257	.263	.819
I would enjoy working in a biology lab.	13.11	6.559	.698	.573	.678

Women

	Scale Mean if Item Deleted	Scale Variance if Item Deleted	Corrected Item-Total Correlation	Squared Multiple Correlation	Cronbach's Alpha if Item Deleted
I like watching biology related TV.	15.78	4.521	.752	.615	.849
biology is my favorite subject in school.	15.69	6.618	.607	.370	.873
I like reading about famous biologiest	15.75	5.336	.711	.632	.846
I find what we learn in my biology class interesting.	15.69	6.161	.713	.540	.851
I would enjoy working in a biology lab.	15.75	5.279	.849	.754	.812

Men

Table (3.2.2.2.4) The total statistics for each item in the factor in the questionnaire.

TL vs CL

Group Statistics

	Method	N	Mean	Std. Deviation	Std. Error Mean
General interest	TL	35	3.4260	.61568	.10407
	CL	36	3.3017	.65949	.10991

Women

	Method	N	Mean	Std. Deviation	Std. Error Mean
General interest	TL	34	2.7850	.77899	.13360
	CL	36	3.9017	.57367	.09561

Men

Table (3.2.2.3.1) The group statistics for each item in the factors questionnaire with method.

Independent Samples Test

		Levene's Test for Equality of Variances		t-test for Equality of Means						
									95% Confidence Interval of the Difference	
		F	Sig.	t	df	Sig. (2-tailed)	Mean Difference	Std. Error Difference	Lower	Upper
Feeling toward biology	Equal variances assumed	.428	.515	.821	69	.415	.12433	.15151	-.17793	.42660
	Equal variances not assumed			.821	68.889	.414	.12433	.15137	-.17764	.42631

Women

Independent Samples Test

		Levene's Test for Equality of Variances		t-test for Equality of Means						
									95% Confidence Interval of the Difference	
		F	Sig.	t	df	Sig. (2-tailed)	Mean Difference	Std. Error Difference	Lower	Upper
Feeling toward biology	Equal variances assumed	4.545	.037	-6.856	68	.000	-1.11667	.16288	-1.44168	-.79165
	Equal variances not assumed			-6.797	60.498	.000	-1.11667	.16428	-1.44523	-.78811

Men

Table (3.2.2.3.2) The independent samples test for feelings towards biology questionnaire to determine the F values and significance.

Factor 3 Motivation Towards Learning

Traditional Learning

Case Processing Summary

		N	%
Cases	Valid	35	100.0
	Excluded[a]	0	.0
	Total	35	100.0

Women

		N	%
Cases	Valid	34	100.0
	Excluded[a]	0	.0
	Total	34	100.0

Men

Table (3.2.3.1.1) Listwise deletion based on all variables in the procedure

Reliability Statistics

Cronbach's Alpha	Cronbach's Alpha Based on Standardized Items	N of Items
.775	.769	10

Women

Cronbach's Alpha	Cronbach's Alpha Based on Standardized Items	N of Items
.880	.879	10

Men

Table (3.2.3.1.2) The Cronbach's alpha for the factor is calculated based on the number of items.

Item Statistics

	Mean	Std. Deviation	N
I will ask my teacher for an explanation if I do not understand the science topic.	3.1714	.70651	35
I will look for an explanation in the textbook if I do not understand the science topic.	3.3143	.75815	35
I care about completing assignments in this class.	3.1429	.55002	35
Getting a good grade in biology is important to me.	3.3714	.77024	35
I am interested in understanding the teacher in this class.	3.1429	.60112	35
The biology I learn is relevant to my life.	3.2857	.66737	35
Learning biology is interesting.	3.1714	.56806	35
Learning biology makes my life more meaningful.	3.2286	.68966	35
I am curious about discoveries in biology.	3.4571	.56061	35
I enjoy learning biology	2.9714	.61767	35

Women

	Mean	Std. Deviation	N
I will ask my teacher for an explanation if I do not understand the science topic.	3.0000	.69631	34

I will look for an explanation in the textbook if I do not understand the science topic.	3.2059	.80827	34
I care about completing assignments in this class.	3.2353	.85489	34
Getting a good grade in biology is important to me.	3.0000	.85280	34
I am interested in understanding the teacher in this class.	3.1765	.71650	34
The biology I learn is relevant to my life.	3.0882	.75348	34
Learning biology is interesting.	3.0294	.71712	34
Learning biology makes my life more meaningful.	3.3529	.69117	34
I am curious about discoveries in biology.	3.0882	.71213	34
I enjoy learning biology	3.1471	.74396	34

Men

Table (3.2.3.1.3) The statistics for the mean and standard deviation for each item in the factor in the questionnaire.

Item-Total Statistics

	Scale Mean if Item Deleted	Scale Variance if Item Deleted	Corrected Item-Total Correlation	Squared Multiple Correlation	Cronbach's Alpha if Item Deleted
I will ask my teacher for an explanation if I do not understand the science topic.	29.0857	11.139	.530	.474	.744
I will look for an explanation in the textbook if I do not understand the science topic.	28.9429	11.291	.446	.687	.757

I care about completing assignments in this class.	29.1143	12.398	.371	.385	.765
Getting a good grade in biology is important to me.	28.8857	10.692	.566	.539	.738
I am interested in understanding the teacher in this class.	29.1143	11.810	.476	.475	.753
The biology I learn is relevant to my life.	28.9714	11.852	.401	.525	.762
Learning biology is interesting.	29.0857	12.081	.439	.366	.757
Learning biology makes my life more meaningful.	29.0286	10.911	.604	.554	.734
I am curious about discoveries in biology.	28.8000	13.576	.060	.190	.797
I enjoy learning biology	29.2857	11.622	.507	.584	.749

Women

	Scale Mean if Item Deleted	Scale Variance if Item Deleted	Corrected Item-Total Correlation	Squared Multiple Correlation	Cronbach's Alpha if Item Deleted
I will ask my teacher for an explanation if I do not understand the science topic.	28.3235	23.680	.492	.498	.876
I will look for an explanation in the textbook if I do not understand the science topic.	28.1176	21.925	.650	.740	.865
I care about completing assignments in this class.	28.0882	22.204	.566	.661	.872
Getting a good grade in biology is important to me.	28.3235	20.953	.745	.671	.856

I am interested in understanding the teacher in this class.	28.1471	23.038	.574	.676	.870
The biology I learn is relevant to my life.	28.2353	22.307	.650	.814	.865
Learning biology is interesting.	28.2941	23.426	.513	.366	.875
Learning biology makes my life more meaningful.	27.9706	23.605	.509	.529	.875
I am curious about discoveries in biology.	28.2353	22.307	.696	.618	.862
I enjoy learning biology	28.1765	22.271	.666	.742	.864

Men

Table (3.2.3.1.4) The total statistics for each item in the factor in the questionnaire.

Collaborative Learning

Case Processing Summary

		N	%
Cases	Valid	36	100.0
	Excluded[a]	0	.0
	Total	36	100.0

Women

Cases	Valid	36	100.0
	Excluded[a]	0	.0
	Total	36	100.0

Men

Table (3.2.3.2.1) Listwise deletion based on all variables in the procedure

Reliability Statistics

Cronbach's Alpha	Cronbach's Alpha Based on Standardized Items	N of Items
.817	.803	10

Women

Cronbach's Alpha	Cronbach's Alpha Based on Standardized Items	N of Items
.815	.827	10

Men

Table (3.2.3.2.2) The Cronbach's alpha for the factor is calculated based on the number of items.

Item Statistics

	Mean	Std. Deviation	N
I will ask my teacher for an explanation if I do not understand the science topic.	2.9722	.60880	36
I will look for an explanation in the textbook if I do not understand the science topic.	3.1667	.73679	36
I care about completing assignments in this class.	2.9722	.60880	36
Getting a good grade in biology is important to me.	3.1389	.89929	36
I am interested in understanding the teacher in this class.	3.1667	.73679	36
The biology I learn is relevant to my life.	3.1111	.78478	36
Learning biology is interesting.	3.1667	.50709	36
Learning biology makes my life more meaningful.	3.1389	.59295	36
I am curious about discoveries in biology.	3.0000	.63246	36
I enjoy learning biology	3.0833	.84092	36

Women

	Mean	Std. Deviation	N
I will ask my teacher for an explanation if I do not understand the science topic.	3.5556	.69465	36

I will look for an explanation in the textbook if I do not understand the science topic.	3.8333	.60945	36
I care about completing assignments in this class.	3.6944	.88864	36
Getting a good grade in biology is important to me.	3.7778	.59094	36
I am interested in understanding the teacher in this class.	3.7778	.79682	36
The biology I learn is relevant to my life.	3.5833	1.05221	36
Learning biology is interesting.	3.6944	.88864	36
Learning biology makes my life more meaningful.	3.7778	.95950	36
I am curious about discoveries in biology.	3.8056	.98036	36
I enjoy learning biology	3.9722	.84468	36

Men

Table (3.2.3.2.3) The statistics for the mean and standard deviation for each item in the factor in the questionnaire.

Item-Total Statistics

	Scale Mean if Item Deleted	Scale Variance if Item Deleted	Corrected Item-Total Correlation	Squared Multiple Correlation	Cronbach's Alpha if Item Deleted
I will ask my teacher for an explanation if I do not understand the science topic.	27.9444	16.625	.356	.598	.814

I will look for an explanation in the textbook if I do not understand the science topic.	27.7500	14.307	.702	.686	.777
I care about completing assignments in this class.	27.9444	16.111	.467	.586	.804
Getting a good grade in biology is important to me.	27.7778	13.435	.686	.674	.777
I am interested in understanding the teacher in this class.	27.7500	15.450	.478	.630	.803
The biology I learn is relevant to my life.	27.8056	13.875	.731	.725	.772
Learning biology is interesting.	27.7500	17.279	.291	.274	.818
Learning biology makes my life more meaningful.	27.7778	17.549	.174	.207	.829
I am curious about discoveries in biology.	27.9167	16.307	.403	.578	.810
I enjoy learning biology	27.8333	14.143	.619	.685	.786

Women

	Scale Mean if Item Deleted	Scale Variance if Item Deleted	Corrected Item-Total Correlation	Squared Multiple Correlation	Cronbach's Alpha if Item Deleted
I will ask my teacher for an explanation if I do not understand the science topic.	33.9167	23.164	.459	.403	.803
I will look for an explanation in the textbook if I do not understand the science topic.	33.6389	22.809	.607	.618	.792
I care about completing assignments in this class.	33.7778	23.549	.275	.405	.823

Getting a good grade in biology is important to me.	33.6944	22.675	.655	.642	.789
I am interested in understanding the teacher in this class.	33.6944	24.390	.215	.262	.826
The biology I learn is relevant to my life.	33.8889	21.244	.450	.504	.807
Learning biology is interesting.	33.7778	20.806	.631	.664	.783
Learning biology makes my life more meaningful.	33.6944	20.961	.550	.643	.792
I am curious about discoveries in biology.	33.6667	20.971	.532	.582	.795
I enjoy learning biology	33.5000	20.257	.755	.745	.769

Men

Table (3.2.3.2.4) The total statistics for each item in the factor in the questionnaire.

TL vs CL

Group Statistics

	Method	N	Mean	Std. Deviation	Std. Error Mean
Motivation Towards Learning Biology	TL	35	3.2211	.38423	.06495
	CL	36	3.1031	.41719	.06953

Women

	Method	N	Mean	Std. Deviation	Std. Error Mean
Motivation Towards Learning Biology	TL	34	3.0812	.41199	.07066
	CL	36	3.7519	.51445	.08574

Men

Table (3.2.3.3.1) The group statistics for each item in the factors questionnaire with method.

Independent Samples Test

		Levene's Test for Equality of Variances		t-test for Equality of Means						
									95% Confidence Interval of the Difference	
		F	Sig.	t	df	Sig. (2-tailed)	Mean Difference	Std. Error Difference	Lower	Upper
Feeling toward biology	Equal variances assumed	.075	.785	1.240	69	.219	.11809	.09526	-.07195	.30812
	Equal variances not assumed			1.241	68.802	.219	.11809	.09515	-.07173	.30791

Women

Independent Samples Test

		Levene's Test for Equality of Variances		t-test for Equality of Means						
									95% Confidence Interval of the Difference	
		F	Sig.	t	df	Sig. (2-tailed)	Mean Difference	Std. Error Difference	Lower	Upper
Feeling toward biology	Equal variances assumed	.004	.949	-5.999	68	.000	-.67077	.11181	-.89388	-.44766
	Equal variances not assumed			-6.037	66.265	.000	-.67077	.11110	-.89258	-.44896

Men

Table (3.2.3.3.2) The independent samples test for feelings towards biology questionnaire to determine the F values and significance.

Factor 4 Benefit and Utility of biology

Traditional Learning

Case Processing Summary

		N	%
Cases	Valid	35	100.0
	Excluded[a]	0	.0
	Total	35	100.0

Women

		N	%
Cases	Valid	34	100.0
	Excluded[a]	0	.0
	Total	34	100.0

Men

Table (3.2.4.1.1) Listwise deletion based on all variables in the procedure

Cronbach's Alpha	Cronbach's Alpha Based on Standardized Items	N of Items
.820	.820	5

Women

Cronbach's Alpha	Cronbach's Alpha Based on Standardized Items	N of Items
.780	.782	5

Men

Table (3.2.4.1.2) The Cronbach's alpha for the factor is calculated based on the number of items.

Item Statistics

	Mean	Std. Deviation	N
I use the biology that I learn in school in my life.	3.26	.817	35
What I learn in my biology class helps me understand how things work in life.	3.31	.758	35
Learning biology makes me curious about things that I observe in my life.	3.31	.832	35
What we learn in biology class helps me to understand how biology affects my life.	3.31	.718	35
Learning biology helps me to make wiser decisions about my lifestyle and health.	3.49	.919	35

Women

	Mean	Std. Deviation	N
I use the biology that I learn in school in my life.	2.24	.741	34
What I learn in my biology class helps me understand how things work in life.	2.38	.739	34
Learning biology makes me curious about things that I observe in my life.	2.32	.727	34
What we learn in biology class helps me to understand how biology affects my life.	2.26	.710	34

Learning biology helps me to make wiser decisions about my lifestyle and health.	2.32	.768	34

Men

Table (3.2.4.1.3) The statistics for the mean and standard deviation for each item in the factor in the questionnaire.

Item-Total Statistics

	Scale Mean if Item Deleted	Scale Variance if Item Deleted	Corrected Item-Total Correlation	Squared Multiple Correlation	Cronbach's Alpha if Item Deleted
I use the biology that I learn in school in my life.	13.43	6.370	.616	.445	.783
What I learn in my biology class helps me understand how things work in life.	13.37	6.476	.654	.493	.773
Learning biology makes me curious about things that I observe in my life.	13.37	6.358	.602	.475	.787
What we learn in biology class helps me to understand how biology affects my life.	13.37	7.005	.540	.331	.804
Learning biology helps me to make wiser decisions about my lifestyle and health.	13.20	5.812	.658	.537	.771

Women

	Scale Mean if Item Deleted	Scale Variance if Item Deleted	Corrected Item-Total Correlation	Squared Multiple Correlation	Cronbach's Alpha if Item Deleted
I use the biology that I learn in school in my life.	9.29	4.699	.616	.421	.718
What I learn in my biology class helps me understand how things work in life.	9.15	5.463	.352	.252	.804
Learning biology makes me curious about things that I observe in my life.	9.21	4.775	.605	.541	.722
What we learn in biology class helps me to understand how biology affects my life.	9.26	4.625	.687	.575	.695
Learning biology helps me to make wiser decisions about my lifestyle and health.	9.21	4.835	.534	.323	.747

Men

Table (3.2.4.1.4) The total statistics for each item in the factor in the questionnaire.

Collaborative Learning

Case Processing Summary

		N	%
Cases	Valid	36	100.0
	Excluded[a]	0	.0
	Total	36	100.0

Women

Cases	Valid	36	100.0
	Excluded[a]	0	.0
	Total	36	100.0

Men

Table (3.2.4.2.1) Listwise deletion based on all variables in the procedure

Reliability Statistics

Cronbach's Alpha	Cronbach's Alpha Based on Standardized Items	N of Items
.828	.831	5

Women

Cronbach's Alpha	Cronbach's Alpha Based on Standardized Items	N of Items
.871	.865	5

Men

Table (3.2.4.2.2) The Cronbach's alpha for the factor is calculated based on the number of items.

Item Statistics

	Mean	Std. Deviation	N
I use the biology that I learn in school in my life.	2.81	.668	36
What I learn in my biology class helps me understand how things work in life.	2.92	.732	36
Learning biology makes me curious about things that I observe in my life.	3.00	.756	36
What we learn in biology class helps me to understand how biology affects my life.	2.89	.785	36
Learning biology helps me to make wiser decisions about my lifestyle and health.	2.89	.820	36

Women

	Mean	Std. Deviation	N
I use the biology that I learn in school in my life.	3.67	.676	36
What I learn in my biology class helps me understand how things work in life.	3.50	.845	36
Learning biology makes me curious about things that I observe in my life.	3.72	.882	36
What we learn in biology class helps me to understand how biology affects my life.	3.78	.866	36

Learning biology helps me to make wiser decisions about my lifestyle and health.	3.47	.878	36

Men

Table (3.2.4.2.3) The statistics for the mean and standard deviation for each item in the factor in the questionnaire.

Item-Total Statistics

	Scale Mean if Item Deleted	Scale Variance if Item Deleted	Corrected Item-Total Correlation	Squared Multiple Correlation	Cronbach's Alpha if Item Deleted
I use the biology that I learn in school in my life.	11.69	5.818	.671	.667	.784
What I learn in my biology class helps me understand how things work in life.	11.58	5.393	.735	.609	.763
Learning biology makes me curious about things that I observe in my life.	11.50	5.857	.547	.354	.816
What we learn in biology class helps me to understand how biology affects my life.	11.61	6.016	.467	.265	.840
Learning biology helps me to make wiser decisions about my lifestyle and health.	11.61	5.044	.736	.633	.760

Women

	Scale Mean if Item Deleted	Scale Variance if Item Deleted	Corrected Item-Total Correlation	Squared Multiple Correlation	Cronbach's Alpha if Item Deleted
I use the biology that I learn in school in my life.	14.47	9.228	.427	.231	.898
What I learn in my biology class helps me understand how things work in life.	14.64	7.209	.774	.611	.824
Learning biology makes me curious about things that I observe in my life.	14.42	7.107	.755	.710	.828
What we learn in biology class helps me to understand how biology affects my life.	14.36	7.037	.795	.748	.818
Learning biology helps me to make wiser decisions about my lifestyle and health.	14.67	7.200	.736	.597	.834

Men

Table (3.2.4.2.4) The total statistics for each item in the factor in the questionnaire.

TL vs CL

Group Statistics

	Method	N	Mean	Std. Deviation	Std. Error Mean
Benefit and Utility of biology	TL	35	3.3117	.58087	.09819
	CL	36	2.9028	.57120	.09520

Women

	Method	N	Mean	Std. Deviation	Std. Error Mean
Benefit and Utility of biology	TL	34	2.2968	.53665	.09203
	CL	36	3.6511	.65179	.10863

Men

Table (3.2.4.3.1) The group statistics for each item in the factors Questionnaire with method.

Independent Samples Test

		Levene's Test for Equality of Variances		t-test for Equality of Means					95% Confidence Interval of the Difference	
		F	Sig.	t	df	Sig. (2-tailed)	Mean Difference	Std. Error Difference	Lower	Upper
Benefit and Utility of biology	Equal variances assumed	.130	.720	2.991	69	.004	.40894	.13673	.13617	.68170
	Equal variances not assumed			2.990	68.858	.004	.40894	.13676	.13610	.68178

Women

Independent Samples Test

		Levene's Test for Equality of Variances		t-test for Equality of Means					95% Confidence Interval of the Difference	
		F	Sig.	t	df	Sig. (2-tailed)	Mean Difference	Std. Error Difference	Lower	Upper
Benefit and Utility of biology	Equal variances assumed	.007	.934	-9.460	68	.000	-1.35435	.14317	-1.64004	-1.06865
	Equal variances not assumed			-9.512	66.784	.000	-1.35435	.14238	-1.63855	-1.07014

Men

Table (3.2.4.3.2) The independent samples test for feelings towards biology questionnaire to determine the F values and significance.

Factor 5 Career Motivation

Traditional Learning

Case Processing Summary

		N	%
Cases	Valid	35	100.0
	Excluded[a]	0	.0
	Total	35	100.0

Women

		N	%
Cases	Valid	34	100.0
	Excluded[a]	0	.0
	Total	34	100.0

Men

Table (3.2.5.1.1) Listwise deletion based on all variables in the procedure

Reliability Statistics

Cronbach's Alpha	Cronbach's Alpha Based on Standardized Items	N of Items
.810	.803	5

Women

Cronbach's Alpha	Cronbach's Alpha Based on Standardized Items	N of Items
.849	.854	5

Men

Table (3.2.5.1.2) The Cronbach's alpha for the factor is calculated based on the number of items.

Item Statistics

	Mean	Std. Deviation	N
Learning biology will help me get a good job.	2.91	.562	35
Knowing biology will give me a career advantage.	2.94	.725	35
Understanding biology will benefit me in my career.	2.97	.785	35
My career will involve science.	2.86	.845	35
I will use biology problem-solving skills in my career	3.00	.686	35

Women

	Mean	Std. Deviation	N
Learning biology will help me get a good job.	2.35	.597	34
Knowing biology will give me a career advantage.	2.38	.817	34
Understanding biology will benefit me in my career.	2.38	.817	34
My career will involve science.	2.41	.743	34
I will use biology problem-solving skills in my career	2.41	.821	34

Men

Table (3.2.5.1.3) The statistics for the mean and standard deviation for each item in the factor in the questionnaire.

Item-Total Statistics

	Scale Mean if Item Deleted	Scale Variance if Item Deleted	Corrected Item-Total Correlation	Squared Multiple Correlation	Cronbach's Alpha if Item Deleted
Learning biology will help me get a good job.	11.77	6.182	.364	.269	.831
Knowing biology will give me a career advantage.	11.74	4.903	.650	.491	.757
Understanding biology will benefit me in my career.	11.71	4.445	.741	.562	.725
My career will involve science.	11.83	4.382	.684	.601	.746
I will use biology problem-solving skills in my career	11.69	5.281	.560	.408	.785

Women

	Scale Mean if Item Deleted	Scale Variance if Item Deleted	Corrected Item-Total Correlation	Squared Multiple Correlation	Cronbach's Alpha if Item Deleted
Learning biology will help me get a good job.	9.59	6.674	.667	.474	.822
Knowing biology will give me a career advantage.	9.56	5.648	.714	.524	.802
Understanding biology will benefit me in my career.	9.56	6.072	.583	.433	.839
My career will involve science.	9.53	5.954	.712	.513	.804
I will use biology problem-solving skills in my career	9.53	5.832	.651	.537	.820

Men

Table (3.2.5.1.4) The total statistics for each item in the factor in the questionnaire.

Collaborative Learning

Case Processing Summary

		N	%
Cases	Valid	36	100.0
	Excluded[a]	0	.0
	Total	36	100.0

Women

Cases	Valid	36	100.0
	Excluded[a]	0	.0
	Total	36	100.0

Men

Table (3.2.5.2.1) Listwise deletion based on all variables in the procedure

Reliability Statistics

Cronbach's Alpha	Cronbach's Alpha Based on Standardized Items	N of Items
.826	.828	5

Women

Cronbach's Alpha	Cronbach's Alpha Based on Standardized Items	N of Items
.864	.862	5

Men

Table (3.2.5.2.2) The Cronbach’s alpha for the factor is calculated based on the number of items.

Item Statistics

	Mean	Std. Deviation	N
I use the biology that I learn in school in my life.	2.58	.841	36
What I learn in my biology class helps me understand how things work in life.	2.56	.695	36
Learning biology makes me curious about things that I observe in my life.	2.53	.810	36
What we learn in biology class helps me to understand how biology affects my life.	2.39	.803	36
Learning biology helps me to make wiser decisions about my lifestyle and health.	2.56	.843	36

Women

	Mean	Std. Deviation	N
I use the biology that I learn in school in my life.	3.81	.710	36
What I learn in my biology class helps me understand how things work in life.	3.97	.696	36
Learning biology makes me curious about things that I observe in my life.	3.89	.622	36
What we learn in biology class helps me to understand how biology affects my life.	4.00	.586	36

Learning biology helps me to make wiser decisions about my lifestyle and health.	3.94	.715	36

Men

Table (3.2.5.2.3) The statistics for the mean and standard deviation for each item in the factor in the questionnaire.

Item-Total Statistics

	Scale Mean if Item Deleted	Scale Variance if Item Deleted	Corrected Item-Total Correlation	Squared Multiple Correlation	Cronbach's Alpha if Item Deleted
I use the biology that I learn in school in my life.	10.03	5.913	.690	.485	.771
What I learn in my biology class helps me understand how things work in life.	10.06	6.625	.653	.452	.786
Learning biology makes me curious about things that I observe in my life.	10.08	6.021	.696	.491	.770
What we learn in biology class helps me to understand how biology affects my life.	10.22	6.863	.460	.266	.837
Learning biology helps me to make wiser decisions about my lifestyle and health.	10.06	6.111	.629	.477	.790

Women

	Scale Mean if Item Deleted	Scale Variance if Item Deleted	Corrected Item-Total Correlation	Squared Multiple Correlation	Cronbach's Alpha if Item Deleted

I use the biology that I learn in school in my life.	15.81	4.390	.781	.632	.809
What I learn in my biology class helps me understand how things work in life.	15.64	4.466	.770	.700	.812
Learning biology makes me curious about things that I observe in my life.	15.72	5.578	.426	.246	.893
What we learn in biology class helps me to understand how biology affects my life.	15.61	5.044	.695	.499	.834
Learning biology helps me to make wiser decisions about my lifestyle and health.	15.67	4.400	.768	.694	.812

Men

Table (3.2.5.2.4) The total statistics for each item in the factor in the questionnaire.

TL vs CL

Group Statistics

	Method	N	Mean	Std. Deviation	Std. Error Mean
Career Motivation	TL	35	2.9123	.53767	.09088
	CL	36	2.5128	.60117	.10020

Women

	Method	N	Mean	Std. Deviation	Std. Error Mean
Career Motivation	TL	34	2.3897	.57921	.09933
	CL	36	3.9133	.53314	.08886

Men

Table (3.2.5.3.1) The group statistics for each item in the factors questionnaire with method.

Independent Samples Test

		Levene's Test for Equality of Variances		t-test for Equality of Means						
									95% Confidence Interval of the Difference	
		F	Sig.	t	df	Sig. (2-tailed)	Mean Difference	Std. Error Difference	Lower	Upper
Benefit and Utility of biology	Equal variances assumed	.700	.406	2.949	69	.004	.39951	.13549	.12922	.66980
	Equal variances not assumed			2.953	68.531	.004	.39951	.13527	.12961	.66940

Women

Independent Samples Test

		Levene's Test for Equality of Variances		t-test for Equality of Means						
									95% Confidence Interval of the Difference	
		F	Sig.	t	df	Sig. (2-tailed)	Mean Difference	Std. Error Difference	Lower	Upper
Benefit and Utility of biology	Equal variances assumed	.220	.641	-11.459	68	.000	-1.52363	.13296	-1.78894	-1.25831
	Equal variances not assumed			-11.432	66.684	.000	-1.52363	.13328	-1.78967	-1.25758

Men

Table (3.2.5.3.2) The independent samples test for the factor in the questionnaire to determine the F values and significance.

Factor 6 Self-Efficacy in Biology Learning

Traditional Learning

Case Processing Summary

		N	%
Cases	Valid	35	100.0
	Excluded[a]	0	.0
	Total	35	100.0

Women

		N	%
Cases	Valid	34	100.0
	Excluded[a]	0	.0
	Total	34	100.0

Men

Table (3.2.6.1.1) Listwise deletion based on all variables in the procedure

Reliability Statistics

Cronbach's Alpha	Cronbach's Alpha Based on Standardized Items	N of Items
.782	.780	8

Women

Cronbach's Alpha	Cronbach's Alpha Based on Standardized Items	N of Items
.858	.856	8

Men

Table (3.2.6.1.2) The Cronbach's alpha for the factor is calculated based on the number of items.

Item Statistics

	Mean	Std. Deviation	N
If I study hard I can do well in biology	2.8000	.58410	35
I believe biology is too easy for me to learn	2.6857	.58266	35
The idea of taking biology makes me excited.	2.6857	.75815	35
I am confident I will do well on biology tests.	2.8286	.66358	35
I am confident I will do well on biology labs and projects.	2.9714	.61767	35
I believe I can master biology knowledge and skills.	2.7429	.56061	35
I believe I can earn a grade of "A" in biology.	2.8000	.63246	35
I am sure I can understand biology.	2.7714	.64561	35

Women

	Mean	Std. Deviation	N
If I study hard I can do well in biology	2.4706	.70648	34
I believe biology is too easy for me to learn	2.4412	.66017	34
The idea of taking biology makes me excited.	2.3235	.58881	34
I am confident I will do well on biology tests.	2.2941	.62906	34
I am confident I will do well on biology labs and projects.	2.4118	.65679	34
I believe I can master biology knowledge and skills.	2.3235	.58881	34

I believe I can earn a grade of “A” in biology.	2.4118	.49955	34
I am sure I can understand biology.	2.3529	.59708	34

Men

Table (3.2.6.1.3) The statistics for the mean and standard deviation for each item in the factor in the questionnaire.

Item-Total Statistics

	Scale Mean if Item Deleted	Scale Variance if Item Deleted	Corrected Item-Total Correlation	Squared Multiple Correlation	Cronbach's Alpha if Item Deleted
If I study hard I can do well in biology	19.4857	8.375	.425	.438	.767
I believe biology is too easy for me to learn	19.6000	8.482	.392	.385	.772
The idea of taking biology makes me excited.	19.6000	7.365	.537	.375	.749
I am confident I will do well on biology tests.	19.4571	7.844	.502	.447	.755
I am confident I will do well on biology labs and projects.	19.3143	7.928	.530	.540	.751
I believe I can master biology knowledge and skills.	19.5429	8.608	.373	.248	.774
I believe I can earn a grade of “A” in biology.	19.4857	8.139	.447	.594	.764
I am sure I can understand biology.	19.5143	7.375	.673	.725	.725

Women

	Scale Mean if Item Deleted	Scale Variance if Item Deleted	Corrected Item-Total Correlation	Squared Multiple Correlation	Cronbach's Alpha if Item Deleted

If I study hard I can do well in biology	16.5588	9.042	.643	.582	.836
I believe biology is too easy for me to learn	16.5882	9.765	.502	.529	.853
The idea of taking biology makes me excited.	16.7059	9.790	.580	.588	.843
I am confident I will do well on biology tests.	16.7353	9.473	.621	.627	.838
I am confident I will do well on biology labs and projects.	16.6176	8.789	.784	.780	.818
I believe I can master biology knowledge and skills.	16.7059	9.365	.710	.660	.828
I believe I can earn a grade of "A" in biology.	16.6176	10.849	.356	.452	.864
I am sure I can understand biology.	16.6765	9.619	.620	.665	.839

Men

Table (3.2.6.1.4) The total statistics for each item in the factor in the questionnaire.

Collaborative Learning

Case Processing Summary

		N	%
Cases	Valid	36	100.0
	Excluded[a]	0	.0
	Total	36	100.0

Women

		N	%
Cases	Valid	36	100.0
	Excluded[a]	0	.0
	Total	36	100.0

Men

Table (3.2.6.2.1) Listwise deletion based on all variable in the procedure

Reliability Statistics

Cronbach's Alpha	Cronbach's Alpha Based on Standardized Items	N of Items
.748	.735	8

Women

Cronbach's Alpha	Cronbach's Alpha Based on Standardized Items	N of Items
.774	.772	8

Men

Table (3.2.6.2.2) The Cronbach's alpha for the factor is calculated based on the number of items.

Item Statistics

	Mean	Std. Deviation	N
If I study hard I can do well in biology	2.5000	.65465	36
I believe biology is too easy for me to learn	2.3056	.82183	36
The idea of taking biology makes me excited.	2.4444	.69465	36
I am confident I will do well on biology tests.	2.5278	.65405	36
I am confident I will do well on biology labs and projects.	2.3056	.62425	36
I believe I can master biology knowledge and skills.	2.3611	.59295	36
I believe I can earn a grade of "A" in biology.	2.6667	.75593	36
I am sure I can understand biology.	2.4167	.76997	36

Women

	Mean	Std. Deviation	N
If I study hard I can do well in biology	3.2500	.96732	36
I believe biology is too easy for me to learn	3.5278	.73625	36
The idea of taking biology makes me excited.	3.6944	.98036	36
I am confident I will do well on biology tests.	3.3611	.83333	36
I am confident I will do well on biology labs and projects.	3.3889	.72812	36
I believe I can master biology knowledge and skills.	3.6389	.83333	36

I believe I can earn a grade of “A” in biology.	3.5000	.84515	36
I am sure I can understand biology.	3.3056	.85589	36

Men

Table (3.2.6.2.3) The statistics for the mean and standard deviation for each item in the factor in the questionnaire.

Item-Total Statistics

	Scale Mean if Item Deleted	Scale Variance if Item Deleted	Corrected Item-Total Correlation	Squared Multiple Correlation	Cronbach's Alpha if Item Deleted
If I study hard I can do well in biology	17.0278	9.171	.440	.458	.723
I believe biology is too easy for me to learn	17.2222	8.063	.558	.503	.697
The idea of taking biology makes me excited.	17.0833	8.364	.621	.473	.687
I am confident I will do well on biology tests.	17.0000	8.857	.528	.340	.707
I am confident I will do well on biology labs and projects.	17.2222	9.835	.285	.269	.748
I believe I can master biology knowledge and skills.	17.1667	10.943	.012	.239	.786
I believe I can earn a grade of “A” in biology.	16.8611	8.523	.509	.488	.708
I am sure I can understand biology.	17.1111	8.216	.574	.461	.694

Women

	Scale Mean if Item Deleted	Scale Variance if Item Deleted	Corrected Item-Total Correlation	Squared Multiple Correlation	Cronbach's Alpha if Item Deleted
If I study hard I can do well in biology	24.4167	13.221	.546	.474	.737
I believe biology is too easy for me to learn	24.1389	14.580	.512	.344	.746
The idea of taking biology makes me excited.	23.9722	12.542	.648	.573	.716
I am confident I will do well on biology tests.	24.3056	15.247	.316	.231	.776
I am confident I will do well on biology labs and projects.	24.2778	15.063	.426	.416	.758
I believe I can master biology knowledge and skills.	24.0278	14.542	.435	.459	.757
I believe I can earn a grade of "A" in biology.	24.1667	15.000	.349	.355	.771
I am sure I can understand biology.	24.3611	13.609	.579	.473	.732

Men

Table (3.2.6.2.4) The total statistics for each item in the factor in the questionnaire.

TL vs CL

Group Statistics

	Method	N	Mean	Std. Deviation	Std. Error Mean
Career Motivation	TL	35	2.7980	.38500	.06508
	CL	36	2.4306	.40405	.06734

Women

	Method	N	Mean	Std. Deviation	Std. Error Mean
Career Motivation	TL	34	2.3941	.43665	.07489
	CL	36	3.4456	.54631	.09105

Men

Table (3.2.6.3.1) The group statistics for each item in the factors questionnaire with method.

Independent Samples Test

		Levene's Test for Equality of Variances		t-test for Equality of Means						
									95% Confidence Interval of the Difference	
		F	Sig.	t	df	Sig. (2-tailed)	Mean Difference	Std. Error Difference	Lower	Upper
Self-Efficacy in biology Learning	Equal variances assumed	.337	.563	3.921	69	.000	.36744	.09371	.18049	.55439
	Equal variances not assumed			3.924	68.973	.000	.36744	.09365	.18062	.55427

Women

Independent Samples Test

		Levene's Test for Equality of Variances		t-test for Equality of Means						
									95% Confidence Interval of the Difference	
		F	Sig.	t	df	Sig. (2-tailed)	Mean Difference	Std. Error Difference	Lower	Upper
Self-Efficacy in biology Learning	Equal variances assumed	.361	.550	-8.862	68	.000	-1.05144	.11865	-1.28819	-.81468
	Equal variances not assumed			-8.919	66.226	.000	-1.05144	.11789	-1.28680	-.81607

Men

Table (3.2.6.3.2) The independent samples test for factors' questionnaire to determine the F values and significance

Factor 7 Self-Determination

Traditional Learning

Case Processing Summary

		N	%
Cases	Valid	35	100.0
	Excluded[a]	0	.0
	Total	35	100.0

Women

		N	%
Cases	Valid	34	100.0
	Excluded[a]	0	.0
	Total	34	100.0

Men

Table (3.2.7.1.1) Listwise deletion based on all variables in the procedure

Reliability Statistics

Cronbach's Alpha	Cronbach's Alpha Based on Standardized Items	N of Items
.800	.795	5

Women

Cronbach's Alpha	Cronbach's Alpha Based on Standardized Items	N of Items
.807	.803	5

Men

Table (3.2.7.1.2) The Cronbach's alpha for the factor is calculated based on the number of items.

Item Statistics

	Mean	Std. Deviation	N
I put enough effort into learning biology.	3.09	.702	35
I use strategies to learn biology well.	3.03	.618	35
I spend a lot of time learning biology.	3.20	.719	35
I prepare well for biology tests and labs.	3.29	.572	35
I study hard to learn biology.	2.97	.785	35

Women

	Mean	Std. Deviation	N
I put enough effort into learning biology.	2.59	.743	34
I use strategies to learn biology well.	2.53	.896	34
I spend a lot of time learning biology.	2.59	.783	34
I prepare well for biology tests and labs.	2.59	.701	34
I study hard to learn biology.	2.62	.853	34

Men

Table (3.2.7.1.3) The statistics for the mean and standard deviation for each item in the factor in the questionnaire.

Item-Total Statistics

	Scale Mean if Item Deleted	Scale Variance if Item Deleted	Corrected Item-Total Correlation	Squared Multiple Correlation	Cronbach's Alpha if Item Deleted
I put enough effort into learning biology.	12.49	4.434	.528	.283	.779
I use strategies to learn biology well.	12.54	4.667	.539	.396	.775
I spend a lot of time learning biology.	12.37	4.005	.682	.482	.728
I prepare well for biology tests and labs.	12.29	5.034	.438	.297	.801
I study hard to learn biology.	12.60	3.659	.736	.552	.707

Women

	Scale Mean if Item Deleted	Scale Variance if Item Deleted	Corrected Item-Total Correlation	Squared Multiple Correlation	Cronbach's Alpha if Item Deleted
I put enough effort into learning biology.	10.32	7.135	.329	.164	.841
I use strategies to learn biology well.	10.38	5.334	.690	.499	.737
I spend a lot of time learning biology.	10.32	5.983	.625	.403	.760
I prepare well for biology tests and labs.	10.32	6.347	.609	.518	.767
I study hard to learn biology.	10.29	5.365	.733	.599	.722

Men

Table (3.2.7.1.4) The total statistics for each item in the factor in the questionnaire.

Collaborative Learning

Case Processing Summary

		N	%
Cases	Valid	36	100.0
	Excluded[a]	0	.0
	Total	36	100.0

Women

Cases	Valid	36	100.0
	Excluded[a]	0	.0
	Total	36	100.0

Men

Table (3.2.7.2.1) Listwise deletion based on all variable in the procedure

Reliability Statistics

Cronbach's Alpha	Cronbach's Alpha Based on Standardized Items	N of Items
.771	.760	5

Women

Cronbach's Alpha	Cronbach's Alpha Based on Standardized Items	N of Items
.777	.777	5

Men

Table (3.2.7.2.2) The Cronbach's alpha for the factor is calculated based on the number of items.

Item Statistics

	Mean	Std. Deviation	N

I use the biology that I learn in school in my life.	2.22	.722	36
What I learn in my biology class helps me understand how things work in life.	2.08	.649	36
Learning biology makes me curious about things that I observe in my life.	2.17	.507	36
What we learn in biology class helps me to understand how biology affects my life.	2.17	.507	36
Learning biology helps me to make wiser decisions about my lifestyle and health.	2.19	.710	36

Women

	Mean	Std. Deviation	N
I use the biology that I learn in school in my life.	3.67	.535	36
What I learn in my biology class helps me understand how things work in life.	3.47	.736	36
Learning biology makes me curious about things that I observe in my life.	3.61	.688	36
What we learn in biology class helps me to understand how biology affects my life.	3.47	.774	36
Learning biology helps me to make wiser decisions about my lifestyle and health.	3.58	.604	36

Men

Table (3.2.7.2.3) The statistics for the mean and standard deviation for each item in the factor in the questionnaire.

Item-Total Statistics

	Scale Mean if Item Deleted	Scale Variance if Item Deleted	Corrected Item-Total Correlation	Squared Multiple Correlation	Cronbach's Alpha if Item Deleted
I use the biology that I learn in school in my life.	8.61	2.987	.644	.521	.691
What I learn in my biology class helps me understand how things work in life.	8.75	2.936	.790	.671	.634
Learning biology makes me curious about things that I observe in my life.	8.67	4.571	.132	.030	.837
What we learn in biology class helps me to understand how biology affects my life.	8.67	3.657	.619	.471	.712
Learning biology helps me to make wiser decisions about my lifestyle and health.	8.64	3.152	.579	.426	.717

Women

	Scale Mean if Item Deleted	Scale Variance if Item Deleted	Corrected Item-Total Correlation	Squared Multiple Correlation	Cronbach's Alpha if Item Deleted
I use the biology that I learn in school in my life.	14.14	4.466	.548	.352	.743
What I learn in my biology class helps me understand how things work in life.	14.33	3.429	.741	.569	.664

Learning biology makes me curious about things that I observe in my life.	14.19	4.161	.483	.274	.759
What we learn in biology class helps me to understand how biology affects my life.	14.33	3.600	.610	.507	.717
Learning biology helps me to make wiser decisions about my lifestyle and health.	14.22	4.578	.406	.314	.780

Men

Table (3.2.6.2.4) The total statistics for each item in the factor in the questionnaire.

TL vs CL

Group Statistics

	Method	N	Mean	Std. Deviation	Std. Error Mean
Self-Determination	TL	35	3.1291	.45383	.07671
	CL	36	2.1953	.43262	.07210

Women

	Method	N	Mean	Std. Deviation	Std. Error Mean
Self-Determination	TL	34	2.6356	.60354	.10351
	CL	36	3.5583	.45289	.07548

Men

Table (3.2.7.3.1) The group statistics for each item in the factor's questionnaire with method.

Independent Samples Test

		Levene's Test for Equality of Variances		t-test for Equality of Means						
									95% Confidence Interval of the Difference	
		F	Sig.	t	df	Sig. (2-tailed)	Mean Difference	Std. Error Difference	Lower	Upper
Self-Determination	Equal variances assumed	.070	.792	8.876	69	.000	.93387	.10521	.72398	1.14375
	Equal variances not assumed			8.870	68.600	.000	.93387	.10528	.72382	1.14391

Women

Independent Samples Test

		Levene's Test for Equality of Variances		t-test for Equality of Means						
									95% Confidence Interval of the Difference	
		F	Sig.	t	df	Sig. (2-tailed)	Mean Difference	Std. Error Difference	Lower	Upper
Self-Determination	Equal variances assumed	1.307	.257	-7.262	68	.000	-.92275	.12707	-1.17631	-.66918
	Equal variances not assumed			-7.203	61.131	.000	-.92275	.12811	-1.17890	-.66659

Men

Table (3.2.7.3.2) The independent samples test for factor questionnaire to determine the F values and significance.

Factor 8 Grade Motivation

Traditional Learning

Case Processing Summary

		N	%
Cases	Valid	35	100.0
	Excluded[a]	0	.0
	Total	35	100.0

Women

		N	%
Cases	Valid	34	100.0
	Excluded[a]	0	.0
	Total	34	100.0

Men

Table (3.2.8.1.1) Listwise deletion based on all variables in the procedure

Reliability Statistics

Cronbach's Alpha	Cronbach's Alpha Based on Standardized Items	N of Items
.776	.775	5

Women

Cronbach's Alpha	Cronbach's Alpha Based on Standardized Items	N of Items
.848	.849	5

Men

Table (3.2.8.1.2) The Cronbach’s alpha for the factor is calculated based on the number of items.

Item Statistics

	Mean	Std. Deviation	N
I like to do better than other students on biology tests.	3.20	.632	35
Getting a good biology grade is important to me.	3.17	.707	35
It is important that I get an "A" in biology.	3.03	.664	35
I think about the grade I will get in biology.	3.23	.690	35
Scoring high on biology tests and labs matters to me.	3.00	.728	35

Women

	Mean	Std. Deviation	N
I like to do better than other students on biology tests.	2.82	.673	34
Getting a good biology grade is important to me.	2.79	.641	34
It is important that I get an "A" in biology.	2.94	.600	34
I think about the grade I will get in biology.	2.79	.641	34

Scoring high on biology tests and labs matters to me.	2.94	.694	34

Men

Table (3.2.8.1.3) The statistics for the mean and standard deviation for each item in the factor in the questionnaire.

Item-Total Statistics

	Scale Mean if Item Deleted	Scale Variance if Item Deleted	Corrected Item-Total Correlation	Squared Multiple Correlation	Cronbach's Alpha if Item Deleted
I like to do better than other students on biology tests.	12.43	4.487	.483	.262	.756
Getting a good biology grade is important to me.	12.46	3.961	.612	.428	.712
It is important that I get an "A" in biology.	12.60	4.424	.472	.275	.759
I think about the grade I will get in biology.	12.40	4.071	.588	.383	.721
Scoring high on biology tests and labs matters to me.	12.63	3.946	.590	.392	.720

Women

	Scale Mean if Item Deleted	Scale Variance if Item Deleted	Corrected Item-Total Correlation	Squared Multiple Correlation	Cronbach's Alpha if Item Deleted
I like to do better than other students on biology tests.	11.47	4.196	.700	.521	.805
Getting a good biology grade is important to me.	11.50	4.561	.587	.360	.835
It is important that I get an "A" in biology.	11.35	4.357	.743	.577	.796

I think about the grade I will get in biology.	11.50	4.500	.613	.389	.828
Scoring high on biology tests and labs matters to me.	11.35	4.235	.652	.472	.819

Men

Table (3.2.8.1.4) The total statistics for each item in the factor in the questionnaire.

Collaborative Learning

Case Processing Summary

		N	%
Cases	Valid	36	100.0
	Excluded[a]	0	.0
	Total	36	100.0

Women

Cases	Valid	36	100.0
	Excluded[a]	0	.0
	Total	36	100.0

Men

Table (3.2.8.2.1) Listwise deletion based on all variable in the procedure

Reliability Statistics

Cronbach's Alpha	Cronbach's Alpha Based on Standardized Items	N of Items
.790	.786	5

Women

Cronbach's Alpha	Cronbach's Alpha Based on Standardized Items	N of Items
.866	.871	5

Men

Table (3.2.8.2.2) The Cronbach's alpha for the factor is calculated based on the number of items.

Item Statistics

	Mean	Std. Deviation	N
I use the biology that I learn in school in my life.	2.25	.770	36
What I learn in my biology class helps me understand how things work in life.	2.22	.681	36
Learning biology makes me curious about things that I observe in my life.	2.50	.737	36
What we learn in biology class helps me to understand how biology affects my life.	2.03	.736	36
Learning biology helps me to make wiser decisions about my lifestyle and health.	2.42	.732	36

Women

	Mean	Std. Deviation	N
I use the biology that I learn in school in my life.	3.89	.747	36
What I learn in my biology class helps me understand how things work in life.	4.11	.919	36
Learning biology makes me curious about things that I observe in my life.	4.14	.683	36

What we learn in biology class helps me to understand how biology affects my life.	4.06	1.040	36
Learning biology helps me to make wiser decisions about my lifestyle and health.	3.89	.667	36

Men

Table (3.2.8.2.3) The statistics for the mean and standard deviation for each item in the factor in the questionnaire.

Item-Total Statistics

	Scale Mean if Item Deleted	Scale Variance if Item Deleted	Corrected Item-Total Correlation	Squared Multiple Correlation	Cronbach's Alpha if Item Deleted
I use the biology that I learn in school in my life.	9.17	4.657	.610	.444	.737
What I learn in my biology class helps me understand how things work in life.	9.19	5.933	.266	.180	.836
Learning biology makes me curious about things that I observe in my life.	8.92	4.536	.701	.517	.706
What we learn in biology class helps me to understand how biology affects my life.	9.39	4.816	.594	.441	.742
Learning biology helps me to make wiser decisions about my lifestyle and health.	9.00	4.571	.694	.531	.709

Women

	Scale Mean if Item Deleted	Scale Variance if Item Deleted	Corrected Item-Total Correlation	Squared Multiple Correlation	Cronbach's Alpha if Item Deleted
I use the biology that I learn in school in my life.	16.19	8.161	.546	.416	.870
What I learn in my biology class helps me understand how things work in life.	15.97	6.542	.779	.731	.813
Learning biology makes me curious about things that I observe in my life.	15.94	7.711	.758	.635	.827
What we learn in biology class helps me to understand how biology affects my life.	16.03	5.971	.786	.685	.817
Learning biology helps me to make wiser decisions about my lifestyle and health.	16.19	8.161	.642	.456	.851

Men

Table (3.2.8.2.4) The total statistics for each item in the factor in the questionnaire.

TL vs CL

Group Statistics

	Method	N	Mean	Std. Deviation	Std. Error Mean
Self-Determination	TL	35	3.1194	.48481	.08195
	CL	36	2.2572	.52861	.08810

Women

	Method	N	Mean	Std. Deviation	Std. Error Mean
Self-Determination	TL	34	2.8076	.40625	.06967
	CL	36	3.9106	.61243	.10207

Men

Table (3.2.8.3.1) The group statistics for each item in the factor's questionnaire with method.

Independent Samples Test

		Levene's Test for Equality of Variances		t-test for Equality of Means						
									95% Confidence Interval of the Difference	
		F	Sig.	t	df	Sig. (2-tailed)	Mean Difference	Std. Error Difference	Lower	Upper
Grade Motivation	Equal variances assumed	.111	.741	7.157	69	.000	.86221	.12047	.62187	1.10254
	Equal variances not assumed			7.166	68.770	.000	.86221	.12032	.62216	1.10226

Women

Independent Samples Test

		Levene's Test for Equality of Variances		t-test for Equality of Means						
									95% Confidence Interval of the Difference	
		F	Sig.	t	df	Sig. (2-tailed)	Mean Difference	Std. Error Difference	Lower	Upper
Grade Motivation	Equal variances assumed	.602	.441	-8.824	68	.000	-1.10291	.12498	-1.35231	-.85351
	Equal variances not assumed			-8.924	61.136	.000	-1.10291	.12358	-1.35002	-.85580

Men

Table (3.2.8.3.2) The independent samples test for the factor to determine the F values and significance.

Factor 9 Assessment anxiety

Traditional Learning

Case Processing Summary

		N	%
Cases	Valid	35	100.0
	Excluded[a]	0	.0
	Total	35	100.0

Women

		N	%
Cases	Valid	34	100.0
	Excluded[a]	0	.0
	Total	34	100.0

Men

Table (3.2.9.1.1) Listwise deletion based on all variables in the procedure

Reliability Statistics

Cronbach's Alpha	Cronbach's Alpha Based on Standardized Items	N of Items
.772	.770	5

Women

Cronbach's Alpha	Cronbach's Alpha Based on Standardized Items	N of Items
.792	.776	5

Men

Table (3.2.9.1.2) The Cronbach's alpha for the factor is calculated based on the number of items.

Item Statistics

	Mean	Std. Deviation	N
I am nervous about how I will do on the biology tests.	2.51	.612	35
I become anxious when it is time to take a biology test.	2.57	.608	35
I worry about failing the biology tests.	2.71	.667	35
I am concerned that the other students are better in biology.	2.71	.710	35
I hate taking the biology tests.	2.49	.612	35

Women

	Mean	Std. Deviation	N
I am nervous about how I will do on the biology tests.	2.65	.691	34
I become anxious when it is time to take a biology test.	3.03	.870	34
I worry about failing the biology tests.	3.15	.989	34
I am concerned that the other students are better in biology.	3.15	1.019	34
I hate taking the biology tests.	2.65	.646	34

Men

Table (3.2.9.1.3) The statistics for the mean and standard deviation for each item in the factor in the questionnaire.

Item-Total Statistics

	Scale Mean if Item Deleted	Scale Variance if Item Deleted	Corrected Item-Total Correlation	Squared Multiple Correlation	Cronbach's Alpha if Item Deleted
I am nervous about how I will do on the biology tests.	10.49	4.081	.387	.190	.780
I become anxious when it is time to take a biology test.	10.43	3.782	.533	.287	.734
I worry about failing the biology tests.	10.29	3.563	.557	.418	.726
I am concerned that the other students are better in biology.	10.29	3.210	.667	.520	.683
I hate taking the biology tests.	10.51	3.669	.583	.380	.718

Women

	Scale Mean if Item Deleted	Scale Variance if Item Deleted	Corrected Item-Total Correlation	Squared Multiple Correlation	Cronbach's Alpha if Item Deleted
I am nervous about how I will do on the biology tests.	11.97	8.151	.348	.310	.812
I become anxious when it is time to take a biology test.	11.59	6.189	.706	.568	.706
I worry about failing the biology tests.	11.47	5.590	.734	.819	.692
I am concerned that the other students are better in biology.	11.47	5.348	.767	.789	.678
I hate taking the biology tests.	11.97	8.393	.318	.300	.817

Men

Table (3.2.9.1.4) The total statistics for each item in the factor in the questionnaire

Collaborative Learning

Case Processing Summary

		N	%
Cases	Valid	36	100.0
	Excluded[a]	0	.0
	Total	36	100.0

Women

Cases	Valid	36	100.0
	Excluded[a]	0	.0
	Total	36	100.0

Men

Table (3.2.9.2.1) Listwise deletion based on all variable in the procedure

Reliability Statistics

Cronbach's Alpha	Cronbach's Alpha Based on Standardized Items	N of Items
.787	.787	5

Women

Cronbach's Alpha	Cronbach's Alpha Based on Standardized Items	N of Items
.793	.792	5

Men

Table (3.2.9.2.2) The Cronbach's alpha for the factor is calculated based on the number of items.

Item Statistics

	Mean	Std. Deviation	N
I am nervous about how I will do on the biology tests.	2.03	.654	36
I become anxious when it is time to take a biology test.	2.22	.760	36
I worry about failing the biology tests.	2.22	.866	36
I am concerned that the other students are better in biology.	2.08	.806	36
I hate taking the biology tests.	2.08	.937	36

Women

	Mean	Std. Deviation	N
I am nervous about how I will do on the biology tests.	3.58	.770	36
I become anxious when it is time to take a biology test.	3.69	.624	36
I worry about failing the biology tests.	3.89	.854	36
I am concerned that the other students are better in biology.	3.83	.775	36
I hate taking the biology tests.	3.75	.874	36

Men

Table (3.2.9.2.3) The statistics for the mean and standard deviation for each item in the factor in the questionnaire.

Item-Total Statistics

	Scale Mean if Item Deleted	Scale Variance if Item Deleted	Corrected Item-Total Correlation	Squared Multiple Correlation	Cronbach's Alpha if Item Deleted
I am nervous about how I will do on the biology tests.	8.61	6.816	.475	.338	.775
I become anxious when it is time to take a biology test.	8.42	5.964	.626	.434	.728
I worry about failing the biology tests.	8.42	5.450	.660	.464	.713
I am concerned that the other students are better in biology.	8.56	6.311	.470	.315	.777
I hate taking the biology tests.	8.56	5.340	.611	.403	.733

Women

	Scale Mean if Item Deleted	Scale Variance if Item Deleted	Corrected Item-Total Correlation	Squared Multiple Correlation	Cronbach's Alpha if Item Deleted
I am nervous about how I will do on the biology tests.	15.17	5.971	.494	.449	.779
I become anxious when it is time to take a biology test.	15.06	6.283	.559	.481	.763
I worry about failing the biology tests.	14.86	5.209	.636	.445	.733
I am concerned that the other students are better in biology.	14.92	6.136	.439	.473	.795
I hate taking the biology tests.	15.00	4.743	.765	.650	.683

Men

Table (3.2.9.2.4) The total statistics for each item in the factor in the questionnaire.

TL vs CL

Group Statistics

	Method	N	Mean	Std. Deviation	Std. Error Mean
Self-Determination	TL	35	2.6117	.41953	.07091
	CL	36	2.1003	.58776	.09796

Women

	Method	N	Mean	Std. Deviation	Std. Error Mean
Self-Determination	TL	34	2.9238	.60845	.10435
	CL	36	3.6597	.51958	.08660

Men

Table (3.2.9.3.1) The group statistics for each item in the factor's questionnaire with method.

Independent Samples Test

		Levene's Test for Equality of Variances		t-test for Equality of Means						
									95% Confidence Interval of the Difference	
		F	Sig.	t	df	Sig. (2-tailed)	Mean Difference	Std. Error Difference	Lower	Upper
Aassessment anxiety	Equal variances assumed	4.815	.032	4.210	69	.000	.51144	.12150	.26906	.75381
	Equal variances not assumed			4.229	63.378	.000	.51144	.12093	.26980	.75307

Women

Independent Samples Test

		Levene's Test for Equality of Variances		t-test for Equality of Means						
									95% Confidence Interval of the Difference	
		F	Sig.	t	df	Sig. (2-tailed)	Mean Difference	Std. Error Difference	Lower	Upper
Aassessment anxiety	Equal variances assumed	1.750	.190	-5.452	68	.000	-.73590	.13499	-1.00526	-.46654
	Equal variances not assumed			-5.427	65.027	.000	-.73590	.13560	-1.00671	-.46509

Men

Table (3.2.9.3.2) The independent samples test for factor in questionnaire to determine the F values and significance.

All factors comparison

Group Statistics

	Method	N	Mean	Std. Deviation	Std. Error Mean
Attitude_9 Factors	TL	315	3.1807	.62924	.03545
	CL	324	2.7035	.71397	.03967

Women

	Method	N	Mean	Std. Deviation	Std. Error Mean
Attitude_9 Factors	TL	306	2.6899	.60389	.03452
	CL	324	3.7489	.55266	.03070

Men

Table (3.2.10.3.1) The group statistics for each item in the factor's questionnaire with method

Independent Samples Test

		Levene's Test for Equality of Variances		t-test for Equality of Means						
									95% Confidence Interval of the Difference	
		F	Sig.	t	df	Sig. (2-tailed)	Mean Difference	Std. Error Difference	Lower	Upper
Attitude_9 Factors	Equal variances assumed	15.311	.000	8.955	637	.000	.47727	.05329	.37262	.58193
	Equal variances not assumed			8.971	630.982	.000	.47727	.05320	.37280	.58174

Women

Independent Samples Test

		Levene's Test for Equality of Variances		t-test for Equality of Means						
									95% Confidence Interval of the Difference	
		F	Sig.	t	df	Sig. (2-tailed)	Mean Difference	Std. Error Difference	Lower	Upper
Attitude_9 Factors	Equal variances assumed	6.865	.009	-22.980	628	.000	-1.05899	.04608	-1.14948	-.96849
	Equal variances not assumed			-22.922	614.994	.000	-1.05899	.04620	-1.14971	-.96826

Men

Table (3.2.10.3.2) The independent samples test for all factor in questionnaire to determine the F values and significance.

Attitude Women TL (All factors) VS Men TL (All factors)

Group Statistics

	Gender	N	Mean	Std. Deviation	Std. Error Mean
Attitude_9 Factors	Women	315	3.0594	.55055	.03102
	Men	306	2.6899	.60389	.03452

Table (3.2.10.3.3) The group statistics to compare women and men in all factors in TL

Independent Samples Test

		Levene's Test for Equality of Variances		t-test for Equality of Means						
									95% Confidence Interval of the Difference	
		F	Sig.	t	df	Sig. (2-tailed)	Mean Difference	Std. Error Difference	Lower	Upper
Attitude_9 Factors	Equal variances assumed	4.310	.038	7.972	619	.000	.36949	.04635	.27847	.46052
	Equal variances not assumed			7.961	610.050	.000	.36949	.04641	.27835	.46064

Table (3.2.10.3.4) The independent samples test for all factor in questionnaire to determine the F values and significance.

Attitude Women CL (All factors) VS Men CL (All factors)

Group Statistics

	Gender	N	Mean	Std. Deviation	Std. Error Mean
Attitude_9 Factors	Women	324	2.5969	.65646	.03647
	Men	324	3.7489	.55266	.03070

Table (3.2.10.3.5) The group statistics to compare women and men in all factors in CL

Independent Samples Test

		Levene's Test for Equality of Variances		t-test for Equality of Means						
									95% Confidence Interval of the Difference	
		F	Sig.	t	df	Sig. (2-tailed)	Mean Difference	Std. Error Difference	Lower	Upper
Attitude_9 Factors	Equal variances assumed	19.406	.000	-24.164	646	.000	-1.15198	.04767	-1.24559	-1.05836
	Equal variances not assumed			-24.164	627.763	.000	-1.15198	.04767	-1.24559	-1.05836

Table (3.2.10.3.6) The independent samples test for all factor in questionnaire to determine the F values and significance.

Major Biology

Single Gender Students

Factor 1 Feelings towards Biology

Traditional Learning

		N	%
Cases	Valid	30	100.0
	Excluded[a]	0	.0
	Total	30	100.0

Women

		N	%
Cases	Valid	30	100.0
	Excluded[a]	0	.0
	Total	30	100.0

Men

Table (3.3.1.1.1) Listwise deletion based on all variables in the procedure.

Reliability Statistics

Cronbach's Alpha	Cronbach's Alpha Based on Standardized Items	N of Items
.883	.876	14

Women

Cronbach's Alpha	Cronbach's Alpha Based on Standardized Items	N of Items
.898	.890	14

Men

Table (3.3.1.1.2) The Cronbach's alpha for the factor is calculated based on the number of items.

Item Statistics

	Mean	Std. Deviation	N
Biology is very interesting to me.	4.40	.814	30
I have always enjoyed studying biology in school.	4.37	.850	30
I am always under a terrible strain in a biology class.	4.33	.661	30
I feel a definite positive reaction to biology; it's enjoyable.	3.93	1.143	30
Biology makes me feel secure, and at the same time it is stimulating.	3.93	.868	30
I feel at ease in biology and like it very much.	3.77	.935	30
In general, I have a good feeling toward biology.	3.90	.803	30
I really like biology.	3.83	.986	30
Biology is fascinating and fun.	3.87	.860	30
When I hear the word biology, I have a feeling of dislike.	3.67	.994	30
I approach biology with a feeling of hesitation.	4.00	.983	30
It makes me nervous to even think about doing a biology experiment.	3.83	1.117	30
Biology makes me feel uncomfortable, restless, irritable, and impatient.	4.30	.837	30

I don't like biology, and it scares me to have to take it.	3.83	1.020	30

	Women		
	Mean	Std. Deviation	N
Biology is very interesting to me.	4.30	.877	30
I have always enjoyed studying biology in school.	3.17	1.315	30
I am always under a terrible strain in a biology class.	3.33	1.348	30
I feel a definite positive reaction to biology; it's enjoyable.	3.17	1.315	30
Biology makes me feel secure, and at the same time it is stimulating.	3.20	1.157	30
I feel at ease in biology and like it very much.	2.93	1.172	30
In general, I have a good feeling toward biology.	3.20	1.126	30
I really like biology.	2.93	1.230	30
Biology is fascinating and fun.	3.13	1.167	30
When I hear the word biology, I have a feeling of dislike.	2.87	1.167	30
I approach biology with a feeling of hesitation.	3.33	1.213	30
It makes me nervous to even think about doing a biology experiment.	3.67	1.124	30
Biology makes me feel uncomfortable, restless, irritable, and impatient.	4.13	.900	30
I don't like biology, and it scares me to have to take it.	3.83	1.020	30

Men

Table (3.3.1.1.3) The statistics for the mean and standard deviation for each item in the factor in the questionnaire.

Item-Total Statistics

	Scale Mean if Item Deleted	Scale Variance if Item Deleted	Corrected Item-Total Correlation	Squared Multiple Correlation	Cronbach's Alpha if Item Deleted
Biology is very interesting to me.	51.57	63.082	.236	.828	.888
I have always enjoyed studying biology in school.	51.60	65.490	.042	.554	.896
I am always under a terrible strain in a biology class.	51.63	61.620	.456	.541	.879
I feel a definite positive reaction to biology; it's enjoyable.	52.03	55.206	.605	.771	.872
Biology makes me feel secure, and at the same time it is stimulating.	52.03	57.137	.678	.728	.869
I feel at ease in biology and like it very much.	52.20	54.648	.815	.833	.862
In general, I have a good feeling toward biology.	52.07	60.340	.465	.669	.878
I really like biology.	52.13	55.223	.724	.662	.866
Biology is fascinating and fun.	52.10	57.403	.663	.849	.870
When I hear the word biology, I have a feeling of dislike.	52.30	53.459	.849	.847	.859
I approach biology with a feeling of hesitation.	51.97	56.378	.640	.812	.870
It makes me nervous to even think about doing a biology experiment.	52.13	51.430	.881	.914	.856
Biology makes me feel uncomfortable, restless, irritable, and impatient.	51.67	63.540	.191	.734	.890

I don't like biology, and it scares me to have to take it.	52.13	58.602	.458	.675	.880

Women

	Scale Mean if Item Deleted	Scale Variance if Item Deleted	Corrected Item-Total Correlation	Squared Multiple Correlation	Cronbach's Alpha if Item Deleted
Biology is very interesting to me.	42.90	112.093	.100	.473	.909
I have always enjoyed studying biology in school.	44.03	94.999	.702	.801	.888
I am always under a terrible strain in a biology class.	43.87	92.257	.797	.880	.883
I feel a definite positive reaction to biology; it's enjoyable.	44.03	92.102	.827	.832	.882
Biology makes me feel secure, and at the same time it is stimulating.	44.00	94.621	.834	.934	.883
I feel at ease in biology and like it very much.	44.27	93.030	.898	.927	.880
In general, I have a good feeling toward biology.	44.00	98.345	.676	.818	.890
I really like biology.	44.27	92.754	.863	.913	.881
Biology is fascinating and fun.	44.07	95.720	.773	.903	.885
When I hear the word biology, I have a feeling of dislike.	44.33	93.540	.878	.881	.881
I approach biology with a feeling of hesitation.	43.87	113.085	.006	.360	.917
It makes me nervous to even think about doing a biology experiment.	43.53	105.913	.326	.631	.903

Biology makes me feel uncomfortable, restless, irritable, and impatient.	43.07	112.685	.064	.476	.910
I don't like biology, and it scares me to have to take it.	43.37	104.309	.450	.568	.898

Men

Table (3.3.1.1.4) The total statistics for each item in the factor in the questionnaire.

Collaborative Learning

Case Processing Summary

		N	%
Cases	Valid	31	100.0
	Excluded[a]	0	.0
	Total	31	100.0

Women

		N	%
Cases	Valid	32	100.0
	Excluded[a]	0	.0
	Total	32	100.0

Men

Table (3.3.1.2.1) Listwise deletion based on all variables in the procedure.

Reliability Statistics

Cronbach's Alpha	Cronbach's Alpha Based on Standardized Items	N of Items
.896	.854	13

Women

Cronbach's Alpha	Cronbach's Alpha Based on Standardized Items	N of Items

.797	.792	14

Men

Table (3.3.1.2.2) The Cronbach's alpha for the feelings towards biology factor is calculated based on the number of items

Item Statistics

	Mean	Std. Deviation	N
I have always enjoyed studying biology in school.	4.97	.180	31
I am always under a terrible strain in a biology class.	4.94	.250	31
I feel a definite positive reaction to biology; it's enjoyable.	4.81	.402	31
Biology makes me feel secure, and at the same time it is stimulating.	4.77	.762	31
I feel at ease in biology and like it very much.	4.84	.374	31
In general, I have a good feeling toward biology.	4.61	1.022	31
I really like biology.	4.68	.541	31
Biology is fascinating and fun.	4.65	1.018	31

Women

	Mean	Std. Deviation	N
Biology is very interesting to me.	4.69	.693	32
I have always enjoyed studying biology in school.	4.41	1.103	32
I am always under a terrible strain in a biology class.	3.91	.818	32

I feel a definite positive reaction to biology; it's enjoyable.	3.78	.832	32
Biology makes me feel secure, and at the same time it is stimulating.	3.94	.716	32
I feel at ease in biology and like it very much.	3.81	.965	32
In general, I have a good feeling toward biology.	3.59	1.073	32
I really like biology.	4.13	.751	32
Biology is fascinating and fun.	4.19	1.306	32
When I hear the word biology, I have a feeling of dislike.	4.41	1.132	32
I approach biology with a feeling of hesitation.	4.34	1.285	32
It makes me nervous to even think about doing a biology experiment.	4.56	1.076	32
Biology makes me feel uncomfortable, restless, irritable, and impatient.	4.56	1.014	32
I don't like biology, and it scares me to have to take it.	4.66	.787	32

Men

Table (3.3.1.2.3) The statistics for the mean and standard deviation for each item in the factor in the questionnaire.

Item-Total Statistics

	Scale Mean if Item Deleted	Scale Variance if Item Deleted	Corrected Item-Total Correlation	Squared Multiple Correlation	Cronbach's Alpha if Item Deleted

I have always enjoyed studying biology in school.	56.71	40.480	-.067	.180	.903
I am always under a terrible strain in a biology class.	56.74	40.398	-.032	.311	.904
I feel a definite positive reaction to biology; it's enjoyable.	56.87	38.849	.269	.813	.899
Biology makes me feel secure, and at the same time it is stimulating.	56.90	33.357	.730	.918	.881
I feel at ease in biology and like it very much.	56.84	38.873	.289	.730	.899
In general, I have a good feeling toward biology.	57.06	28.996	.937	.937	.867
I really like biology.	57.00	36.200	.594	.663	.889
Biology is fascinating and fun.	57.03	29.099	.931	.926	.868
When I hear the word biology, I have a feeling of dislike.	56.94	31.729	.867	.912	.873
I approach biology with a feeling of hesitation.	57.06	29.062	.930	.930	.868
It makes me nervous to even think about doing a biology experiment.	56.97	34.966	.484	.700	.894
Biology makes me feel uncomfortable, restless, irritable, and impatient.	56.97	34.766	.539	.687	.891
I don't like biology, and it scares me to have to take it.	57.03	34.166	.596	.864	.888

Women

	Scale Mean if Item Deleted	Scale Variance if Item Deleted	Corrected Item-Total Correlation	Squared Multiple Correlation	Cronbach's Alpha if Item Deleted

Biology is very interesting to me.	54.28	46.725	.564	.872	.778
I have always enjoyed studying biology in school.	54.56	44.835	.440	.789	.783
I am always under a terrible strain in a biology class.	55.06	47.673	.373	.694	.788
I feel a definite positive reaction to biology; it's enjoyable.	55.19	47.964	.338	.601	.790
Biology makes me feel secure, and at the same time it is stimulating.	55.03	48.805	.323	.806	.791
I feel at ease in biology and like it very much.	55.16	47.297	.325	.543	.792
In general, I have a good feeling toward biology.	55.38	45.726	.391	.464	.787
I really like biology.	54.84	49.943	.192	.605	.799
Biology is fascinating and fun.	54.78	37.983	.799	.850	.742
When I hear the word biology, I have a feeling of dislike.	54.56	46.512	.308	.711	.795
I approach biology with a feeling of hesitation.	54.63	41.210	.587	.718	.768
It makes me nervous to even think about doing a biology experiment.	54.41	47.604	.255	.704	.799
Biology makes me feel uncomfortable, restless, irritable, and impatient.	54.41	43.604	.591	.649	.769
I don't like biology, and it scares me to have to take it.	54.31	48.351	.327	.867	.791

Men

Table (3.3.1.2.4) The total statistics for each item in the factor in the questionnaire.

TL vs CL

Group Statistics

	Method	N	Mean	Std. Deviation	Std. Error Mean
Feeling toward biology	TL	30	4.0183	.56912	.10391
	CL	31	4.8287	.45678	.08204

Women

	Method	N	Mean	Std. Deviation	Std. Error Mean
Feeling toward biology	TL	30	3.3830	.74417	.13587
	CL	32	4.1331	.53831	.09516

Men

Table (3.3.1.3.1) The group statistics for each item in the factors questionnaire with method.

Independent Samples Test

		Levene's Test for Equality of Variances		t-test for Equality of Means						
									95% Confidence Interval of the Difference	
		F	Sig.	t	df	Sig. (2-tailed)	Mean Difference	Std. Error Difference	Lower	Upper
Feeling toward biology	Equal variances assumed	7.225	.009	-6.143	59	.000	-.81038	.13191	-1.07433	-.54642
	Equal variances not assumed			-6.121	55.556	.000	-.81038	.13239	-1.07563	-.54512

Women

Independent Samples Test

		Levene's Test for Equality of Variances		t-test for Equality of Means						
									95% Confidence Interval of the Difference	
		F	Sig.	t	df	Sig. (2-tailed)	Mean Difference	Std. Error Difference	Lower	Upper
Feeling toward biology	Equal variances assumed	10.043	.002	-4.569	60	.000	-.75013	.16418	-1.07854	-.42171
	Equal variances not assumed			-4.522	52.592	.000	-.75013	.16588	-1.08289	-.41736

Men

Table (3.3.1.3.2) The independent samples test for feelings towards biology questionnaire to determine the F values and significance.

Factor 2 General Interest

Traditional Learning

Case Processing Summary

		N	%
Cases	Valid	30	100.0
	Excluded[a]	0	.0
	Total	30	100.0

Women

		N	%
Cases	Valid	30	100.0
	Excluded[a]	0	.0
	Total	30	100.0

Men

Table (3.3.2.1.1) Listwise deletion based on all variables in the procedure.

Reliability Statistics

Cronbach's Alpha	Cronbach's Alpha Based on Standardized Items	N of Items
.840	.845	5

Women

Cronbach's Alpha	Cronbach's Alpha Based on Standardized Items	N of Items
.830	.832	5

Men

Table (3.3.2.1.2) The Cronbach's alpha for the factor is calculated based on the number of items.

Item Statistics

	Mean	Std. Deviation	N

I like watching biology related TV.	4.10	.712	30
biology is my favorite subject in school.	4.17	.699	30
I like reading about famous biologiest	4.03	1.066	30
I find what we learn in my biology class interesting.	4.10	.923	30
I would enjoy working in a biology lab.	4.17	.874	30

Women

	Mean	Std. Deviation	N
I like watching biology related TV.	3.97	.765	30
biology is my favorite subject in school.	3.87	.860	30
I like reading about famous biologiest	3.83	.950	30
I find what we learn in my biology class interesting.	3.97	.928	30
I would enjoy working in a biology lab.	3.93	.980	30

Men

Table (3.3.2.1.3) The statistics for the mean and standard deviation for each item in the factor in the questionnaire.

Item-Total Statistics

	Scale Mean if Item Deleted	Scale Variance if Item Deleted	Corrected Item-Total Correlation	Squared Multiple Correlation	Cronbach's Alpha if Item Deleted
I like watching biology related TV.	16.47	8.189	.670	.496	.806
biology is my favorite subject in school.	16.40	8.524	.591	.504	.823

I like reading about famous biologiest	16.53	6.395	.722	.575	.789
I find what we learn in my biology class interesting.	16.47	7.499	.609	.474	.818
I would enjoy working in a biology lab.	16.40	7.421	.680	.504	.797

Women

	Scale Mean if Item Deleted	Scale Variance if Item Deleted	Corrected Item-Total Correlation	Squared Multiple Correlation	Cronbach's Alpha if Item Deleted
I like watching biology related TV.	15.60	8.593	.640	.596	.796
biology is my favorite subject in school.	15.70	8.286	.610	.475	.801
I like reading about famous biologiest	15.73	7.513	.698	.583	.775
I find what we learn in my biology class interesting.	15.60	8.455	.506	.550	.831
I would enjoy working in a biology lab.	15.63	7.344	.704	.609	.773

Men

Table (3.3.2.1.4) The total statistics for each item in the factor in the questionnaire.

Collaborative Learning

Case Processing Summary

		N	%
Cases	Valid	31	100.0
	Excluded[a]	0	.0
	Total	31	100.0

Women

		N	%
Cases	Valid	32	100.0
	Excluded[a]	0	.0
	Total	32	100.0

Men

Table (3.3.2.2.1) Listwise deletion based on all variables in the procedure.

Reliability Statistics

Cronbach's Alpha	Cronbach's Alpha Based on Standardized Items	N of Items
.902	.899	5

Women

Cronbach's Alpha	Cronbach's Alpha Based on Standardized Items	N of Items
.863	.859	5

Men

Table (3.3.2.2.2) The Cronbach’s alpha for the factor is calculated based on the number of items.

Item Statistics

	Mean	Std. Deviation	N
I like watching biology related TV.	4.55	.723	31
biology is my favorite subject in school.	4.48	.811	31
I like reading about famous biologiest	4.61	.667	31
I find what we learn in my biology class interesting.	4.61	.803	31
I would enjoy working in a biology lab.	4.48	.811	31

Women

	Mean	Std. Deviation	N
I like watching biology related TV.	4.31	.693	32
biology is my favorite subject in school.	4.31	.780	32
I like reading about famous biologiest	4.41	.712	32
I find what we learn in my biology class interesting.	4.44	.801	32
I would enjoy working in a biology lab.	4.25	.762	32

Men

Table (3.3.2.1.3) The statistics for the mean and standard deviation for each item in the factor in the questionnaire.

Item-Total Statistics

	Scale Mean if Item Deleted	Scale Variance if Item Deleted	Corrected Item-Total Correlation	Squared Multiple Correlation	Cronbach's Alpha if Item Deleted
I like watching biology related TV.	18.19	7.361	.675	.505	.897
biology is my favorite subject in school.	18.26	6.465	.826	.781	.865
I like reading about famous biologiest	18.13	8.049	.538	.381	.922
I find what we learn in my biology class interesting.	18.13	6.383	.863	.826	.856
I would enjoy working in a biology lab.	18.26	6.265	.889	.806	.850

Women

	Scale Mean if Item Deleted	Scale Variance if Item Deleted	Corrected Item-Total Correlation	Squared Multiple Correlation	Cronbach's Alpha if Item Deleted

I like watching biology related TV.	17.41	6.636	.559	.445	.864
biology is my favorite subject in school.	17.41	5.604	.785	.753	.808
I like reading about famous biologiest	17.31	6.931	.446	.292	.889
I find what we learn in my biology class interesting.	17.28	5.305	.858	.825	.786
I would enjoy working in a biology lab.	17.47	5.676	.786	.677	.808

Men

Table (3.3.2.2.4) The total statistics for each item in the factor in the questionnaire.

TL vs CL

Group Statistics

	Method	N	Mean	Std. Deviation	Std. Error Mean
General interest	TL	30	4.0990	.62379	.11389
	CL	31	4.5461	.60988	.10954

Women

	Method	N	Mean	Std. Deviation	Std. Error Mean
General interest	TL	30	3.9207	.67931	.12403
	CL	33	4.3082	.61703	.10741

Men

Table (3.3.2.3.1) The group statistics for each item in the factors questionnaire with method.

Independent Samples Test

		Levene's Test for Equality of Variances		t-test for Equality of Means						
									95% Confidence Interval of the Difference	
		F	Sig.	t	df	Sig. (2-tailed)	Mean Difference	Std. Error Difference	Lower	Upper
General interest	Equal variances assumed	.105	.747	-2.831	59	.006	-.44713	.15796	-.76320	-.13106
	Equal variances not assumed			-2.830	58.816	.006	-.44713	.15802	-.76334	-.13092

Women

Independent Samples Test

		Levene's Test for Equality of Variances		t-test for Equality of Means						
									95% Confidence Interval of the Difference	
		F	Sig.	t	df	Sig. (2-tailed)	Mean Difference	Std. Error Difference	Lower	Upper
General interest	Equal variances assumed	.354	.554	-2.373	61	.021	-.38752	.16331	-.71408	-.06095
	Equal variances not assumed			-2.362	58.826	.022	-.38752	.16407	-.71584	-.05919

Men

Table (3.3.2.3.2) The independent samples test for feelings towards biology questionnaire to determine the F values and significance.

Factor 3 Motivation Towards Learning

Traditional Learning

Case Processing Summary

		N	%
Cases	Valid	30	100.0
	Excluded[a]	0	.0
	Total	30	100.0

Women

		N	%
Cases	Valid	30	100.0
	Excluded[a]	0	.0
	Total	30	100.0

Men

Table (3.3.3.1.1) Listwise deletion based on all variables in the procedure

Reliability Statistics

Cronbach's Alpha	Cronbach's Alpha Based on Standardized Items	N of Items
.881	.827	10

Women

Cronbach's Alpha	Cronbach's Alpha Based on Standardized Items	N of Items
.772	.765	10

Men

Table (3.3.3.1.2) The Cronbach's alpha for the factor is calculated based on the number of items.

Item Statistics

	Mean	Std. Deviation	N
I will ask my teacher for an explanation if I do not understand the science topic.	4.7000	.46609	30

I will look for an explanation in the textbook if I do not understand the science topic.	4.7000	.53498	30
I care about completing assignments in this class.	4.9667	.18257	30
Getting a good grade in biology is important to me.	4.6667	.47946	30
I am interested in understanding the teacher in this class.	4.6333	1.03335	30
The biology I learn is relevant to my life.	4.7000	1.05536	30
Learning biology is interesting.	4.4333	1.07265	30
Learning biology makes my life more meaningful.	4.6000	.85501	30
I am curious about discoveries in biology.	4.6333	.80872	30
I enjoy learning biology	4.3000	.79438	30

Women

	Mean	Std. Deviation	N
I will ask my teacher for an explanation if I do not understand the science topic.	4.3333	.84418	30
I will look for an explanation in the textbook if I do not understand the science topic.	4.0000	.83045	30
I care about completing assignments in this class.	4.2000	.99655	30

Getting a good grade in biology is important to me.	4.0333	.92786	30
I am interested in understanding the teacher in this class.	3.8667	1.13664	30
The biology I learn is relevant to my life.	3.9333	1.14269	30
Learning biology is interesting.	3.7667	1.00630	30
Learning biology makes my life more meaningful.	3.9667	.92786	30
I am curious about discoveries in biology.	4.2000	.99655	30
I enjoy learning biology	4.0667	.69149	30

Men

Table (3.3.3.1.3) The statistics for the mean and standard deviation for each item in the factor in the questionnaire.

Item-Total Statistics

	Scale Mean if Item Deleted	Scale Variance if Item Deleted	Corrected Item-Total Correlation	Squared Multiple Correlation	Cronbach's Alpha if Item Deleted
I will ask my teacher for an explanation if I do not understand the science topic.	41.6333	28.585	.148	.451	.893
I will look for an explanation in the textbook if I do not understand the science topic.	41.6333	26.861	.432	.576	.881
I care about completing assignments in this class.	41.3667	29.689	-.091	.230	.895
Getting a good grade in biology is important to me.	41.6667	28.851	.089	.346	.896

I am interested in understanding the teacher in this class.	41.7000	20.148	.897	.925	.843
The biology I learn is relevant to my life.	41.6333	20.309	.853	.909	.848
Learning biology is interesting.	41.9000	20.093	.863	.859	.847
Learning biology makes my life more meaningful.	41.7333	22.064	.840	.767	.850
I am curious about discoveries in biology.	41.7000	22.424	.844	.804	.851
I enjoy learning biology	42.0333	23.413	.715	.723	.861

Women

	Scale Mean if Item Deleted	Scale Variance if Item Deleted	Corrected Item-Total Correlation	Squared Multiple Correlation	Cronbach's Alpha if Item Deleted
I will ask my teacher for an explanation if I do not understand the science topic.	36.0333	39.068	.521	.775	.881
I will look for an explanation in the textbook if I do not understand the science topic.	36.3667	37.551	.691	.871	.870
I care about completing assignments in this class.	36.1667	37.247	.578	.797	.877
Getting a good grade in biology is important to me.	36.3333	40.092	.368	.809	.891
I am interested in understanding the teacher in this class.	36.5000	32.741	.864	.940	.853
The biology I learn is relevant to my life.	36.4333	35.013	.662	.757	.871
Learning biology is interesting.	36.6000	36.593	.630	.879	.873

Learning biology makes my life more meaningful.	36.4000	36.317	.724	.912	.867
I am curious about discoveries in biology.	36.1667	36.144	.679	.782	.870
I enjoy learning biology	36.3000	40.631	.473	.790	.884

Men

Table (3.3.3.1.4) The total statistics for each item in the factor in the questionnaire.

Collaborative Learning

Case Processing Summary

		N	%
Cases	Valid	31	100.0
	Excluded[a]	0	.0
	Total	31	100.0

Women

Cases	Valid	32	100.0
	Excluded[a]	0	.0
	Total	32	100.0

Men

Table (3.3.3.2.1) Listwise deletion based on all variables in the procedure

Reliability Statistics

Cronbach's Alpha	Cronbach's Alpha Based on Standardized Items	N of Items
.867	.814	10

Women

Cronbach's Alpha	Cronbach's Alpha Based on Standardized Items	N of Items
.848	.813	10

Men

Table (3.3.3.2.2) The Cronbach's alpha for the factor is calculated based on the number of items.

Item Statistics

	Mean	Std. Deviation	N
I will ask my teacher for an explanation if I do not understand the science topic.	4.7742	.56034	31
I will look for an explanation in the textbook if I do not understand the science topic.	4.8710	.49946	31
I care about completing assignments in this class.	4.9355	.35921	31
Getting a good grade in biology is important to me.	4.8065	.40161	31
I am interested in understanding the teacher in this class.	4.6452	1.01812	31
The biology I learn is relevant to my life.	4.6774	1.01282	31
Learning biology is interesting.	4.4516	1.05952	31
Learning biology makes my life more meaningful.	4.6452	.83859	31
I am curious about discoveries in biology.	4.6774	.79108	31
I enjoy learning biology	4.4516	.80989	31

Women

	Mean	Std. Deviation	N
I will ask my teacher for an explanation if I do not understand the science topic.	4.6563	.86544	32

I will look for an explanation in the textbook if I do not understand the science topic.	4.8125	.53506		32
I care about completing assignments in this class.	4.6250	1.03954		32
Getting a good grade in biology is important to me.	4.7188	.45680		32
I am interested in understanding the teacher in this class.	4.5000	1.19137		32
The biology I learn is relevant to my life.	4.5313	1.10671		32
Learning biology is interesting.	4.4375	1.04534		32
Learning biology makes my life more meaningful.	4.5313	1.04679		32
I am curious about discoveries in biology.	4.4375	1.18967		32
I enjoy learning biology	4.3750	1.00803		32

Men

Table (3.3.3.2.3) The statistics for the mean and standard deviation for each item in the factor in the questionnaire.

Item-Total Statistics

	Scale Mean if Item Deleted	Scale Variance if Item Deleted	Corrected Item-Total Correlation	Squared Multiple Correlation	Cronbach's Alpha if Item Deleted
I will ask my teacher for an explanation if I do not understand the science topic.	42.1613	27.606	-.089	.273	.892
I will look for an explanation in the textbook if I do not understand the science topic.	42.0645	26.862	.055	.265	.883

I care about completing assignments in this class.	42.0000	27.267	.000	.596	.882
Getting a good grade in biology is important to me.	42.1290	25.716	.373	.668	.869
I am interested in understanding the teacher in this class.	42.2903	18.480	.900	.947	.821
The biology I learn is relevant to my life.	42.2581	18.665	.880	.931	.824
Learning biology is interesting.	42.4839	18.191	.894	.881	.822
Learning biology makes my life more meaningful.	42.2903	20.213	.859	.888	.829
I am curious about discoveries in biology.	42.2581	20.865	.817	.874	.834
I enjoy learning biology	42.4839	21.325	.724	.814	.842

Women

	Scale Mean if Item Deleted	Scale Variance if Item Deleted	Corrected Item-Total Correlation	Squared Multiple Correlation	Cronbach's Alpha if Item Deleted
I will ask my teacher for an explanation if I do not understand the science topic.	40.9688	38.805	.094	.551	.869
I will look for an explanation in the textbook if I do not understand the science topic.	40.8125	39.706	.085	.287	.861
I care about completing assignments in this class.	41.0000	32.516	.588	.653	.831
Getting a good grade in biology is important to me.	40.9063	39.765	.103	.434	.860

I am interested in understanding the teacher in this class.	41.1250	29.468	.748	.885	.813
The biology I learn is relevant to my life.	41.0938	30.023	.768	.870	.812
Learning biology is interesting.	41.1875	32.286	.605	.844	.829
Learning biology makes my life more meaningful.	41.0938	30.797	.746	.831	.815
I am curious about discoveries in biology.	41.1875	28.673	.822	.902	.804
I enjoy learning biology	41.2500	32.065	.656	.890	.824

Men

Table (3.3.3.2.4) The total statistics for each item in the factor in the questionnaire.

TL vs CL

Group Statistics

	Method	N	Mean	Std. Deviation	Std. Error Mean
Motivation Towards Learning Biology	TL	30	4.4210	.49438	.09026
	CL	31	4.7639	.52704	.09466

Women

	Method	N	Mean	Std. Deviation	Std. Error Mean
Motivation Towards Learning Biology	TL	30	4.1077	.65004	.11868
	CL	32	4.6250	.65653	.11606

Men

Table (3.3.3.3.1) The group statistics for each item in the factors Questionnaire with method.

Independent Samples Test

		Levene's Test for Equality of Variances		t-test for Equality of Means						
									95% Confidence Interval of the Difference	
		F	Sig.	t	df	Sig. (2-tailed)	Mean Difference	Std. Error Difference	Lower	Upper
Motivation Towards Learning Biology	Equal variances assumed	.709	.403	-2.619	59	.011	-.34287	.13093	-.60487	-.08087
	Equal variances not assumed			-2.621	58.945	.011	-.34287	.13080	-.60460	-.08115

Women

Independent Samples Test

		Levene's Test for Equality of Variances		t-test for Equality of Means						
									95% Confidence Interval of the Difference	
		F	Sig.	t	df	Sig. (2-tailed)	Mean Difference	Std. Error Difference	Lower	Upper
Motivation Towards Learning Biology	Equal variances assumed	.002	.967	-3.116	60	.003	-.51733	.16605	-.84948	-.18518
	Equal variances not assumed			-3.117	59.814	.003	-.51733	.16600	-.84940	-.18527

Men

Table (3.3.3.3.2) The independent samples test for factor to determine the F values and significance.

Factor 4 Benefit and Utility of biology

Traditional Learning

Case Processing Summary

		N	%
Cases	Valid	30	100.0
	Excluded[a]	0	.0
	Total	30	100.0

Women

		N	%
Cases	Valid	30	100.0
	Excluded[a]	0	.0
	Total	30	100.0

Men

Table (3.3.4.1.1) Listwise deletion based on all variables in the procedure

Reliability Statistics

Cronbach's Alpha	Cronbach's Alpha Based on Standardized Items	N of Items
.824	.824	5

Women

Cronbach's Alpha	Cronbach's Alpha Based on Standardized Items	N of Items
.845	.852	5

Men

Table (3.3.4.1.2) The Cronbach’s alpha for the factor is calculated based on the number of items.

Item Statistics

	Mean	Std. Deviation	N
I use the biology that I learn in school in my life.	3.63	.850	30
What I learn in my biology class helps me understand how things work in life.	4.40	.814	30
Learning biology makes me curious about things that I observe in my life.	4.13	.973	30
What we learn in biology class helps me to understand how biology affects my life.	4.20	.887	30
Learning biology helps me to make wiser decisions about my lifestyle and health.	4.10	.995	30

Women

Table (3.3.4.1.3) The statistics for the mean and standard deviation for each item in the factor in the questionnaire.

Item-Total Statistics

	Scale Mean if Item Deleted	Scale Variance if Item Deleted	Corrected Item-Total Correlation	Squared Multiple Correlation	Cronbach's Alpha if Item Deleted
I use the biology that I learn in school in my life.	16.83	9.661	.315	.326	.866
What I learn in my biology class helps me understand how things work in life.	16.07	7.789	.793	.803	.743
Learning biology makes me curious about things that I observe in my life.	16.33	6.920	.817	.688	.724
What we learn in biology class helps me to understand how biology affects my life.	16.27	8.064	.635	.773	.784
Learning biology helps me to make wiser decisions about my lifestyle and health.	16.37	7.826	.581	.409	.802

Women

Table (3.3.4.1.4) The total statistics for each item in the factor in the questionnaire.

Collaborative Learning

Case Processing Summary

		N	%
Cases	Valid	31	100.0
	Excluded[a]	0	.0
	Total	31	100.0

Women

Cases	Valid	32	100.0
	Excluded[a]	0	.0
	Total	32	100.0

Men

Table (3.3.4.2.1) Listwise deletion based on all variables in the procedure

Reliability Statistics

Cronbach's Alpha	Cronbach's Alpha Based on Standardized Items	N of Items
.962	.968	5

Women

Cronbach's Alpha	Cronbach's Alpha Based on Standardized Items	N of Items
.823	.835	5

Men

Table (3.3.4.2.2) The Cronbach's alpha for the factor is calculated based on the number of items.

Item Statistics

	Mean	Std. Deviation	N
I use the biology that I learn in school in my life.	4.68	.832	31

What I learn in my biology class helps me understand how things work in life.	4.84	.583	31
Learning biology makes me curious about things that I observe in my life.	4.77	.762	31
What we learn in biology class helps me to understand how biology affects my life.	4.84	.523	31
Learning biology helps me to make wiser decisions about my lifestyle and health.	4.77	.762	31

Women

	Mean	Std. Deviation	N
I use the biology that I learn in school in my life.	4.88	.336	32
What I learn in my biology class helps me understand how things work in life.	4.59	1.012	32
Learning biology makes me curious about things that I observe in my life.	4.66	.902	32
What we learn in biology class helps me to understand how biology affects my life.	4.72	.813	32
Learning biology helps me to make wiser decisions about my lifestyle and health.	4.75	.762	32

Men

Table (3.3.4.2.3) The statistics for the mean and standard deviation for each item in the factor in the questionnaire.

	Scale Mean if Item Deleted	Scale Variance if Item Deleted	Corrected Item-Total Correlation	Cronbach's Alpha if Item Deleted
I use the biology that I learn in school in my life.	19.23	6.381	.860	.963
What I learn in my biology class helps me understand how things work in life.	19.06	7.462	.907	.953
Learning biology makes me curious about things that I observe in my life.	19.13	6.383	.968	.939
What we learn in biology class helps me to understand how biology affects my life.	19.06	7.929	.845	.964
Learning biology helps me to make wiser decisions about my lifestyle and health.	19.13	6.383	.968	.939

Women

	Scale Mean if Item Deleted	Scale Variance if Item Deleted	Corrected Item-Total Correlation	Squared Multiple Correlation	Cronbach's Alpha if Item Deleted
I use the biology that I learn in school in my life.	18.72	8.209	.532	.697	.833
What I learn in my biology class helps me understand how things work in life.	19.00	4.581	.864	.949	.702
Learning biology makes me curious about things that I observe in my life.	18.94	5.480	.723	.945	.755

What we learn in biology class helps me to understand how biology affects my life.	18.88	6.758	.456	.922	.834
Learning biology helps me to make wiser decisions about my lifestyle and health.	18.84	6.201	.676	.828	.772

Men

Table (3.3.4.2.4) The total statistics for each item in the factor in the questionnaire.

TL vs CL

Group Statistics

	Method	N	Mean	Std. Deviation	Std. Error Mean
Benefit and Utility of biology	TL	30	3.9153	.59429	.10850
	CL	31	4.4026	.53669	.09639

Women

	Method	N	Mean	Std. Deviation	Std. Error Mean
Benefit and Utility of biology	TL	30	4.0717	.65328	.11927
	CL	32	4.3669	.49494	.08749

Men

Table (3.3.4.3.1) The group statistics for each item in the factors questionnaire with method.

Independent Samples Test

		Levene's Test for Equality of Variances		t-test for Equality of Means					95% Confidence Interval of the Difference	
		F	Sig.	t	df	Sig. (2-tailed)	Mean Difference	Std. Error Difference	Lower	Upper
Benefit and Utility of biology	Equal variances assumed	3.040	.086	-2.014	60	.049	-.29521	.14661	-.58848	-.00194
	Equal variances not assumed			-1.996	53.985	.051	-.29521	.14792	-.59178	.00136

Women

Independent Samples Test

		Levene's Test for Equality of Variances		t-test for Equality of Means					95% Confidence Interval of the Difference	
		F	Sig.	t	df	Sig. (2-tailed)	Mean Difference	Std. Error Difference	Lower	Upper
Benefit and Utility of biology	Equal variances assumed	3.040	.086	-2.014	60	.049	-.29521	.14661	-.58848	-.00194
	Equal variances not assumed			-1.996	53.985	.051	-.29521	.14792	-.59178	.00136

Men

Table (3.3.4.3.2) The independent samples test for factor to determine the F values and significance

Factor 5 Career Motivation

Traditional Learning

Case Processing Summary

		N	%
Cases	Valid	30	100.0
	Excluded[a]	0	.0
	Total	30	100.0

Women

		N	%
Cases	Valid	30	100.0
	Excluded[a]	0	.0
	Total	30	100.0

Men

Table (3.3.5.1.1) Listwise deletion based on all variables in the procedure

Reliability Statistics

Cronbach's Alpha	Cronbach's Alpha Based on Standardized Items	N of Items
.845	.865	5

Women

Cronbach's Alpha	Cronbach's Alpha Based on Standardized Items	N of Items
.874	.875	5

Men

Table (3.3.5.1.2) The Cronbach's alpha for the factor is calculated based on the number of items.

Item Statistics

	Mean	Std. Deviation	N
Learning biology will help me get a good job.	4.73	.640	30
Knowing biology will give me a career advantage.	4.57	.504	30
Understanding biology will benefit me in my career.	4.57	.568	30
My career will involve science.	4.63	.490	30
I will use biology problem-solving skills in my career	4.40	.675	30

Women

	Mean	Std. Deviation	N
Learning biology will help me get a good job.	4.33	.884	30
Knowing biology will give me a career advantage.	4.17	.747	30
Understanding biology will benefit me in my career.	4.10	.845	30
My career will involve science.	4.17	.791	30
I will use biology problem-solving skills in my career	4.13	.629	30

Men

Table (3.3.5.1.3) The statistics for the mean and standard deviation for each item in the factor in the questionnaire.

Item-Total Statistics

	Scale Mean if Item Deleted	Scale Variance if Item Deleted	Corrected Item-Total Correlation	Squared Multiple Correlation	Cronbach's Alpha if Item Deleted
Learning biology will help me get a good job.	18.17	3.661	.460	.343	.870
Knowing biology will give me a career advantage.	18.33	3.402	.828	.799	.774
Understanding biology will benefit me in my career.	18.33	3.195	.826	.833	.766
My career will involve science.	18.27	3.444	.832	.891	.775
I will use biology problem-solving skills in my career	18.50	3.569	.460	.320	.875

Women

	Scale Mean if Item Deleted	Scale Variance if Item Deleted	Corrected Item-Total Correlation	Squared Multiple Correlation	Cronbach's Alpha if Item Deleted
Learning biology will help me get a good job.	16.57	6.461	.665	.480	.860
Knowing biology will give me a career advantage.	16.73	6.547	.818	.731	.821
Understanding biology will benefit me in my career.	16.80	6.441	.717	.540	.845
My career will involve science.	16.73	6.340	.819	.738	.818
I will use biology problem-solving skills in my career	16.77	7.978	.523	.310	.886

Men

Table (3.3.5.1.4) The total statistics for each item in the factor in the questionnaire.

Collaborative Learning

Case Processing Summary

		N	%
Cases	Valid	31	100.0
	Excluded[a]	0	.0
	Total	31	100.0

Women

Cases	Valid	32	100.0
	Excluded[a]	0	.0
	Total	32	100.0

Men

Table (3.3.5.2.1) Listwise deletion based on all variables in the procedure

Reliability Statistics

Cronbach's Alpha	Cronbach's Alpha Based on Standardized Items	N of Items
.796	.788	4

Women

Cronbach's Alpha	Cronbach's Alpha Based on Standardized Items	N of Items
.898	.915	5

Men

Table (3.3.5.2.2) The Cronbach's alpha for the factor is calculated based on the number of items.

Item Statistics

	Mean	Std. Deviation	N
What I learn in my biology class helps me understand how things work in life.	4.77	.884	31

Learning biology makes me curious about things that I observe in my life.	4.87	.718	31
What we learn in biology class helps me to understand how biology affects my life.	4.87	.718	31
Learning biology helps me to make wiser decisions about my lifestyle and health.	4.87	.718	31

Women

	Mean	Std. Deviation	N
I use the biology that I learn in school in my life.	4.78	.751	32
What I learn in my biology class helps me understand how things work in life.	4.88	.707	32
Learning biology makes me curious about things that I observe in my life.	4.91	.530	32
What we learn in biology class helps me to understand how biology affects my life.	4.78	.870	32
Learning biology helps me to make wiser decisions about my lifestyle and health.	4.88	.492	32

Men

Table (3.3.5.2.3) The statistics for the mean and standard deviation for each item in the factor in the questionnaire.

	Scale Mean if Item Deleted	Scale Variance if Item Deleted	Corrected Item-Total Correlation	Cronbach's Alpha if Item Deleted
What I learn in my biology class helps me understand how things work in life.	14.61	2.512	.888	.575
Learning biology makes me curious about things that I observe in my life.	14.52	3.525	.644	.728
What we learn in biology class helps me to understand how biology affects my life.	14.52	4.325	.314	.871
Learning biology helps me to make wiser decisions about my lifestyle and health.	14.52	3.525	.644	.728

Women

	Scale Mean if Item Deleted	Scale Variance if Item Deleted	Corrected Item-Total Correlation	Cronbach's Alpha if Item Deleted
I use the biology that I learn in school in my life.	19.44	5.544	.640	.905
What I learn in my biology class helps me understand how things work in life.	19.34	4.943	.931	.836
Learning biology makes me curious about things that I observe in my life.	19.31	5.706	.941	.851
What we learn in biology class helps me to understand how biology affects my life.	19.44	4.770	.748	.890

Learning biology helps me to make wiser decisions about my lifestyle and health.	19.34	6.491	.653	.902

Men

Table (3.3.5.2.4) The total statistics for each item in the factor in the questionnaire.

TL vs CL

Group Statistics

	Method	N	Mean	Std. Deviation	Std. Error Mean
Career Motivation	TL	30	4.2817	.36861	.06730
	CL	31	4.8181	.41010	.10958

Women

	Method	N	Mean	Std. Deviation	Std. Error Mean
Career Motivation	TL	30	3.9277	.51870	.09470
	CL	32	4.8737	.57031	.10082

Men

Table (3.3.5.3.1) The group statistics for each item in the factors questionnaire with method.

Independent Samples Test

		Levene's Test for Equality of Variances		t-test for Equality of Means						
									95% Confidence Interval of the Difference	
		F	Sig.	t	df	Sig. (2-tailed)	Mean Difference	Std. Error Difference	Lower	Upper
Career Motivation	Equal variances assumed	.050	.825	-4.139	59	.000	-.53640	.12959	-.79572	-.27708
	Equal variances not assumed			-4.171	49.600	.000	-.53640	.12859	-.79474	-.27806

Women

Independent Samples Test

		Levene's Test for Equality of Variances		t-test for Equality of Means						
									95% Confidence Interval of the Difference	
		F	Sig.	t	df	Sig. (2-tailed)	Mean Difference	Std. Error Difference	Lower	Upper
Career Motivation	Equal variances assumed	2.635	.110	-6.819	60	.000	-.94608	.13875	-1.22362	-.66854
	Equal variances not assumed			-6.840	59.949	.000	-.94608	.13832	-1.22277	-.66940

Men

Table (3.3.5.3.2) The independent samples test for factors determine the F values and significance.

Factor 6 Self-Efficacy in Biology Learning

Traditional Learning

Case Processing Summary

		N	%
Cases	Valid	30	100.0
	Excluded[a]	0	.0
	Total	30	100.0

Women

		N	%
Cases	Valid	30	100.0
	Excluded[a]	0	.0
	Total	30	100.0

Men

Table (3.3.6.1.1) Listwise deletion based on all variables in the procedure

Reliability Statistics

Cronbach's Alpha	Cronbach's Alpha Based on Standardized Items	N of Items
.848	.851	8

Women

Cronbach's Alpha	Cronbach's Alpha Based on Standardized Items	N of Items
.837	.835	8

Men

Table (3.3.6.1.2) The Cronbach's alpha for the factor is calculated based on the number of items.

Item Statistics

	Mean	Std. Deviation	N
If I study hard I can do well in biology	3.8667	.81931	30
I believe biology is too easy for me to learn	3.7667	.85836	30
The idea of taking biology makes me excited.	3.4333	1.16511	30
I am confident I will do well on biology tests.	4.3333	1.02833	30
I am confident I will do well on biology labs and projects.	4.1667	1.14721	30
I believe I can master biology knowledge and skills.	4.4000	1.16264	30
I believe I can earn a grade of "A" in biology.	4.6333	.88992	30
I am sure I can understand biology.	4.6000	1.06997	30

Women

	Mean	Std. Deviation	N
If I study hard I can do well in biology	3.8667	.81931	30
I believe biology is too easy for me to learn	3.7667	.85836	30
The idea of taking biology makes me excited.	3.3667	1.09807	30
I am confident I will do well on biology tests.	4.0333	.92786	30

I am confident I will do well on biology labs and projects.	3.9333	1.01483		30
I believe I can master biology knowledge and skills.	4.2000	1.09545		30
I believe I can earn a grade of "A" in biology.	4.5667	.89763		30
I am sure I can understand biology.	4.5667	1.07265		30

Men

Table (3.3.6.1.3) The statistics for the mean and standard deviation for each item in the factor in the questionnaire.

Item-Total Statistics

	Scale Mean if Item Deleted	Scale Variance if Item Deleted	Corrected Item-Total Correlation	Squared Multiple Correlation	Cronbach's Alpha if Item Deleted
If I study hard I can do well in biology	29.3333	28.368	.414	.282	.848
I believe biology is too easy for me to learn	29.4333	25.151	.785	.693	.810
The idea of taking biology makes me excited.	29.7667	25.909	.454	.514	.848
I am confident I will do well on biology tests.	28.8667	27.016	.428	.435	.848
I am confident I will do well on biology labs and projects.	29.0333	25.068	.545	.497	.836
I believe I can master biology knowledge and skills.	28.8000	23.407	.701	.751	.814
I believe I can earn a grade of "A" in biology.	28.5667	26.668	.565	.563	.833
I am sure I can understand biology.	28.6000	22.731	.860	.860	.793

Women

	Scale Mean if Item Deleted	Scale Variance if Item Deleted	Corrected Item-Total Correlation	Squared Multiple Correlation	Cronbach's Alpha if Item Deleted
If I study hard I can do well in biology	28.4333	24.461	.432	.385	.833
I believe biology is too easy for me to learn	28.5333	22.051	.725	.649	.800
The idea of taking biology makes me excited.	28.9333	22.478	.475	.495	.832
I am confident I will do well on biology tests.	28.2667	24.616	.343	.435	.844
I am confident I will do well on biology labs and projects.	28.3667	22.516	.528	.452	.823
I believe I can master biology knowledge and skills.	28.1000	20.300	.722	.744	.795
I believe I can earn a grade of "A" in biology.	27.7333	23.513	.495	.555	.827
I am sure I can understand biology.	27.7333	19.513	.841	.850	.777

Men

Table (3.3.6.1.4) The total statistics for each item in the factor in the questionnaire.

Collaborative Learning

Case Processing Summary

		N	%
Cases	Valid	31	100.0
	Excluded[a]	0	.0
	Total	31	100.0

Women

Cases	Valid	32	100.0
	Excluded[a]	0	.0
	Total	32	100.0

Men

Table (3.3.6.2.1) Listwise deletion based on all variable in the procedure

Reliability Statistics

Cronbach's Alpha	Cronbach's Alpha Based on Standardized Items	N of Items
.889	.845	8

Women

Cronbach's Alpha	Cronbach's Alpha Based on Standardized Items	N of Items
.852	.857	8

Men

Table (3.3.6.2.2) The Cronbach's alpha for the factor is calculated based on the number of items.

Item Statistics

	Mean	Std. Deviation	N
If I study hard I can do well in biology	5.0323	.17961	31
I believe biology is too easy for me to learn	4.7419	.77321	31
The idea of taking biology makes me excited.	4.8387	.63754	31
I am confident I will do well on biology tests.	4.8065	.79244	31
I am confident I will do well on biology labs and projects.	4.7419	.63075	31
I believe I can master biology knowledge and skills.	4.7097	.78288	31
I believe I can earn a grade of "A" in biology.	4.8065	.40161	31
I am sure I can understand biology.	4.8387	.82044	31

Women

	Mean	Std. Deviation	N
If I study hard I can do well in biology	4.6250	.70711	32
I believe biology is too easy for me to learn	4.3750	.90696	32
The idea of taking biology makes me excited.	4.5313	.71772	32
I am confident I will do well on biology tests.	4.2188	1.18415	32
I am confident I will do well on biology labs and projects.	4.3125	.89578	32
I believe I can master biology knowledge and skills.	4.0938	1.20106	32

I believe I can earn a grade of “A” in biology.	4.3750	.79312		32
I am sure I can understand biology.	4.0625	1.18967		32

Men

Table (3.3.6.2.3) The statistics for the mean and standard deviation for each item in the factor in the questionnaire

Item-Total Statistics

	Scale Mean if Item Deleted	Scale Variance if Item Deleted	Corrected Item-Total Correlation	Squared Multiple Correlation	Cronbach's Alpha if Item Deleted
If I study hard I can do well in biology	33.4839	15.658	.071	.021	.908
I believe biology is too easy for me to learn	33.7742	10.981	.822	.807	.857
The idea of taking biology makes me excited.	33.6774	11.892	.794	.696	.862
I am confident I will do well on biology tests.	33.7097	10.746	.850	.796	.854
I am confident I will do well on biology labs and projects.	33.7742	11.847	.817	.771	.860
I believe I can master biology knowledge and skills.	33.8065	10.695	.876	.839	.851
I believe I can earn a grade of “A” in biology.	33.7097	15.880	-.078	.200	.921
I am sure I can understand biology.	33.6774	10.559	.855	.804	.853

Women

	Scale Mean if Item Deleted	Scale Variance if Item Deleted	Corrected Item-Total Correlation	Squared Multiple Correlation	Cronbach's Alpha if Item Deleted

If I study hard I can do well in biology	29.9688	25.193	.551	.421	.841
I believe biology is too easy for me to learn	30.2188	22.564	.722	.730	.820
The idea of taking biology makes me excited.	30.0625	23.415	.817	.769	.817
I am confident I will do well on biology tests.	30.3750	21.468	.614	.684	.834
I am confident I will do well on biology labs and projects.	30.2813	23.370	.627	.574	.831
I believe I can master biology knowledge and skills.	30.5000	19.935	.767	.715	.810
I believe I can earn a grade of "A" in biology.	30.2188	27.660	.158	.406	.876
I am sure I can understand biology.	30.5313	21.547	.601	.594	.836

Men

Table (3.3.6.2.4) The total statistics for each item in the factor in the questionnaire.

TL vs CL

Group Statistics

	Method	N	Mean	Std. Deviation	Std. Error Mean
Career Motivation	TL	30	3.9843	.71575	.13068
	CL	31	4.8074	.48765	.08758

Women

	Method	N	Mean	Std. Deviation	Std. Error Mean
Career Motivation	TL	30	3.8773	.66008	.12051
	CL	32	4.4091	.61818	.10928

Men

Table (3.3.6.3.1) The group statistics for each item in the factors Questionnaire with method.

Independent Samples Test

		Levene's Test for Equality of Variances		t-test for Equality of Means						
									95% Confidence Interval of the Difference	
		F	Sig.	t	df	Sig. (2-tailed)	Mean Difference	Std. Error Difference	Lower	Upper
Self-Efficacy in biology Learning	Equal variances assumed	2.495	.120	-5.264	59	.000	-.82309	.15636	-1.13595	-.51022
	Equal variances not assumed			-5.232	50.965	.000	-.82309	.15731	-1.13891	-.50726

Women

Independent Samples Test

		Levene's Test for Equality of Variances		t-test for Equality of Means						
									95% Confidence Interval of the Difference	
		F	Sig.	t	df	Sig. (2-tailed)	Mean Difference	Std. Error Difference	Lower	Upper
Self-Efficacy in biology Learning	Equal variances assumed	.520	.474	-3.276	60	.002	-.53173	.16233	-.85644	-.20701
	Equal variances not assumed			-3.269	58.988	.002	-.53173	.16268	-.85726	-.20620

Men

Table (3.3.6.3.2) The independent samples test for factor in the questionnaire to determine the F values and significance.

Factor 7 Self-Determination

Traditional Learning

Case Processing Summary

		N	%
Cases	Valid	30	100.0
	Excluded[a]	0	.0
	Total	30	100.0

Women

		N	%
Cases	Valid	30	100.0
	Excluded[a]	0	.0
	Total	30	100.0

Men

Table (3.3.7.1.1) Listwise deletion based on all variables in the procedure

Reliability Statistics

Cronbach's Alpha	Cronbach's Alpha Based on Standardized Items	N of Items
.757	.757	5

Women

Cronbach's Alpha	Cronbach's Alpha Based on Standardized Items	N of Items
.716	.717	5

Men

Table (3.3.7.1.2) The Cronbach's alpha for the factor is calculated based on the number of items.

Item Statistics

	Mean	Std. Deviation	N
I put enough effort into learning biology.	4.03	1.129	30
I use strategies to learn biology well.	3.90	1.125	30
I spend a lot of time learning biology.	3.97	.890	30
I prepare well for biology tests and labs.	4.20	1.031	30
I study hard to learn biology.	4.47	.860	30

Women

	Mean	Std. Deviation	N
I put enough effort into learning biology.	3.77	1.073	30
I use strategies to learn biology well.	3.47	1.196	30
I spend a lot of time learning biology.	3.40	1.037	30
I prepare well for biology tests and labs.	3.43	1.278	30
I study hard to learn biology.	3.77	1.104	30

Men

Table (3.3.7.1.3) The statistics for the mean and standard deviation for each item in the factor in the questionnaire.

Item-Total Statistics

	Scale Mean if Item Deleted	Scale Variance if Item Deleted	Corrected Item-Total Correlation	Squared Multiple Correlation	Cronbach's Alpha if Item Deleted
I put enough effort into learning biology.	16.53	8.051	.576	.631	.694

I use strategies to learn biology well.	16.67	7.747	.639	.646	.667
I spend a lot of time learning biology.	16.60	9.490	.498	.286	.723
I prepare well for biology tests and labs.	16.37	9.275	.426	.542	.748
I study hard to learn biology.	16.10	9.610	.499	.552	.724

Women

	Scale Mean if Item Deleted	Scale Variance if Item Deleted	Corrected Item-Total Correlation	Squared Multiple Correlation	Cronbach's Alpha if Item Deleted
I put enough effort into learning biology.	14.07	10.202	.568	.547	.633
I use strategies to learn biology well.	14.37	9.344	.612	.593	.609
I spend a lot of time learning biology.	14.43	11.289	.414	.269	.692
I prepare well for biology tests and labs.	14.40	9.972	.451	.366	.681
I study hard to learn biology.	14.07	11.444	.346	.320	.717

Men

Table (3.3.7.1.4) The total statistics for each item in the factor in the questionnaire.

Collaborative Learning

Case Processing Summary

		N	%
Cases	Valid	31	100.0
	Excluded[a]	0	.0
	Total	31	100.0

Women

Cases	Valid	32	100.0
	Excluded[a]	0	.0
	Total	32	100.0

Men

Table (3.3.7.2.1) Listwise deletion based on all variable in the procedure

Reliability Statistics

Cronbach's Alpha	Cronbach's Alpha Based on Standardized Items	N of Items
.817	.809	5

Women

Cronbach's Alpha	Cronbach's Alpha Based on Standardized Items	N of Items
.828	.815	5

Men

Table (3.3.7.2.2) The Cronbach's alpha for the factor is calculated based on the number of items.

Item Statistics

	Mean	Std. Deviation	N
I use the biology that I learn in school in my life.	4.84	.638	31
What I learn in my biology class helps me understand how things work in life.	4.68	.832	31
Learning biology makes me curious about things that I observe in my life.	4.68	.702	31
What we learn in biology class helps me to understand how biology affects my life.	4.61	.919	31
Learning biology helps me to make wiser decisions about my lifestyle and health.	4.68	.702	31

Women

	Mean	Std. Deviation	N
I use the biology that I learn in school in my life.	4.84	.628	32
What I learn in my biology class helps me understand how things work in life.	4.44	.840	32
Learning biology makes me curious about things that I observe in my life.	4.47	.718	32
What we learn in biology class helps me to understand how biology affects my life.	4.44	.914	32

Learning biology helps me to make wiser decisions about my lifestyle and health.	4.63	.707	32

Men

Table (3.3.7.2.3) The statistics for the mean and standard deviation for each item in the factor in the questionnaire.

Item-Total Statistics

	Scale Mean if Item Deleted	Scale Variance if Item Deleted	Corrected Item-Total Correlation	Squared Multiple Correlation	Cronbach's Alpha if Item Deleted
I use the biology that I learn in school in my life.	18.65	7.570	.137	.066	.889
What I learn in my biology class helps me understand how things work in life.	18.81	4.695	.852	.779	.698
Learning biology makes me curious about things that I observe in my life.	18.81	5.495	.751	.637	.742
What we learn in biology class helps me to understand how biology affects my life.	18.87	4.983	.641	.573	.776
Learning biology helps me to make wiser decisions about my lifestyle and health.	18.81	5.561	.726	.559	.749

Women

	Scale Mean if Item Deleted	Scale Variance if Item Deleted	Corrected Item-Total Correlation	Squared Multiple Correlation	Cronbach's Alpha if Item Deleted
I use the biology that I learn in school in my life.	17.97	8.096	.069	.058	.912

What I learn in my biology class helps me understand how things work in life.	18.38	4.823	.870	.819	.713
Learning biology makes me curious about things that I observe in my life.	18.34	5.523	.800	.707	.746
What we learn in biology class helps me to understand how biology affects my life.	18.38	5.081	.685	.641	.778
Learning biology helps me to make wiser decisions about my lifestyle and health.	18.19	5.641	.773	.638	.754

Men

Table (3.3.6.2.4) The total statistics for each item in the factor in the questionnaire.

TL vs CL

Group Statistics

	Method	N	Mean	Std. Deviation	Std. Error Mean
Self-Determination	TL	30	4.1240	.70190	.12815
	CL	31	4.6965	.57635	.10352

Women

	Method	N	Mean	Std. Deviation	Std. Error Mean
Self-Determination	TL	30	3.5767	.76926	.14045
	CL	31	4.5445	.58452	.10498

Men

Table (3.3.7.3.1) The group statistics for each item in the factor's questionnaire with method.

Independent Samples Test

		Levene's Test for Equality of Variances		t-test for Equality of Means						
									95% Confidence Interval of the Difference	
		F	Sig.	t	df	Sig. (2-tailed)	Mean Difference	Std. Error Difference	Lower	Upper
Self-Determination	Equal variances assumed	4.183	.045	-3.486	59	.001	-.57245	.16420	-.90102	-.24389
	Equal variances not assumed			-3.475	56.103	.001	-.57245	.16473	-.90244	-.24246

Women

Independent Samples Test

		Levene's Test for Equality of Variances		t-test for Equality of Means						
									95% Confidence Interval of the Difference	
		F	Sig.	t	df	Sig. (2-tailed)	Mean Difference	Std. Error Difference	Lower	Upper
Grade Motivation	Equal variances assumed	3.906	.053	-5.544	59	.000	-.96785	.17457	-1.31716	-.61854
	Equal variances not assumed			-5.520	54.126	.000	-.96785	.17535	-1.31938	-.61632

Men

Table (3.3.7.3.2) The independent samples test for factor to determine the F values and significance.

Factor 8 Grade Motivation

Traditional Learning

Case Processing Summary

		N	%
Cases	Valid	30	100.0
	Excluded[a]	0	.0
	Total	30	100.0

Women

		N	%
Cases	Valid	30	100.0
	Excluded[a]	0	.0
	Total	30	100.0

Men

Table (3.3.8.1.1) Listwise deletion based on all variables in the procedure

Reliability Statistics

Cronbach's Alpha	Cronbach's Alpha Based on Standardized Items	N of Items
.721	.726	5

Women

Cronbach's Alpha	Cronbach's Alpha Based on Standardized Items	N of Items
.834	.836	5

Men

Table (3.3.8.1.2) The Cronbach's alpha for the factor is calculated based on the number of items.

Item Statistics

	Mean	Std. Deviation	N
I like to do better than other students on biology tests.	4.00	.871	30
Getting a good biology grade is important to me.	4.07	.944	30
It is important that I get an "A" in biology.	4.20	.887	30
I think about the grade I will get in biology.	4.37	.964	30

Scoring high on biology tests and labs matters to me.	3.97	.999	30

Women

	Mean	Std. Deviation	N
I like to do better than other students on biology tests.	3.73	.907	30
Getting a good biology grade is important to me.	3.77	1.165	30
It is important that I get an "A" in biology.	3.83	1.117	30
I think about the grade I will get in biology.	4.10	1.094	30
Scoring high on biology tests and labs matters to me.	3.67	1.093	30

Men

Table (3.3.8.1.3) The statistics for the mean and standard deviation for each item in the factor in the questionnaire.

Item-Total Statistics

	Scale Mean if Item Deleted	Scale Variance if Item Deleted	Corrected Item-Total Correlation	Squared Multiple Correlation	Cronbach's Alpha if Item Deleted
I like to do better than other students on biology tests.	16.60	6.800	.607	.629	.626
Getting a good biology grade is important to me.	16.53	6.602	.582	.546	.632
It is important that I get an "A" in biology.	16.40	7.283	.470	.248	.678
I think about the grade I will get in biology.	16.23	7.220	.418	.475	.699
Scoring high on biology tests and labs matters to me.	16.63	7.413	.350	.536	.728

Women

	Scale Mean if Item Deleted	Scale Variance if Item Deleted	Corrected Item-Total Correlation	Squared Multiple Correlation	Cronbach's Alpha if Item Deleted
I like to do better than other students on biology tests.	15.37	12.378	.669	.663	.795
Getting a good biology grade is important to me.	15.33	10.575	.731	.684	.771
It is important that I get an "A" in biology.	15.27	11.168	.678	.490	.787
I think about the grade I will get in biology.	15.00	11.793	.597	.647	.811
Scoring high on biology tests and labs matters to me.	15.43	12.323	.515	.564	.833

Men

Table (3.3.8.1.4) The total statistics for each item in the factor in the questionnaire.

Collaborative Learning

Case Processing Summary

		N	%
Cases	Valid	31	100.0
	Excluded[a]	0	.0
	Total	31	100.0

Women

Cases	Valid	32	100.0
	Excluded[a]	0	.0
	Total	32	100.0

Men

Table (3.3.8.2.1) Listwise deletion based on all variable in the procedure

Reliability Statistics

Cronbach's Alpha	Cronbach's Alpha Based on Standardized Items	N of Items
.747	.812	5

Women

Cronbach's Alpha	Cronbach's Alpha Based on Standardized Items	N of Items
.792	.795	5

Men

Table (3.3.8.2.2) The Cronbach's alpha for the factor is calculated based on the number of items.

Item Statistics

	Mean	Std. Deviation	N
I use the biology that I learn in school in my life.	4.58	.720	31
What I learn in my biology class helps me understand how things work in life.	4.84	.638	31
Learning biology makes me curious about things that I observe in my life.	4.90	.539	31
What we learn in biology class helps me to understand how biology affects my life.	4.94	.359	31
Learning biology helps me to make wiser decisions about my lifestyle and health.	4.26	.965	31

Women

	Mean	Std. Deviation	N
I use the biology that I learn in school in my life.	4.75	.568	32
What I learn in my biology class helps me understand how things work in life.	4.88	.554	32
Learning biology makes me curious about things that I observe in my life.	4.91	.530	32
What we learn in biology class helps me to understand how biology affects my life.	4.91	.390	32

Learning biology helps me to make wiser decisions about my lifestyle and health.	4.88	.421	32

Men

Table (3.3.8.2.3) The statistics for the mean and standard deviation for each item in the factor in the questionnaire.

	Scale Mean if Item Deleted	Scale Variance if Item Deleted	Corrected Item-Total Correlation	Cronbach's Alpha if Item Deleted
I use the biology that I learn in school in my life.	18.94	3.929	.424	.737
What I learn in my biology class helps me understand how things work in life.	18.68	3.559	.704	.633
Learning biology makes me curious about things that I observe in my life.	18.61	4.045	.610	.679
What we learn in biology class helps me to understand how biology affects my life.	18.58	4.518	.662	.700
Learning biology helps me to make wiser decisions about my lifestyle and health.	19.26	3.198	.443	.773

Women

	Scale Mean if Item Deleted	Scale Variance if Item Deleted	Corrected Item-Total Correlation	Squared Multiple Correlation	Cronbach's Alpha if Item Deleted

I use the biology that I learn in school in my life.	19.56	2.190	.518	.827	.775
What I learn in my biology class helps me understand how things work in life.	19.44	1.996	.691	.932	.710
Learning biology makes me curious about things that I observe in my life.	19.41	2.055	.688	.937	.711
What we learn in biology class helps me to understand how biology affects my life.	19.41	2.314	.773	.828	.706
Learning biology helps me to make wiser decisions about my lifestyle and health.	19.44	2.835	.262	.819	.834

Men

Table (3.3.8.2.4) The total statistics for each item in the factor in the questionnaire.

TL vs CL

Group Statistics

	Method	N	Mean	Std. Deviation	Std. Error Mean
Self-Determination	TL	30	4.0950	.65347	.11931
	CL	31	4.7071	.46654	.08379

Women

	Method	N	Mean	Std. Deviation	Std. Error Mean
Self-Determination	TL	30	3.7733	.63649	.15272
	CL	32	4.8619	.59585	.06450

Men

Table (3.3.8.3.1) The group statistics for each item in the factor's questionnaire with method.

Independent Samples Test

		Levene's Test for Equality of Variances		t-test for Equality of Means						
									95% Confidence Interval of the Difference	
		F	Sig.	t	df	Sig. (2-tailed)	Mean Difference	Std. Error Difference	Lower	Upper
Grade Motivation	Equal variances assumed	4.942	.030	-4.221	59	.000	-.61210	.14501	-.90225	-.32194
	Equal variances not assumed			-4.198	52.352	.000	-.61210	.14579	-.90460	-.31959

Women

Independent Samples Test

		Levene's Test for Equality of Variances		t-test for Equality of Means					95% Confidence Interval of the Difference	
		F	Sig.	t	df	Sig. (2-tailed)	Mean Difference	Std. Error Difference	Lower	Upper
Grade Motivation	Equal variances assumed	8.526	.005	-6.714	60	.000	-1.08854	.16212	-1.41283	-.76425
	Equal variances not assumed			-6.566	39.103	.000	-1.08854	.16578	-1.42384	-.75325

Men

Table (3.3.8.3.2) The independent samples test for the factor to determine the F values and significance.

Factor 9 Assessment anxiety

Traditional Learning

Case Processing Summary

		N	%
Cases	Valid	30	100.0
	Excluded[a]	0	.0
	Total	30	100.0

Women

		N	%
Cases	Valid	30	100.0
	Excluded[a]	0	.0
	Total	30	100.0

Men

Table (3.3.9.1.1) Listwise deletion based on all variables in the procedure

Reliability Statistics

Cronbach's Alpha	Cronbach's Alpha Based on Standardized Items	N of Items
.809	.816	5

Women

Cronbach's Alpha	Cronbach's Alpha Based on Standardized Items	N of Items
.801	.792	5

Men

Table (3.3.9.1.2) The Cronbach's alpha for the factor is calculated based on the number of items.

Item Statistics

	Mean	Std. Deviation	N
I am nervous about how I will do on the biology tests.	3.30	1.317	30
I become anxious when it is time to take a biology test.	4.13	1.137	30
I worry about failing the biology tests.	4.17	1.206	30
I am concerned that the other students are better in biology.	4.37	1.217	30
I hate taking the biology tests.	2.97	1.159	30

Women

	Mean	Std. Deviation	N
I am nervous about how I will do on the biology tests.	3.27	1.363	30
I become anxious when it is time to take a biology test.	4.00	1.259	30
I worry about failing the biology tests.	3.93	1.437	30
I am concerned that the other students are better in biology.	4.10	1.470	30
I hate taking the biology tests.	2.97	1.159	30

Men

Table (3.3.9.1.3) The statistics for the mean and standard deviation for each item in the factor in the questionnaire.

Item-Total Statistics

	Scale Mean if Item Deleted	Scale Variance if Item Deleted	Corrected Item-Total Correlation	Squared Multiple Correlation	Cronbach's Alpha if Item Deleted
I am nervous about how I will do on the biology tests.	15.63	11.689	.561	.417	.698
I become anxious when it is time to take a biology test.	14.80	12.441	.592	.473	.689
I worry about failing the biology tests.	14.77	11.978	.605	.862	.682
I am concerned that the other students are better in biology.	14.57	11.426	.677	.897	.654
I hate taking the biology tests.	15.97	15.206	.213	.370	.811

Women

	Scale Mean if Item Deleted	Scale Variance if Item Deleted	Corrected Item-Total Correlation	Squared Multiple Correlation	Cronbach's Alpha if Item Deleted
I am nervous about how I will do on the biology tests.	15.00	16.138	.649	.530	.742
I become anxious when it is time to take a biology test.	14.27	17.444	.577	.430	.766
I worry about failing the biology tests.	14.33	15.195	.700	.914	.724
I am concerned that the other students are better in biology.	14.17	14.489	.755	.934	.703
I hate taking the biology tests.	15.30	21.045	.255	.385	.847

Men

Table (3.3.9.1.4) The total statistics for each item in the factor in the questionnaire.

Collaborative Learning

Case Processing Summary

		N	%
Cases	Valid	31	100.0
	Excluded[a]	0	.0
	Total	31	100.0

Women

Cases	Valid	32	100.0
	Excluded[a]	0	.0
	Total	32	100.0

Men

Table (3.3.9.2.1) Listwise deletion based on all variable in the procedure

Reliability Statistics

Cronbach's Alpha	Cronbach's Alpha Based on Standardized Items	N of Items
.805	.817	5

Women

Cronbach's Alpha	Cronbach's Alpha Based on Standardized Items	N of Items
.870	.872	5

Men

Table (3.3.9.2.2) The Cronbach's alpha for the factor is calculated based on the number of items.

Item Statistics

	Mean	Std. Deviation	N
I am nervous about how I will do on the biology tests.	4.52	.724	31
I become anxious when it is time to take a biology test.	4.61	.844	31
I worry about failing the biology tests.	4.77	.762	31
I am concerned that the other students are better in biology.	4.84	.638	31
I hate taking the biology tests.	4.55	.850	31

Women

	Mean	Std. Deviation	N
I am nervous about how I will do on the biology tests.	4.25	.718	32
I become anxious when it is time to take a biology test.	4.31	.896	32
I worry about failing the biology tests.	4.38	1.008	32
I am concerned that the other students are better in biology.	4.47	.842	32
I hate taking the biology tests.	4.38	.976	32

Men

Table (3.3.9.2.3) The statistics for the mean and standard deviation for each item in the factor in the questionnaire.

Item-Total Statistics

	Scale Mean if Item Deleted	Scale Variance if Item Deleted	Corrected Item-Total Correlation	Squared Multiple Correlation	Cronbach's Alpha if Item Deleted
I am nervous about how I will do on the biology tests.	18.77	6.314	.396	.288	.822
I become anxious when it is time to take a biology test.	18.68	4.692	.787	.704	.698
I worry about failing the biology tests.	18.52	4.991	.795	.912	.701
I am concerned that the other students are better in biology.	18.45	5.389	.839	.917	.705
I hate taking the biology tests.	18.74	6.398	.270	.095	.870

Women

	Scale Mean if Item Deleted	Scale Variance if Item Deleted	Corrected Item-Total Correlation	Squared Multiple Correlation	Cronbach's Alpha if Item Deleted
I am nervous about how I will do on the biology tests.	17.53	10.193	.531	.367	.878
I become anxious when it is time to take a biology test.	17.47	8.386	.762	.631	.826
I worry about failing the biology tests.	17.41	7.668	.799	.724	.815
I am concerned that the other students are better in biology.	17.31	8.157	.890	.806	.796
I hate taking the biology tests.	17.41	9.023	.541	.506	.884

Men

Table (3.3.9.2.4) The total statistics for each item in the factor in the questionnaire.

TL vs CL

Group Statistics

	Method	N	Mean	Std. Deviation	Std. Error Mean
Self-Determination	TL	30	3.8003	.55457	.15420
	CL	31	4.6716	.57846	.10390

Women

	Method	N	Mean	Std. Deviation	Std. Error Mean
Self-Determination	TL	30	3.6643	.59441	.17882
	CL	32	4.3578	.52702	.12852

Men

Table (3.3.9.3.1) The group statistics for each item in the factor's questionnaire with method.

Independent Samples Test

		Levene's Test for Equality of Variances		t-test for Equality of Means						
									95% Confidence Interval of the Difference	
		F	Sig.	t	df	Sig. (2-tailed)	Mean Difference	Std. Error Difference	Lower	Upper
Aassessment anxiety	Equal variances assumed	4.471	.039	-4.714	59	.000	-.87128	.18482	-1.24109	-.50146
	Equal variances not assumed			-4.686	51.123	.000	-.87128	.18593	-1.24453	-.49803

Women

Independent Samples Test

		Levene's Test for Equality of Variances		t-test for Equality of Means						
									95% Confidence Interval of the Difference	
		F	Sig.	t	df	Sig. (2-tailed)	Mean Difference	Std. Error Difference	Lower	Upper
Aassessment anxiety	Equal variances assumed	1.543	.219	-3.179	60	.002	-.69348	.21813	-1.12980	-.25716
	Equal variances not assumed			-3.149	53.375	.003	-.69348	.22021	-1.13509	-.25187

Men

Table (3.3.9.3.2) The independent samples test for factor in questionnaire to determine the F values and significance.

All factors comparison

Group Statistics

	Method	N	Mean	Std. Deviation	Std. Error Mean
Attitude_9 Factors	TL	270	4.0821	.64667	.03936
	CL	278	4.7001	.53671	.03219

Women

	Method	N	Mean	Std. Deviation	Std. Error Mean
Attitude_9 Factors	TL	270	3.8020	.65679	.04606
	CL	288	4.4970	.52199	.03665

Men

Table (3.3.10.3.1) The group statistics for each item in the factor's questionnaire with method

Independent Samples Test

		Levene's Test for Equality of Variances		t-test for Equality of Means						
									95% Confidence Interval of the Difference	
		F	Sig.	t	df	Sig. (2-tailed)	Mean Difference	Std. Error Difference	Lower	Upper
Attitude_9 Factors	Equal variances assumed	19.338	.000	-12.187	546	.000	-.61796	.05071	-.71756	-.51836
	Equal variances not assumed			-12.154	522.304	.000	-.61796	.05084	-.71784	-.51808

Women

Independent Samples Test

		Levene's Test for Equality of Variances		t-test for Equality of Means						
									95% Confidence Interval of the Difference	
		F	Sig.	t	df	Sig. (2-tailed)	Mean Difference	Std. Error Difference	Lower	Upper
Attitude_9 Factors	Equal variances assumed	13.378	.000	-11.882	556	.000	-.69502	.05849	-.80991	-.58012
	Equal variances not assumed			-11.808	521.541	.000	-.69502	.05886	-.81065	-.57938

Men

Table (3.3.10.3.2) The independent samples test for all factor in questionnaire to determine the F values and significance.

Attitude Women TL (All factors) VS Men TL (All factors)

Estimates

Dependent Variable: Attitude_9 Factors

Gender	Mean	Std. Error	95% Confidence Interval Lower Bound	Upper Bound
Women	3.700	.038	3.625	3.775
Men	2.539	.038	2.465	2.614

Table (3.3.10.3.3) Estimates for students in TL

Between-Subjects Factors

		Value Label	N
Gender	1.00	Women	269
	2.00	Men	270
Method	1.00	TL	539

Table (3.3.10.3.4) The subject factor based on gender and method

Descriptive Statistics

Dependent Variable: Attitude_9 Factors

Gender	Method	Mean	Std. Deviation	N
Women	TL	3.7002	.64419	269
	Total	3.7002	.64419	269
Men	TL	2.5393	.60263	270
	Total	2.5393	.60263	270
Total	TL	3.1186	.85197	539
	Total	3.1186	.85197	539

Table (3.3.10.3.5) The mean and standard deviation of students in TL

Univariate Tests

Dependent Variable: Attitude_9 Factors

	Sum of Squares	df	Mean Square	F	Sig.	Partial Eta Squared
Contrast	181.603	1	181.603	466.819	.000	.465
Error	208.905	537	.389			

Table (3.3.10.3.6) The F tests the effect of Gender. This test is based on the linearly independent pairwise comparisons among the estimated marginal means.

Attitude Women CL (All factors) VS Men CL (All factors)

Table (3.3.10.3.7) Estimates for students in CL

Between-Subjects Factors

		Value Label	N
Gender	1.00	Women	279
	2.00	Men	288
Method	2.00	CL	567

Table (3.3.10.3.8.) The subject factor based on gender and method

Descriptive Statistics

Dependent Variable: Attitude_9 Factors

Gender	Method	Mean	Std. Deviation	N
Women	CL	4.1053	.64930	279
	Total	4.1053	.64930	279
Men	CL	3.2847	.59377	288
	Total	3.2847	.59377	288
Total	CL	3.6885	.74461	567
	Total	3.6885	.74461	567

Table (3.3.10.3.9) The mean and standard deviation of students in CL

Univariate Tests

Dependent Variable: Attitude_9 Factors

	Sum of Squares	df	Mean Square	F	Sig.	Partial Eta Squared
Contrast	95.424	1	95.424	246.876	.000	.304
Error	218.388	565	.387			

Table (3.3.10.3.10) The F tests the effect of Gender. This test is based on the linearly independent pairwise comparisons among the estimated marginal means.

Mixed Gender Students

Factor 1 Feelings towards Biology

Traditional Learning

		N	%
Cases	Valid	28	100.0
	Excluded[a]	0	.0
	Total	28	100.0

Women

		N	%
Cases	Valid	34	100.0
	Excluded[a]	0	.0
	Total	34	100.0

Men

Table (3.4.1.1.1) Listwise deletion based on all variables in the procedure.

Reliability Statistics

Cronbach's Alpha	Cronbach's Alpha Based on Standardized Items	N of Items
.850	.815	14

Women

Cronbach's Alpha	Cronbach's Alpha Based on Standardized Items	N of Items
.851	.851	14

Men

Table (3.4.1.1.2) The Cronbach's alpha for the factor is calculated based on the number of items.

Item Statistics

	Mean	Std. Deviation	N
Biology is very interesting to me.	4.21	1.031	28
I have always enjoyed studying biology in school.	4.46	.793	28
I am always under a terrible strain in a biology class.	4.50	.793	28
I feel a definite positive reaction to biology; it's enjoyable.	4.32	1.020	28
Biology makes me feel secure, and at the same time it is stimulating.	4.39	.956	28
I feel at ease in biology and like it very much.	4.18	1.188	28
In general, I have a good feeling toward biology.	4.25	1.041	28
I really like biology.	4.14	1.208	28
Biology is fascinating and fun.	4.57	.920	28
When I hear the word biology, I have a feeling of dislike.	4.14	1.177	28
I approach biology with a feeling of hesitation.	4.32	1.020	28
It makes me nervous to even think about doing a biology experiment.	4.64	.559	28
Biology makes me feel uncomfortable, restless, irritable, and impatient.	4.54	.744	28
I don't like biology, and it scares me to have to take it.	4.64	.559	28

Women

	Mean	Std. Deviation	N
Biology is very interesting to me.	4.79	.774	29
I have always enjoyed studying biology in school.	4.97	.186	29
I am always under a terrible strain in a biology class.	4.90	.310	29
I feel a definite positive reaction to biology; it's enjoyable.	4.83	.759	29
Biology makes me feel secure, and at the same time it is stimulating.	4.97	.186	29
I feel at ease in biology and like it very much.	4.28	1.162	29
In general, I have a good feeling toward biology.	4.41	.983	29
I really like biology.	4.28	1.192	29
Biology is fascinating and fun.	4.62	.862	29
When I hear the word biology, I have a feeling of dislike.	4.28	1.162	29
I approach biology with a feeling of hesitation.	4.41	.983	29
It makes me nervous to even think about doing a biology experiment.	4.86	.351	29
Biology makes me feel uncomfortable, restless, irritable, and impatient.	4.79	.620	29
I don't like biology, and it scares me to have to take it.	4.97	.186	29

Men

Table (3.4.1.1.3) The statistics for the mean and standard deviation for each item in the factor in the questionnaire.

Item-Total Statistics

	Scale Mean if Item Deleted	Scale Variance if Item Deleted	Corrected Item-Total Correlation	Squared Multiple Correlation	Cronbach's Alpha if Item Deleted
Biology is very interesting to me.	57.11	53.507	.365	.734	.848
I have always enjoyed studying biology in school.	56.86	59.683	-.019	.402	.865
I am always under a terrible strain in a biology class.	56.82	51.337	.713	.742	.829
I feel a definite positive reaction to biology; it's enjoyable.	57.00	48.889	.711	.840	.825
Biology makes me feel secure, and at the same time it is stimulating.	56.93	48.587	.794	.850	.821
I feel at ease in biology and like it very much.	57.14	44.794	.872	.911	.811
In general, I have a good feeling toward biology.	57.07	50.365	.584	.857	.834
I really like biology.	57.18	46.522	.734	.906	.822
Biology is fascinating and fun.	56.75	52.491	.506	.871	.839
When I hear the word biology, I have a feeling of dislike.	57.18	47.856	.665	.931	.828
I approach biology with a feeling of hesitation.	57.00	48.296	.757	.894	.822
It makes me nervous to even think about doing a biology experiment.	56.68	60.078	-.036	.802	.860

Biology makes me feel uncomfortable, restless, irritable, and impatient.	56.79	61.138	-.139	.761	.868
I don't like biology, and it scares me to have to take it.	56.68	60.078	-.036	.720	.860

Women

	Scale Mean if Item Deleted	Scale Variance if Item Deleted	Corrected Item-Total Correlation	Cronbach's Alpha if Item Deleted
Biology is very interesting to me.	60.55	35.185	.602	.835
I have always enjoyed studying biology in school.	60.38	39.815	.621	.848
I am always under a terrible strain in a biology class.	60.45	39.256	.503	.847
I feel a definite positive reaction to biology; it's enjoyable.	60.52	35.330	.598	.835
Biology makes me feel secure, and at the same time it is stimulating.	60.38	39.815	.621	.848
I feel at ease in biology and like it very much.	61.07	29.781	.802	.817
In general, I have a good feeling toward biology.	60.93	34.352	.520	.840
I really like biology.	61.07	29.209	.828	.814
Biology is fascinating and fun.	60.72	35.064	.538	.838
When I hear the word biology, I have a feeling of dislike.	61.07	29.352	.842	.813

I approach biology with a feeling of hesitation.	60.93	32.995	.651	.830
It makes me nervous to even think about doing a biology experiment.	60.48	41.330	-.033	.859
Biology makes me feel uncomfortable, restless, irritable, and impatient.	60.55	41.542	-.078	.867
I don't like biology, and it scares me to have to take it.	60.38	41.315	-.019	.857

Men

Table (3.4.1.1.4) The total statistics for each item in the factor in the questionnaire.

Collaborative Learning

Case Processing Summary

		N	%
Cases	Valid	32	100.0
	Excluded[a]	0	.0
	Total	32	100.0

Women

		N	%
Cases	Valid	32	100.0
	Excluded[a]	0	.0
	Total	32	100.0

Men

Table (3.4.1.2.1) Listwise deletion based on all variables in the procedure.

Reliability Statistics

Cronbach's Alpha	Cronbach's Alpha Based on Standardized Items	N of Items
.899	.892	14

Women

Cronbach's Alpha	Cronbach's Alpha Based on Standardized Items	N of Items
.868	.854	13

Men

Table (3.4.1.2.2) The Cronbach's alpha for the feelings towards biology factor is calculated based on the number of items

Item Statistics

	Mean	Std. Deviation	N
Biology is very interesting to me.	4.38	1.185	32
I have always enjoyed studying biology in school.	4.34	1.181	32
I am always under a terrible strain in a biology class.	4.31	1.176	32
I feel a definite positive reaction to biology; it's enjoyable.	4.25	1.078	32
Biology makes me feel secure, and at the same time it is stimulating.	4.00	.803	32
I feel at ease in biology and like it very much.	4.06	.669	32
In general, I have a good feeling toward biology.	4.06	.504	32
I really like biology.	3.91	.928	32

Biology is fascinating and fun.	4.00	.880	32
When I hear the word biology, I have a feeling of dislike.	4.13	1.129	32
I approach biology with a feeling of hesitation.	4.06	1.216	32
It makes me nervous to even think about doing a biology experiment.	4.25	.950	32
Biology makes me feel uncomfortable, restless, irritable, and impatient.	4.28	1.198	32
I don't like biology, and it scares me to have to take it.	4.06	1.268	32

	Women		
	Mean	Std. Deviation	N
Biology is very interesting to me.	4.78	.870	32
I have always enjoyed studying biology in school.	4.88	.554	32
I am always under a terrible strain in a biology class.	4.88	.554	32
I feel a definite positive reaction to biology; it's enjoyable.	4.78	.870	32
Biology makes me feel secure, and at the same time it is stimulating.	4.88	.336	32
I feel at ease in biology and like it very much.	4.28	1.198	32
In general, I have a good feeling toward biology.	4.47	.950	32
I really like biology.	4.50	1.078	32
Biology is fascinating and fun.	4.72	.813	32

When I hear the word biology, I have a feeling of dislike.	4.78	.792	32
I approach biology with a feeling of hesitation.	4.81	.644	32
It makes me nervous to even think about doing a biology experiment.	4.97	.177	32
Biology makes me feel uncomfortable, restless, irritable, and impatient.	4.97	.177	32

Men

Table (3.4.1.2.3) The statistics for the mean and standard deviation for each item in the factor in the questionnaire.

Item-Total Statistics

	Scale Mean if Item Deleted	Scale Variance if Item Deleted	Corrected Item-Total Correlation	Squared Multiple Correlation	Cronbach's Alpha if Item Deleted
Biology is very interesting to me.	53.72	73.886	.774	.994	.884
I have always enjoyed studying biology in school.	53.75	74.129	.764	.994	.884
I am always under a terrible strain in a biology class.	53.78	74.757	.733	.907	.886
I feel a definite positive reaction to biology; it's enjoyable.	53.84	79.943	.516	.895	.895
Biology makes me feel secure, and at the same time it is stimulating.	54.09	86.346	.272	.746	.903
I feel at ease in biology and like it very much.	54.03	84.483	.498	.696	.896
In general, I have a good feeling toward biology.	54.03	88.096	.286	.768	.902
I really like biology.	54.19	78.609	.704	.875	.888
Biology is fascinating and fun.	54.09	84.862	.334	.878	.901
When I hear the word biology, I have a feeling of dislike.	53.97	74.289	.796	.939	.883
I approach biology with a feeling of hesitation.	54.03	79.967	.442	.789	.900
It makes me nervous to even think about doing a biology experiment.	53.84	82.265	.457	.747	.897
Biology makes me feel uncomfortable, restless, irritable, and impatient.	53.81	73.190	.802	.934	.882
I don't like biology, and it scares me to have to take it.	54.03	72.870	.765	.682	.884

Women

	Scale Mean if Item Deleted	Scale Variance if Item Deleted	Corrected Item-Total Correlation	Cronbach's Alpha if Item Deleted
Biology is very interesting to me.	56.91	29.959	.734	.845
I have always enjoyed studying biology in school.	56.81	33.899	.543	.859
I am always under a terrible strain in a biology class.	56.81	33.899	.543	.859
I feel a definite positive reaction to biology; it's enjoyable.	56.91	29.959	.734	.845
Biology makes me feel secure, and at the same time it is stimulating.	56.81	35.512	.520	.864
I feel at ease in biology and like it very much.	57.41	25.410	.899	.830
In general, I have a good feeling toward biology.	57.22	31.660	.481	.863
I really like biology.	57.19	28.415	.708	.847
Biology is fascinating and fun.	56.97	33.773	.346	.870
When I hear the word biology, I have a feeling of dislike.	56.91	31.120	.674	.850
I approach biology with a feeling of hesitation.	56.88	33.661	.485	.861
It makes me nervous to even think about doing a biology experiment.	56.72	37.499	.081	.874
Biology makes me feel uncomfortable, restless, irritable, and impatient.	56.72	37.499	.081	.874

Men

Table (3.4.1.2.4) The total statistics for each item in the factor in the questionnaire.

TL vs CL

Group Statistics

	Method	N	Mean	Std. Deviation	Std. Error Mean
Feeling toward biology	TL	28	4.3079	.51232	.09682
	CL	32	4.1713	.66320	.11724

Women

	Method	N	Mean	Std. Deviation	Std. Error Mean
Feeling toward biology	TL	29	4.5410	.41001	.07614
	CL	32	4.6219	.39070	.06907

Men

Table (3.4.1.3.1) The group statistics for each item in the factors questionnaire with method.

Independent Samples Test

		Levene's Test for Equality of Variances		t-test for Equality of Means					95% Confidence Interval of the Difference	
		F	Sig.	t	df	Sig. (2-tailed)	Mean Difference	Std. Error Difference	Lower	Upper
Feeling toward biology	Equal variances assumed	2.713	.105	.883	58	.381	.13661	.15467	-.17301	.44622
	Equal variances not assumed			.898	57.171	.373	.13661	.15205	-.16784	.44106

Women

Independent Samples Test

		Levene's Test for Equality of Variances		t-test for Equality of Means					95% Confidence Interval of the Difference	
		F	Sig.	t	df	Sig. (2-tailed)	Mean Difference	Std. Error Difference	Lower	Upper
Feeling toward biology	Equal variances assumed	.202	.655	-.788	59	.434	-.08084	.10255	-.28604	.12436
	Equal variances not assumed			-.786	57.732	.435	-.08084	.10280	-.28663	.12495

Men

Table (3.4.1.3.2) The independent samples test for feelings towards biology questionnaire to determine the F values and significance.

Factor 2 General Interest

Traditional Learning

Case Processing Summary

		N	%
Cases	Valid	38	100.0
	Excluded[a]	0	.0
	Total	38	100.0

Women

		N	%
Cases	Valid	29	100.0
	Excluded[a]	0	.0
	Total	29	100.0

Men

Table (3.4.2.1.1) Listwise deletion based on all variables in the procedure.

Reliability Statistics

Cronbach's Alpha	Cronbach's Alpha Based on Standardized Items	N of Items
.821	.829	5

Women

Cronbach's Alpha	Cronbach's Alpha Based on Standardized Items	N of Items
.889	.896	5

Men

Table (3.4.2.1.2) The Cronbach's alpha for the factor is calculated based on the number of items.

Item Statistics

	Mean	Std. Deviation	N
I like watching biology related TV.	3.96	.793	28
biology is my favourite subject in school.	4.14	.705	28
I like reading about famous biologist	3.86	1.113	28
I find what we learn in my biology class interesting.	4.00	1.018	28
I would enjoy working in a biology lab.	4.07	.979	28

Women

	Mean	Std. Deviation	N
I like watching biology related TV.	4.17	.889	29
biology is my favorite subject in school.	4.28	.751	29
I like reading about famous biologiest	4.24	1.327	29
I find what we learn in my biology class interesting.	4.38	1.015	29
I would enjoy working in a biology lab.	4.48	.949	29

Men

Table (3.4.2.1.3) The statistics for the mean and standard deviation for each item in the factor in the questionnaire.

Item-Total Statistics

	Scale Mean if Item Deleted	Scale Variance if Item Deleted	Corrected Item-Total Correlation	Squared Multiple Correlation	Cronbach's Alpha if Item Deleted
I like watching biology related TV.	16.07	8.958	.657	.510	.778
biology is my favorite subject in school.	15.89	9.581	.601	.565	.796
I like reading about famous biologiest	16.18	7.560	.638	.584	.783
I find what we learn in my biology class interesting.	16.04	8.258	.582	.417	.797
I would enjoy working in a biology lab.	15.96	8.110	.652	.545	.774

Women

Table (3.4.2.1.4) The total statistics for each item in the factor in the questionnaire.

Collaborative Learning

Case Processing Summary

		N	%
Cases	Valid	32	100.0
	Excluded[a]	0	.0
	Total	32	100.0

Women

		N	%
Cases	Valid	32	100.0
	Excluded[a]	0	.0
	Total	32	100.0

Men

Table (3.4.2.2.1) Listwise deletion based on all variables in the procedure.

Reliability Statistics

Cronbach's Alpha	Cronbach's Alpha Based on Standardized Items	N of Items
.755	.742	5

Women

Cronbach's Alpha	Cronbach's Alpha Based on Standardized Items	N of Items
.765	.781	5

Men

Table (3.4.2.2.2) The Cronbach's alpha for the factor is calculated based on the number of items.

Item Statistics

	Mean	Std. Deviation	N
I like watching biology related TV.	3.84	.920	32
biology is my favorite subject in school.	3.41	1.103	32
I like reading about famous biologiest	3.50	.842	32
I find what we learn in my biology class interesting.	3.78	.751	32
I would enjoy working in a biology lab.	3.94	.669	32

Women

	Mean	Std. Deviation	N
I like watching biology related TV.	4.59	.798	32

biology is my favorite subject in school.	4.56	.669	32
I like reading about famous biologiest	4.63	.833	32
I find what we learn in my biology class interesting.	4.31	.859	32
I would enjoy working in a biology lab.	4.38	.942	32

Men

Table (3.4.2.1.3) The statistics for the mean and standard deviation for each item in the factor in the questionnaire.

Item-Total Statistics

	Scale Mean if Item Deleted	Scale Variance if Item Deleted	Corrected Item-Total Correlation	Squared Multiple Correlation	Cronbach's Alpha if Item Deleted
I like watching biology related TV.	14.63	5.855	.640	.480	.664
biology is my favorite subject in school.	15.06	4.770	.739	.574	.616
I like reading about famous biologist	14.97	6.741	.479	.293	.725
I find what we learn in my biology class interesting.	14.69	7.060	.482	.267	.725
I would enjoy working in a biology lab.	14.53	7.999	.291	.101	.777

Women

	Scale Mean if Item Deleted	Scale Variance if Item Deleted	Corrected Item-Total Correlation	Squared Multiple Correlation	Cronbach's Alpha if Item Deleted
I like watching biology related TV.	17.88	5.855	.591	.511	.703
biology is my favorite subject in school.	17.91	6.023	.701	.729	.679

I like reading about famous biologiest	17.84	6.007	.508	.482	.732
I find what we learn in my biology class interesting.	18.16	5.426	.652	.488	.679
I would enjoy working in a biology lab.	18.09	6.410	.309	.279	.810

Men

Table (3.4.2.2.4) The total statistics for each item in the factor in the questionnaire.

TL vs CL

Group Statistics

	Method	N	Mean	Std. Deviation	Std. Error Mean
General interest	TL	28	4.0036	.66569	.12580
	CL	32	3.7775	.63716	.11263

Women

	Method	N	Mean	Std. Deviation	Std. Error Mean
General interest	TL	29	4.2045	.74250	.13788
	CL	33	4.5524	.61812	.10760

Men

Table (3.4.2.3.1) The group statistics for each item in the factors questionnaire with method.

Independent Samples Test

		Levene's Test for Equality of Variances		t-test for Equality of Means						
									95% Confidence Interval of the Difference	
		F	Sig.	t	df	Sig. (2-tailed)	Mean Difference	Std. Error Difference	Lower	Upper
General interest	Equal variances assumed	.442	.509	1.343	58	.185	.22607	.16836	-.11093	.56308
	Equal variances not assumed			1.339	56.189	.186	.22607	.16886	-.11217	.56431

Women

Independent Samples Test

		Levene's Test for Equality of Variances		t-test for Equality of Means						
									95% Confidence Interval of the Difference	
		F	Sig.	t	df	Sig. (2-tailed)	Mean Difference	Std. Error Difference	Lower	Upper
General interest	Equal variances assumed	.040	.842	-2.013	60	.049	-.34794	.17283	-.69365	-.00223
	Equal variances not assumed			-1.989	54.729	.052	-.34794	.17490	-.69848	.00260

Men

Table (3.4.2.3.2) The independent samples test for feelings towards biology questionnaire to determine the F values and significance.

Factor 3 Motivation Towards Learning

Traditional Learning

Case Processing Summary

		N	%
Cases	Valid	30	100.0
	Excluded[a]	0	.0
	Total	30	100.0

Women

		N	%
Cases	Valid	29	100.0
	Excluded[a]	0	.0
	Total	29	100.0

Men

Table (3.4.3.1.1) Listwise deletion based on all variables in the procedure

Reliability Statistics

Cronbach's Alpha	Cronbach's Alpha Based on Standardized Items	N of Items
.872	.806	10

Women

Cronbach's Alpha	Cronbach's Alpha Based on Standardized Items	N of Items
.875	.862	10

Men

Table (3.4.3.1.2) The Cronbach's alpha for the factor is calculated based on the number of items.

Item Statistics

	Mean	Std. Deviation	N
I will ask my teacher for an explanation if I do not understand the science topic.	4.6786	.47559	28
I will look for an explanation in the textbook if I do not understand the science topic.	4.6786	.54796	28
I care about completing assignments in this class.	4.9643	.18898	28
Getting a good grade in biology is important to me.	4.3929	.49735	28
I am interested in understanding the teacher in this class.	4.3929	1.06595	28
The biology I learn is relevant to my life.	4.4286	1.10315	28
Learning biology is interesting.	4.1429	1.04401	28
Learning biology makes my life more meaningful.	4.4286	.87891	28
I am curious about discoveries in biology.	4.4643	.83808	28
I enjoy learning biology	4.2143	.78680	28

Women

	Mean	Std. Deviation	N

I will ask my teacher for an explanation if I do not understand the science topic.	4.1034	.55709	29
I will look for an explanation in the textbook if I do not understand the science topic.	4.1379	.63943	29
I care about completing assignments in this class.	4.2069	.61987	29
Getting a good grade in biology is important to me.	4.1034	.40925	29
I am interested in understanding the teacher in this class.	4.1724	1.03748	29
The biology I learn is relevant to my life.	4.2759	1.06558	29
Learning biology is interesting.	3.8621	1.02554	29
Learning biology makes my life more meaningful.	4.2759	.84077	29
I am curious about discoveries in biology.	4.2759	.84077	29
I enjoy learning biology	4.0345	.73108	29

Men

Table (3.4.3.1.3) The statistics for the mean and standard deviation for each item in the factor in the questionnaire.

Item-Total Statistics

	Scale Mean if Item Deleted	Scale Variance if Item Deleted	Corrected Item-Total Correlation	Squared Multiple Correlation	Cronbach's Alpha if Item Deleted
I will ask my teacher for an explanation if I do not understand the science topic.	40.1071	28.396	.160	.452	.883

I will look for an explanation in the textbook if I do not understand the science topic.	40.1071	27.062	.363	.637	.874
I care about completing assignments in this class.	39.8214	29.708	-.150	.325	.886
Getting a good grade in biology is important to me.	40.3929	29.136	.009	.441	.889
I am interested in understanding the teacher in this class.	40.3929	19.951	.877	.903	.831
The biology I learn is relevant to my life.	40.3571	19.868	.849	.898	.835
Learning biology is interesting.	40.6429	20.386	.844	.848	.835
Learning biology makes my life more meaningful.	40.3571	21.868	.826	.735	.838
I am curious about discoveries in biology.	40.3214	22.374	.802	.818	.841
I enjoy learning biology	40.5714	23.069	.760	.800	.846

Women

	Scale Mean if Item Deleted	Scale Variance if Item Deleted	Corrected Item-Total Correlation	Squared Multiple Correlation	Cronbach's Alpha if Item Deleted
I will ask my teacher for an explanation if I do not understand the science topic.	37.3448	29.091	.178	.169	.886
I will look for an explanation in the textbook if I do not understand the science topic.	37.3103	27.793	.336	.783	.879
I care about completing assignments in this class.	37.2414	28.333	.266	.798	.883

Getting a good grade in biology is important to me.	37.3448	28.091	.510	.543	.872
I am interested in understanding the teacher in this class.	37.2759	21.707	.795	.854	.845
The biology I learn is relevant to my life.	37.1724	21.505	.792	.864	.845
Learning biology is interesting.	37.5862	21.823	.793	.805	.845
Learning biology makes my life more meaningful.	37.1724	23.719	.738	.743	.851
I am curious about discoveries in biology.	37.1724	23.362	.788	.813	.847
I enjoy learning biology	37.4138	24.823	.702	.756	.855

Men

Table (3.4.3.1.4) The total statistics for each item in the factor in the questionnaire.

Collaborative Learning

Case Processing Summary

		N	%
Cases	Valid	32	100.0
	Excluded[a]	0	.0
	Total	32	100.0

Women

Cases	Valid	32	100.0
	Excluded[a]	0	.0
	Total	32	100.0

Men

Table (3.4.3.2.1) Listwise deletion based on all variables in the procedure

Reliability Statistics

Cronbach's Alpha	Cronbach's Alpha Based on Standardized Items	N of Items
.845	.840	10

Women

Cronbach's Alpha	Cronbach's Alpha Based on Standardized Items	N of Items
.877	.869	10

Men

Table (3.4.3.2.2) The Cronbach's alpha for the factor is calculated based on the number of items.

Item Statistics

	Mean	Std. Deviation	N
I will ask my teacher for an explanation if I do not understand the science topic.	4.1875	.78030	32
I will look for an explanation in the textbook if I do not understand the science topic.	4.0313	.96668	32
I care about completing assignments in this class.	3.7188	1.34966	32
Getting a good grade in biology is important to me.	3.9063	1.11758	32
I am interested in understanding the teacher in this class.	3.7188	1.19770	32
The biology I learn is relevant to my life.	3.8125	1.25563	32
Learning biology is interesting.	3.5000	1.21814	32
Learning biology makes my life more meaningful.	3.9063	.96250	32
I am curious about discoveries in biology.	3.8438	1.08090	32
I enjoy learning biology	3.7188	.88843	32

Women

	Mean	Std. Deviation	N
I will ask my teacher for an explanation if I do not understand the science topic.	4.5938	.71208	32

I will look for an explanation in the textbook if I do not understand the science topic.	4.7188	.68318	32
I care about completing assignments in this class.	4.6250	1.00803	32
Getting a good grade in biology is important to me.	4.7813	.60824	32
I am interested in understanding the teacher in this class.	4.5938	1.10306	32
The biology I learn is relevant to my life.	4.5938	1.10306	32
Learning biology is interesting.	4.4375	1.04534	32
Learning biology makes my life more meaningful.	4.6250	.79312	32
I am curious about discoveries in biology.	4.5625	.80071	32
I enjoy learning biology	4.5000	.67202	32

Men

Table (3.4.3.2.3) The statistics for the mean and standard deviation for each item in the factor in the questionnaire.

Item-Total Statistics

	Scale Mean if Item Deleted	Scale Variance if Item Deleted	Corrected Item-Total Correlation	Squared Multiple Correlation	Cronbach's Alpha if Item Deleted
I will ask my teacher for an explanation if I do not understand the science topic.	34.1563	47.233	.211	.351	.854
I will look for an explanation in the textbook if I do not understand the science topic.	34.3125	43.125	.476	.585	.837

I care about completing assignments in this class.	34.6250	37.597	.646	.506	.821
Getting a good grade in biology is important to me.	34.4375	40.835	.562	.699	.829
I am interested in understanding the teacher in this class.	34.6250	40.694	.522	.662	.833
The biology I learn is relevant to my life.	34.5313	38.064	.675	.742	.817
Learning biology is interesting.	34.8438	38.910	.639	.742	.821
Learning biology makes my life more meaningful.	34.4375	42.319	.548	.645	.831
I am curious about discoveries in biology.	34.5000	40.645	.602	.576	.825
I enjoy learning biology	34.6250	43.403	.505	.544	.835

Women

	Scale Mean if Item Deleted	Scale Variance if Item Deleted	Corrected Item-Total Correlation	Squared Multiple Correlation	Cronbach's Alpha if Item Deleted
I will ask my teacher for an explanation if I do not understand the science topic.	41.4375	33.609	.232	.521	.887
I will look for an explanation in the textbook if I do not understand the science topic.	41.3125	32.480	.396	.765	.878
I care about completing assignments in this class.	41.4063	25.926	.885	.910	.839
Getting a good grade in biology is important to me.	41.2500	31.161	.663	.899	.864
I am interested in understanding the teacher in this class.	41.4375	26.706	.711	.958	.856

The biology I learn is relevant to my life.	41.4375	26.060	.777	.969	.849
Learning biology is interesting.	41.5938	26.894	.742	.826	.852
Learning biology makes my life more meaningful.	41.4063	30.701	.535	.798	.869
I am curious about discoveries in biology.	41.4688	29.483	.679	.855	.859
I enjoy learning biology	41.5313	32.838	.356	.593	.880

Men

Table (3.4.3.2.4) The total statistics for each item in the factor in the questionnaire.

TL vs CL

Group Statistics

	Method	N	Mean	Std. Deviation	Std. Error Mean
Motivation Towards Learning Biology	TL	28	4.3350	.51381	.09710
	CL	32	3.9353	.69474	.12281

Women

	Method	N	Mean	Std. Deviation	Std. Error Mean
Motivation Towards Learning Biology	TL	29	4.1031	.52583	.09764
	CL	32	4.6278	.58573	.10354

Men

Table (3.4.3.3.1) The group statistics for each item in the factors questionnaire with method.

Independent Samples Test

		Levene's Test for Equality of Variances		t-test for Equality of Means						
									95% Confidence Interval of the Difference	
		F	Sig.	t	df	Sig. (2-tailed)	Mean Difference	Std. Error Difference	Lower	Upper
Motivation Towards Learning Biology	Equal variances assumed	7.930	.007	2.503	58	.015	.39969	.15970	.08001	.71936
	Equal variances not assumed			2.553	56.515	.013	.39969	.15656	.08612	.71325

Women

Independent Samples Test

		Levene's Test for Equality of Variances		t-test for Equality of Means						
									95% Confidence Interval of the Difference	
		F	Sig.	t	df	Sig. (2-tailed)	Mean Difference	Std. Error Difference	Lower	Upper
Motivation Towards Learning Biology	Equal variances assumed	.321	.573	-3.667	59	.001	-.52471	.14309	-.81103	-.23839
	Equal variances not assumed			-3.687	58.996	.000	-.52471	.14232	-.80949	-.23992

Men

Table (3.4.3.3.2) The independent samples test for factor to determine the F values and significance.

Factor 4 Benefit and Utility of biology

Traditional Learning

Case Processing Summary

		N	%
Cases	Valid	28	100.0
	Excluded[a]	0	.0
	Total	28	100.0

Women

		N	%
Cases	Valid	29	100.0
	Excluded[a]	0	.0
	Total	29	100.0

Men

Table (3.4.4.1.1) Listwise deletion based on all variables in the procedure

Reliability Statistics

Cronbach's Alpha	Cronbach's Alpha Based on Standardized Items	N of Items
.814	.817	5

Women

Cronbach's Alpha	Cronbach's Alpha Based on Standardized Items	N of Items
.889	.890	5

Men

Table (3.4.4.1.2) The Cronbach's alpha for the factor is calculated based on the number of items.

Item Statistics

	Mean	Std. Deviation	N
I use the biology that I learn in school in my life.	3.68	.905	28
What I learn in my biology class helps me understand how things work in life.	4.14	.756	28
Learning biology makes me curious about things that I observe in my life.	4.04	.962	28
What we learn in biology class helps me to understand how biology affects my life.	4.14	.803	28
Learning biology helps me to make wiser decisions about my lifestyle and health.	4.04	.962	28

Women

	Mean	Std. Deviation	N
I use the biology that I learn in school in my life.	4.07	.961	29
What I learn in my biology class helps me understand how things work in life.	4.38	.820	29
Learning biology makes me curious about things that I observe in my life.	4.31	1.004	29
What we learn in biology class helps me to understand how biology affects my life.	4.38	.820	29

Learning biology helps me to make wiser decisions about my lifestyle and health.	4.24	1.023	29

Men

Table (3.4.4.1.3) The statistics for the mean and standard deviation for each item in the factor in the questionnaire.

Item-Total Statistics

	Scale Mean if Item Deleted	Scale Variance if Item Deleted	Corrected Item-Total Correlation	Squared Multiple Correlation	Cronbach's Alpha if Item Deleted
I use the biology that I learn in school in my life.	16.36	7.794	.502	.352	.809
What I learn in my biology class helps me understand how things work in life.	15.89	7.877	.636	.483	.772
Learning biology makes me curious about things that I observe in my life.	16.00	6.370	.794	.658	.714
What we learn in biology class helps me to understand how biology affects my life.	15.89	7.951	.563	.564	.790
Learning biology helps me to make wiser decisions about my lifestyle and health.	16.00	7.333	.555	.381	.795

Women

	Scale Mean if Item Deleted	Scale Variance if Item Deleted	Corrected Item-Total Correlation	Squared Multiple Correlation	Cronbach's Alpha if Item Deleted
I use the biology that I learn in school in my life.	17.31	9.579	.749	.673	.860
What I learn in my biology class helps me understand how things work in life.	17.00	10.500	.712	.617	.870
Learning biology makes me curious about things that I observe in my life.	17.07	8.924	.838	.708	.838
What we learn in biology class helps me to understand how biology affects my life.	17.00	10.786	.650	.619	.882
Learning biology helps me to make wiser decisions about my lifestyle and health.	17.14	9.409	.717	.664	.869

Men

Table (3.4.4.1.4) The total statistics for each item in the factor in the questionnaire.

Collaborative Learning

Case Processing Summary

		N	%
Cases	Valid	32	100.0
	Excluded[a]	0	.0
	Total	32	100.0

Women

Cases	Valid	32	100.0
	Excluded[a]	0	.0
	Total	32	100.0

Men

Table (3.4.4.2.1) Listwise deletion based on all variables in the procedure

Reliability Statistics

Cronbach's Alpha	Cronbach's Alpha Based on Standardized Items	N of Items
.812	.817	5

Women

Cronbach's Alpha	Cronbach's Alpha Based on Standardized Items	N of Items
.796	.822	5

Men

Table (3.4.4.2.2) The Cronbach's alpha for the factor is calculated based on the number of items.

Item Statistics

	Mean	Std. Deviation	N
I use the biology that I learn in school in my life.	4.19	.592	32
What I learn in my biology class helps me understand how things work in life.	3.66	1.066	32
Learning biology makes me curious about things that I observe in my life.	3.84	.987	32
What we learn in biology class helps me to understand how biology affects my life.	3.72	1.085	32
Learning biology helps me to make wiser decisions about my lifestyle and health.	4.03	.822	32

Women

	Mean	Std. Deviation	N
I use the biology that I learn in school in my life.	4.91	.530	32
What I learn in my biology class helps me understand how things work in life.	4.66	.937	32
Learning biology makes me curious about things that I observe in my life.	4.63	1.100	32
What we learn in biology class helps me to understand how biology affects my life.	4.84	.574	32

Learning biology helps me to make wiser decisions about my lifestyle and health.	4.66	1.096	32

Men

Table (3.4.4.2.3) The statistics for the mean and standard deviation for each item in the factor in the questionnaire.

Item-Total Statistics

	Scale Mean if Item Deleted	Scale Variance if Item Deleted	Corrected Item-Total Correlation	Squared Multiple Correlation	Cronbach's Alpha if Item Deleted
I use the biology that I learn in school in my life.	15.25	10.065	.506	.388	.808
What I learn in my biology class helps me understand how things work in life.	15.78	7.080	.723	.716	.735
Learning biology makes me curious about things that I observe in my life.	15.59	7.604	.687	.774	.748
What we learn in biology class helps me to understand how biology affects my life.	15.72	7.628	.587	.753	.785
Learning biology helps me to make wiser decisions about my lifestyle and health.	15.41	8.894	.560	.789	.788

Women

	Scale Mean if Item Deleted	Scale Variance if Item Deleted	Corrected Item-Total Correlation	Squared Multiple Correlation	Cronbach's Alpha if Item Deleted
I use the biology that I learn in school in my life.	18.78	8.757	.541	.517	.782
What I learn in my biology class helps me understand how things work in life.	19.03	6.741	.641	.815	.736
Learning biology makes me curious about things that I observe in my life.	19.06	5.415	.803	.689	.671
What we learn in biology class helps me to understand how biology affects my life.	18.84	8.201	.671	.722	.753
Learning biology helps me to make wiser decisions about my lifestyle and health.	19.03	6.999	.438	.552	.819

Men

Table (3.4.4.2.4) The total statistics for each item in the factor in the questionnaire.

TL vs CL

Group Statistics

	Method	N	Mean	Std. Deviation	Std. Error Mean
Benefit and Utility of biology	TL	28	3.9093	.60031	.11345
	CL	32	3.7956	.60003	.10607

Women

	Method	N	Mean	Std. Deviation	Std. Error Mean
Benefit and Utility of biology	TL	29	4.0517	.65019	.12074
	CL	32	4.7509	.64537	.11409

Men

Table (3.4.4.3.1) The group statistics for each item in the factors questionnaire with method.

Independent Samples Test

		Levene's Test for Equality of Variances		t-test for Equality of Means						
									95% Confidence Interval of the Difference	
		F	Sig.	t	df	Sig. (2-tailed)	Mean Difference	Std. Error Difference	Lower	Upper
Benefit and Utility of biology	Equal variances assumed	.019	.892	.732	58	.467	.11366	.15531	-.19722	.42454
	Equal variances not assumed			.732	56.940	.467	.11366	.15531	-.19735	.42467

Women

Independent Samples Test

		Levene's Test for Equality of Variances		t-test for Equality of Means						
									95% Confidence Interval of the Difference	
		F	Sig.	t	df	Sig. (2-tailed)	Mean Difference	Std. Error Difference	Lower	Upper
Benefit and Utility of biology	Equal variances assumed	1.270	.264	-4.211	59	.000	-.69921	.16605	-1.03148	-.36695
	Equal variances not assumed			-4.209	58.325	.000	-.69921	.16611	-1.03168	-.36674

Men

Table (3.4.4.3.2) The independent samples test for factor to determine the F values and significance.

Factor 5 Career Motivation

Traditional Learning

Case Processing Summary

		N	%
Cases	Valid	28	100.0
	Excluded[a]	0	.0
	Total	28	100.0

Women

		N	%
Cases	Valid	29	100.0
	Excluded[a]	0	.0
	Total	29	100.0

Men

Table (3.4.5.1.1) Listwise deletion based on all variables in the procedure

Reliability Statistics

Cronbach's Alpha	Cronbach's Alpha Based on Standardized Items	N of Items
.779	.813	5

Women

Cronbach's Alpha	Cronbach's Alpha Based on Standardized Items	N of Items
.728	.794	5

Men

Table (3.4.5.1.2) The Cronbach's alpha for the factor is calculated based on the number of items.

Item Statistics

	Mean	Std. Deviation	N
Learning biology will help me get a good job.	4.32	1.056	28
Knowing biology will give me a career advantage.	4.25	.928	28
Understanding biology will benefit me in my career.	4.36	.731	28
My career will involve science.	4.50	.509	28
I will use biology problem-solving skills in my career	4.25	.645	28

Women

	Mean	Std. Deviation	N
Learning biology will help me get a good job.	4.17	1.071	29
Knowing biology will give me a career advantage.	3.97	.778	29
Understanding biology will benefit me in my career.	4.03	.566	29
My career will involve science.	4.17	.384	29
I will use biology problem-solving skills in my career	4.21	.491	29

Men

Table (3.4.5.1.3) The statistics for the mean and standard deviation for each item in the factor in the questionnaire.

Item-Total Statistics

	Scale Mean if Item Deleted	Scale Variance if Item Deleted	Corrected Item-Total Correlation	Squared Multiple Correlation	Cronbach's Alpha if Item Deleted
Learning biology will help me get a good job.	17.36	5.127	.461	.296	.795
Knowing biology will give me a career advantage.	17.43	4.847	.671	.609	.694
Understanding biology will benefit me in my career.	17.32	5.560	.683	.675	.697
My career will involve science.	17.18	6.152	.806	.785	.699
I will use biology problem-solving skills in my career	17.43	6.847	.351	.205	.794

Women

	Scale Mean if Item Deleted	Scale Variance if Item Deleted	Corrected Item-Total Correlation	Squared Multiple Correlation	Cronbach's Alpha if Item Deleted
Learning biology will help me get a good job.	16.38	3.030	.462	.353	.754
Knowing biology will give me a career advantage.	16.59	3.394	.662	.541	.604
Understanding biology will benefit me in my career.	16.52	4.401	.496	.448	.684
My career will involve science.	16.38	4.601	.697	.616	.662
I will use biology problem-solving skills in my career	16.34	4.734	.432	.370	.708

Men

Table (3.4.5.1.4) The total statistics for each item in the factor in the questionnaire.

Collaborative Learning

Case Processing Summary

		N	%
Cases	Valid	32	100.0
	Excluded[a]	0	.0
	Total	32	100.0

Women

Cases	Valid	32	100.0
	Excluded[a]	0	.0
	Total	32	100.0

Men

Table (3.4.5.2.1) Listwise deletion based on all variables in the procedure

Reliability Statistics

Cronbach's Alpha	Cronbach's Alpha Based on Standardized Items	N of Items
.792	.796	5

Women

Cronbach's Alpha	Cronbach's Alpha Based on Standardized Items	N of Items
.803	.812	5

Men

Table (3.4.5.2.2) The Cronbach's alpha for the factor is calculated based on the number of items.

Item Statistics

	Mean	Std. Deviation	N
I use the biology that I learn in school in my life.	3.50	1.016	32
What I learn in my biology class helps me understand how things work in life.	3.44	1.014	32
Learning biology makes me curious about things that I observe in my life.	3.50	1.016	32
What we learn in biology class helps me to understand how biology affects my life.	3.81	.780	32
Learning biology helps me to make wiser decisions about my lifestyle and health.	3.44	.982	32

Women

	Mean	Std. Deviation	N
I use the biology that I learn in school in my life.	4.81	.738	32
What I learn in my biology class helps me understand how things work in life.	4.69	.644	32
Learning biology makes me curious about things that I observe in my life.	4.66	.827	32
What we learn in biology class helps me to understand how biology affects my life.	4.59	.875	32

Learning biology helps me to make wiser decisions about my lifestyle and health.	4.72	.813	32

Men

Table (3.4.5.2.3) The statistics for the mean and standard deviation for each item in the factor in the questionnaire.

Item-Total Statistics

	Scale Mean if Item Deleted	Scale Variance if Item Deleted	Corrected Item-Total Correlation	Squared Multiple Correlation	Cronbach's Alpha if Item Deleted
I use the biology that I learn in school in my life.	14.19	7.770	.695	.528	.710
What I learn in my biology class helps me understand how things work in life.	14.25	8.065	.633	.508	.732
Learning biology makes me curious about things that I observe in my life.	14.19	8.415	.558	.335	.758
What we learn in biology class helps me to understand how biology affects my life.	13.88	9.210	.616	.397	.746
Learning biology helps me to make wiser decisions about my lifestyle and health.	14.25	9.419	.391	.181	.809

Women

	Scale Mean if Item Deleted	Scale Variance if Item Deleted	Corrected Item-Total Correlation	Squared Multiple Correlation	Cronbach's Alpha if Item Deleted
I use the biology that I learn in school in my life.	18.66	5.846	.614	.671	.757
What I learn in my biology class helps me understand how things work in life.	18.78	5.789	.766	.897	.722
Learning biology makes me curious about things that I observe in my life.	18.81	5.964	.478	.721	.800
What we learn in biology class helps me to understand how biology affects my life.	18.88	5.081	.693	.775	.729

Learning biology helps me to make wiser decisions about my lifestyle and health.	18.75	6.129	.445	.671	.809

Men

Table (3.4.5.2.4) The total statistics for each item in the factor in the questionnaire.

TL vs CL

Group Statistics

	Method	N	Mean	Std. Deviation	Std. Error Mean
Career Motivation	TL	28	4.0725	.49090	.09277
	CL	32	3.5316	.70892	.12532

Women

	Method	N	Mean	Std. Deviation	Std. Error Mean
Career Motivation	TL	29	3.8938	.41949	.07790
	CL	32	4.8791	.64099	.11331

Men

Table (3.4.5.3.1) The group statistics for each item in the factors questionnaire with method.

Independent Samples Test

		Levene's Test for Equality of Variances		t-test for Equality of Means					95% Confidence Interval of the Difference	
		F	Sig.	t	df	Sig. (2-tailed)	Mean Difference	Std. Error Difference	Lower	Upper
Career Motivation	Equal variances assumed	.591	.445	3.388	58	.001	.54094	.15969	.22129	.86058
	Equal variances not assumed			3.469	55.239	.001	.54094	.15592	.22849	.85338

Women

Independent Samples Test

		Levene's Test for Equality of Variances		t-test for Equality of Means					95% Confidence Interval of the Difference	
		F	Sig.	t	df	Sig. (2-tailed)	Mean Difference	Std. Error Difference	Lower	Upper
Benefit and Utility of biology	Equal variances assumed	2.682	.107	-7.023	59	.000	-.98527	.14029	-1.26598	-.70456
	Equal variances not assumed			-7.165	53.897	.000	-.98527	.13751	-1.26096	-.70957

Men

Table (3.4.5.3.2) The independent samples test for factors determine the F values and significance.

Factor 6 Self-Efficacy in Biology Learning

Traditional Learning

Case Processing Summary

		N	%
Cases	Valid	28	100.0
	Excluded[a]	0	.0
	Total	28	100.0

Women

		N	%
Cases	Valid	29	100.0
	Excluded[a]	0	.0
	Total	29	100.0

Men

Table (3.4.6.1.1) Listwise deletion based on all variables in the procedure

Reliability Statistics

Cronbach's Alpha	Cronbach's Alpha Based on Standardized Items	N of Items
.804	.805	8

Women

Cronbach's Alpha	Cronbach's Alpha Based on Standardized Items	N of Items
.835	.837	8

Men

Table (3.4.6.1.2) The Cronbach's alpha for the factor is calculated based on the number of items.

Item Statistics

	Mean	Std. Deviation	N
If I study hard I can do well in biology	3.7857	.78680	28
I believe biology is too easy for me to learn	3.5714	.87891	28
The idea of taking biology makes me excited.	3.4643	.99934	28
I am confident I will do well on biology tests.	4.0714	1.01575	28
I am confident I will do well on biology labs and projects.	3.5357	1.23175	28
I believe I can master biology knowledge and skills.	3.9286	1.21499	28
I believe I can earn a grade of "A" in biology.	4.0714	1.01575	28
I am sure I can understand biology.	4.1429	1.07890	28

Women

	Mean	Std. Deviation	N
If I study hard I can do well in biology	4.0345	.94426	29
I believe biology is too easy for me to learn	3.8276	1.07135	29
The idea of taking biology makes me excited.	3.7241	1.19213	29
I am confident I will do well on biology tests.	4.0690	.99753	29
I am confident I will do well on biology labs and projects.	3.6207	1.26530	29
I believe I can master biology knowledge and skills.	4.1034	1.20549	29

I believe I can earn a grade of "A" in biology.	4.2069	.94034	29
I am sure I can understand biology.	4.1724	1.07135	29

Men

Table (3.4.6.1.3) The statistics for the mean and standard deviation for each item in the factor in the questionnaire.

Item-Total Statistics

	Scale Mean if Item Deleted	Scale Variance if Item Deleted	Corrected Item-Total Correlation	Squared Multiple Correlation	Cronbach's Alpha if Item Deleted
If I study hard I can do well in biology	26.7857	25.434	.380	.308	.800
I believe biology is too easy for me to learn	27.0000	22.815	.653	.661	.766
The idea of taking biology makes me excited.	27.1071	24.544	.356	.557	.805
I am confident I will do well on biology tests.	26.5000	23.296	.483	.425	.787
I am confident I will do well on biology labs and projects.	27.0357	23.073	.378	.337	.808
I believe I can master biology knowledge and skills.	26.6429	19.646	.738	.737	.743
I believe I can earn a grade of "A" in biology.	26.5000	24.185	.386	.236	.801
I am sure I can understand biology.	26.4286	20.032	.815	.789	.733

Women

	Scale Mean if Item Deleted	Scale Variance if Item Deleted	Corrected Item-Total Correlation	Squared Multiple Correlation	Cronbach's Alpha if Item Deleted
If I study hard I can do well in biology	27.7241	29.564	.489	.433	.825
I believe biology is too easy for me to learn	27.9310	26.638	.695	.741	.799
The idea of taking biology makes me excited.	28.0345	28.106	.471	.694	.830
I am confident I will do well on biology tests.	27.6897	29.007	.509	.435	.823
I am confident I will do well on biology labs and projects.	28.1379	28.337	.411	.403	.840
I believe I can master biology knowledge and skills.	27.6552	24.591	.789	.746	.783
I believe I can earn a grade of "A" in biology.	27.5517	30.113	.434	.410	.831
I am sure I can understand biology.	27.5862	26.037	.758	.736	.791

Men

Table (3.4.6.1.4) The total statistics for each item in the factor in the questionnaire.

Collaborative Learning

Case Processing Summary

		N	%
Cases	Valid	32	100.0
	Excluded[a]	0	.0
	Total	32	100.0

Women

Cases	Valid	32	100.0
	Excluded[a]	0	.0
	Total	32	100.0

Men

Table (3.4.6.2.1) Listwise deletion based on all variable in the procedure

Reliability Statistics

Cronbach's Alpha	Cronbach's Alpha Based on Standardized Items	N of Items
.826	.823	8

Women

Cronbach's Alpha	Cronbach's Alpha Based on Standardized Items	N of Items
.710	.744	8

Men

Table (3.4.6.2.2) The Cronbach's alpha for the factor is calculated based on the number of items.

Item Statistics

	Mean	Std. Deviation	N
If I study hard I can do well in biology	3.7813	.83219	32
I believe biology is too easy for me to learn	3.4375	1.01401	32
The idea of taking biology makes me excited.	3.0625	1.13415	32
I am confident I will do well on biology tests.	2.9375	1.13415	32
I am confident I will do well on biology labs and projects.	3.0313	1.06208	32
I believe I can master biology knowledge and skills.	3.2188	1.12836	32

I believe I can earn a grade of “A” in biology.	3.0938	1.05828	32
I am sure I can understand biology.	3.0000	1.10716	32

Women

	Mean	Std. Deviation	N
If I study hard I can do well in biology	4.5313	1.10671	32
I believe biology is too easy for me to learn	4.1250	1.43122	32
The idea of taking biology makes me excited.	3.5625	1.58496	32
I am confident I will do well on biology tests.	4.8750	.70711	32
I am confident I will do well on biology labs and projects.	4.8750	.49187	32
I believe I can master biology knowledge and skills.	4.8125	.78030	32
I believe I can earn a grade of “A” in biology.	4.6563	.82733	32
I am sure I can understand biology.	4.7500	.80322	32

Men

Table (3.4.6.2.3) The statistics for the mean and standard deviation for each item in the factor in the questionnaire

Item-Total Statistics

	Scale Mean if Item Deleted	Scale Variance if Item Deleted	Corrected Item-Total Correlation	Squared Multiple Correlation	Cronbach's Alpha if Item Deleted
If I study hard I can do well in biology	21.7813	29.789	.231	.316	.840
I believe biology is too easy for me to learn	22.1250	23.661	.800	.779	.772

The idea of taking biology makes me excited.	22.5000	24.839	.571	.597	.802
I am confident I will do well on biology tests.	22.6250	25.145	.540	.476	.807
I am confident I will do well on biology labs and projects.	22.5313	24.257	.687	.691	.786
I believe I can master biology knowledge and skills.	22.3438	26.491	.414	.492	.825
I believe I can earn a grade of "A" in biology.	22.4688	24.967	.614	.500	.797
I am sure I can understand biology.	22.5625	25.351	.538	.559	.807

Women

	Scale Mean if Item Deleted	Scale Variance if Item Deleted	Corrected Item-Total Correlation	Squared Multiple Correlation	Cronbach's Alpha if Item Deleted
If I study hard I can do well in biology	31.6563	15.459	.651	.546	.620
I believe biology is too easy for me to learn	32.0625	13.802	.611	.534	.624
The idea of taking biology makes me excited.	32.6250	14.952	.399	.517	.702
I am confident I will do well on biology tests.	31.3125	19.190	.430	.549	.682
I am confident I will do well on biology labs and projects.	31.3125	19.577	.582	.700	.677
I believe I can master biology knowledge and skills.	31.3750	19.919	.262	.355	.706
I believe I can earn a grade of "A" in biology.	31.5313	18.580	.433	.482	.678
I am sure I can understand biology.	31.4375	21.093	.083	.280	.734

Men

Table (3.4.6.2.4) The total statistics for each item in the factor in the questionnaire.

TL vs CL

Group Statistics

	Method	N	Mean	Std. Deviation	Std. Error Mean
Career Motivation	TL	28	3.7036	.65325	.12345
	CL	32	3.3913	.82426	.14571

Women

	Method	N	Mean	Std. Deviation	Std. Error Mean
Career Motivation	TL	29	3.8776	.74734	.13878
	CL	32	4.5500	.59407	.10502

Men

Table (3.4.6.3.1) The group statistics for each item in the factors questionnaire with method.

Independent Samples Test

		Levene's Test for Equality of Variances		t-test for Equality of Means						
									95% Confidence Interval of the Difference	
		F	Sig.	t	df	Sig. (2-tailed)	Mean Difference	Std. Error Difference	Lower	Upper
Career Motivation	Equal variances assumed	5.843	.019	1.610	58	.113	.31232	.19396	-.07593	.70057
	Equal variances not assumed			1.635	57.476	.107	.31232	.19098	-.07003	.69468

Women

Independent Samples Test

		Levene's Test for Equality of Variances		t-test for Equality of Means						
									95% Confidence Interval of the Difference	
		F	Sig.	t	df	Sig. (2-tailed)	Mean Difference	Std. Error Difference	Lower	Upper
Self-Efficacy in biology Learning	Equal variances assumed	.278	.600	-3.908	59	.000	-.67241	.17208	-1.01675	-.32808
	Equal variances not assumed			-3.864	53.426	.000	-.67241	.17403	-1.02142	-.32341

Men

Table (3.4.6.3.2) The independent samples test for factor in the questionnaire to determine the F values and significance

Factor 7 Self-Determination

Traditional Learning

Case Processing Summary

		N	%
Cases	Valid	28	100.0
	Excluded[a]	0	.0
	Total	28	100.0

Women

		N	%
Cases	Valid	29	100.0
	Excluded[a]	0	.0
	Total	29	100.0

Men

Table (3.4.7.1.1) Listwise deletion based on all variables in the procedure

Reliability Statistics

Cronbach's Alpha	Cronbach's Alpha Based on Standardized Items	N of Items
.830	.828	5

Women

Cronbach's Alpha	Cronbach's Alpha Based on Standardized Items	N of Items
.732	.728	5

Men

Table (3.4.7.1.2) The Cronbach’s alpha for the factor is calculated based on the number of items.

Item Statistics

	Mean	Std. Deviation	N
I put enough effort into learning biology.	4.00	1.155	28
I use strategies to learn biology well.	3.93	1.184	28
I spend a lot of time learning biology.	4.25	1.041	28
I prepare well for biology tests and labs.	4.36	1.062	28
I study hard to learn biology.	4.46	.881	28

Women

	Mean	Std. Deviation	N
I put enough effort into learning biology.	3.69	.967	29
I use strategies to learn biology well.	3.66	.974	29
I spend a lot of time learning biology.	3.79	.774	29
I prepare well for biology tests and labs.	3.97	.906	29
I study hard to learn biology.	4.10	.724	29

Men

Table (3.4.7.1.3) The statistics for the mean and standard deviation for each item in the factor in the questionnaire.

Item-Total Statistics

	Scale Mean if Item Deleted	Scale Variance if Item Deleted	Corrected Item-Total Correlation	Squared Multiple Correlation	Cronbach's Alpha if Item Deleted
I put enough effort into learning biology.	17.00	10.667	.668	.665	.785
I use strategies to learn biology well.	17.07	10.069	.741	.732	.761
I spend a lot of time learning biology.	16.75	11.231	.677	.526	.783
I prepare well for biology tests and labs.	16.64	11.794	.565	.623	.814
I study hard to learn biology.	16.54	13.073	.500	.578	.829

Women

	Scale Mean if Item Deleted	Scale Variance if Item Deleted	Corrected Item-Total Correlation	Squared Multiple Correlation	Cronbach's Alpha if Item Deleted
I put enough effort into learning biology.	15.52	5.687	.567	.544	.655
I use strategies to learn biology well.	15.55	5.328	.660	.642	.613
I spend a lot of time learning biology.	15.41	6.823	.450	.394	.702
I prepare well for biology tests and labs.	15.24	6.547	.404	.602	.721
I study hard to learn biology.	15.10	7.167	.399	.582	.719

Men

Table (3.4.7.1.4) The total statistics for each item in the factor in the questionnaire.

Collaborative Learning

Case Processing Summary

		N	%
Cases	Valid	32	100.0
	Excluded[a]	0	.0
	Total	32	100.0

Women

Cases	Valid	32	100.0
	Excluded[a]	0	.0
	Total	32	100.0

Men

Table (3.4.7.2.1) Listwise deletion based on all variable in the procedure

Reliability Statistics

Cronbach's Alpha	Cronbach's Alpha Based on Standardized Items	N of Items
.713	.716	5

Women

Cronbach's Alpha	Cronbach's Alpha Based on Standardized Items	N of Items
.728	.778	5

Men

Table (3.4.7.2.2) The Cronbach's alpha for the factor is calculated based on the number of items.

Item Statistics

	Mean	Std. Deviation	N
I use the biology that I learn in school in my life.	3.03	1.177	32
What I learn in my biology class helps me understand how things work in life.	3.13	1.238	32
Learning biology makes me curious about things that I observe in my life.	3.03	1.231	32
What we learn in biology class helps me to understand how biology affects my life.	2.97	1.231	32
Learning biology helps me to make wiser decisions about my lifestyle and health.	2.59	1.434	32

Women

	Mean	Std. Deviation	N
I use the biology that I learn in school in my life.	4.53	1.107	32
What I learn in my biology class helps me understand how things work in life.	4.13	1.431	32
Learning biology makes me curious about things that I observe in my life.	3.56	1.585	32
What we learn in biology class helps me to understand how biology affects my life.	4.88	.707	32

Learning biology helps me to make wiser decisions about my lifestyle and health.	4.88	.492	32

Men

Table (3.4.7.2.3) The statistics for the mean and standard deviation for each item in the factor in the questionnaire.

Item-Total Statistics

	Scale Mean if Item Deleted	Scale Variance if Item Deleted	Corrected Item-Total Correlation	Squared Multiple Correlation	Cronbach's Alpha if Item Deleted
I use the biology that I learn in school in my life.	11.72	12.660	.549	.488	.636
What I learn in my biology class helps me understand how things work in life.	11.63	11.274	.702	.567	.568
Learning biology makes me curious about things that I observe in my life.	11.72	14.725	.255	.108	.746
What we learn in biology class helps me to understand how biology affects my life.	11.78	13.402	.414	.271	.688
Learning biology helps me to make wiser decisions about my lifestyle and health.	12.16	11.878	.477	.333	.666

Women

	Scale Mean if Item Deleted	Scale Variance if Item Deleted	Corrected Item-Total Correlation	Squared Multiple Correlation	Cronbach's Alpha if Item Deleted
I use the biology that I learn in school in my life.	17.44	10.319	.577	.445	.648
What I learn in my biology class helps me understand how things work in life.	17.84	8.007	.690	.529	.588
Learning biology makes me curious about things that I observe in my life.	18.41	8.378	.518	.476	.694
What we learn in biology class helps me to understand how biology affects my life.	17.09	13.314	.355	.490	.730
Learning biology helps me to make wiser decisions about my lifestyle and health.	17.09	13.443	.543	.610	.710

Men

Table (3.4.6.2.4) The total statistics for each item in the factor in the questionnaire.

TL vs CL

Group Statistics

	Method	N	Mean	Std. Deviation	Std. Error Mean
Self-Determination	TL	28	4.2104	.80573	.15227
	CL	32	3.0850	.89910	.15894

Women

	Method	N	Mean	Std. Deviation	Std. Error Mean
Self-Determination	TL	29	3.8517	.58718	.10904
	CL	32	4.3622	.75307	.13313

Men

Table (3.4.7.3.1) The group statistics for each item in the factor's questionnaire with method.

Independent Samples Test

		Levene's Test for Equality of Variances		t-test for Equality of Means						
									95% Confidence Interval of the Difference	
		F	Sig.	t	df	Sig. (2-tailed)	Mean Difference	Std. Error Difference	Lower	Upper
Grade Motivation	Equal variances assumed	.295	.589	5.075	58	.000	1.12536	.22174	.68149	1.56923
	Equal variances not assumed			5.113	57.960	.000	1.12536	.22011	.68476	1.56596

Women

Independent Samples Test

		Levene's Test for Equality of Variances		t-test for Equality of Means						
									95% Confidence Interval of the Difference	
		F	Sig.	t	df	Sig. (2-tailed)	Mean Difference	Std. Error Difference	Lower	Upper
Self-Determination	Equal variances assumed	3.774	.057	-2.930	59	.005	-.51046	.17419	-.85902	-.16191
	Equal variances not assumed			-2.966	57.763	.004	-.51046	.17208	-.85495	-.16598

Men

Table (3.4.7.3.2) The independent samples test for factors to determine the F values and significance.

Factor 8 Grade Motivation

Traditional Learning

Case Processing Summary

		N	%
Cases	Valid	28	100.0
	Excluded[a]	0	.0
	Total	28	100.0

Women

		N	%
Cases	Valid	29	100.0
	Excluded[a]	0	.0
	Total	29	100.0

Men

Table (3.4.8.1.1) Listwise deletion based on all variables in the procedure

Reliability Statistics

Cronbach's Alpha	Cronbach's Alpha Based on Standardized Items	N of Items
.749	.764	5

Women

Cronbach's Alpha	Cronbach's Alpha Based on Standardized Items	N of Items
.749	.764	5

Men

Table (3.4.8.1.2) The Cronbach's alpha for the factor is calculated based on the number of items.

Item Statistics

	Mean	Std. Deviation	N
I like to do better than other students on biology tests.	4.57	.742	28
Getting a good biology grade is important to me.	4.82	.390	28
It is important that I get an "A" in biology.	4.82	.390	28
Scoring high on biology tests and labs matters to me.	4.50	1.000	28

Women

	Mean	Std. Deviation	N
I like to do better than other students on biology tests.	4.00	1.165	29
Getting a good biology grade is important to me.	3.76	.988	29
It is important that I get an "A" in biology.	4.03	.566	29
I think about the grade I will get in biology.	3.93	.753	29
Scoring high on biology tests and labs matters to me.	4.14	.639	29

Men

Table (3.4.8.1.3) The statistics for the mean and standard deviation for each item in the factor in the questionnaire.

Item-Total Statistics

	Scale Mean if Item Deleted	Scale Variance if Item Deleted	Corrected Item-Total Correlation	Squared Multiple Correlation	Cronbach's Alpha if Item Deleted
I like to do better than other students on biology tests.	14.14	3.016	.854	.	.851
Getting a good biology grade is important to me.	13.89	4.173	.905	.	.888
It is important that I get an "A" in biology.	13.89	4.173	.905	.	.888
Scoring high on biology tests and labs matters to me.	14.21	2.026	.963	.	.867

Women

	Scale Mean if Item Deleted	Scale Variance if Item Deleted	Corrected Item-Total Correlation	Squared Multiple Correlation	Cronbach's Alpha if Item Deleted
I like to do better than other students on biology tests.	15.86	4.909	.540	.323	.717
Getting a good biology grade is important to me.	16.10	4.953	.710	.532	.619
It is important that I get an "A" in biology.	15.83	7.291	.472	.343	.728
I think about the grade I will get in biology.	15.93	6.924	.394	.287	.744
Scoring high on biology tests and labs matters to me.	15.72	6.707	.585	.348	.693

Men

Table (3.4.8.1.4) The total statistics for each item in the factor in the questionnaire.

Collaborative Learning

Case Processing Summary

		N	%
Cases	Valid	31	100.0
	Excluded[a]	0	.0
	Total	31	100.0

Women

Cases	Valid	32	100.0
	Excluded[a]	0	.0
	Total	32	100.0

Men

Table (3.4.8.2.1) Listwise deletion based on all variable in the procedure

Reliability Statistics

Cronbach's Alpha	Cronbach's Alpha Based on Standardized Items	N of Items
.747	.812	5

Women

Cronbach's Alpha	Cronbach's Alpha Based on Standardized Items	N of Items
.792	.795	5

Men

Table (3.4.8.2.2) The Cronbach's alpha for the factor is calculated based on the number of items.

Item Statistics

	Mean	Std. Deviation	N
I use the biology that I learn in school in my life.	4.28	.634	32
What I learn in my biology class helps me understand how things work in life.	3.69	1.091	32
Learning biology makes me curious about things that I observe in my life.	3.91	1.027	32
What we learn in biology class helps me to understand how biology affects my life.	3.75	1.107	32
Learning biology helps me to make wiser decisions about my lifestyle and health.	4.09	.856	32

Women

	Mean	Std. Deviation	N
I use the biology that I learn in school in my life.	4.75	.568	32
What I learn in my biology class helps me understand how things work in life.	4.88	.554	32

Learning biology makes me curious about things that I observe in my life.	4.91	.530	32
What we learn in biology class helps me to understand how biology affects my life.	4.91	.390	32
Learning biology helps me to make wiser decisions about my lifestyle and health.	4.88	.421	32

Men

Table (3.4.8.2.3) The statistics for the mean and standard deviation for each item in the factor in the questionnaire.

	Scale Mean if Item Deleted	Scale Variance if Item Deleted	Corrected Item-Total Correlation	Squared Multiple Correlation	Cronbach's Alpha if Item Deleted
I use the biology that I learn in school in my life.	15.44	10.770	.450	.345	.813
What I learn in my biology class helps me understand how things work in life.	16.03	7.580	.712	.719	.732
Learning biology makes me curious about things that I observe in my life.	15.81	7.899	.709	.788	.734
What we learn in biology class helps me to understand how biology affects my life.	15.97	8.225	.566	.732	.785
Learning biology helps me to make wiser decisions about my lifestyle and health.	15.63	9.274	.583	.797	.777

Women

	Scale Mean if Item Deleted	Scale Variance if Item Deleted	Corrected Item-Total Correlation	Squared Multiple Correlation	Cronbach's Alpha if Item Deleted
I use the biology that I learn in school in my life.	19.56	2.190	.518	.827	.775
What I learn in my biology class helps me understand how things work in life.	19.44	1.996	.691	.932	.710
Learning biology makes me curious about things that I observe in my life.	19.41	2.055	.688	.937	.711
What we learn in biology class helps me to understand how biology affects my life.	19.41	2.314	.773	.828	.706
Learning biology helps me to make wiser decisions about my lifestyle and health.	19.44	2.835	.262	.819	.834

Men

Table (3.4.8.2.4) The total statistics for each item in the factor in the questionnaire.

TL vs CL

Group Statistics

	Method	N	Mean	Std. Deviation	Std. Error Mean
Self-Determination	TL	28	4.7311	.49640	.09381
	CL	32	3.8519	.63235	.11178

Women

	Method	N	Mean	Std. Deviation	Std. Error Mean
Self-Determination	TL	29	3.7710	.53344	.09906
	CL	32	4.8456	.63467	.11219

Men

Table (3.4.8.3.1) The group statistics for each item in the factor's questionnaire with method.

Independent Samples Test

		Levene's Test for Equality of Variances		t-test for Equality of Means					95% Confidence Interval of the Difference	
		F	Sig.	t	df	Sig. (2-tailed)	Mean Difference	Std. Error Difference	Lower	Upper
Grade Motivation	Equal variances assumed	1.661	.203	5.928	58	.000	.87920	.14830	.58234	1.17605
	Equal variances not assumed			6.025	57.370	.000	.87920	.14593	.58701	1.17138

Women

Independent Samples Test

		Levene's Test for Equality of Variances		t-test for Equality of Means					95% Confidence Interval of the Difference	
		F	Sig.	t	df	Sig. (2-tailed)	Mean Difference	Std. Error Difference	Lower	Upper
Grade Motivation	Equal variances assumed	.280	.599	-7.118	59	.000	-1.07459	.15096	-1.37666	-.77252
	Equal variances not assumed			-7.180	58.686	.000	-1.07459	.14967	-1.37411	-.77507

Men

Table (3.4.8.3.2) The independent samples test for the factor to determine the F values and significance.

Factor 9 Assessment anxiety

Traditional Learning

Case Processing Summary

		N	%
Cases	Valid	28	100.0
	Excluded[a]	0	.0
	Total	28	100.0

Women

		N	%
Cases	Valid	30	100.0
	Excluded[a]	0	.0
	Total	30	100.0

Men

Table (3.4.9.1.1) Listwise deletion based on all variables in the procedure

Reliability Statistics

Cronbach's Alpha	Cronbach's Alpha Based on Standardized Items	N of Items
.749	.753	5

Women

Cronbach's Alpha	Cronbach's Alpha Based on Standardized Items	N of Items
.738	.734	5

Men

Table (3.4.9.1.2) The Cronbach's alpha for the factor is calculated based on the number of items.

Item Statistics

	Mean	Std. Deviation	N
I am nervous about how I will do on the biology tests.	3.57	.879	28
I become anxious when it is time to take a biology test.	4.14	.803	28
I worry about failing the biology tests.	3.93	.979	28
I am concerned that the other students are better in biology.	4.32	.772	28
I hate taking the biology tests.	4.07	.979	28

Women

	Mean	Std. Deviation	N
I am nervous about how I will do on the biology tests.	3.34	1.111	29
I become anxious when it is time to take a biology test.	3.86	1.187	29
I worry about failing the biology tests.	3.72	1.222	29
I am concerned that the other students are better in biology.	3.97	1.117	29
I hate taking the biology tests.	3.97	1.052	29

Men

Table (3.4.9.1.3) The statistics for the mean and standard deviation for each item in the factor in the questionnaire.

Item-Total Statistics

	Scale Mean if Item Deleted	Scale Variance if Item Deleted	Corrected Item-Total Correlation	Squared Multiple Correlation	Cronbach's Alpha if Item Deleted
I am nervous about how I will do on the biology tests.	16.46	6.554	.553	.389	.691
I become anxious when it is time to take a biology test.	15.89	6.766	.575	.339	.686
I worry about failing the biology tests.	16.11	5.803	.648	.510	.650
I am concerned that the other students are better in biology.	15.71	7.323	.453	.368	.726
I hate taking the biology tests.	15.96	6.925	.375	.173	.761

Women

	Scale Mean if Item Deleted	Scale Variance if Item Deleted	Corrected Item-Total Correlation	Squared Multiple Correlation	Cronbach's Alpha if Item Deleted
I am nervous about how I will do on the biology tests.	15.52	10.401	.586	.491	.660
I become anxious when it is time to take a biology test.	15.00	9.857	.613	.439	.646
I worry about failing the biology tests.	15.14	9.909	.577	.405	.661
I am concerned that the other students are better in biology.	14.90	11.382	.425	.243	.719
I hate taking the biology tests.	14.90	12.453	.307	.110	.757

Men

Table (3.4.9.1.4) The total statistics for each item in the factor in the questionnaire.

Collaborative Learning

Case Processing Summary

		N	%
Cases	Valid	32	100.0
	Excluded[a]	0	.0
	Total	32	100.0

Women

Cases	Valid	32	100.0
	Excluded[a]	0	.0
	Total	32	100.0

Men

Table (3.4.9.2.1) Listwise deletion based on all variable in the procedure

Reliability Statistics

Cronbach's Alpha	Cronbach's Alpha Based on Standardized Items	N of Items
.717	.716	5

Women

Cronbach's Alpha	Cronbach's Alpha Based on Standardized Items	N of Items
.743	.746	5

Men

Table (3.4.9.2.2) The Cronbach's alpha for the factor is calculated based on the number of items.

Item Statistics

	Mean	Std. Deviation	N
I am nervous about how I will do on the biology tests.	3.09	1.174	32
I become anxious when it is time to take a biology test.	2.94	1.190	32
I worry about failing the biology tests.	2.91	1.118	32
I am concerned that the other students are better in biology.	3.25	1.047	32
I hate taking the biology tests.	2.88	1.185	32

Women

	Mean	Std. Deviation	N
I am nervous about how I will do on the biology tests.	4.63	1.008	32
I become anxious when it is time to take a biology test.	4.66	.653	32
I worry about failing the biology tests.	4.53	.950	32
I am concerned that the other students are better in biology.	4.53	.915	32
I hate taking the biology tests.	4.75	.803	32

Men

Table (3.4.9.2.3) The statistics for the mean and standard deviation for each item in the factor in the questionnaire.

Item-Total Statistics

	Scale Mean if Item Deleted	Scale Variance if Item Deleted	Corrected Item-Total Correlation	Squared Multiple Correlation	Cronbach's Alpha if Item Deleted
I am nervous about how I will do on the biology tests.	11.97	10.160	.509	.306	.656
I become anxious when it is time to take a biology test.	12.13	9.790	.557	.381	.635
I worry about failing the biology tests.	12.16	10.652	.473	.295	.671
I am concerned that the other students are better in biology.	11.81	11.512	.386	.159	.703
I hate taking the biology tests.	12.19	10.480	.452	.213	.679

Women

	Scale Mean if Item Deleted	Scale Variance if Item Deleted	Corrected Item-Total Correlation	Squared Multiple Correlation	Cronbach's Alpha if Item Deleted
I am nervous about how I will do on the biology tests.	18.47	5.612	.589	.376	.665
I become anxious when it is time to take a biology test.	18.44	7.028	.574	.442	.688
I worry about failing the biology tests.	18.56	6.254	.481	.463	.710
I am concerned that the other students are better in biology.	18.56	5.544	.710	.592	.614
I hate taking the biology tests.	18.34	7.717	.242	.408	.783

Men

Table (3.4.9.2.4) The total statistics for each item in the factor in the questionnaire.

TL vs CL

Group Statistics

	Method	N	Mean	Std. Deviation	Std. Error Mean
Self-Determination	TL	28	3.9064	.56085	.10599
	CL	32	3.0119	.78395	.13858

Women

	Method	N	Mean	Std. Deviation	Std. Error Mean
Self-Determination	TL	29	3.6566	.70851	.13157
	CL	32	4.7891	.67698	.11967

Men

Table (3.4.9.3.1) The group statistics for each item in the factor's questionnaire with method.

Independent Samples Test

		Levene's Test for Equality of Variances		t-test for Equality of Means						
									95% Confidence Interval of the Difference	
		F	Sig.	t	df	Sig. (2-tailed)	Mean Difference	Std. Error Difference	Lower	Upper
Aassessment anxiety	Equal variances assumed	8.714	.005	5.016	58	.000	.89455	.17833	.53759	1.25152
	Equal variances not assumed			5.127	55.909	.000	.89455	.17447	.54504	1.24407

Women

Independent Samples Test

		Levene's Test for Equality of Variances		t-test for Equality of Means						
									95% Confidence Interval of the Difference	
		F	Sig.	t	df	Sig. (2-tailed)	Mean Difference	Std. Error Difference	Lower	Upper
Aassessment anxiety	Equal variances assumed	.460	.500	-6.382	59	.000	-1.13251	.17745	-1.48759	-.77744
	Equal variances not assumed			-6.368	57.777	.000	-1.13251	.17785	-1.48855	-.77647

Men

Table (3.3.9.3.2) The independent samples test for factor in questionnaire to determine the F values and significance.

All factors comparison

Group Statistics

	Method	N	Mean	Std. Deviation	Std. Error Mean
Attitude_9 Factors	TL	261	3.9946	.64565	.03996
	CL	289	4.6639	.63349	.03726

Women

	Method	N	Mean	Std. Deviation	Std. Error Mean
Attitude_9 Factors	TL	29	3.6566	.70851	.13157
	CL	32	4.7891	.67698	.11967

Men

Table (3.4.10.3.1) The group statistics for each item in the factor's questionnaire with method

Independent Samples Test

		Levene's Test for Equality of Variances		t-test for Equality of Means						
									95% Confidence Interval of the Difference	
		F	Sig.	t	df	Sig. (2-tailed)	Mean Difference	Std. Error Difference	Lower	Upper
Attitude_9 Factors	Equal variances assumed	.092	.762	-12.262	548	.000	-.66939	.05459	-.77662	-.56216
	Equal variances not assumed			-12.250	540.078	.000	-.66939	.05464	-.77672	-.56205

Women

Independent Samples Test

		Levene's Test for Equality of Variances		t-test for Equality of Means						
									95% Confidence Interval of the Difference	
		F	Sig.	t	df	Sig. (2-tailed)	Mean Difference	Std. Error Difference	Lower	Upper
Aassessment anxiety	Equal variances assumed	.460	.500	-6.382	59	.000	-1.13251	.17745	-1.48759	-.77744
	Equal variances not assumed			-6.368	57.777	.000	-1.13251	.17785	-1.48855	-.77647

Men

Table (3.4.10.3.2) The independent samples test for all factor in questionnaire to determine the F values and significance.

Attitude Women TL (All factors) VS Men TL (All factors)

Group Statistics

	Gender	N	Mean	Std. Deviation	Std. Error Mean
Attitude_9 Factors	Women	252	4.1311	.65403	.04120
	Men	261	3.9946	.64565	.03996

Table (3.4.10.3.3) The group statistics to compare women and men in all factors in TL

Independent Samples Test

		Levene's Test for Equality of Variances		t-test for Equality of Means						
									95% Confidence Interval of the Difference	
		F	Sig.	t	df	Sig. (2-tailed)	Mean Difference	Std. Error Difference	Lower	Upper
Attitude_9 Factors	Equal variances assumed	.310	.578	2.379	511	.018	.13651	.05739	.02377	.24925
	Equal variances not assumed			2.378	509.823	.018	.13651	.05740	.02374	.24928

Table (3.4.10.3.4) The independent samples test for all factor in questionnaire to determine the F values and significance.

Attitude Women CL (All factors) VS Men CL (All factors)

Group Statistics

	Gender	N	Mean	Std. Deviation	Std. Error Mean
Attitude_9 Factors	Women	289	3.6174	.80113	.04713
	Men	289	4.6639	.63349	.03726

Table (3.4.10.3.5) The group statistics to compare women and men in all factors in CL

Independent Samples Test

		Levene's Test for Equality of Variances		t-test for Equality of Means						
									95% Confidence Interval of the Difference	
		F	Sig.	t	df	Sig. (2-tailed)	Mean Difference	Std. Error Difference	Lower	Upper
Attitude_9 Factors	Equal variances assumed	25.084	.000	-17.420	576	.000	-1.04657	.06008	-1.16457	-.92858
	Equal variances not assumed			-17.420	546.926	.000	-1.04657	.06008	-1.16459	-.92856

Table (3.4.10.3.6) The independent samples test for all factor in questionnaire to determine the F values and significance.

Questionnaire

1-Feeling toward biology questionnaires (Factor # 1)

Russell and Hollander (1975):

James Russell, Steven Hollander
Each of the statements below expresses a feeling toward biology.

Please rate each statement on the extent to which you agree.

For each, you may (A) strongly agree, (B) agree, (C) be undecided, (D) disagree, or (E) strongly disagree. After you have made your choice, blacken in the appropriate response in the columns on the IBM card corresponding to each item.

(Factor # 1) Feeling toward biology
1. Biology is very interesting to me.
2. I don't like biology, and it scares me to have to take it.
3. I am always under a terrible strain in a biology class.
4. Biology is fascinating and fun.
5. Biology makes me feel secure, and at the same time it is stimulating. 6. Biology makes me feel uncomfortable, restless, irritable, and impatient.
7. In general, I have a good feeling toward biology.
8. When I hear the word biology, I have a feeling of dislike.
9. I approach biology with a feeling of hesitation.
10. I really like biology.
11. I have always enjoyed studying biology in school.
12. It makes me nervous to even think about doing a biology experiment.
13. I feel at ease in biology and like it very much.
14. I feel a definite positive reaction to biology; it's enjoyable.

2) *Students' Attitudes Towards science* (SATS) modified to Biology questionnaires course:

They included: Factor # 2 *General interest*, Factor # 3 *Motivation Towards Learning Biology, factor # 4 Benefit and Utility of biology, factor # 5 Career Motivation, factor #6 Self-Efficacy in biology Learning factor #7 Self-Determination, factor # 8 Grade Motivation, factor # 9 Aassessment anxiety*

Students respond to each items on a five-point Likert-type scale of temporal frequency ranging from 1 (never) to 5 (always). The anxiety about science assessment items are reverse scored when added to the total, so a higher score on this component means less anxiety.

1 = strongly disagree, 2 = disagree, 3 = no opinion, 4= agree, and 4 = strongly agree.

Factor # 2 *General interest:*
1. I like watching *biology* related TV.
2. *Biology* is my favorite subject in school.
3. I like reading about famous physicists like Albert Einstein and Isaac Newton.
4. I find what we learn in my *biology* class interesting.
5. I would enjoy working in a *biology* lab.

Factor # 3 *Motivation Towards Learning Biology*
6. I will ask my teacher for an explanation if I do not understand the science topic.
7. I will look for an explanation in the textbook if I do not understand the science topic.
8. I care about completing assignments in this class.
9. Getting a good grade in *biology* is important to me.
10. I am interested in understanding the teacher in this class.
11. The *biology* I learn is relevant to my life.
12. Learning *biology* is interesting.
13. Learning *biology* makes my life more meaningful.
14. I am curious about discoveries in *biology*.
15. I enjoy learning *biology*

Factor # 4 *Benefit and Utility of biology*
16. I use the *biology* that I learn in school in my life.
17. What I learn in my *biology* class helps me understand how things work in life.
18. Learning *biology* makes me curious about things that I observe in my life.
19. What we learn in *biology* class helps me to understand how *biology* affects my life.
20. Learning *biology* helps me to make wiser decisions about my lifestyle and health.

Factor # 5 *Career Motivation*
21. Learning *biology* will help me get a good job.
22. Knowing *biology* will give me a career advantage.
23. Understanding *biology* will benefit me in my career.
24. My career will involve science.

25. I will use *biology* problem-solving skills in my career

Factor # 6 *Self-Efficacy in biology Learning*
27.If I study hard I can do well in *biology*
28.I believe *biology* is too easy for me to learn
29.The idea of taking *biology* makes me excited.
30.I am confident I will do well on *biology* tests.
31.I am confident I will do well on *biology* labs and projects.
32.I believe I can master *biology* knowledge and skills.
33.I believe I can earn a grade of "A" in *biology*.
34.I am sure I can understand *biology*.

Factor # 7 *Self-Determination*
35. I put enough effort into learning *biology*.
36. I use strategies to learn *biology* well.
37. I spend a lot of time learning *biology*.
38. I prepare well for *biology* tests and labs.
39. I study hard to learn *biology*.

Factor # 8 *Grade Motivation*
40. I like to do better than other students on *biology* tests.
41. Getting a good *biology* grade is important to me.
42. It is important that I get an "A" in *biology*.
43. I think about the grade I will get in *biology*.
44. Scoring high on *biology* tests and labs matters to me.

Factor # 9 *Assessment anxiety*
45. I am nervous about how I will do on the *biology* tests.
46. I become anxious when it is time to take a *biology* test.
47. I worry about failing the *biology* tests.
48. I am concerned that the other students are better in *biology*.
49. I hate taking the *biology* tests.
