# Supplementary material for: Measuring attitudes towards biology major and non-major: Effect of students’ gender, group composition, and learning environment
Source: PLoS One. 2021 May 14;16(5):e0251453. doi: 10.1371/journal.pone.0251453 (PMC8121319; doi:10.1371/journal.pone.0251453)
Supplement: S1 Questionnaire — (PDF) [file pone.0251453.s002.pdf]

Factor #1(Q1-14): Russel and Hollander, (1975) and Glynn et al (2007)

Factor #2 (Q1-48): Questionnaire Students' Attitudes Towards science (SATS) modified to biology course (SATB)

Likert-type scale 1 (Strongly disagree) 2(Disagree) 3(Neutral) 4(Agree) 5 (Strongly agree).

Sub-scale			
1	Feeling toward biology	1	Biology is very interesting to me.
		2	I have always enjoyed studying biology in school.
		3	I am always under a terrible strain in a biology class. *
		4	I feel a definite positive reaction to biology ; it's enjoyable.
		5	Biology makes me feel secure, and at the same time it is stimulating.
		6	I feel at ease in biology and like it very much.
		7	In general, I have a good feeling toward biology .
		8	I really like biology .
		9	biology is fascinating and fun.
		10	When I hear the word biology , I have a feeling of dislike.
		11	I approach biology with a feeling of hesitation.
		12	It makes me nervous to even think about doing a biology experiment. *
		13	Biology makes me feel uncomfortable, restless, irritable, and impatient. *
		14	I don't like biology , and it scares me to have to take it.
2	*General interest*	1	I like watching biology related TV.
		2	biology is my favorite subject in school.
		3	I like reading about famous biologiest
		4	I find what we learn in my biology class interesting.
		5	I would enjoy working in a biology lab.
3	*Motivation Towards Learning biology*	6	I will ask my teacher for an explanation if I do not understand the science topic.
		7	I will look for an explanation in the textbook if I do not understand the science topic.
		8	I care about completing assignments in this class.
		9	Getting a good grade in biology is important to me.
		10	I am interested in understanding the teacher in this class.
		11	The biology I learn is relevant to my life.
		12	Learning biology is interesting.
		13	Learning biology makes my life more meaningful.
		14	I am curious about discoveries in biology .
		15	I enjoy learning biology
4	*Benefit and Utility of biology*	16	I use the biology that I learn in school in my life.
		17	What I learn in my biology class helps me understand how things work in life.
		18	Learning biology makes me curious about things that I observe in my life.
		19	What we learn in biology class helps me to understand how biology affects my life.

		20	Learning biology helps me to make wiser decisions about my lifestyle and health.
5	*Career Motivation*	21	Learning biology will help me get a good job.
		22	Knowing biology will give me a career advantage.
		23	Understanding biology will benefit me in my career.
		24	My career will involve science.
		25	I will use biology problem-solving skills in my career
6	*Self-Efficacy in biology Learning*	26	If I study hard I can do well in biology
		27	I believe biology is too easy for me to learn
		28	The idea of taking biology makes me excited.
		29	I am confident I will do well on biology tests.
		30	I am confident I will do well on biology labs and projects.
		31	I believe I can master biology knowledge and skills.
		32	I believe I can earn a grade of "A" in biology .
		33	I am sure I can understand biology .
7	*Self-Determination*	34	I put enough effort into learning biology .
		35	I use strategies to learn biology well.
		36	I spend a lot of time learning biology .
		37	I prepare well for biology tests and labs.
		38	I study hard to learn biology .
8	*Grade Motivation*	39	I like to do better than other students on biology tests.
		40	Getting a good biology grade is important to me.
		41	It is important that I get an "A" in biology .
		42	I think about the grade I will get in biology .
		43	Scoring high on biology tests and labs matters to me.
9	*Aassessment anxiety*	44	I am nervous about how I will do on the biology tests. *
		45	I become anxious when it is time to take a biology test. *
		46	I worry about failing the biology tests. *
		47	I am concerned that the other students are better in biology *.
		48	I hate taking the biology tests. *

*.Reverse-scored
